# Neural Correlates of Vocal Pitch Compensation in Individuals Who Stutter

**DOI:** 10.3389/fnhum.2020.00018

**Published:** 2020-02-25

**Authors:** Anastasia G. Sares, Mickael L. D. Deroche, Hiroki Ohashi, Douglas M. Shiller, Vincent L. Gracco

**Affiliations:** ^1^Speech Motor Control Lab, Integrated Program in Neuroscience and School of Communication Sciences and Disorders, McGill University, Montreal, QC, Canada; ^2^Centre for Research on Brain, Language, and Music, Montreal, QC, Canada; ^3^Laboratory for Hearing and Cognition, Department of Psychology, Concordia University, Montreal, QC, Canada; ^4^Haskins Laboratories, New Haven, CT, United States; ^5^École d’orthophonie et d’audiologie, Université de Montréal, Montreal, QC, Canada

**Keywords:** stuttering, pitch, vocalization, altered feedback, fMRI, speech, sensorimotor

## Abstract

Stuttering is a disorder that impacts the smooth flow of speech production and is associated with a deficit in sensorimotor integration. In a previous experiment, individuals who stutter were able to vocally compensate for pitch shifts in their auditory feedback, but they exhibited more variability in the timing of their corrective responses. In the current study, we focused on the neural correlates of the task using functional MRI. Participants produced a vowel sound in the scanner while hearing their own voice in real time through headphones. On some trials, the audio was shifted up or down in pitch, eliciting a corrective vocal response. Contrasting pitch-shifted vs. unshifted trials revealed bilateral superior temporal activation over all the participants. However, the groups differed in the activation of middle temporal gyrus and superior frontal gyrus [Brodmann area 10 (BA 10)], with individuals who stutter displaying deactivation while controls displayed activation. In addition to the standard univariate general linear modeling approach, we employed a data-driven technique (independent component analysis, or ICA) to separate task activity into functional networks. Among the networks most correlated with the experimental time course, there was a combined auditory-motor network in controls, but the two networks remained separable for individuals who stuttered. The decoupling of these networks may account for temporal variability in pitch compensation reported in our previous work, and supports the idea that neural network coherence is disturbed in the stuttering brain.

## Introduction

Persistent developmental stuttering is a neurobiological disorder that results in the repetition and prolongation of speech sounds, syllables, and words (Bloodstein and Bernstein Ratner, 2008). It has been suggested that, on a neural level, stuttering is the result of a problem with sensorimotor integration (Max et al., 2004). Consistent with this idea is the observation that individuals who stutter do not respond to altered auditory feedback in the same way as fluent individuals during vocalization (Kalinowski et al., 1993; Bauer et al., 2007; Cai et al., 2012; Loucks et al., 2012; Daliri et al., 2018). We recently showed that individuals who stutter are more variable in responding to manipulations of pitch feedback while speaking, both in the number of compensatory responses and in the timing of those responses, and that this variability correlates with self-rated stuttering severity (Sares et al., 2018). The results of this and other behavioral studies (Kalinowski et al., 1993; Cai et al., 2014) point to a timing problem during auditory-motor behavior, something that also appears to extend to non-speech (Cooper and Allen, 1977; Ward, 1997; Boutsen et al., 2000; Subramanian and Yairi, 2006; Falk et al., 2015; van de Vorst and Gracco, 2017; Sares et al., 2019). Neuroimaging studies have identified differences in motor and auditory regions of the brain in adults who stutter (AWS; Foundas et al., 2001; Brown et al., 2005; Nil et al., 2008; Chang et al., 2009; Kell et al., 2009; Beal et al., 2010, 2011; Kikuchi et al., 2011; Budde et al., 2014; Belyk et al., 2015). Taken together, these behavioral and neuroimaging studies are consistent with compromised sensorimotor feedback interactions during speech production. In the current experiment, we will explore the neural processes underlying altered pitch feedback compensation in individuals who stutter using fMRI.

Several fMRI studies using pitch-altered feedback have been conducted on individuals with typical speech development (Watkins et al., 2005; Toyomura et al., 2007; Zarate and Zatorre, 2008; Zarate et al., 2010; Parkinson et al., 2012; Behroozmand et al., 2015). The regions involved are similar to those of delayed auditory feedback studies (Hashimoto and Sakai, 2003; Watkins et al., 2005), generating more activity in temporal areas during altered auditory feedback compared to normal feedback. Motor activation is less consistent, ranging from prefrontal and premotor (Toyomura et al., 2007) to the supplementary motor and primary motor areas (Zarate and Zatorre, 2008). In some cases, motor activation is not seen in the main contrast (Parkinson et al., 2012; Behroozmand et al., 2015).

In individuals who stutter, only one fMRI study by Watkins et al. (2008) examined altered pitch feedback. They had participants speak in the scanner and continuously played back their speech to them as auditory feedback. A consistent pitch shift was applied for all trials in one block, compared to another block where no shift was applied. This was intended to be a fluency-enhancing condition, and it was predictable in that the shift lasted for the entire block. The shift was also quite large (six semitones) and may not have been interpreted as the participants’ own voice. Thus, the manipulation used by Watkins et al. (2008) may have recruited additional processes (cognitive and attentional) and recruited brain areas associated with short-term sensorimotor learning. In contrast, our pitch compensation paradigm uses unpredictable and subtle shifts, allowing for a better estimation of on-line sensorimotor control processes. We can nevertheless predict that some common areas would be activated in a pitch-compensation experiment: namely, premotor/sensorimotor cortex, auditory cortex, and perhaps cerebellum. Some predictions about the stuttering brain’s response to pitch-altered feedback may also be made based on fMRI studies of delayed feedback in stuttering, which usually show that individuals who stutter differ in their recruitment of superior and middle temporal gyrus, as well as inferior frontal gyrus (Watkins et al., 2008; Sakai et al., 2009).

However, there is also evidence that neural differences in individuals who stutter may go beyond levels of activity in specific brain regions, additionally affecting connectivity between brain regions. Recent resting-state connectivity analyses suggest atypical functional brain organization in stuttering (Lu et al., 2009, 2010; Xuan et al., 2012; Chang et al., 2018), and white matter structure also seems to be affected (Jäncke et al., 2004; Watkins et al., 2008; Blecher et al., 2016; Kemerdere et al., 2016; Kronfeld-Duenias et al., 2016). Functional MRI analysis techniques like independent component analysis (ICA) can identify brain “networks” from fMRI data without resorting to seed regions or other *a priori* hypotheses. This whole-brain, data-driven approach separates coherent networks of voxels based on statistical patterns in the data. Data can be examined based on the number and type of networks, and which networks correlate with the time course of the task (Calhoun et al., 2001, 2004; Xu et al., 2013; Geranmayeh et al., 2014).

The current study was designed to investigate neural correlates of auditory-motor integration in AWS using an altered pitch feedback task. A General Linear Model (GLM) analysis identifies the brain regions associated with processing shifted vs. unshifted pitch for both AWS and fluent adult controls (AC). In order to obtain a more detailed neural picture of the manner in which AWS and AC accomplished the task, we also employed a spatial ICA analysis (sICA), uncovering additional differences between the two groups.

## Materials and Methods

### Participants

Thirteen AWS and 15 fluent AC participants took part in the experiment (AWS: eight females/five males; AC: 10 females/five males), with both groups ranging in age from 18 to 51 years (mean 29.46 ± 11.19 years). Initial recruitment took place through advertisements, word of mouth, and contacting previous participants from other studies. Nineteen individuals with a stutter underwent the behavioral study (Sares et al., 2018), and 13 elected to go on to the MRI study. Others declined due to scheduling, personal preference, and medical concerns like claustrophobia. Eighteen of the nineteen control participants were willing to perform the MRI; we selected those who created the best-matched group, testing two additional participants for this purpose. The sample size was determined primarily by the ability to recruit local participants who stuttered.

An individual who stutters is usually classified based on one of two criteria: a previous diagnosis or a blind evaluation of a speech clip by a speech-language pathologist. Three of the 13 AWS did not meet either of these two criteria but were included in the present study as self-identified individuals who stutter. Stuttering participants also rated their own stuttering in terms of severity and anxiety on a 9-point Likert scale (O’Brian et al., 2004; Karimi et al., 2014). Self-rated stuttering severity, self-rated anxiety about stuttering, and speech-language pathologist ratings (Stuttering Severity Instrument, 4th edition) are presented in Table 1. Each of the 13 individuals who stutter was matched to a control participant in sex and age within 5 years (mean age difference per pair = 0.38 ± 2.79 years, with no group difference in age: two-tailed *t*_(26)_ = 0.301, *p* = 0.766, Cohen’s *D* = 0.11). Participant groups were also balanced in terms of handedness (with one female in each group being left-handed), as measured by the Edinburgh Handedness Inventory (Oldfield, 1971) and in terms of music experience based on a modified version of the Montreal Music History Questionnaire (Coffey et al., 2011; *t*_(26)_ = −0.574, *p* = 0.571, Cohens *D* = −0.22 for handedness; *t*_(26)_ = −0.301, *p* = 0.765, Cohen’s *D* = −0.11 for log hours of music experience). This study was approved by the McGill Faculty of Medicine Institutional Review Board in accordance with principles expressed in the Declaration of Helsinki; informed written consent was obtained from all participants.

**Table 1:** Characteristics of self-identified participants with a stutter.

Participant	SSI-4 score	SLP classification	Self-rated severity	Self-rated anxiety
1	23	AWS	5	3
2	29	AWS	7	3
3	32	AWS	5	3.5
4	0	AC	2	4
5	13	AWS	3.5	5
6	0	AC	2.5	4
7	0	AC (but previously in therapy)	3	3
8	25	AWS	4	4
9	29	AWS	6.5	7.5
10	26	AWS	7.5	6
11	8	AC	3	1
12	14	AWS	4	4.5
13	22	AWS	4.5	4.5

SSI-4: Stuttering Severity Instrument, 4th edition. SLP Classification: speech-language pathologist’s classification. Self-rated severity: from 1 to 9, 1 being “no stuttering” and 9 being “very severe stuttering.” Self-rated anxiety: from 1 to 9, 1 being “no anxiety” and 9 being “very severe anxiety.”

### Task

The task involved vocalizing while simultaneously hearing one’s own voice through headphones. The auditory feedback was intermittently pitch-shifted and the participant’s vocal response was recorded (Fairbanks, 1955; Yates, 1963; Elman, 1981; Burnett et al., 1998; Houde and Jordan, 2002; Stuart et al., 2002; Liu and Larson, 2007). Participants were instructed to vocalize the vowel /a/ for 74 trials, a task with which they were already familiar, all of them having completed a separate out-of-scanner behavioral session (Sares et al., 2018). There was a partial overlap between the participants whose out-of-scanner data appeared in the previous report (Sares et al., 2018) and the participants whose MRI data appears here, depending on the inclusion criteria for the different studies and whether participants came back for the MRI session.

Participants heard their own voice through the headphones along with pink noise in order to minimize the participants’ bone-conducted feedback. On 26 of the trials, their voice was unaltered (unshifted trials). On the remaining 48 trials, the participants heard their voice briefly perturbed (shifted) by 100 cents, either up (24 trials) or down (24 trials) for a duration of 500 ms. A few participants in each group had truncated scans and thus had fewer than 74 trials (AC: 72, 63, and 62 trials; AWS: 69, 57, and 61 trials). Vocalizations all had a duration of at least 1.4 s. In the previous experiment, participants had been trained to maintain a consistent volume while vocalizing. The onset of the pitch shift was jittered between 350 and 800 ms after vocalization onset detection, to avoid unstable pitch at the beginning of the vocalization and also to make the shift less predictable.

The order of unshifted/up-shift/down-shift trials was randomized, with the constraint that there could be no more than two consecutive unshifted trials, and no more than four consecutive trials shifted in the same direction. An image appeared on the screen indicating the beginning of a trial, and a progress bar began at the bottom of the screen when the vocalization was detected. Participants were instructed to keep vocalizing until the progress bar was filled for each trial but were not informed that there would be shifts in the pitch. The task took about 15 min to complete. It is important to mention that this experiment was not designed to induce stuttering, but rather to study how the trait of stuttering affects basic auditory-motor processing during periods of fluent vocal production.

### MRI Procedure

Testing took place at the Montreal Neurological Institute. Scanning was performed on a Siemens Trio 3T scanner with a 32-channel head coil. A high-resolution T1-weighted anatomical scan was first acquired with an MPRAGE ADNI iPAT2 sequence (voxel size = 1 mm^3^; TR = 2.30 s; TE = 2.98 ms; flip angle = 9°; FOV read = 256 mm).

Immediately after the anatomical scan, a T2*-weighted functional resting-state scan measuring a blood-oxygen-level-dependent signal (BOLD) took place (voxel size = 3 mm^3^; TR = 2.68 s; TE = 30 ms; flip angle = 90°; FOV read = 192 mm). The participant was presented with a black + sign in the middle of a white screen and told to fixate on it while remaining relaxed and not thinking of anything in particular. This lasted for 369 s.

**Figure 1:**
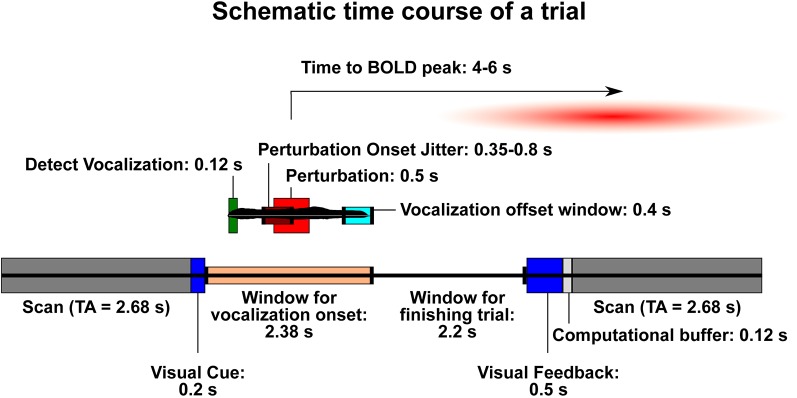
Schematic time course of a trial. The lower bar represents events that are not related to the onset of vocalization in each trial. The smaller bar above it (with a sample vocal trace in black) represents the trial time course once vocalization has begun. Trials are designed such that the blood-oxygen-level-dependent signal (BOLD) signal resulting from the perturbation will be likely to peak during the following acquisition. Dark gray: acquisition scan (brain activity recorded). TA, acquisition time (2.68 s). Dark blue: visual displays for trial onset and feedback. Peach: window of time during which a participant may begin vocalization. Light gray: buffer time necessary to process the trial and be ready for the next one. Dark green: time needed for the Audapter software to recognize vocalization. Maroon: time frame within which the perturbation will begin on a shifted trial (jittered). Red: duration of perturbation (always 500 ms). Turquoise: time window in which participants should stop vocalization in order to receive “success” during visual feedback.

For the speech task, a sparse-sampling paradigm was used (Belin et al., 1999; Gracco et al., 2005; Perrachione and Ghosh, 2013), with the same MRI acquisition protocol (voxel size = 3 mm^3^; TE = 30 ms; flip angle = 90°; FOV read = 192 mm) except for a repetition time (TR) of 8.08 s that was greater than the acquisition time (TA) of 2.68 s (Figure 1). The trial presentation occurred during the 5.4 s between volumes, assuring that scanner noise did not interfere with the auditory feedback, and that jaw motion during vocalization did not contaminate the MR signal. An MR-compatible microphone was used, along with ear inserts to deliver the feedback. The level of a test sound was used to adjust the volume to a comfortable level for the participant. In the experiment, this resulted in a sound pressure level of approximately 70–80 dB for the pink noise alone, and 80–88 dB for pink noise and vocal feedback together.

### Analysis

#### Behavioral Analysis

A pitch trace in Hertz was obtained for each trial using PRAAT’s PSOLA algorithm (Boersma and Weenink, 2013), and data were imported into Matlab R2015a (MATLAB, 2015). For additional details about the pitch trace preprocessing, see our report on the out-of-scanner data (Sares et al., 2018). Pitch traces were aligned at the moment of the perturbation, or for unshifted trials, a random moment when the perturbation could have occurred. Pitch was converted to cents relative to the moment of the perturbation using the following equation:

$$cents=1200\times log_{2}\left(\frac{frequency}{frequency_{\text{PertOnset}}}\right)$$

To control for some participants’ tendency to rise or fall in pitch over the course of a trial, each participant’s unshifted trials were averaged together, and this characteristic pitch trace was subtracted from the pitch-shifted trials to create normalized pitch traces.

We then calculated an average compensation curve per participant, per shift direction. Based on response data from our previous behavioral study (Sares et al., 2018), we chose a window of 150–650 ms after the shift onset to examine the response. We took the area under the curve during this window. This information was submitted to a 2 × 2 ANOVA (group, shift direction). Since no differences by direction were found (see “Results” section), we could compute an average compensation curve across both shift directions, with responses to up-shift trials flipped so that they could be averaged with down-shift trials. The grand average responses for each group were submitted to a one-sample *t*-test (one-tailed) to confirm that they were significantly greater than zero, meaning that compensation behavior was present for both groups.

In addition, we ran a 2 × 2 ANOVA (group, shift direction) on onset and peak time variability of the responses, as described in the previous study (Sares et al., 2018). Effect size for ANOVAs was measured using generalized eta squared ($\eta_{\text{G}}^{2}$; Olejnik and Algina, 2003; Bakeman, 2005).

#### MRI Preprocessing

Preprocessing was realized in SPM12 software (Wellcome Department of Imaging Neuroscience, London, UK) in Matlab. The initial volume(s) were not included in analyses; they were automatically removed during the Siemens scan sequences to allow the magnetization to stabilize. The functional images from each participant’s session were first motion-corrected and then co-registered to each individual’s anatomical file. A transformation matrix from the anatomical image to a standard image (MNI152 T1 average) was estimated and then applied to each functional image to facilitate comparisons across participants. Finally, the preprocessed functional images were spatially smoothed with an 8-mm full-width at half-maximum, and time-course data were high-pass filtered at 128 s. Mango^1^ was used for visualization of data.

#### General Linear Model (GLM) Analysis

The GLM approach was used to examine the neural response to the pitch shift and differences in the response between AWS and AC by subtraction of signals. First level (within-participant) analyses were performed in SPM12 software. The regression model for each participant included unshifted and shifted conditions (upshifts and downshifts were combined), and a proxy measure for the behavioral response to a shift, which was orthogonalized to the “shifted” regressor. Rigid-body movement parameters were included as covariates of no interest: x, y, and z translation, as well as pitch, roll, and yaw rotation. This GLM yielded a first-level shifted > unshifted contrast on a per-subject basis, as well as a contrast of all conditions vs baseline. Baseline consisted of scans before and after the experiment, dropped trials where insufficient vocalization was detected and scans where a trial did not initiate because the computer was still calculating. Because the contrast of interest was shifted vs. unshifted trials, there were not many baseline scans for some participants, so this contrast should be interpreted with caution.

At the second level, the neural response to pitch perturbations was defined as regions where BOLD activity in the shifted condition differed from the unshifted condition. Specifically, the shifted > unshifted contrast was tested by permutation, implemented in FSL software’s non-parametric *randomise* function (Winkler et al., 2014) with threshold-free cluster enhancement (Smith and Nichols, 2009; corrected two-tailed *p* < 0.05). The all conditions > baseline contrast was similarly calculated.

The difference in the neural response to the pitch shift between AWS and AC was also tested. We compared the shifted > unshifted contrast between the two groups by unpaired two-sample *t*-test, also implemented using the *randomize* function. There were no significant differences at the corrected threshold, but we present effects identified at a more lenient threshold (uncorrected voxel-wise two-tailed *p* < 0.001).

For each cluster found in the group difference contrast, *post hoc* correlations with self-rated stuttering severity were performed with the MarsBar toolbox^2^ (Brett et al., 2002). The values input to the correlation were the average beta values from the shifted > unshifted contrast across the cluster, and each individual’s self-rated stuttering severity. Two right frontal clusters in Brodmann area 10 (BA 10) were combined because of their proximity and small size (see “Results” section). Raw p-values are reported, which should be evaluated with a Bonferroni correction (alpha of 0.05 divided by two regions evaluated, giving a new alpha of 0.025).

#### Independent Component Analysis (ICA)

Task-related neural activity across distributed networks was assessed by ICA followed by GLM. Task-related patterns were obtained by applying GLM analysis to the spatiotemporal patterns estimated by ICA using GIFT software (Calhoun et al., 2001, 2004).

Group ICA was run on all participants together, as well as on each group individually. The ICA analysis on all participants allowed us to have the same components across groups so that we could perform group comparisons on the properties of the independent components (ICs). Separate application of ICA on the two groups allowed us to detect spatiotemporal patterns specific to each group.

In the ICA analysis, the time series of each voxel of the preprocessed data was normalized by its average intensity. The normalized data passed through a two-step data reduction by principal component analysis (PCA). In the first step, the data were reduced to 45 dimensions on a per-subject basis. In the second step, the processed data for all subjects were concatenated across time, and the concatenated result was then reduced to 30 dimensions. After these reductions, the number of ICs was estimated using the *Infomax* algorithm (Bell and Sejnowski, 1995) for all participants together. This yielded a spatial map and a time course for each IC. Since the estimation of ICs can vary slightly each time it is run, we repeated the ICA 30 times using the ICASSO toolbox and found reproducible ICs (Himberg et al., 2004). Finally, spatial maps and time courses of ICs at the group level were back-reconstructed to those for each subject.

Spatial maps of ICs shown in subsequent figures reflect a per-voxel permutation test where the beta weights of the component in participants’ back-reconstructed maps were significantly greater than zero, again using the *randomize* algorithm (corrected two-tailed *p* < 0.001, using threshold-free cluster enhancement). The threshold was increased to corrected *p* < 0.001 in order to restrict the spatial extent of the networks and make sure that any overlaps observed with other networks were relatively small. The same procedure was carried out for both task and resting-state data. Task data yielded 28 ICs for all participants together, and 29 ICs for both of the separate group analyses. Resting-state data yielded 33 ICs for all participants together, and 35 ICs for both of the separate group analyses.

The ICs then passed to an identification stage. Two raters (first author and a lab trainee who was blind to task and group) identified components of interest and eliminated ICs related to factors such as respiration, pulse, and scanner artifacts by examining both the spatial and frequency distributions (Griffanti et al., 2017). The mean agreement between the two raters over rest and task data was 94.38 ± 3.43%. Components identified as not-of-interest or unsure by both raters were removed from the analysis. For contested decisions, where one rater classified the component as not-of-interest and the other counted it as of-interest, the component was kept. For the task data across all participants, 13 out of 28 ICs were kept. For the group with a stutter, 16 of 29 ICs were kept, and for the control group, 13 of 29 ICs were kept. For the resting-state data across all participants, 22 out of 33 ICs were kept. For the group with a stutter, 21 of 35 ICs were kept, and for the control group, 21 of 35 ICs were kept. A number of accepted ICs between 10 and 20 are comparable to other recently published work (Rummel et al., 2013; Griffanti et al., 2014), regardless of how many ICs are initially identified.

Multiple regression was performed with the time courses of the experiment conditions (unshifted, up-shift, and down-shift) entered as predictors for each of the IC time courses. For the remaining components, statistics were carried out on the beta weights in the form of 2 × 3 ANOVA (group, condition). ANOVA results were corrected with a false discovery rate (FDR) to account for the total number of ANOVAs performed in that group of ICs.

To associate the spatial distribution of the task-based functional networks identified by ICA with resting-state networks, we derived Tanimoto Indices (also known as Jaccard Indices) by comparing each task-based network to all resting-state networks (Wang and Peterson, 2008; Qiao et al., 2017). Overlaps of the top five task-related networks for each group (all participants, AC, AWS) with resting-state networks are presented in Supplementary Tables S1–S3. Finally, based on the involvement of auditory and motor networks in the task, functional network connectivity over the entire time course was assessed between auditory and motor networks in both rest and task data using the Dynamic FNC (dFNC) toolbox within GIFT (Allen et al., 2011).

## Results

### Behavior

**Figure 2:**
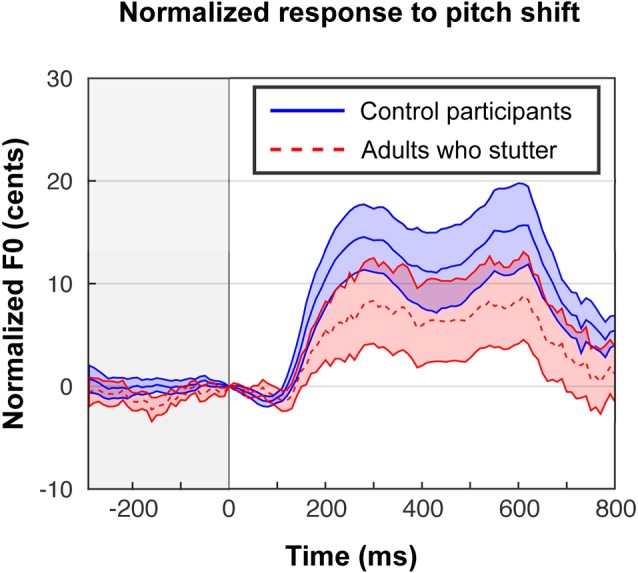
In-scanner behavioral responses to shifted trials in cents, exhibiting a typical compensation effect, although the magnitude of the effect was reduced compared with out-of-scanner responses (Sares et al., 2018). Trials are centered at the moment of the perturbation, and responses to up-shifts are flipped in order to be averaged with down-shifts. Each participant’s shifted trials are normalized by subtracting their characteristic pitch trace in the unshifted trials. Shaded areas show standard error of the mean.

In our previous behavioral study (Sares et al., 2018), we obtained compensation magnitudes around 20–30 cents. Behavioral compensation in the scanner (Figure 2) was smaller—about 10–20 cents—but nonetheless comparable to the results from Parkinson et al. (2012), who conducted the same in-scanner task for fluent participants. The proportion of trials that led to the expected response (opposing the shift) varied widely by each participant (minimum 30%, maximum 93%).

#### Area Under the Average Response Curve

A one-sample *t*-test (one-tailed) on areas under the curve for shifted trials revealed the expected compensation behavior in both groups (AC: *t*_(14)_ = 3.9, *p* < 0.001, Cohen’s *D* = 1.00; AWS: *t*_(12)_ = 1.8, *p* = 0.047, Cohen’s *D* = 0.50). Though an attenuated average response in AWS compared to AC is visually evident, the group difference in area under the curve did not reach significance, whereas it had in the out-of-scanner data (*F*_(1,26)_ = 1.5, *p* = 0.235, $\eta_{\text{G}}^{2}$ = 0.04). There was no effect of shift direction on the magnitude of responses (*F*_(1,26)_ = 0.02, *p* = 0.895, $\eta_{\text{G}}^{2}$ < 0.01) and no interaction between group and shift direction (*F*_(1,26)_ = 0.46, *p* = 0.505, $\eta_{\text{G}}^{2}$ = 0.01).

#### Variance in Onset Time

Though onset time variability (as measured in standard deviation) was less in AC (142.6 ms) than in AWS (159.9 ms), there was no statistically significant main effect of group (*F*_(1,26)_ = 1.7, *p* = 0.199, $\eta_{\text{G}}^{2}$ = 0.03), no main effect of direction (*F*_(1,26)_ = 2.3, *p* = 0.138, $\eta_{\text{G}}^{2}$ = 0.05), and no interaction (*F*_(1,26)_ < 0.1, *p* = 0.858, $\eta_{\text{G}}^{2}$ < 0.01).

#### Variance in Peak Time

Peak time variability (as measured in standard deviation) was slightly less in AC (170 ms) than in AWS (175.2 ms). There was no statistically significant main effect of population (*F*_(1,26)_ = 0.2, *p* = 0.685, $\eta_{\text{G}}^{2}$ < 0.01), no effect of direction (*F*_(1,26)_ = 3.0, *p* = 0.095, $\eta_{\text{G}}^{2}$ = 0.05), and no interaction (*F*_(1,26)_ = 0.1, *p* = 0.735, $\eta_{\text{G}}^{2}$ < 0.01).

The lack of statistical significance in these results could be due to the inclusion of individuals with a milder stutter compared to the previous experiment, or (more likely) differences in the auditory environment of the scanner, where responses to altered auditory feedback seem to be attenuated. However, even when behavior is not noticeably different, neural processing can differ, as we will show.

### MRI—GLM

**Figure 3:**
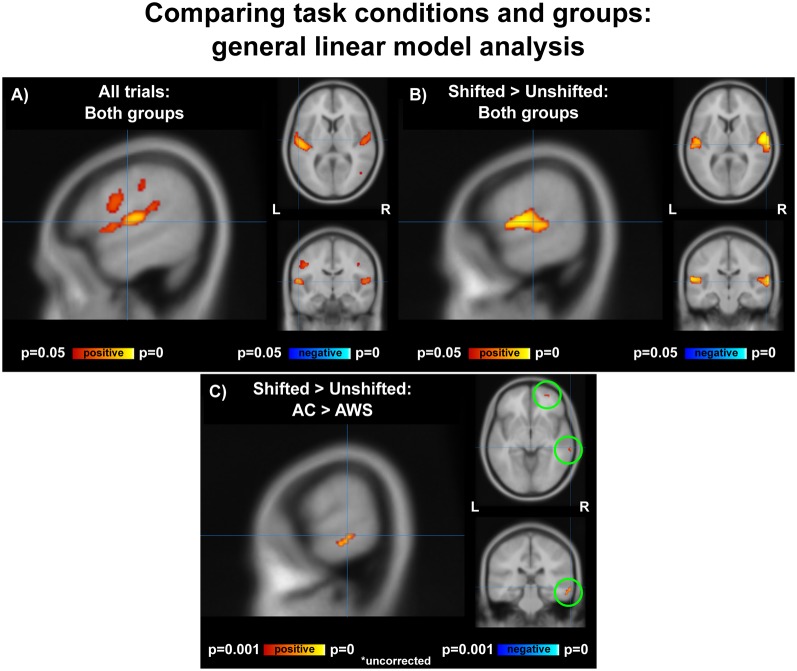
**(A)** Activation over all trials for all participants, corrected *p* < 0.05. **(B)** Shifted > unshifted trials for all participants, corrected *p* < 0.05. **(C)** AC > AWS for shifted > unshifted; uncorrected *p* < 0.001. AC, adult control participants; AWS, adults who stutter.

**Figure 4:**
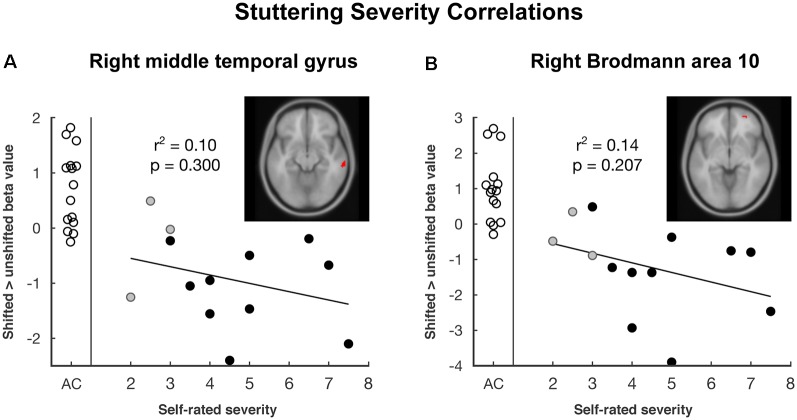
The plot of pitch-shift related activation by self-rated stuttering severity in areas where there was a group difference: **(A)** right middle temporal gyrus and **(B)** right Brodmann area 10. Empty circles represent adult control participants (AC), black and gray circles represent adults who stutter (AWS). Gray circles are self-identified AWS whose stuttering was not confirmed by speech-language pathologist evaluation. The trend line and correlation are shown within the AWS group.

Vocalization resulted in auditory and motor activity (Figure 3A, Table 2). Pooling all participants together, we observed increased BOLD activity in the superior temporal cortices for shifted trials compared to unshifted trials (Figure 3B, Table 2). Additionally, there were two regions where the groups differed in their responses to shifted vs. unshifted trials at an uncorrected threshold (*p* < 0.001; Figure 3C, Table 2). These were the right middle temporal gyrus (rMTG), and the frontal BA 10. AC had greater activity in these areas than AWS. In fact, almost all of the individuals who stuttered showed lower activity in the shifted condition than in the unshifted (negative y values in Figure 4), whereas controls almost all show positive values.

**Table 2:** General linear model analysis: peak tables.

*X*	*Y*	*Z*	Approximate region label	Brodmann area	T	Voxels
			**All trials: both groups together**			
−42	−30	12	L Auditory cortex	41	5.84	1,379
54	−12	8	R Auditory cortex	13/22	4.55	468
−36	−38	42	L Intraparietal sulcus	40	4.04	223
−60	−24	36	L Inferior parietal lobe	40	4.17	87
46	−66	6	R Middle occipital gyrus	37	4.93	25
60	6	24	R Premotor cortex	6	3.85	23
42	−12	36	R Primary motor cortex	4	4.53	10
			**Shifted > Unshifted: both groups together**			
58	−12	8	R Auditory cortex	41	6.95	776
−60	−22	12	L Auditory cortex	41	6.67	617
			**Shifted > Unshifted: AC > AWS**			
66	−30	−6	R Middle temporal gyrus	21	3.44	57
34	62	6	R Superior frontal gyrus	10	4.22	10
30	54	−6	R Middle frontal gyrus	10	2.71	5

*Top: activation over all trials (vocalization vs. silence) for all participants, corrected p < 0.05. Middle: shifted > unshifted trials for all participants, corrected p < 0.05. Bottom: AC > AWS for shifted > unshifted, uncorrected p < 0.001. X, Y, and Z coordinates are in mm, based on MNI 152 space. Clusters containing less than five voxels are not reported. L/R indicate left-or right-sided laterality, respectively. AC, adult control participants; AWS, adults who stutter*.

Looking at these two regions to see whether there was also a relationship with self-rated stuttering severity, this relationship did not reach significance (Figure 4; MTG: *r*^2^ = 0.10, *p* = 0.300; BA 10: *r*^2^ = 0.14, *p* = 0.207). Correlations with SSI scores were similar; these can be found in the Supplementary Figures S4.1, S4.2, and S7.2.

### MRI—ICA

#### ICA ANOVA for All Participants

**Figure 5:**
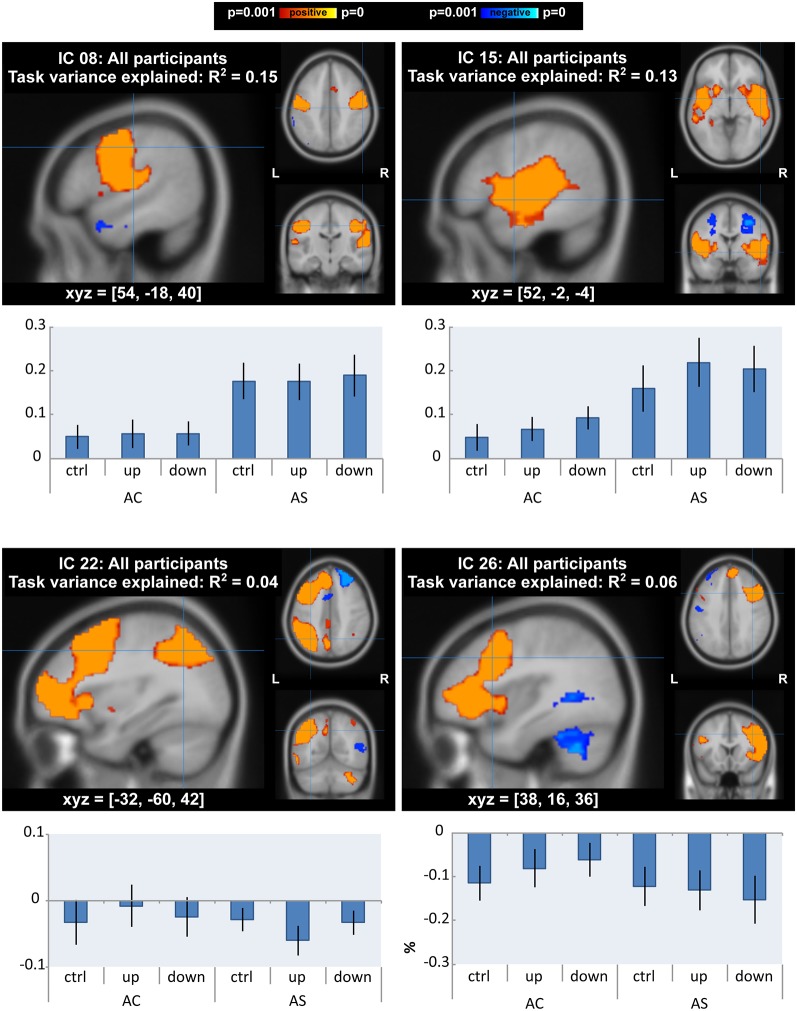
Independent component analysis (ICA) with all participants: components that showed a main effect or interaction between groups/conditions. Spatial maps of components are *p* < 0.001, corrected for multiple comparisons and using threshold-free cluster enhancement). The top two components (IC 8 and 15) also accounted for the most task variance. Below each map is a bar graph of the mean percent signal change by group and condition within each network.

ICA yielded 13 task-related ICs for data from AWS and AC taken together. There were differences of interest in motor, auditory and fronto-temporoparietal networks (IC 8, IC 15, IC 22 and IC 26 in Figure 5 and Supplementary Figure S1, Table S1) in which task- and group-related differences were detected in GLM analysis (see “GLM Analysis” section). IC 8, which accounted for 15% of the variance of the task-related neural activity, was a motor network that extended into auditory areas. IC 15, which accounted for 13% of variance, was a bilateral superior temporal network that also extended into the basal ganglia. IC 22 and IC 26 were left and right fronto-temporoparietal networks. These networks included inferior and middle frontal areas (including BA 10), middle temporal areas (including the MTG), and inferior parietal areas, as well as the anterior cingulate, precuneus, and cerebellar Crus I, II, and VI. We tested differences in the activity of the networks between AWS and AC or over conditions using ANOVA.

In line with the GLM results, there was a significant effect of condition on neural activity of the auditory network (IC 15: *F*_(2,52)_ = 7.41, FDR-corrected *p* = 0.019, $\eta_{\text{G}}^{2}$ = 0.02). *Post hoc* tests with a Tukey correction detected significant differences between each shifted condition and the unshifted condition (down-shift vs. unshifted: *t*_(52)_ = 3.52, *p* = 0.003, Cohen’s *D* = 0.28; up-shift vs. unshifted: *t*_(52)_ = 3.11, *p* = 0.009, Cohen’s *D* = 0.23).

For the AWS group, auditory and motor networks were slightly over-activated across all conditions (IC 8: *F*_(1,26)_ = 6.31, *p* = 0.018, $\eta_{\text{G}}^{2}$ = 0.19; IC 15: *F*_(1,26)_ = 4.89, *p* = 0.036, $\eta_{\text{G}}^{2}$ = 0.15); however, these group differences did not survive FDR correction (FDR-corrected *p* = 0.234). There were no interactions between group and condition in the auditory and motor networks.

In the right and left fronto-temporoparietal networks, AC and AWS diverged under conditions of the shifted pitch. This was evidenced by a significant interaction between group and condition in the left fronto-temporoparietal network (IC 22: *F*_(2,52)_ = 6.43, FDR-corrected *p* = 0.042, $\eta_{\text{G}}^{2}$ = 0.01). Activity in this network seemed to be more negatively related to shifted pitch for adults with a stutter, specifically in the up-shift condition (*post hoc* up-shift vs. unshifted in AWS: *t*_(52)_ = −2.61, *p* = 0.031, Cohen’s *D* = 0.43). A similar trend was also present for the interaction between group and condition in the right homolog of this network, again with slightly more negative values for the shifted condition in AWS (IC 26: *F*_(2,52)_ = 4.51, FDR-corrected *p* = 0.102, $\eta_{\text{G}}^{2}$ = 0.01). Activity in the right-lateralized network was less negative in the down-shift condition in AC (*post hoc* down-shift vs. unshifted in AC: *t*_(52)_ = 2.80, *p* = 0.019, Cohen’s *D* = 0.35). Notably, this right-lateralized network contained a portion of the posterior middle temporal gyrus, immediately posterior to the peak where GLM differences were found between AC and AWS (Table 2; Shifted > Unshifted; AC > AWS).

#### Comparing Task-ICA to Resting State

Next, we measured the overlap of the most task-related components with resting-state components using the Tanimoto index. Comparing task-based ICA with resting-state ICA allowed us to see if the pitch compensation task induced the formation of networks with different spatial distributions than networks at rest. This analysis was performed for all participants together, and for each group separately. Networks were primarily motor, auditory, or default-mode in character; with AC showing a true auditory-motor network and an inferior frontal component while adults who stuttered had separate auditory and motor networks.

**Figure 6:**
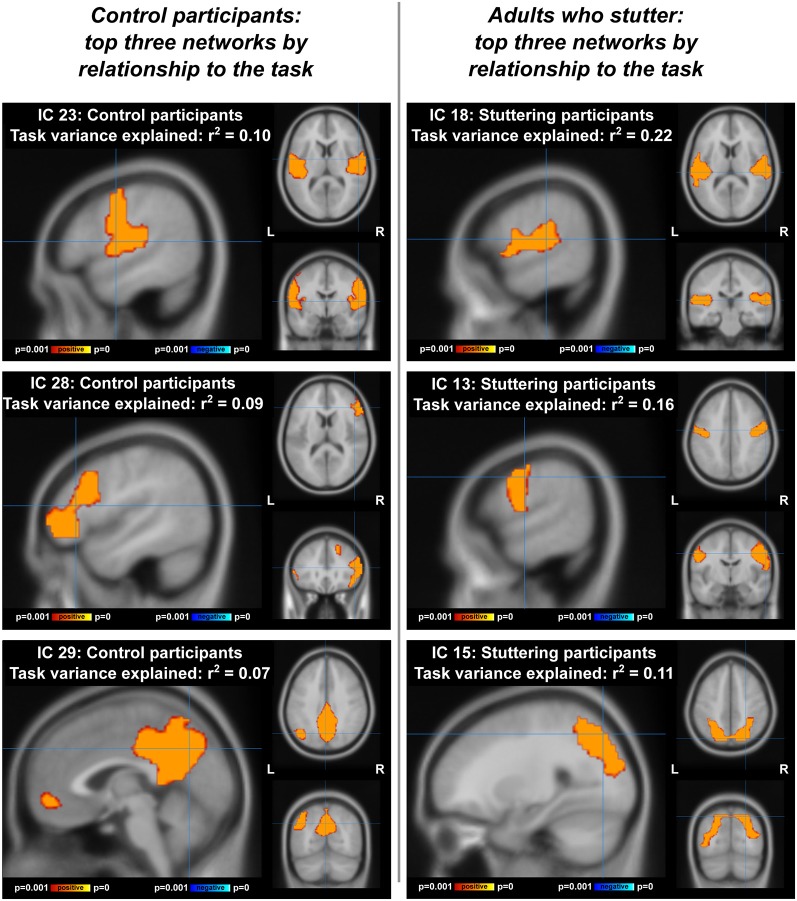
Top three networks, by relationship to the compensation task, for ICA performed separately on each group. IC, independent component. Left: adult controls (AC), at coordinates [54 −4 10] for IC 23, [48 30 12] for IC 28, and [0 28 42)] for IC 29. Right: adults with a stutter, at coordinates [58 −24 12] for IC 18, [60 −8 38] for IC 13, and [26 −74 48] for IC 15.

Visualizations of the top task-related components for AC and AWS separately can be found in Figure 6. In the ICA with AC only (Figure 6, left; Supplementary Figure S2, Table S2), the component accounting for most of the variance was distinctly auditory-motor in nature, overlapping with both a resting-state motor network (Tanimoto overlap = 0.326) and a resting-state auditory network (Tanimoto overlap = 0.322). The component accounting for the second greatest portion of the variance was a bilateral inferior frontal network (somewhat right-lateralized). Interestingly, the top two components of the ICA with individuals who stutter were an auditory and a motor component, still separate (Figure 6, right; Supplementary Figure S3, Table S3). These networks overlapped with only one resting-state network each—an auditory (Tanimoto overlap = 0.524) and a motor network (Tanimoto overlap = 0.6), respectively. Supplementary Tables S1–S3 provide more information, showing the top five components accounting for the greatest amount of variance in task-related neural activity for each sample, and the three resting-state networks that were the most spatially similar to each.

**Figure 7:**
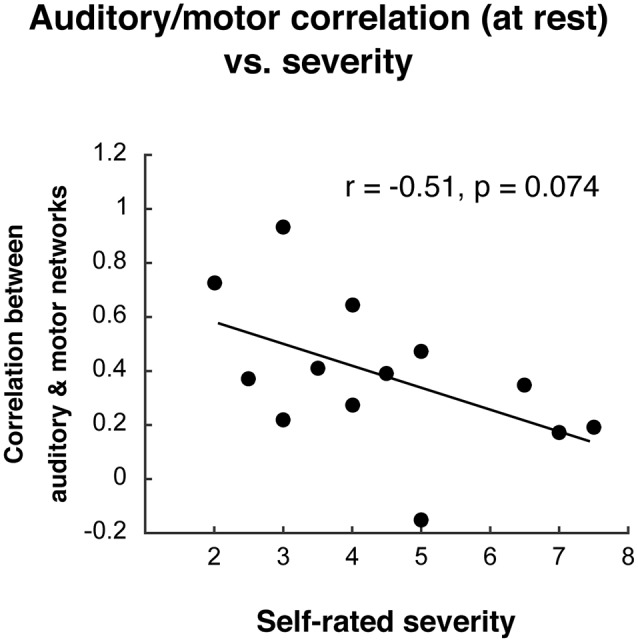
Relationship between auditory-motor connectivity at rest (fisher-z transformed correlation) and self-rated stuttering severity.

After observing the decoupled auditory and motor networks in individuals who stutter during the compensation task, we asked whether decreased functional connectivity between these networks was related to self-rated stuttering severity (see “Materials and Methods” section). In AWS, auditory and motor network functional connectivity during the task did not relate to stuttering severity (*r*^2^ = 0.03, *p* = 0.588). During resting state, AWS did not differ from AC in their auditory-motor connectivity (*t*_(26)_ = 0.24, *p* = 0.816), but there was a trend for a relationship to stuttering severity during resting state (Figure 7; *r*^2^ = −0.26, *p* = 0.074).

## Discussion

### Eliciting Compensation Behavior in the Scanner

In previous work, we showed that individuals who stutter compensate for altered pitch feedback, but do so in a less reliable way; the number of compensatory responses and the timing of those responses were both affected, reflecting increased variability in the stuttering group (Sares et al., 2018). In the current study, we successfully elicited compensation behavior from both groups in the MR environment. We did not fully replicate the differences between groups, but the data trended in the same direction. There are likely factors in the MR environment that affect the degree of response to altered feedback, such as external noise from the helium pump and the use of earbuds rather than on-ear headphones.

While performing the task in the scanner, participants on average recruited the superior temporal gyrus to process shifted pitch relative to unshifted pitch, replicating previous MRI studies on pitch compensation (Toyomura et al., 2007; Zarate and Zatorre, 2008; Zarate et al., 2010; Parkinson et al., 2012; Behroozmand et al., 2015). No motor areas appeared at a corrected threshold in this contrast; this is not surprising as other studies of altered feedback have also failed to find motor activity in their main contrast of shifted vs. unshifted trials (Parkinson et al., 2012; Behroozmand et al., 2015). It could be that more power is needed to detect the motor changes involved in compensating for a small pitch shift (whereas the auditory areas respond much more robustly), or that motor changes are taking place subcortically in brainstem vocal motor areas.

### Decreased Activity in Middle Temporal Gyrus and Frontal BA 10

In the GLM analysis (Figure 3), when compared to controls, individuals who stutter respond to a pitch shift with less activation in both right middle temporal gyrus (MTG) and BA 10. This was supported by an interaction between group and condition in the fronto-temporoparietal networks in the ICA analysis.

The MTG has been implicated in monitoring sensory feedback based on pitch-shifting and delayed feedback studies (McGuire et al., 1996; Hashimoto and Sakai, 2003). When reading aloud under large shifts in pitch, non-stuttering participants increased activation in lateral temporal cortex, with a greater response on the right side compared to the left (McGuire et al., 1996). Similarly, under delayed auditory feedback during oral reading, non-stuttering participants increased activation in right superior temporal gyrus extending into MTG (Hashimoto and Sakai, 2003). A recent study using active and passive movements of the hand showed that MTG, in conjunction with the cerebellum, was involved in the processing of temporal discrepancies in active feedback monitoring (van Kemenade et al., 2019). The MTG may process information about sensory feedback, especially in terms of timing, for sensorimotor integration. Our previous behavioral results demonstrating increased temporal variability (Sares et al., 2018), and a reduced neural response to pitch shifts in the present study suggest that the MTG may contribute to atypical compensatory responses seen in individuals who stutter.

Anatomical structure and functional connectivity also support a link between MTG and sensorimotor integration deficits observed in individuals who stutter. They have less gray matter in MTG (Lu et al., 2010), while people highly trained in sensorimotor integration, like musicians and dancers, show increased gray matter (Bermudez et al., 2009; Cross et al., 2009; Karpati et al., 2017). Children who stutter have weaker functional connectivity between the MTG region and other brain regions at rest, and this is related to stuttering persistence (Chang et al., 2018). This last article proposed the MTG as a hub of the dorsal attentional network, which has hypoconnectivity in stuttering, especially to the default mode network. Thus, relative deactivation of the MTG may be a result of more broad atypical neural organization.

The functional role of the frontopolar cortex (BA 10) is poorly understood, but is known to be involved with task monitoring and switching behaviors (Koechlin and Hyafil, 2007). This area has been found to be densely interconnected with the auditory association area in monkeys (Medalla and Barbas, 2014). In humans, the frontopolar cortex is functionally connected with the MTG (Yeo et al., 2011), consistent with the ICA results in the present study (all subjects; ICs 22 and 26). The coincidental activity of BA 10 and the MTG during the shifted trials implies that BA 10 may receive information about auditory feedback from the MTG and engage a compensatory response for pitch shifts. Fluent participants showed a relative increase in activation in these two coupled areas in response to the shift while individuals who stutter did not. In fact, individuals who stutter showed a relative deactivation of these regions on average (see Figure 4).

It is worth mentioning that the differences seen in the contrast of AC > AS for shifted > unshifted condition are relative. There were not many baseline trials (i.e., no-vocalization trials) in this experiment, so there are not separate contrasts for shifted > baseline and unshifted > baseline to ground this difference. In fact, the ICA results from the fronto-temporoparietal networks (Figure 5; ICs 22 and 26) suggest that these areas may undergo *deactivation* during vocalization in general, with fluent speakers showing *less deactivation* during altered feedback conditions, while individuals who stutter show *more deactivation*.

### Dissociation of Auditory and Motor Components

For all participants combined (Figure 5), we found that the two components most correlated with the task were sensory and motor in nature, with one being located in bilateral auditory areas (IC 15) and the other in bilateral motor areas (IC 8) that included pitch-controlling laryngeal muscles (Dichter et al., 2018). Auditory network activity varied significantly between the shifted and unshifted trials, being more positively correlated with the shifted trials in both groups. Additionally, AWS displayed higher activity in the auditory network (although this statistic did not meet the FDR correction threshold).

ICA results from the separate groups (Figure 6) demonstrate that the auditory and motor components were dissociated (or at least not as tightly associated) in AWS during vocalization (IC 23 for AC, ICs 18 and 13 for AWS). This was in contrast to the resting state data, where the auditory and motor areas were separable for *both* groups (though even here there is a trend towards less connectivity between the two networks with greater stuttering severity).

This apparent neural decoupling fits well with our previous finding of more variable compensation to pitch shifts for individuals who stutter (Sares et al., 2018). Unreliable or inconsistent communication between the auditory and motor networks may explain the more variable behavior we observed previously. A related finding under altered auditory feedback has been demonstrated independently using EEG (Sengupta et al., 2016, 2017), which also suggested a network-level discoordination (or aberrant communication) in stuttering.

### Other Network Differences in Individuals Who Stutter Compared to Fluent Speakers

Both groups had primary auditory and motor cortices in their top networks related to the task. However, in the ICA with all participants, the auditory and motor networks of individuals who stuttered were *more* correlated with every task condition (shifted up, shifted down, and even unshifted; Figure 5). Over-activity in the motor network is consistent with a meta-analytical examination of the speech of individuals who stutter (Brown et al., 2005). However, the same meta-analysis found that *auditory* activation tends to be reduced. In the current study, we observed that individuals who stutter had *increased* activity, much like in the motor network. This may be because our task-based “auditory” component covered some motor regions as well (see Figure 5). As an aside, it is interesting that vocalization alone results in this motor over-activity, even in the absence of spoken words.

Another network involved with the task in AC but not AWS was a somewhat right-lateralized inferior and middle frontal network (Figure 6, IC 28). The inferior frontal cortex was also part of the right and left fronto-temporoparietal networks in the ICA of both groups together, which showed AC responding more positively to a shifted condition and AWS responding more negatively to a shifted condition. The inferior frontal gyrus has been a focus of stuttering research, with individuals who stutter having abnormal activity (Neumann et al., 2003; Kell et al., 2009; Walsh et al., 2017), gray matter (Chang et al., 2008; Beal et al., 2013), and white matter connectivity (Jäncke et al., 2004; Chang et al., 2008; Watkins et al., 2008; Chang and Zhu, 2013) in this region. Improvement in IFG function has been related to post-therapy amelioration of fluency (Kell et al., 2009), and transcranial direct current stimulation of this area shows promise in reducing stuttering when combined with behavioral therapy (Chesters et al., 2018). The frontal aslant tract, connecting medial frontal motor planning regions to inferior frontal regions, has greater mean diffusivity in individuals who stutter, indicating abnormal connectivity between these regions (Neef et al., 2015; Kronfeld-Duenias et al., 2016), and intraoperative stimulation of this tract can also induce stuttering (Kemerdere et al., 2016). The IFG is also an endpoint for the arcuate fasciculus, which connects the temporal and frontal lobes directly and may be affected in stuttering (Chang et al., 2008). Our ICA results confirm the importance of the IFG, showing that it is more implicated in this pitch compensation task for fluent speakers than those with a stutter.

Though this study has not found strong evidence of the involvement of other regions in this task, we do not preclude areas such as basal ganglia and cerebellum. Indeed, the cerebellum was also present in the aforementioned fronto-temporoparietal networks, and the basal ganglia were a part of the auditory network of both groups together. In addition, the default mode network was among the top five networks most correlated with the task for fluent speakers, but not for AWS.

### Implications for Mechanistic Theories of Stuttering

*Civier and colleagues* (Civier et al., 2010, 2013) have proposed some possible mechanisms for stuttering based on the DIVA model of speech production. These mechanisms include an over-reliance on auditory feedback, abnormal levels of dopamine expression in the basal ganglia, and abnormal corticostriatal white matter tracts. We will discuss some ways our data could fit with these hypotheses; however, we caution the reader that the DIVA models are simplified and that MRI experiments like this one cannot always distinguish between the mechanistic explanations they offer.

The auditory over-reliance hypothesis (Civier et al., 2010) is difficult to justify with our current data. The GLM analysis only showed two areas of difference, MTG, and BA 10. Neither of these areas is explicitly present in the DIVA model. However, the MTG and BA 10 could be part of the “monitoring system” proposed in the model, whose neural correlates were not specified. In the ICA, we did observe slightly greater activity in the auditory network during vocalization for the stuttering group (Figure 5); however, the motor network also showed the same pattern.

The biggest problem for the auditory over-reliance hypothesis is its prediction for the behavioral response to pitch shifts in individuals who stutter. To our understanding, a heavy reliance on auditory feedback and an over-active error detection system predicts a delayed, more robust response to shifted feedback, but we see a less robust response that is not delayed. The response to pitch-shifted feedback was not simulated in the 2010 model, but it would be interesting to see whether the simulations can account for this in some way.

The dopamine and white matter models (Civier et al., 2013) seem to be more consistent with our data. They both result in inconsistent communication in the basal ganglia-thalamocortical loop, which has connections throughout the cortex, and could be a factor in the dissociation we see between the auditory and motor networks (Figure 6). This model might even account for increased local activation in both auditory and motor networks (Figure 5): the brain might be increasing the “gain” in a noisy sensorimotor system in order to deal with the demands of speech. Importantly, these models also fit better with the kind of variable, inconsistent pitch-correction behavior we observed for this task in our previous study.

## Conclusion

Using ICA to examine neural activity during a vocal compensation task, we highlight the dissociation between auditory and motor networks during vocalization in individuals who stutter. The coupling of auditory and motor networks is bidirectional (Rauschecker and Scott, 2009) and direct as well as indirect; during vocalization, the brain engages in both feed-forward and feedback signaling, updating its models based on new information from the environment while also engaging in suppression of self-generated stimuli (Eliades and Wang, 2006; Guenther et al., 2006).

This study suggests that middle temporal, frontopolar, and inferior frontal areas could contribute to an auditory-motor dissociation in the case of stuttering. We found that during shifted feedback relative to normal feedback, the right posterior middle temporal gyrus and frontal BA 10 both deactivate on average in individuals who stutter, while activating in controls. Thus, middle temporal gyrus may not be processing pitch shifts normally in AWS, leading to unreliable compensation for pitch shifts. In addition, the inferior frontal region, while implicated in control participants for this task, was less strongly implicated for the stuttering group. Information passing between auditory and motor cortices can pass by way of the inferior frontal regions, making them potentially important for helping maintain auditory-motor connectivity (Rauschecker and Scott, 2009). Overall, the results align well with previous behavioral and neuroimaging research, demonstrating that the effects of stuttering are not observed in a single brain region, but in diverse brain networks.

## Data Availability Statement

Relevant GIFT output for accepted components is included in the Supplementary Datasheets 1–6. The data supporting the findings of this study (raw MRI, behavioral time course) are available from the corresponding author, upon reasonable request.

## Ethics Statement

This study involving human participants was reviewed and approved by McGill Faculty of Medicine Institutional Review Board. The participants provided their written informed consent to participate in this study.

## Author Contributions

AS designed and coded the experiment, tested participants, analyzed data, and wrote the first draft of the manuscript. MD analyzed behavioral data, contributed to writing the results section, and edited the manuscript. DS assisted with the audio setup and implementation of the experimental procedures and edited the manuscript. HO provided training, scripts, and input related to MRI analysis and statistics; he also edited the manuscript. VG funded the work, gave input on the design and analysis, and edited the manuscript.

## Conflict of Interest

The authors declare that the research was conducted in the absence of any commercial or financial relationships that could be construed as a potential conflict of interest.

## Footnotes

^1^http://ric.uthscsa.edu/mango

^2^http://marsbar.sourceforge.net/

## Abbreviations

AC: adult controls
ANOVA: analysis of variance
AWS: adults with a stutter
BA: Brodmann area
BOLD: blood-oxygen-level-dependent (signal)
GLM: general linear model
(s)ICA: (spatial) independent component analysis
IC: independent component
PCA: principal component analysis
TA: acquisition time
TR: repetition time.
