# Supplementary material for: Neural Correlates of Vocal Pitch Compensation in Individuals Who Stutter
Source: Front Hum Neurosci. 2020 Feb 25;14:18. doi: 10.3389/fnhum.2020.00018 (PMC7053555; doi:10.3389/fnhum.2020.00018)
Supplement: Supplementary file 1 [file Table_1.docx]

**Supplementary material:**

**Neural correlates of vocal pitch compensation in individuals who stutter**

***Abbreviations***

*ACC = anterior cingulate cortex*

*BG = basal ganglia*

*DMN = default mode network*

*FTP = fronto-temporo-parietal network*

*IFG = inferior frontal gyrus*

*MFG = middle frontal gyrus*

*MOG = middle occipital gyrus*

*PCC = posterior cingulate cortex*

*pCG = pre/postcentral gyrus*

*pCu = precuneus*

*SMA = supplementary motor area*

*SPL = superior parietal lobe*

*STG = superior temporal gyrus*

*STS = superior temporal sulcus*

*sup = superior*

**Figure S1: All Participants**

**

*Top: IC 8 and 15 overlapping with their respective motor and auditory networks (the two networks with the highest Tanimoto index), at coordinates [52 -16 16] for IC 8 and [52 2 6] for IC 15.*

*Red = IC of interest*

*Yellow = resting state motor network*

*Dark blue = resting state auditory network*

*Orange = overlap of IC of interest and resting state motor*

*Pink = overlap of IC of interest and resting state auditory*

*Light blue = overlap of resting state auditory and motor networks*

*White = all three networks overlapping*

*Bottom: Tanimoto score for all participants, task-IC versus all resting-state ICs. Rest-IC 4 is a motor component covering pre/postcentral gyrus. Rest-IC 23 is an auditory component covering superior temporal areas and insula. Rest-IC 30 covers the superior motor strip, supplementary motor area, and basal ganglia. Note the overlap of IC 15 in the basal ganglia in the axial and coronal views.*

| **All participants: Top task-related ICs and corresponding resting-state ICs** | | | | | | | |
| --- | --- | --- | --- | --- | --- | --- | --- |
| **Task IC** | **Task variance explained (R^2^)** | **Overlapping**  **resting-state IC 1** | | **Overlapping**  **resting-state IC 2** | | **Overlapping**  **resting-state IC 3** | |
|  |  | **name** | **(Tanimoto)** | **name** | **(Tanimoto)** | **name** | **(Tanimoto)** |
| 8: pCG | 0.149 | 4: pCG | (0.526) | 23: STG | (0.128) | 30: SMA/ sup pCG/ BG | (0.117) |
| 15: STG | 0.125 | 23: STG | (0.477) | 30: SMA/ sup pCG/ BG | (0.149) | 21: STS/IFG | (0.136) |
| 20: pCu/MOG | 0.082 | 7: MOG, SPL, pCu | (0.344) | 20: pCu, DMN | (0.216) | 3: DMN | (0.127) |
| 10: SMA/sup pCG | 0.066 | 5: SMA + sup pCG | (0.643) | 30: SMA/ sup pCG/ BG | (0.241) | 8: pCG | (0.191) |
| 26: right FTP | 0.063 | 10: MFG/IFG | (0.441) | 19: right FTP | (0.135) | 21: STS/IFG | (0.111) |

*Table S1. All participants: task-rest comparison. Rows: Top five networks in order of task variance explained. Columns: (1) component from task-based ICA, (2) Task variance explained, (3-5) spatially overlapping resting-state networks, in order of their overlap as measured with the Tanimoto index. Region labels are approximate. Abbreviations: sup = superior; pCG = pre/postcentral gyrus; STG = superior temporal gyrus; SMA = supplementary motor area; BG = basal ganglia; STS = superior temporal sulcus; IFG = inferior frontal gyrus; pCu = precuneus; MOG = middle occipital gyrus; SPL = superior parietal lobe; DMN = default mode network; FTP = fronto-temporo-parietal network; MFG = middle frontal gyrus. Networks had suprathreshold voxels present in both hemispheres (i.e. none were purely unilateral), but dominance is indicated if present.* *No more than three resting networks overlapped significantly with any task network except in the case of IC 20. IC 20 also overlapped with rest-IC 13 (IPL/MTG; index of 0.109) and rest-IC 30 (SMA/preCG/BG; index of 0.108)*

**Figure S2: AC**

******

*Top: Top two networks correlated with the task for control participants and overlapping motor and auditory networks (the networks with the highest Tanimoto index), at coordinates [54 -4 10] for IC 23 and [48 30 12] for IC 28.*

*Red = IC of interest*

*Yellow = resting state motor network*

*Dark blue = resting state auditory network*

*Orange = overlap of IC of interest and resting state motor*

*Pink = overlap of IC of interest and resting state auditory*

*White = all three networks overlapping*

*Bottom: Tanimoto score for all participants, task-IC versus all resting-state ICs. Rest-IC 2 is a motor component covering pre/postcentral gyrus. Rest-IC 11 is an auditory component covering superior temporal areas and insula. Rest-IC 13 covers bilateral inferior frontal gyrus. Note the highly similar overlap scores between auditory and motor networks for component 23 (left).*

| **Control participants: Top task-related ICs and corresponding resting-state ICs** | | | | | | | |
| --- | --- | --- | --- | --- | --- | --- | --- |
| **Task IC** | **Task variance explained (R^2^)** | **Overlapping**  **resting-state IC 1** | | **Overlapping**  **resting-state IC 2** | | **Overlapping**  **resting-state IC 3** | |
|  |  | **name** | **(Tanimoto)** | **name** | **(Tanimoto)** | **name** | **(Tanimoto)** |
| 23: STG/pCG | 0.1 | 2: pCG | (0.326) | 11: STG | (0.322) |  |  |
| 28: right IFG/MFG | 0.087 | 13: IFG/MFG | (0.325) |  |  |  |  |
| 29: DMN | 0.071 | 4:DMN | (0.533) | 26: DMN | (0.165) |  |  |
| 17: SMA/sup pCG | 0.066 | 6: SMA/sup pCG | (0.591) |  |  |  |  |
| 20: pCu/MOG | 0.063 | 16: cuneus/MOG | (0.219) | 26: DMN | (0.172) | 23: pCu | (0.151) |

*Table S2. Control participants: task-rest comparison. Rows: Top five networks in order of task variance explained. Columns: (1) component from task-based ICA, (2) Task variance explained, (3-5) spatially overlapping resting-state networks, in order of their overlap as measured with the Tanimoto index. Region labels are approximate. Abbreviations: sup = superior; STG = superior temporal gyrus; pCG = pre/postcentral gyrus; IFG = inferior frontal gyrus; MFG = middle frontal gyrus; DMN = default mode network; SMA = supplementary motor area; MOG = middle occipital gyrus; pCu = precuneus. Networks had suprathreshold voxels present in both hemispheres (i.e. none were purely unilateral), but dominance is indicated if present.* *No more than three resting networks overlapped significantly with any task network.*

**Figure S3: AWS**

**

*AS_w_overlaps: Top two networks correlated with the task for stuttering participants and overlapping motor and auditory networks (the networks with the highest Tanimoto index), at coordinates [58 -24 12] for IC 18 and [60 -8 38] for IC 13.*

*Red = IC of interest*

*Yellow = resting state motor network*

*Dark blue = resting state auditory network*

*Orange = overlap of IC of interest and resting state motor*

*Pink = overlap of IC of interest and resting state auditory*

*White = all three networks overlapping*

*Bottom: Tanimoto score for all participants, task-IC versus all resting-state ICs. Rest-IC 17 is an auditory component covering superior temporal areas and insula. The second-highest overlap for component 18 was for a rejected IC. Rest-IC 9 is a motor component covering pre/postcentral gyrus.*

| **Stuttering participants: Top task-related ICs and corresponding resting-state ICs** | | | | | |
| --- | --- | --- | --- | --- | --- |
| **Task IC** | **Task variance explained (R^2^)** | **Overlapping**  **resting-state IC 1** | | **Overlapping**  **resting-state IC 2** | |
|  |  | **name** | **(Tanimoto)** | **name** | **(Tanimoto)** |
| 18: STG | 0.216 | 17: STG | (0.524) |  |  |
| 13: pCG | 0.159 | 9: pCG | (0.6) |  |  |
| 15: SPL/MOG/pCu | 0.112 | 16: cuneus/MOG | (0.506) |  |  |
| 12: ACC | 0.069 | 24: ACC | (0.157) |  |  |
| 2: medial visual | 0.069 | 3: SMA/sup pCG | (0.696) | 6: PCC | (0.098) |

*Table S3. Stuttering participants: task-rest comparison. Rows: Top five networks in order of task variance explained. Columns: (1) component from task-based ICA, (2) Task variance explained, (3-5) spatially overlapping resting-state networks, in order of their overlap as measured with the Tanimoto index. Region labels are approximate. Abbreviations: sup = superior; STG = superior temporal gyrus; pCG = pre/postcentral gyrus; SPL = superior parietal lobe; MOG = middle occipital gyrus; pCu = precuneus; ACC = anterior cingulate cortex; PCC = posterior cingulate cortex. Networks had suprathreshold voxels present in both hemispheres (i.e. none were purely unilateral), but dominance is indicated if present. No more than two resting networks overlapped significantly with any task network.*

**Figures S4.1 and S7.1: Correlations with SSI Values**

Here we show figures 4 and 7 from the original paper with SSI scores instead of self-rated severity. SSI and self-rated severity were highly correlated (r^2^ = 0.72, p = 0.0003), nonetheless, the curious reader may be interested in seeing SSI correlations.

For figure 4, the correlations are somewhat stronger but not quite statistically significant. For figure 7, the correlation is slightly weaker, but again it does not change the statistical significance. In sum, substituting the SSI scores does not change the strength or interpretation of the results.


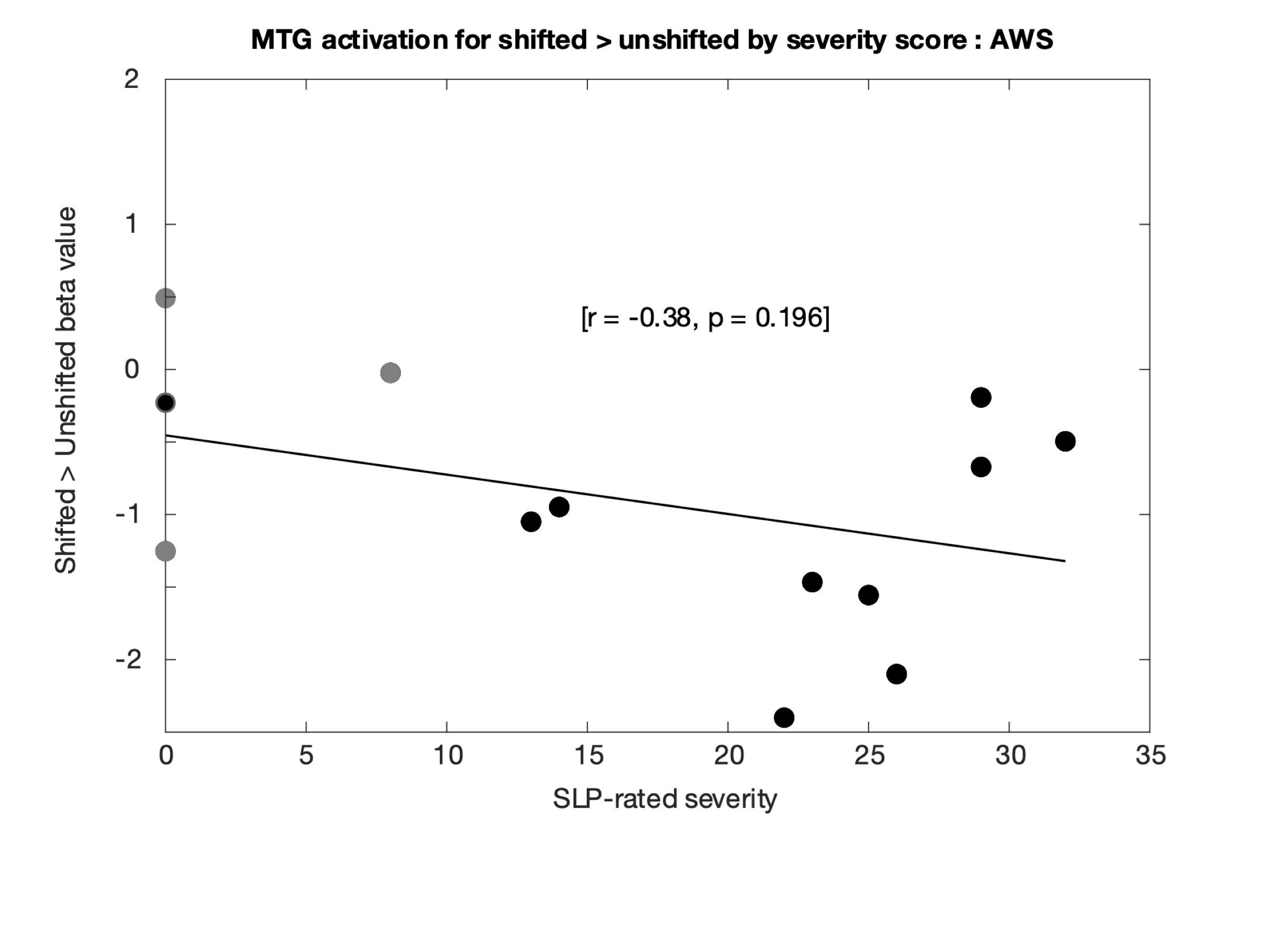

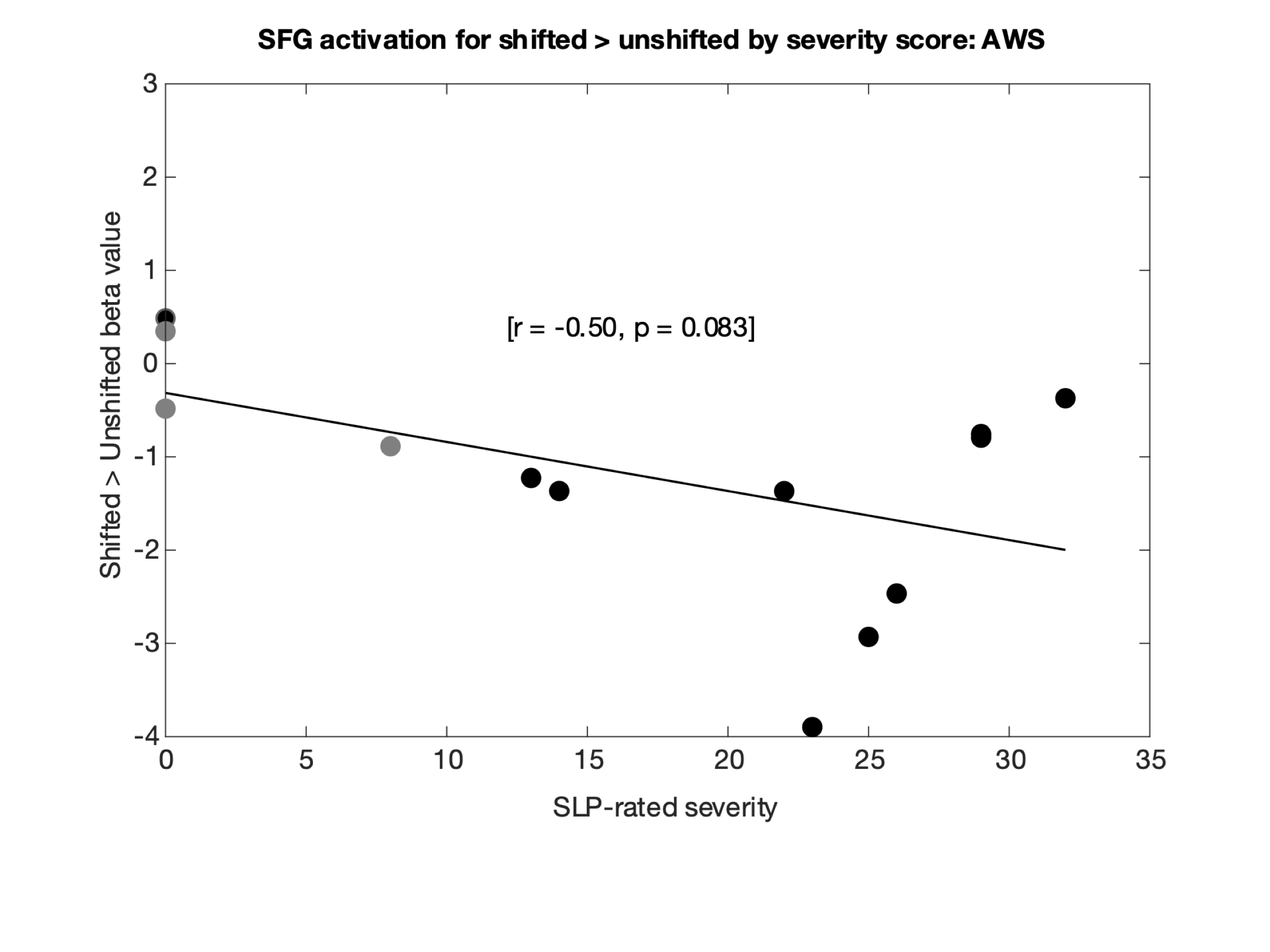


Fig S4.1 (left, MTG; right, SFG)


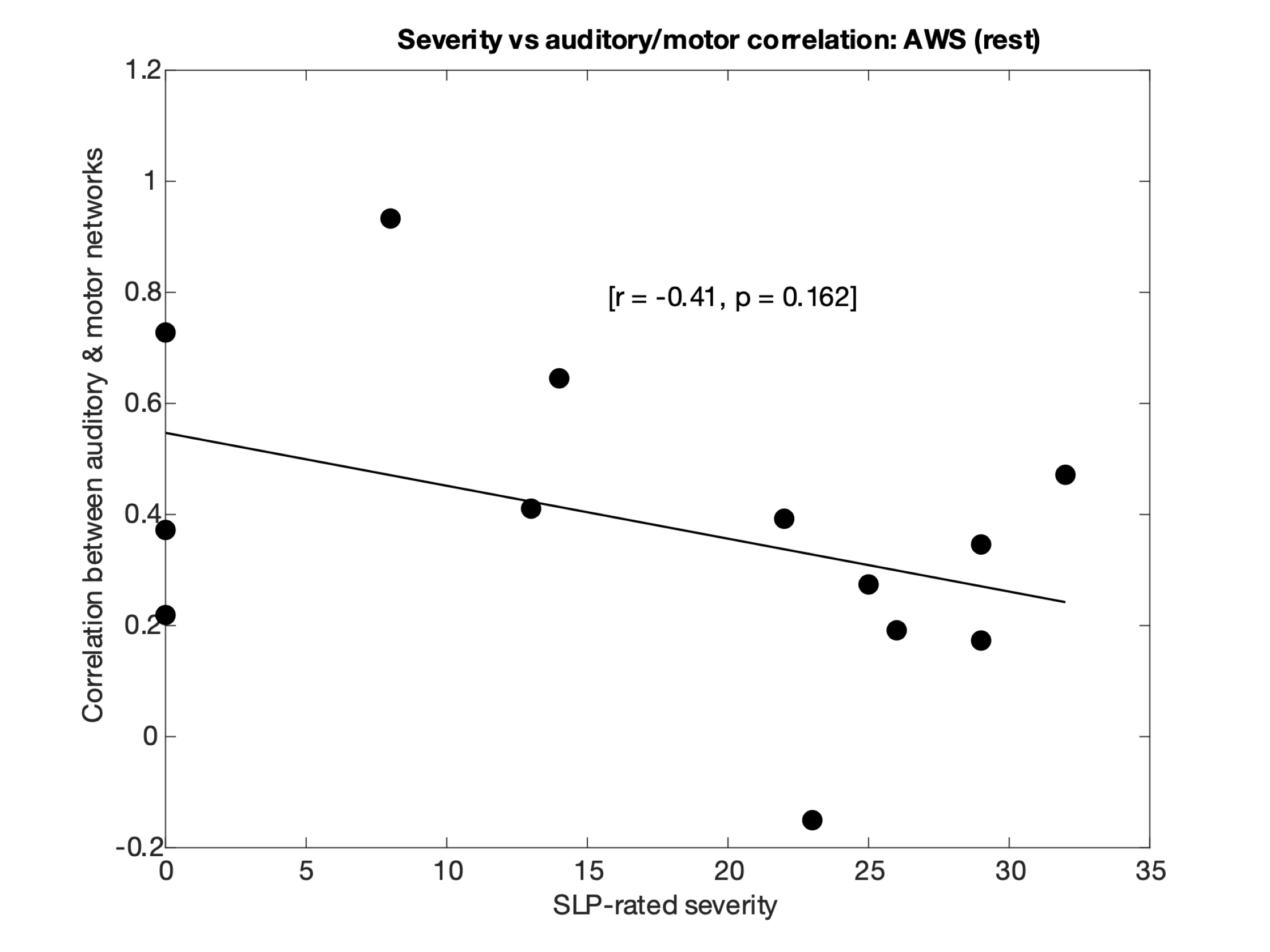


Fig S7.1

**Figures S4.2 and S7.2: Correlations with SSI values above zero only**

Some stuttering participants received an SSI score of zero, and some of the control participants received an SSI score above zero (despite reporting no accompanying diagnosis or history of dysfluency). Below we show figures 4 & 7 with the SSI ratings of individuals who received a score above zero, regardless of their group. Again, this slight variation does not change the interpretation of the results.


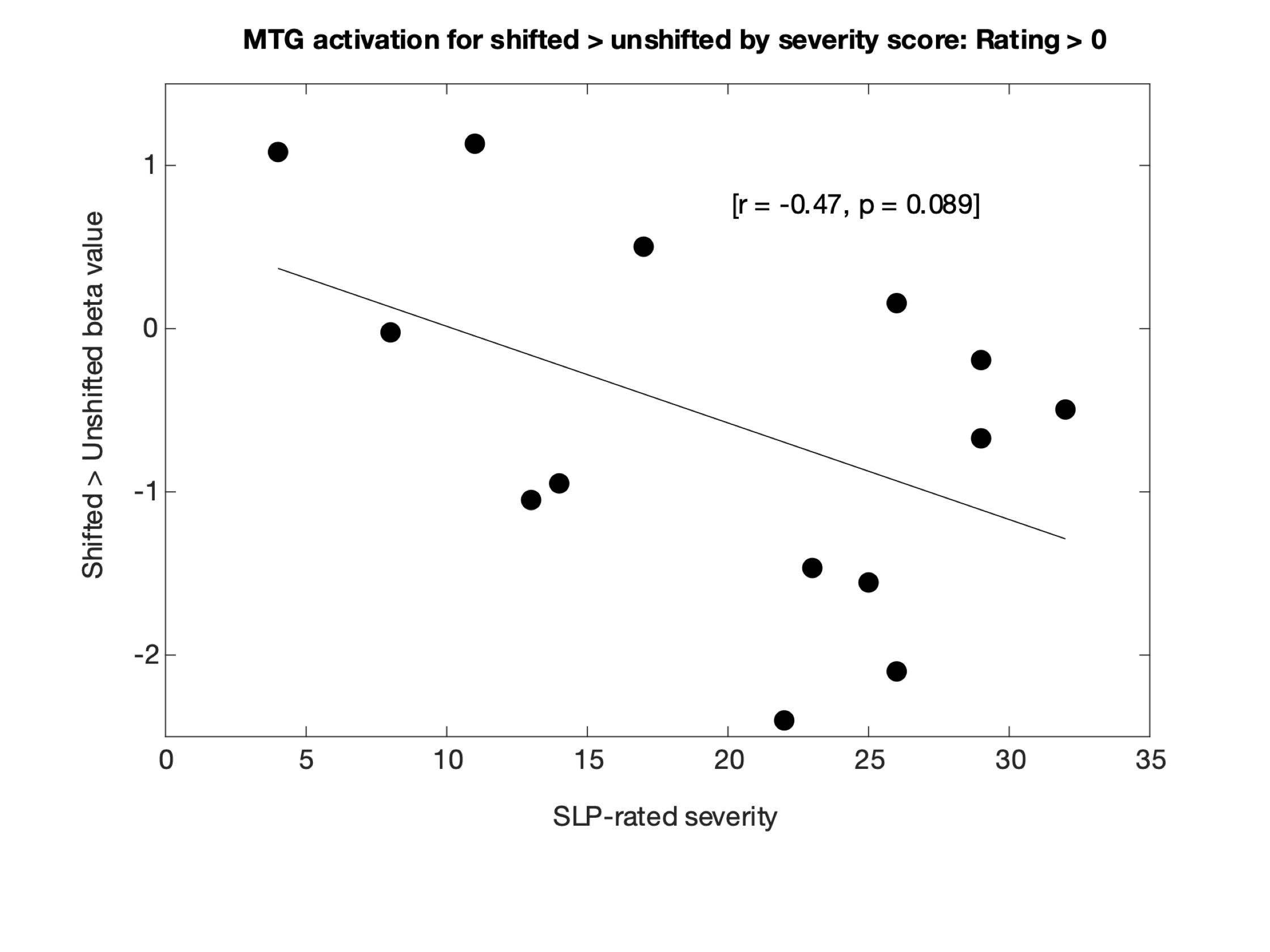

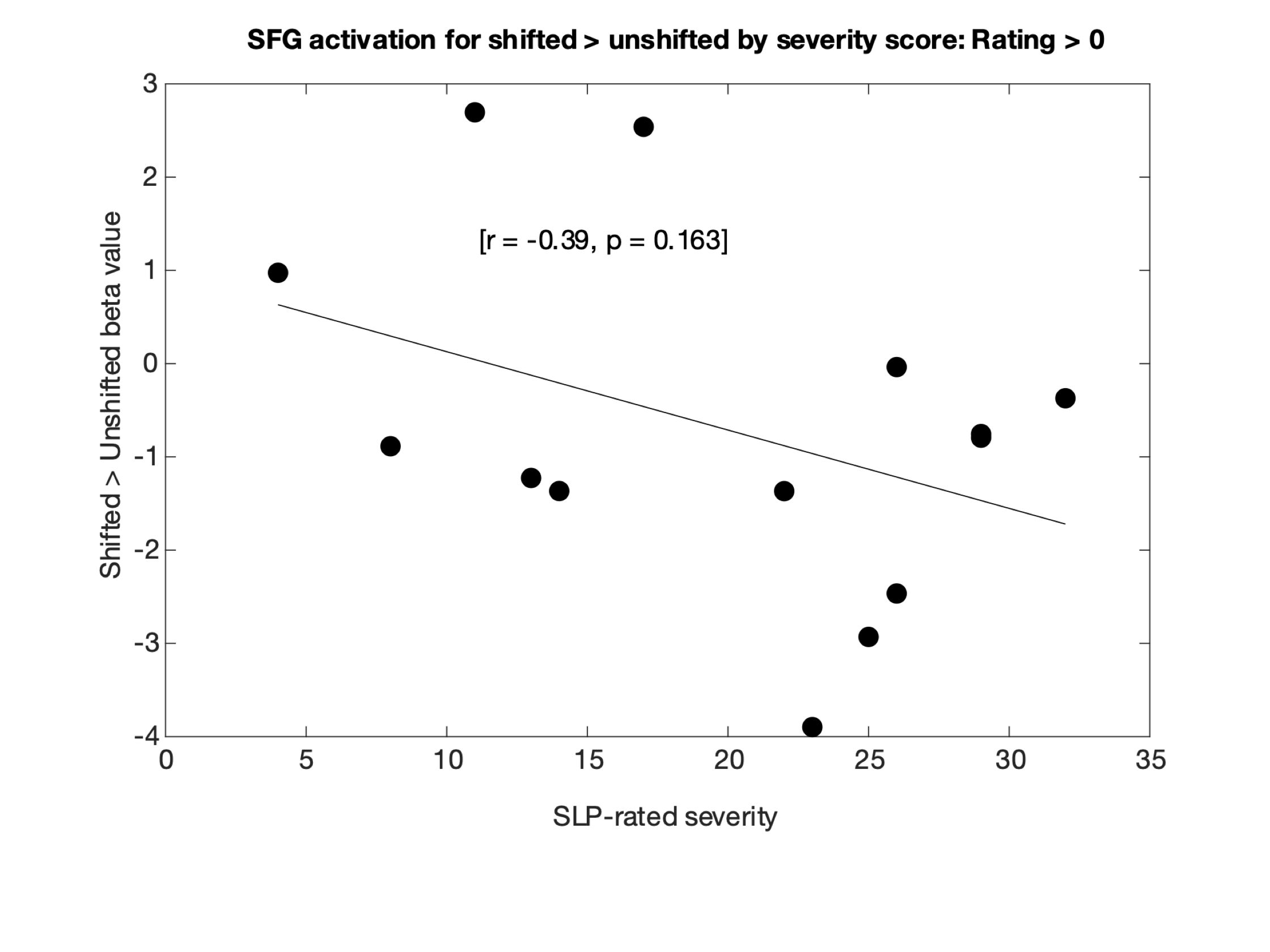


Fig. S4.2 (left, MTG; right, SFG)


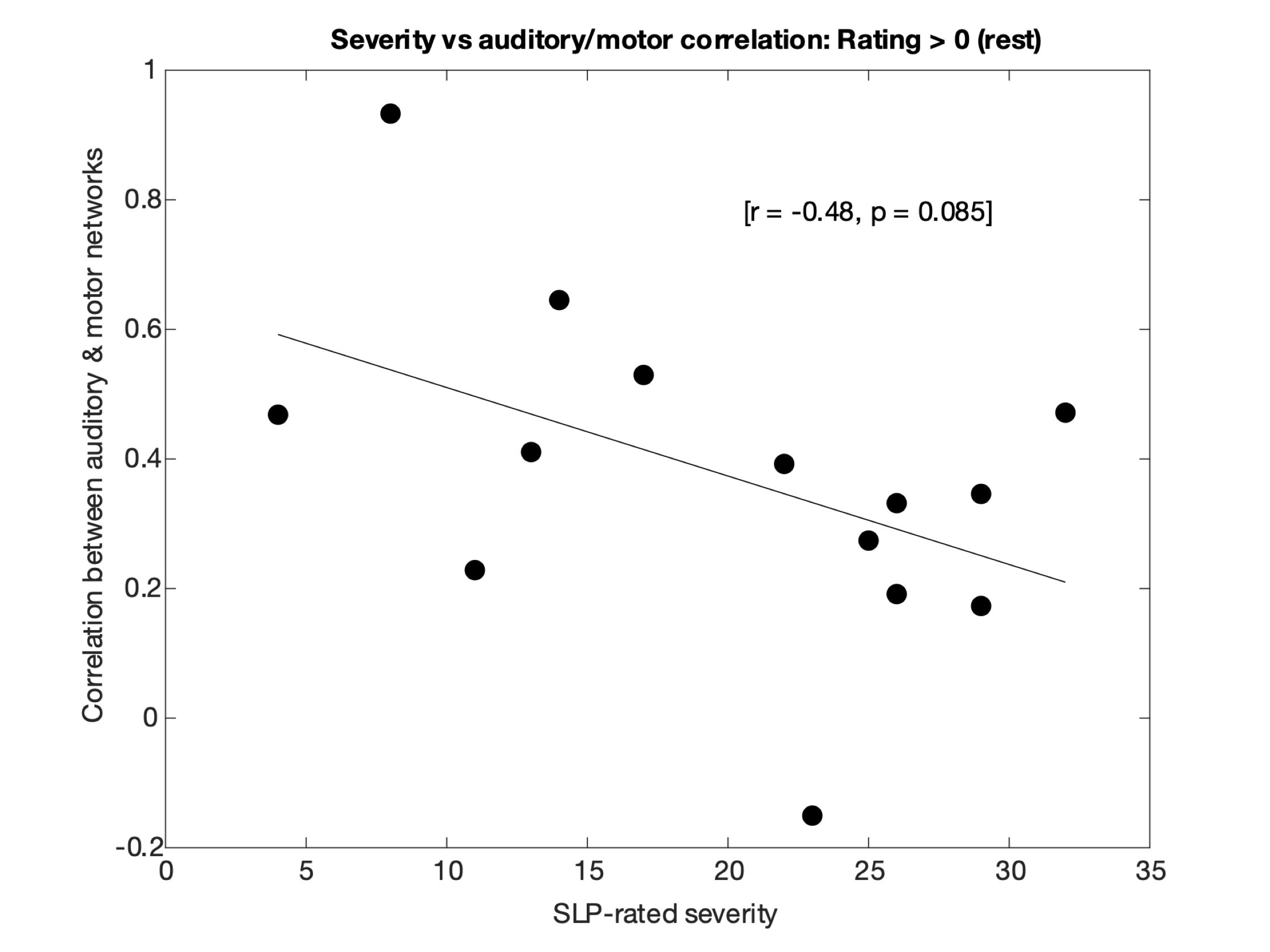


Fig. S7.2
