# Supplementary material for: Neural Correlates of Vocal Pitch Compensation in Individuals Who Stutter
Source: Front Hum Neurosci. 2020 Feb 25;14:18. doi: 10.3389/fnhum.2020.00018 (PMC7053555; doi:10.3389/fnhum.2020.00018)
Supplement: Supplementary file 2 [file Data_Sheet_1.PDF]

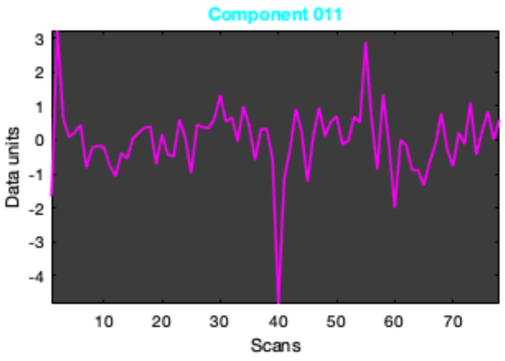
Component 011
Data units
Scans
3
2
1
0
-1
-2
-3
-4
10
20
30
40
50
60
70

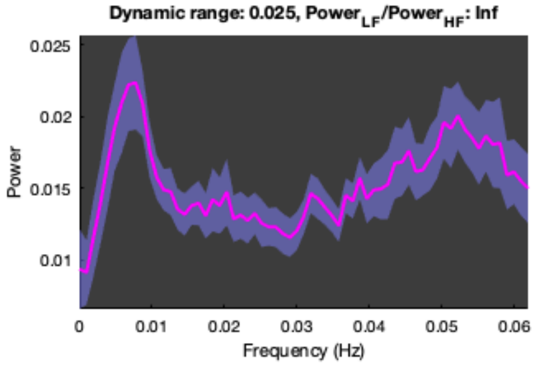
Dynamic range: 0.025, Power$_{LF}$/Power$_{HF}$: Inf
Power
Frequency (Hz)
0.025
0.02
0.015
0.01
0
0.01
0.02
0.03
0.04
0.05
0.06

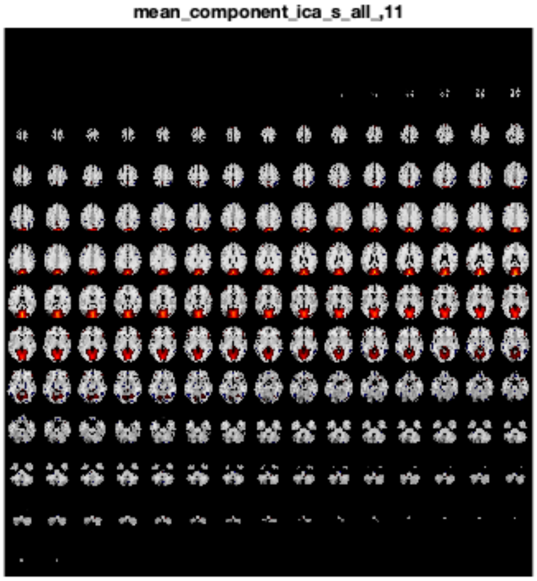
mean_component_ica_s_all_,11

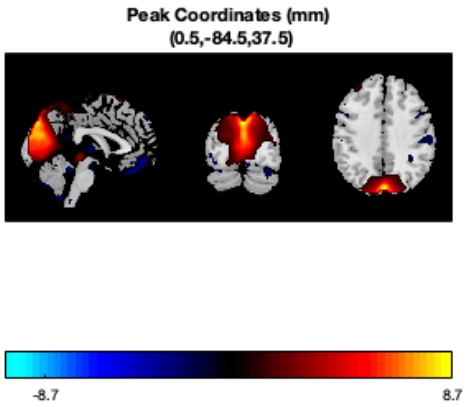
Peak Coordinates (mm)
(0.5,-84.5,37.5)
-8.7
8.7

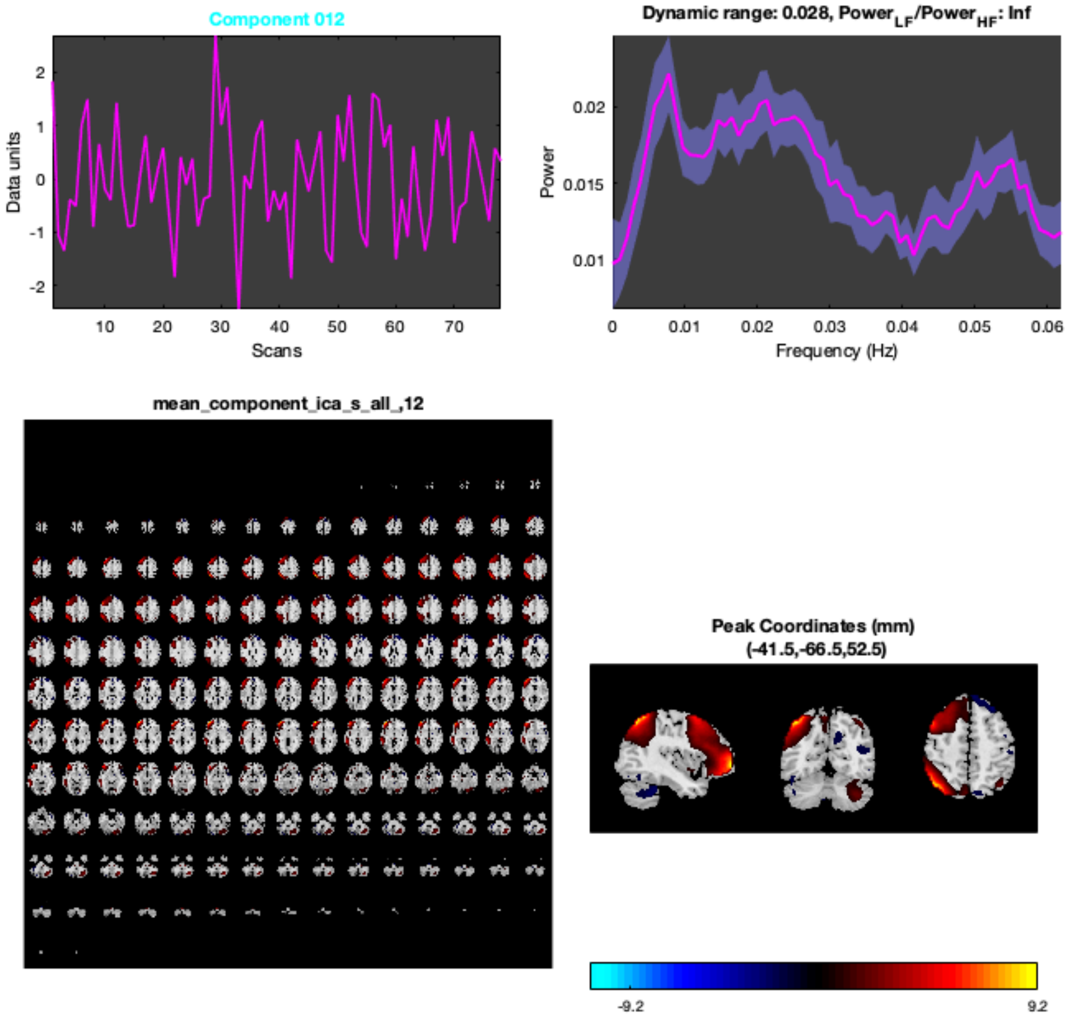
Component 012
Data units
Scans
Dynamic range: 0.028, Power$_{LF}$/Power$_{HF}$: Inf
Power
Frequency (Hz)
mean_component_ica_s_all_,12
Peak Coordinates (mm)
(-41.5,-66.5,52.5)
-9.2
9.2

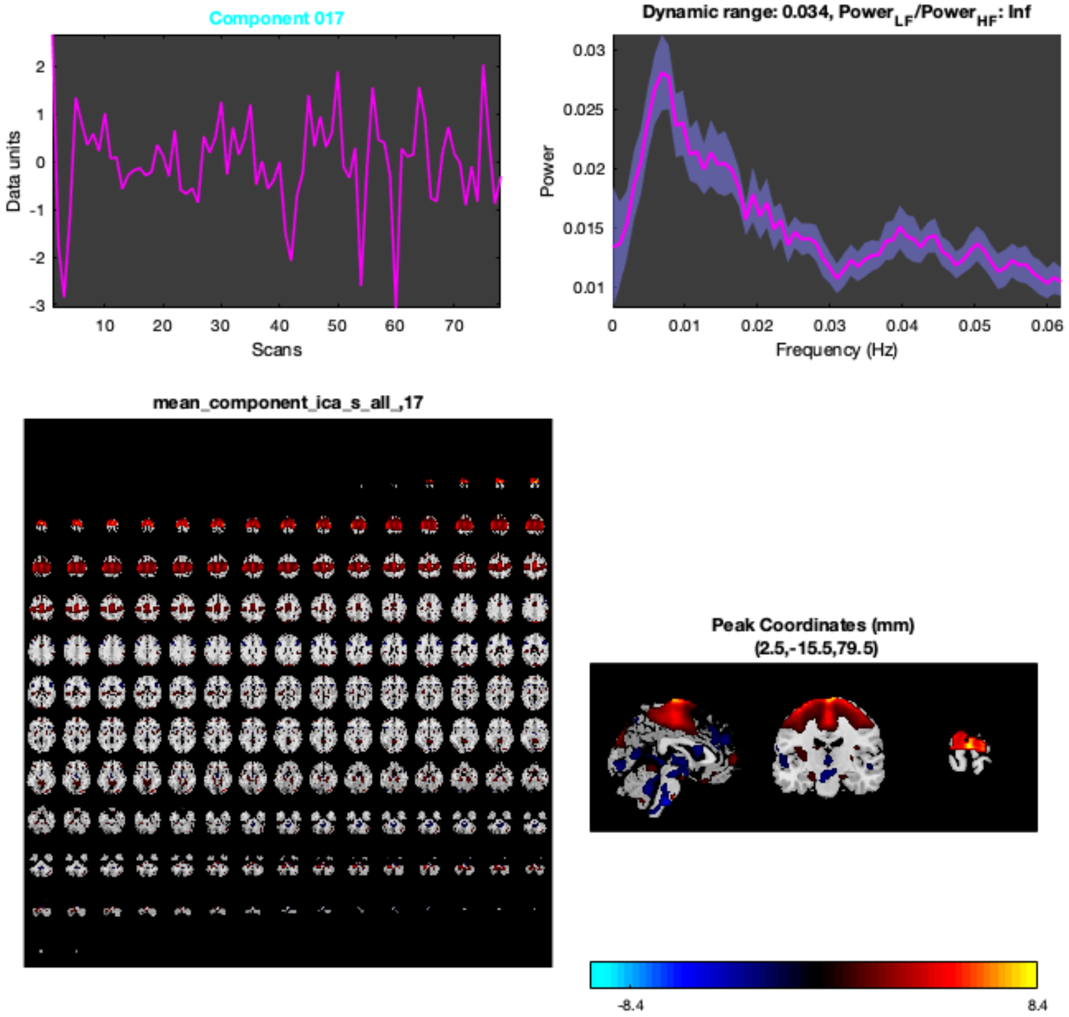
Component 017
Data units
Scans
Dynamic range: 0.034, Power$_{LF}$/Power$_{HF}$: Inf
Power
Frequency (Hz)
mean_component_ica_s_all_,17
Peak Coordinates (mm)
(2.5,-15.5,79.5)
-8.4
8.4

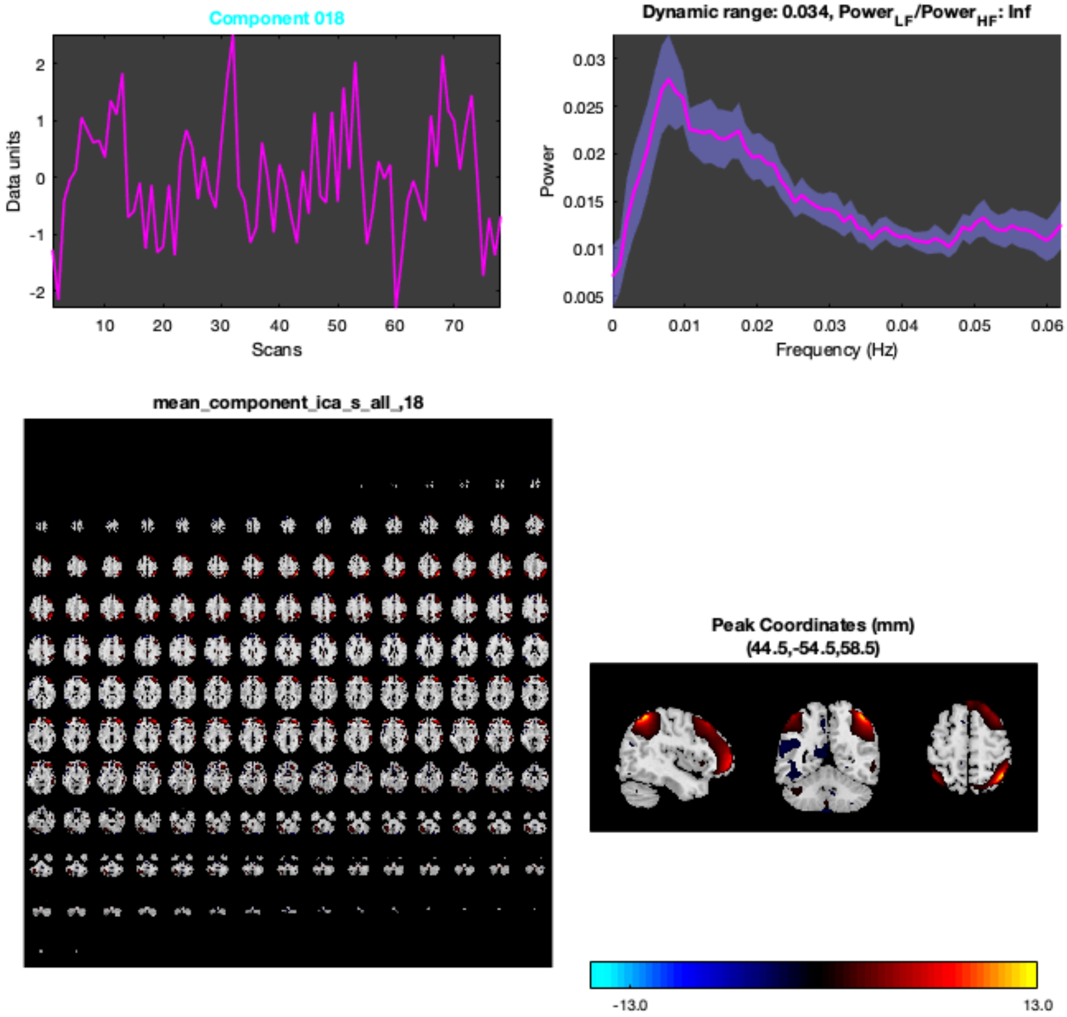
Component 018
Data units
Scans
Dynamic range: 0.034, Power$_{LF}$/Power$_{HF}$: Inf
Power
Frequency (Hz)
mean_component_ica_s_all_,18
Peak Coordinates (mm)
(44.5,-54.5,58.5)
-13.0
13.0

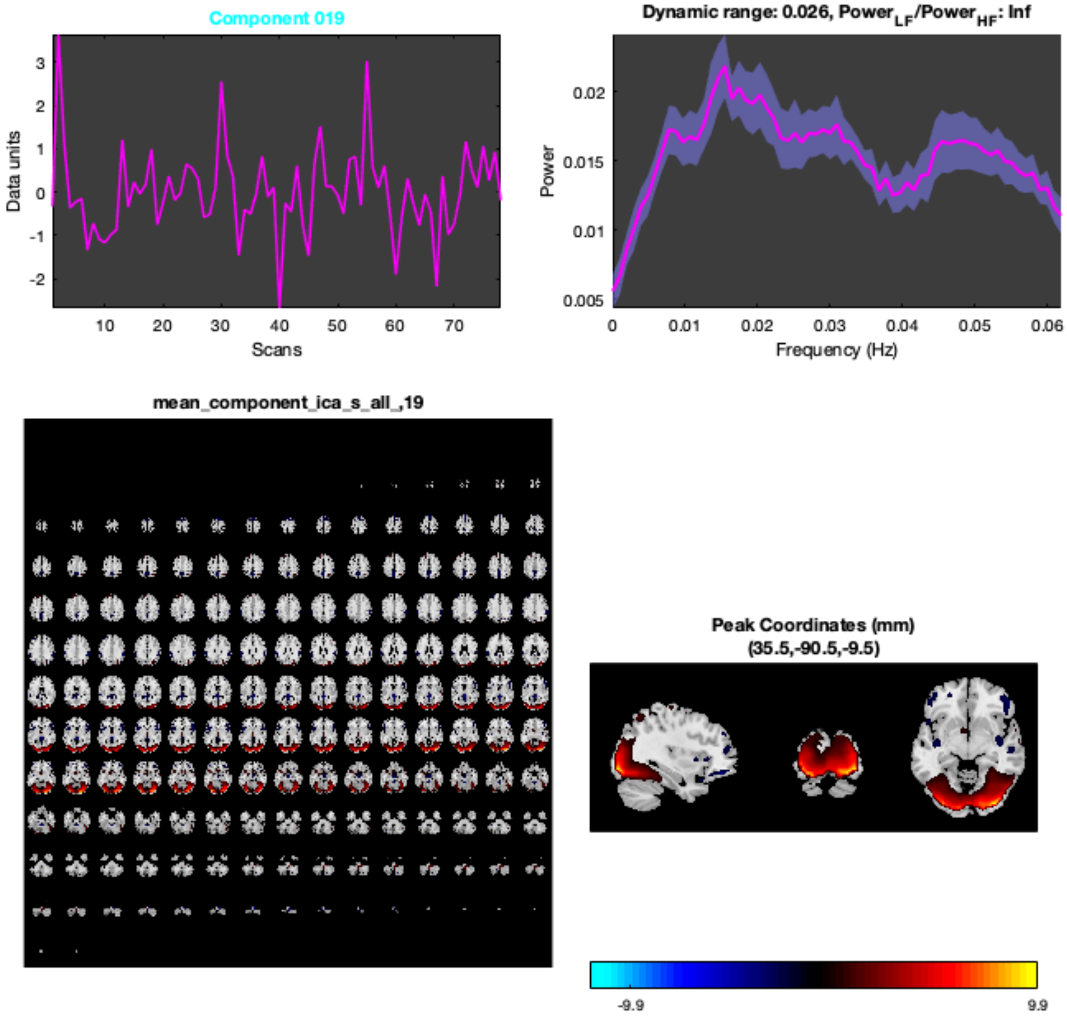
Component 019
Data units
Scans
Dynamic range: 0.026, Power$_{LF}$/Power$_{HF}$: Inf
Power
Frequency (Hz)
mean_component_ica_s_all_,19
Peak Coordinates (mm)
(35.5,-90.5,-9.5)
-9.9
9.9

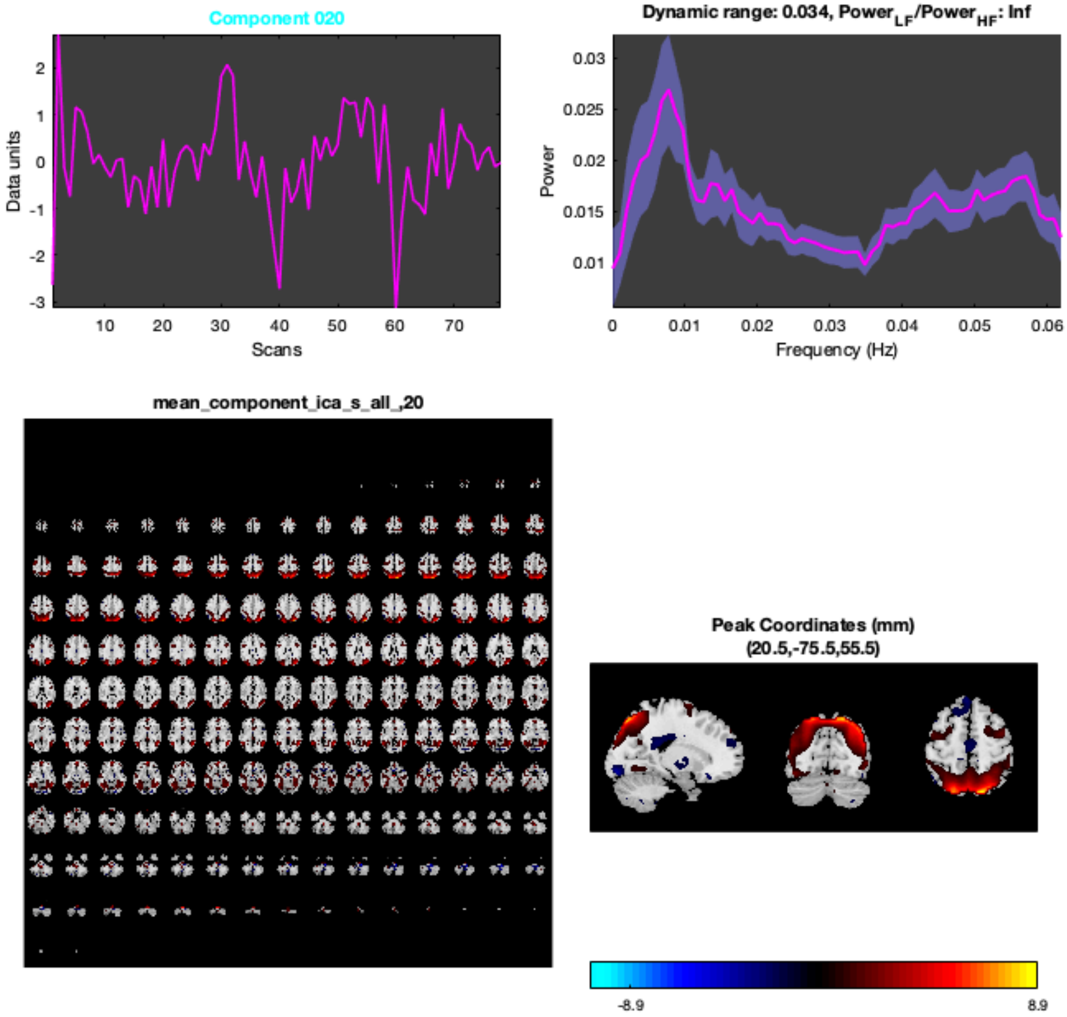
Component 020
Data units
Scans
Dynamic range: 0.034, Power$_{LF}$/Power$_{HF}$: Inf
Power
Frequency (Hz)
mean_component_ica_s_all_,20
Peak Coordinates (mm)
(20.5,-75.5,55.5)
-8.9
8.9

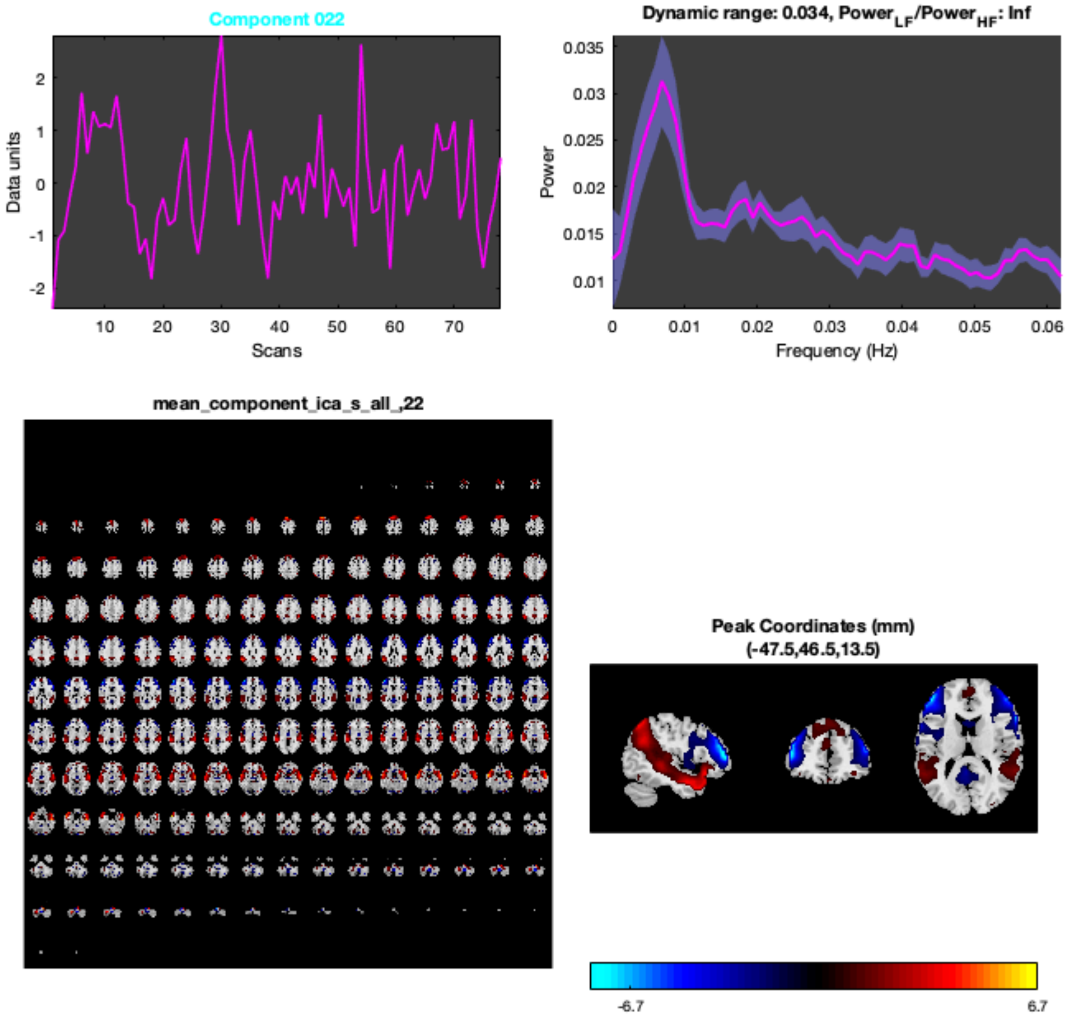
Component 022
Data units
Scans
Dynamic range: 0.034, Power_LF/Power_HF: Inf
Power
Frequency (Hz)
mean_component_ica_s_all_,22
Peak Coordinates (mm)
(-47.5,46.5,13.5)
-6.7
6.7

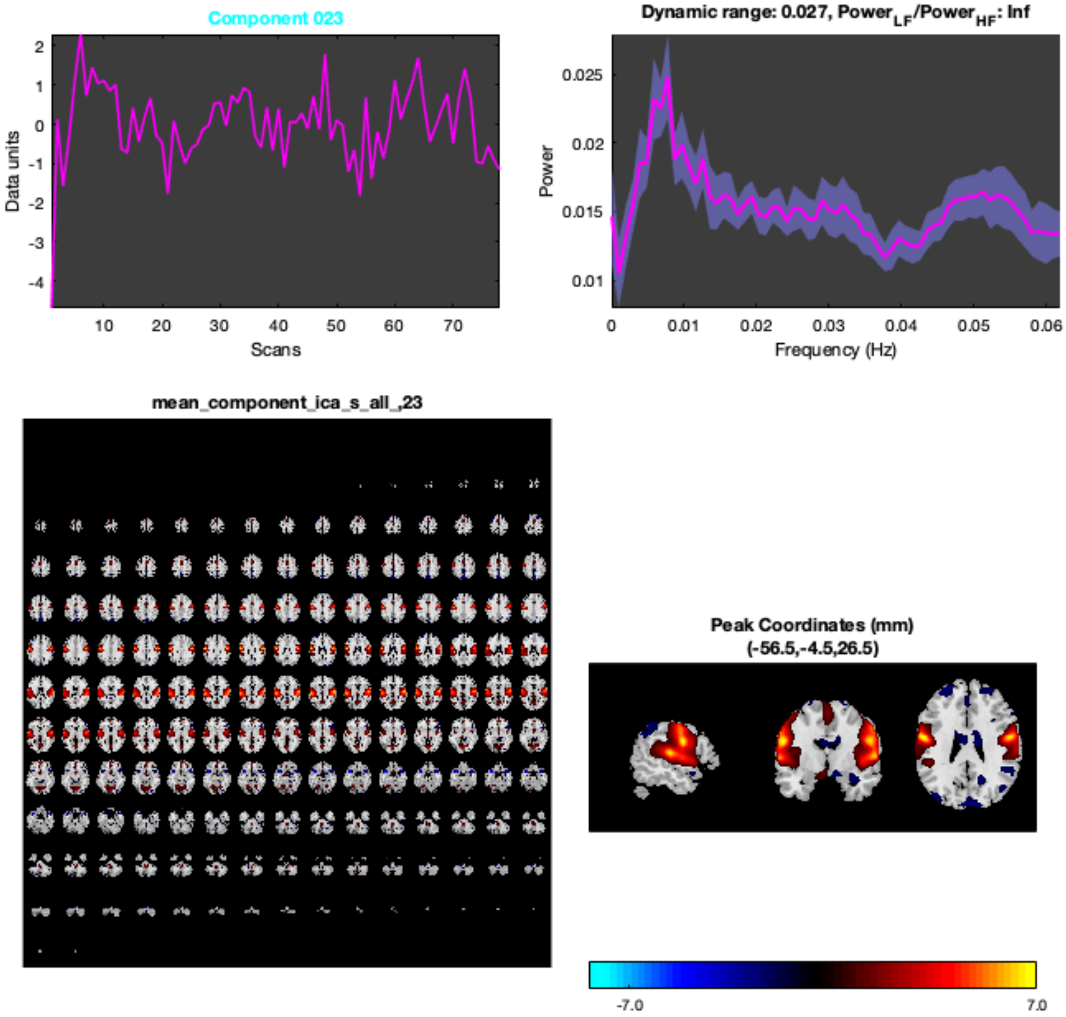
Component 023
Data units
Scans
Dynamic range: 0.027, Power$_{LF}$/Power$_{HF}$: Inf
Power
Frequency (Hz)
mean_component_ica_s_all_,23
Peak Coordinates (mm)
(-56.5,-4.5,26.5)
-7.0
7.0

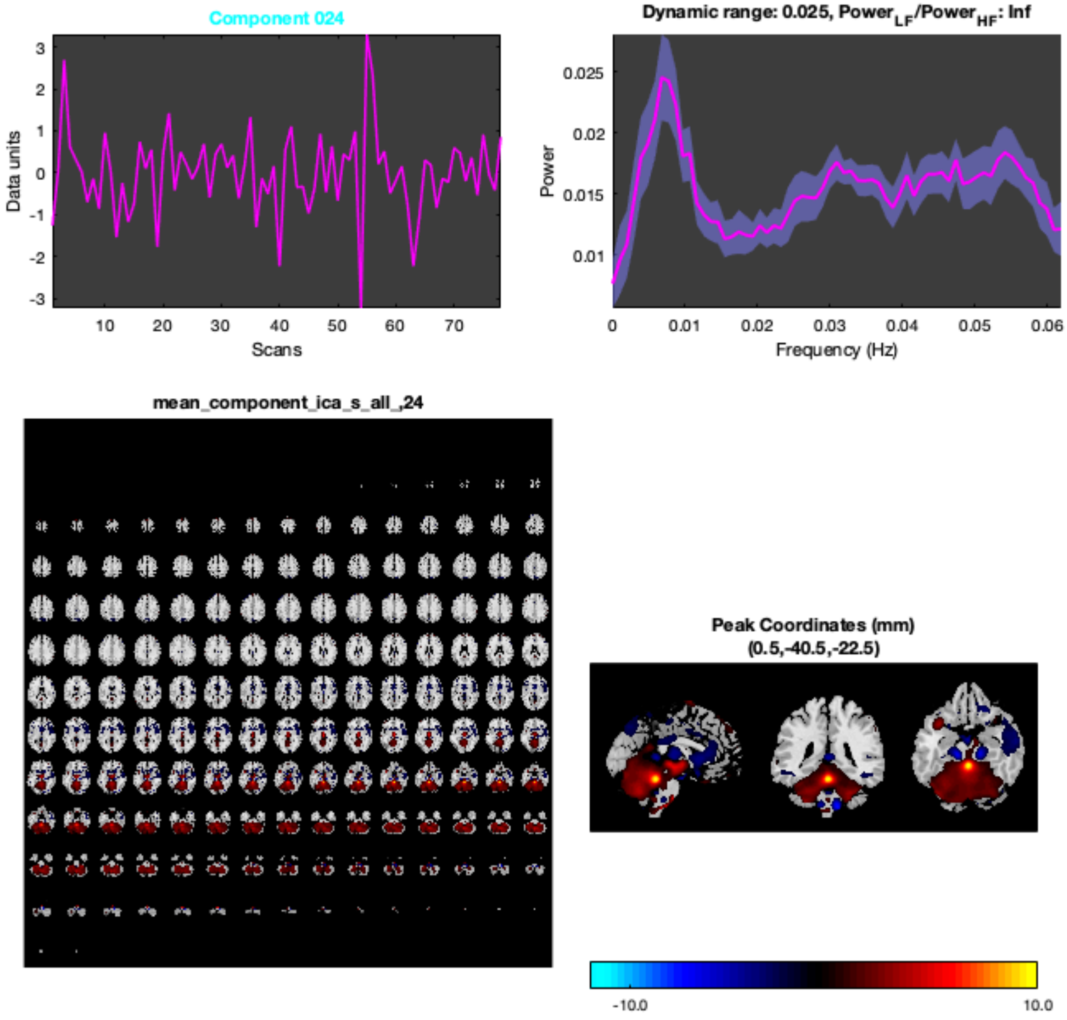
Component 024
Data units
Scans
Dynamic range: 0.025, Power$_{LF}$/Power$_{HF}$: Inf
Power
Frequency (Hz)
mean_component_ica_s_all_,24
Peak Coordinates (mm)
(0.5,-40.5,-22.5)
-10.0
10.0

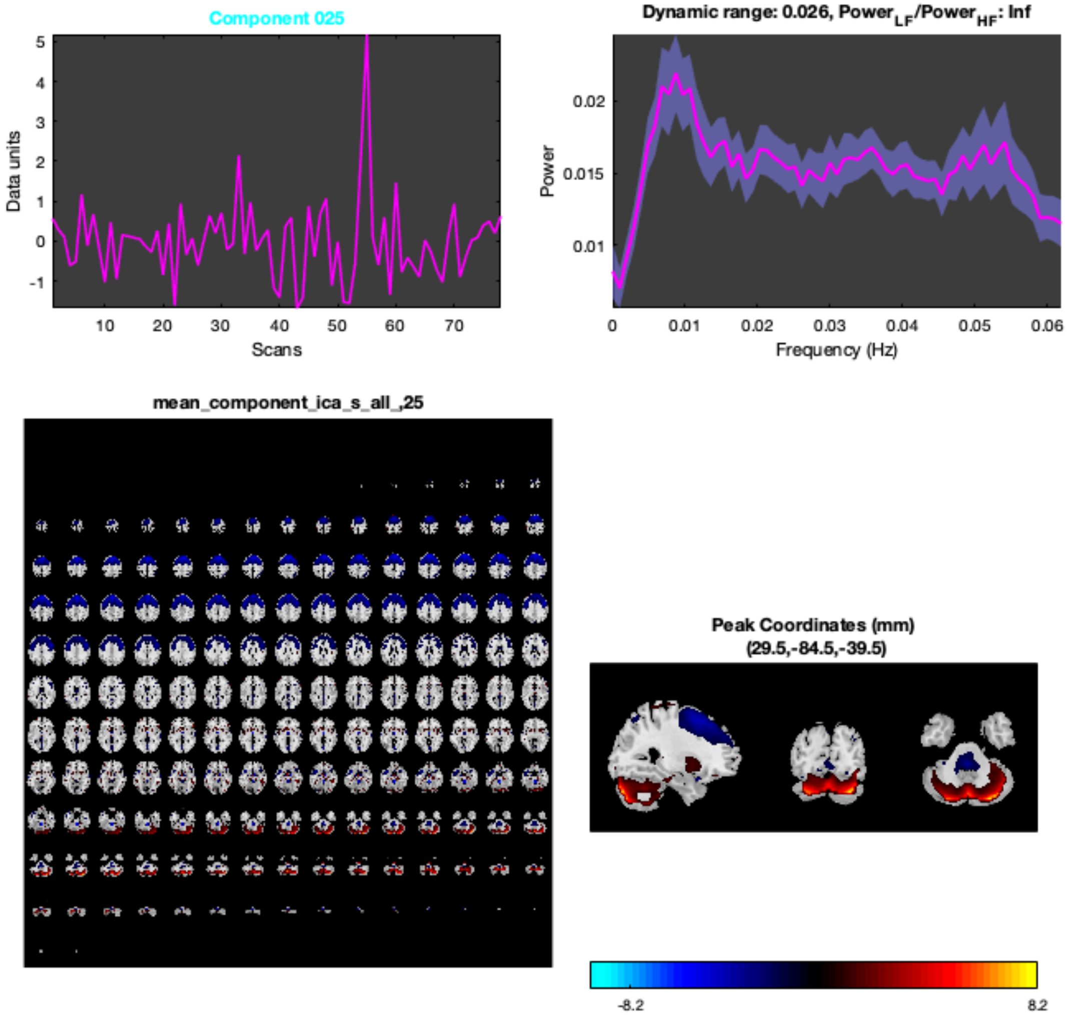
Component 025
Data units
Scans
Dynamic range: 0.026, Power_LF/Power_HF: Inf
Power
Frequency (Hz)
mean_component_ica_s_all_,25
Peak Coordinates (mm)
(29.5,-84.5,-39.5)
-8.2
8.2

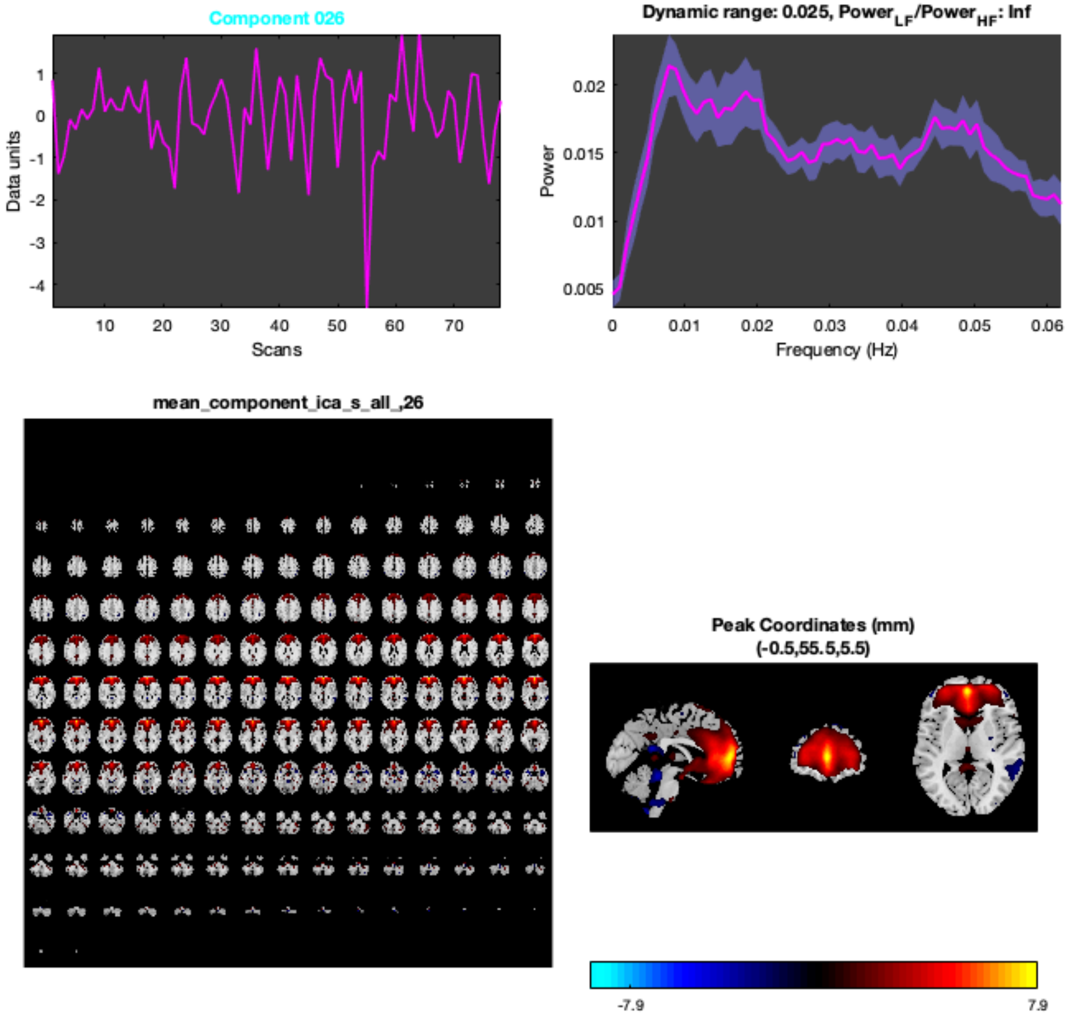
Component 026
Data units
Scans
Dynamic range: 0.025, Power$_{LF}$/Power$_{HF}$: Inf
Power
Frequency (Hz)
mean_component_ica_s_all_,26
Peak Coordinates (mm)
(-0.5,55.5,5.5)
-7.9
7.9

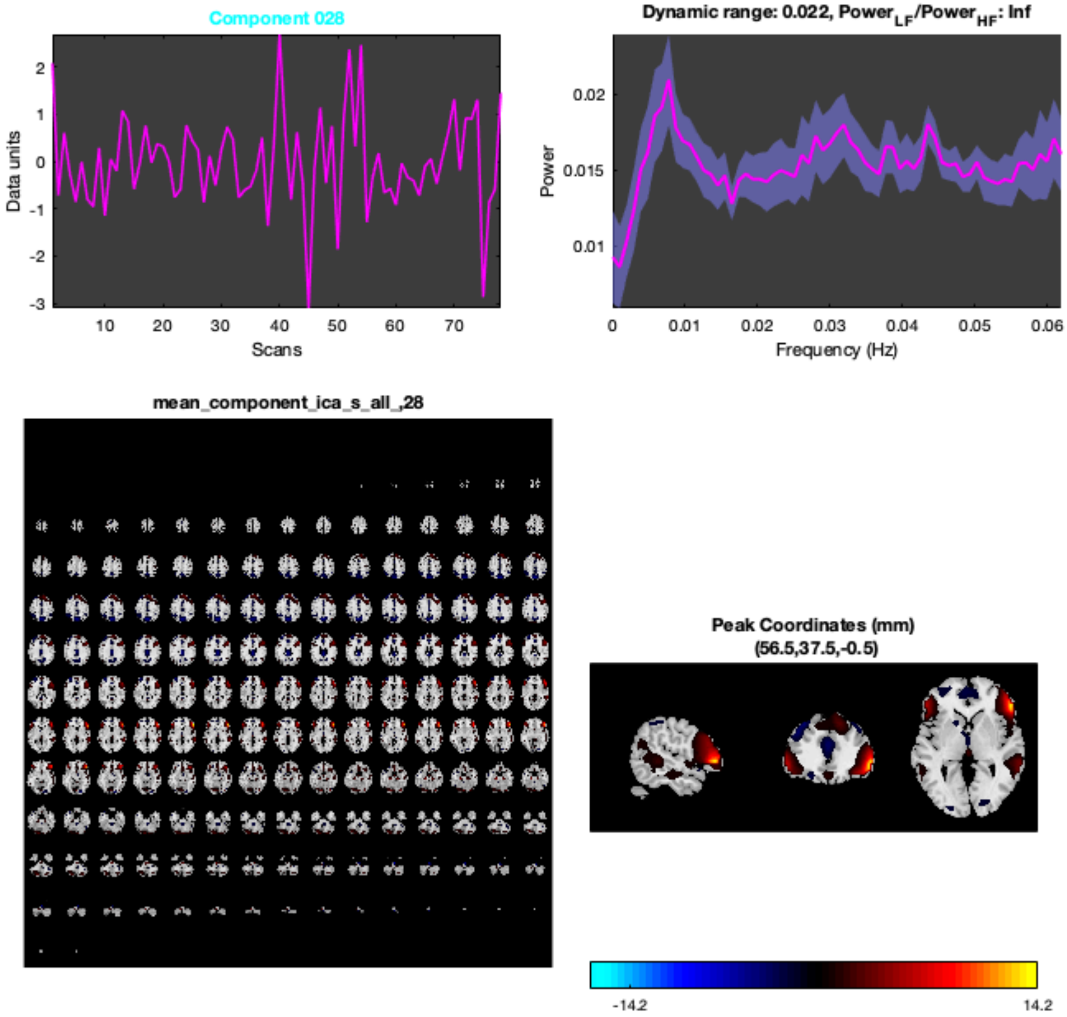
Component 028
Data units
Scans
Dynamic range: 0.022, Power$_{LF}$/Power$_{HF}$: Inf
Power
Frequency (Hz)
mean_component_ica_s_all_,28
Peak Coordinates (mm)
(56.5,37.5,-0.5)
-14.2
14.2

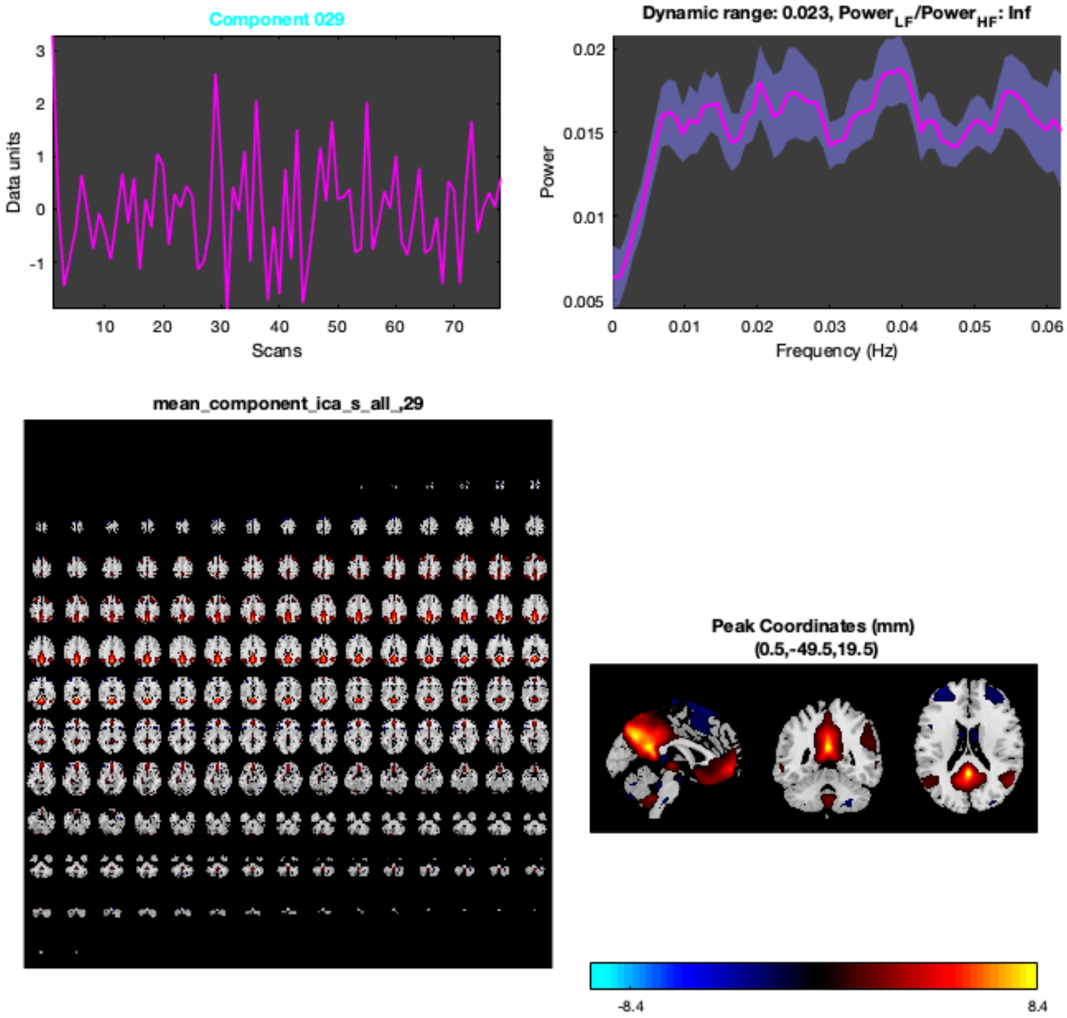

Spectral Summary

- **a) dynamic_range** - Difference between the peak power and minimum power at frequencies to the right of the peak.
- **b) fALFF** - Low frequency to high frequency power ratio.

ComponentNumber	*DynamicRange*	*fALFF*
1	*0.022469*	*Inf*
2	*0.0236*	*Inf*
3	*0.025608*	*Inf*
4	*0.01916*	*Inf*
5	*0.027749*	*Inf*
6	*0.022062*	*Inf*
7	*0.025791*	*Inf*
8	*0.027262*	*Inf*
