# Supplementary material for: Neural Correlates of Vocal Pitch Compensation in Individuals Who Stutter
Source: Front Hum Neurosci. 2020 Feb 25;14:18. doi: 10.3389/fnhum.2020.00018 (PMC7053555; doi:10.3389/fnhum.2020.00018)
Supplement: Supplementary file 3 [file Data_Sheet_2.PDF]

Mean Components

Mean across all subjects and sessions is computed for each component

- **a) Timecourse** - Mean timecourse is converted to z-scores.
- **b) Spectra** - Timecourses spectra is computed for each data-set and averaged across sessions. Mean and standard error of mean is shown in the figure.
- **c) Montage** - Axial slices are shown.
- **d) Ortho slices** - Ortho plot is shown for the peak voxel and coordinates are reported.

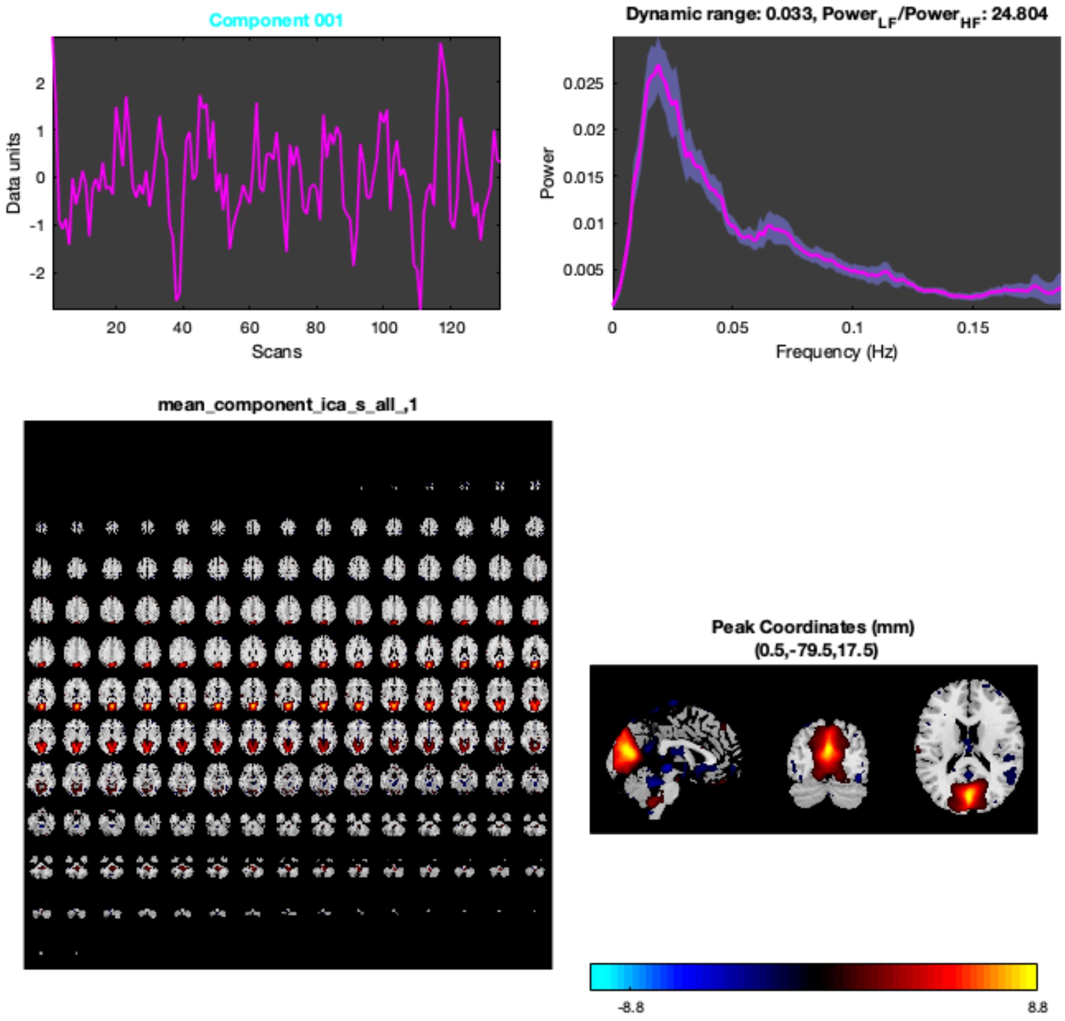

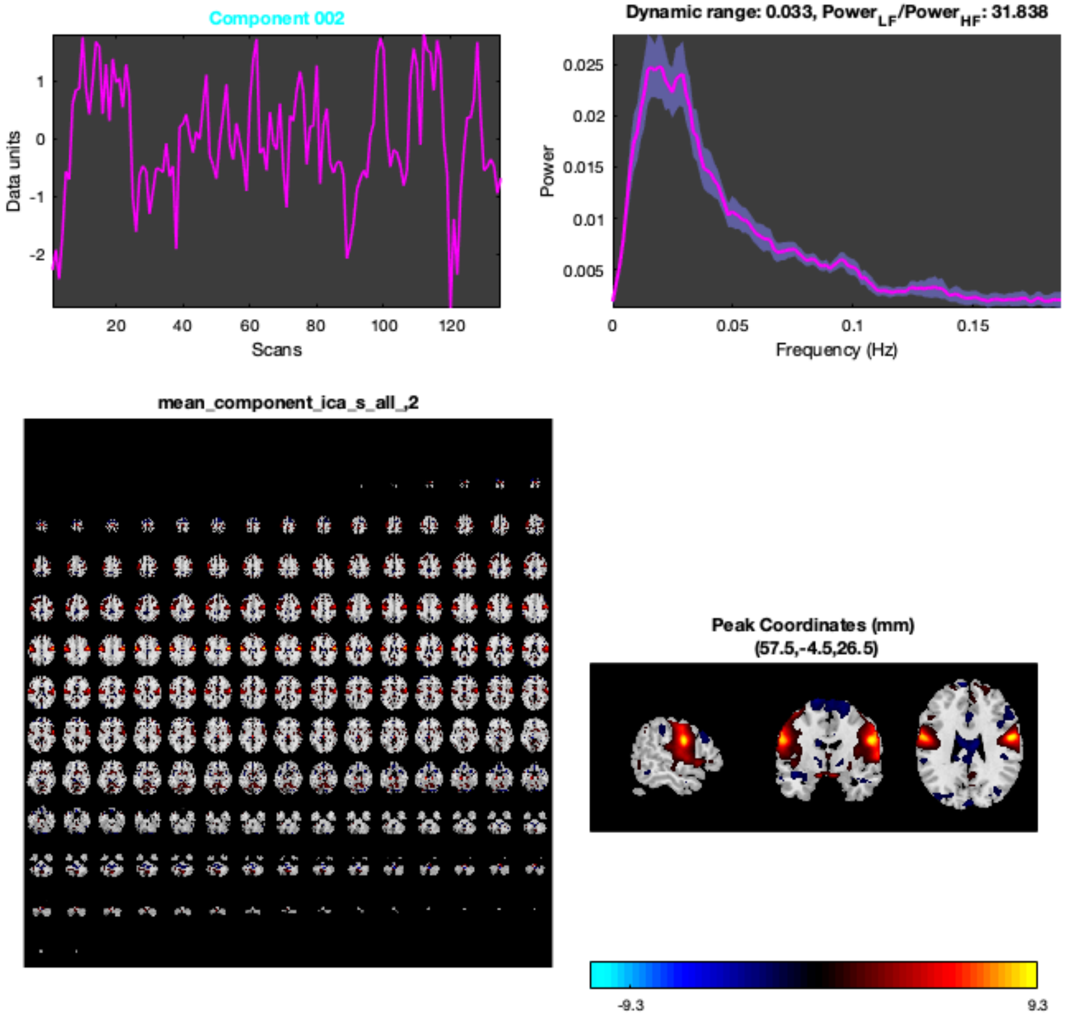
Component 002
Data units
Scans
Dynamic range: 0.033, Power_LF/Power_HF: 31.838
Power
Frequency (Hz)
mean_component_ica_s_all_,2
Peak Coordinates (mm)
(57.5,-4.5,26.5)
-9.3
9.3

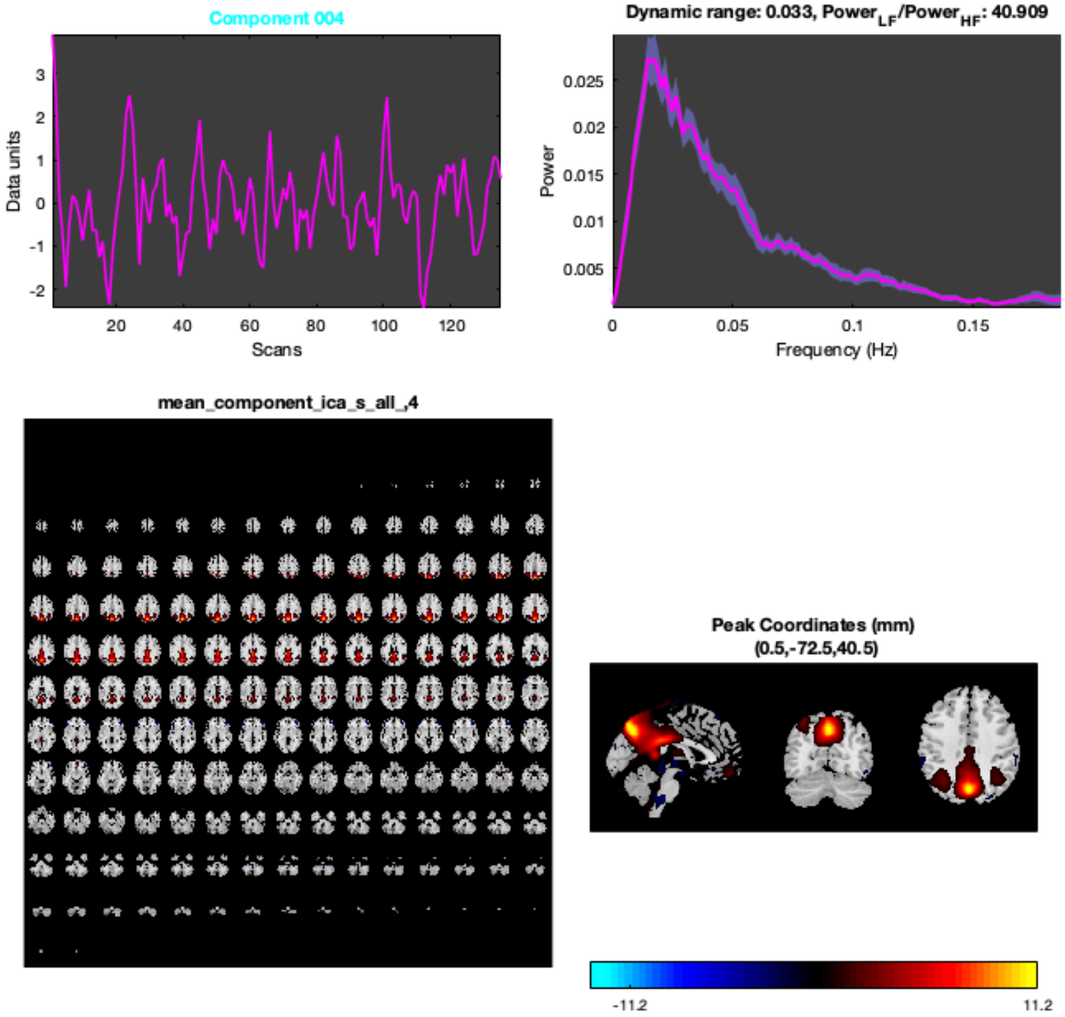
Component 004
Data units
Scans
Dynamic range: 0.033, Power$_{LF}$/Power$_{HF}$: 40.909
Power
Frequency (Hz)
mean_component_ica_s_all_,4
Peak Coordinates (mm)
(0.5,-72.5,40.5)
-11.2
11.2

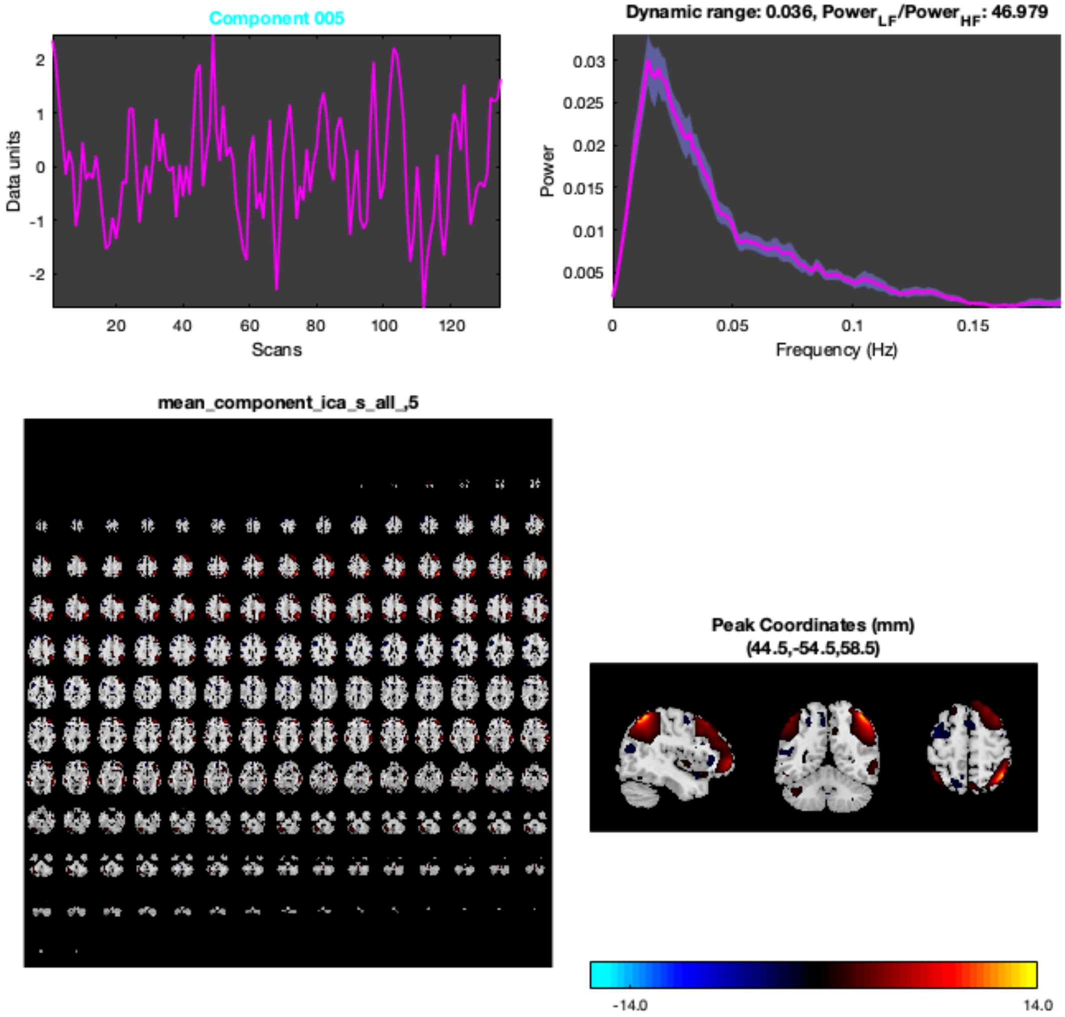

Component 005
Data units
Scans
Dynamic range: 0.036, Power$_{LF}$/Power$_{HF}$: 46.979
Power
Frequency (Hz)
mean_component_ica_s_all_,5
Peak Coordinates (mm)
(44.5,-54.5,58.5)
-14.0
14.0

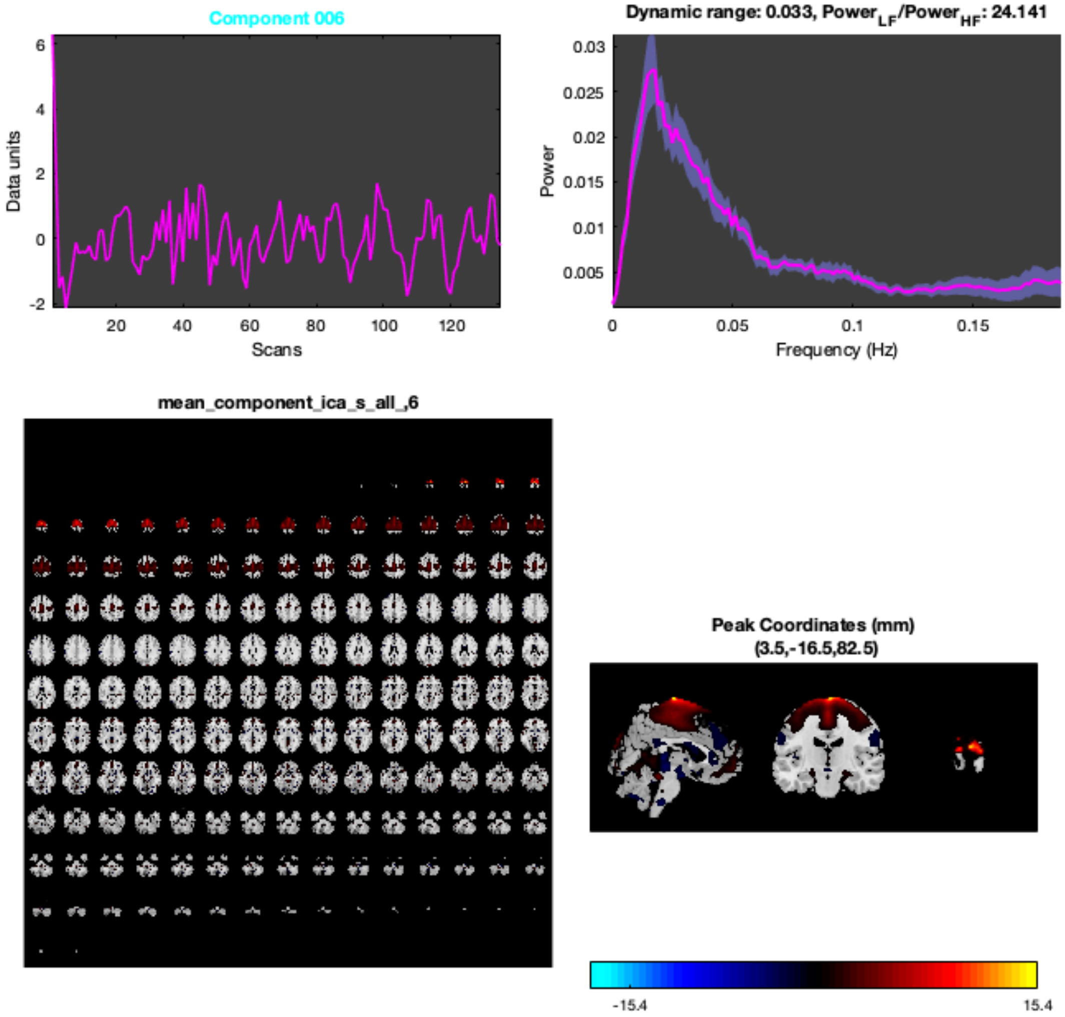
Component 006
Data units
Scans
Dynamic range: 0.033, Power_LF/Power_HF: 24.141
Power
Frequency (Hz)
mean_component_ica_s_all_,6
Peak Coordinates (mm)
(3.5,-16.5,82.5)
-15.4
15.4

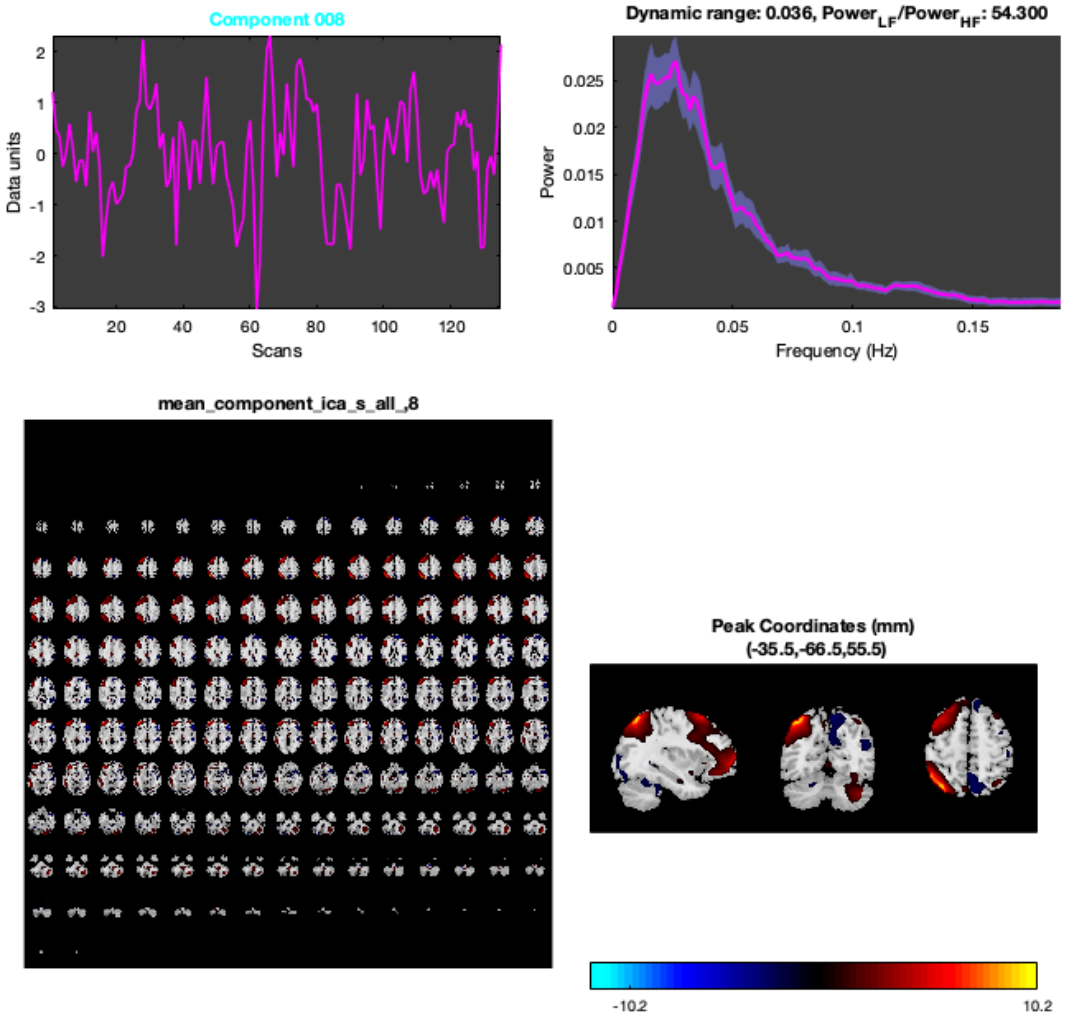

Component 008
Data units
Scans
Dynamic range: 0.036, Power$_{LF}$/Power$_{HF}$: 54.300
Power
Frequency (Hz)
mean_component_ica_s_all_,8
Peak Coordinates (mm)
(-35.5,-66.5,55.5)
-10.2
10.2

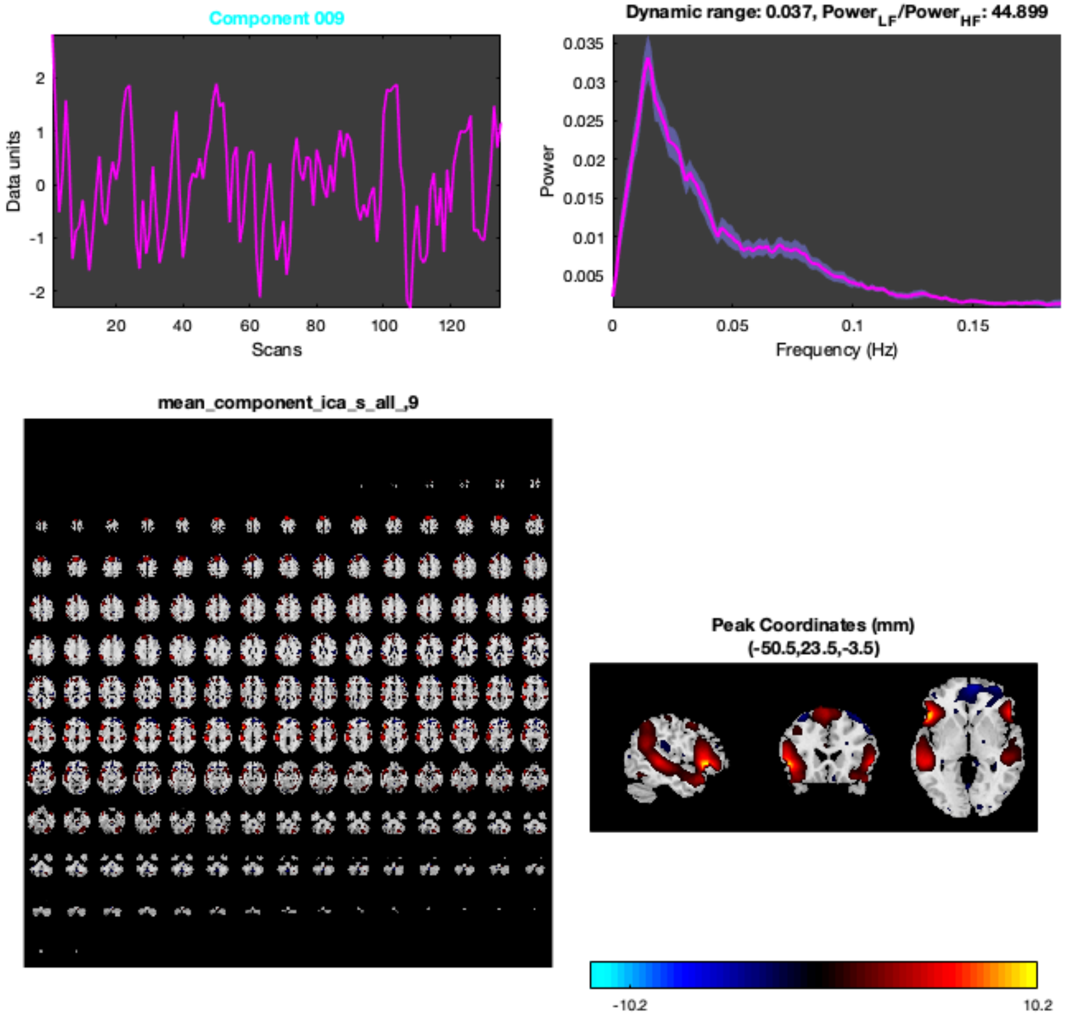

Component 009
Data units
Scans
Dynamic range: 0.037, Power_LF/Power_HF: 44.899
Power
Frequency (Hz)
mean_component_ica_s_all_,9
Peak Coordinates (mm)
(-50.5,23.5,-3.5)
-10.2
10.2

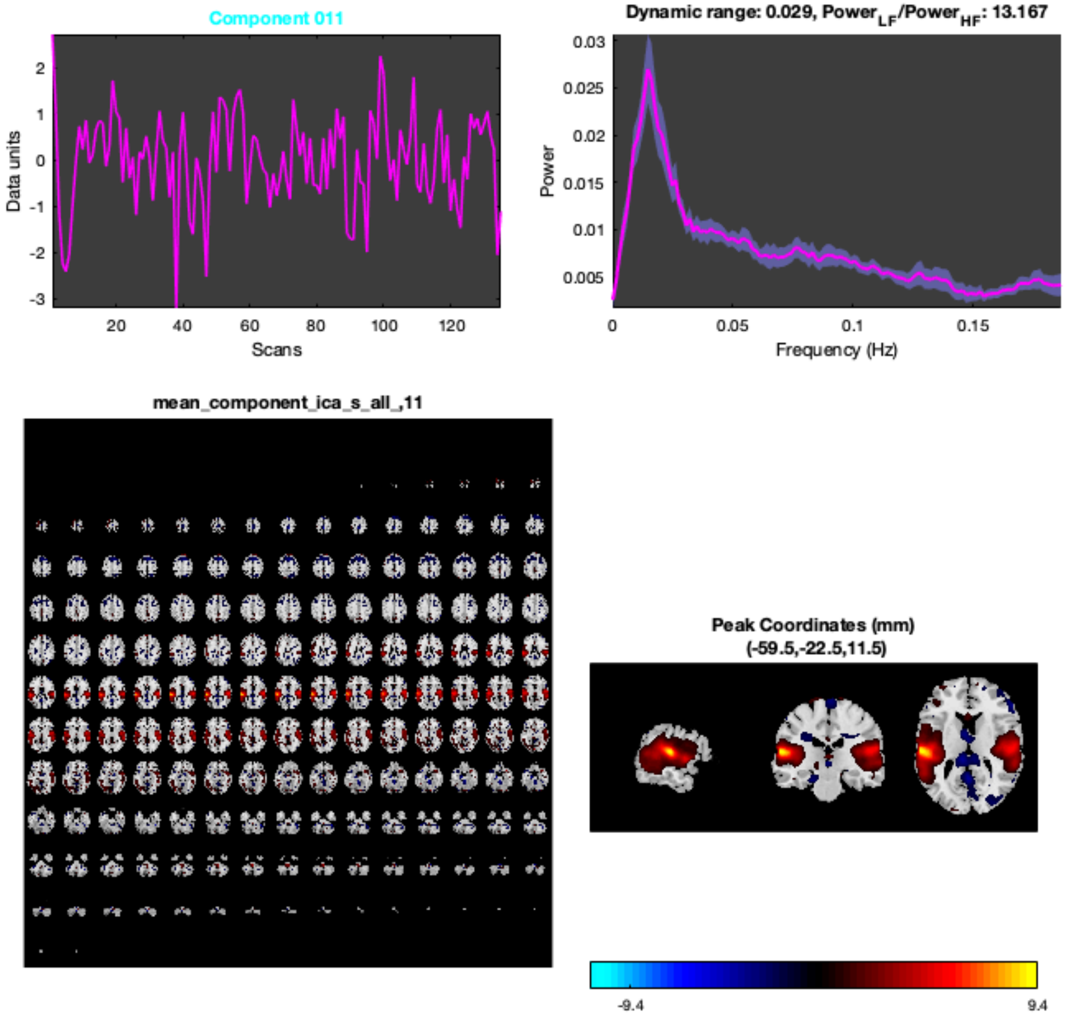

Component 011
Data units
Scans
Dynamic range: 0.029, Power_LF/Power_HF: 13.167
Power
Frequency (Hz)
mean_component_ica_s_all_,11
Peak Coordinates (mm)
(-59.5,-22.5,11.5)
-9.4
9.4

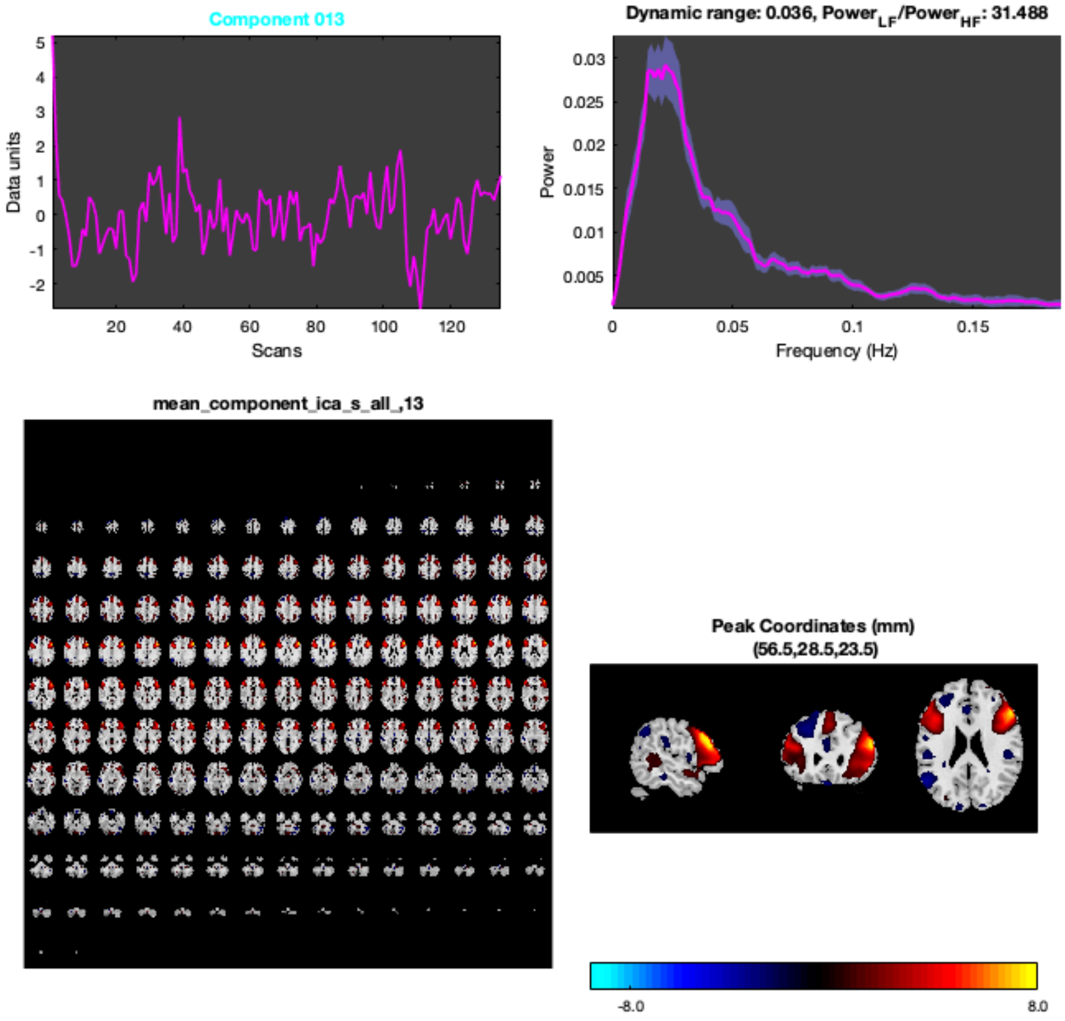
Component 013
Data units
Scans
Dynamic range: 0.036, Power$_{LF}$/Power$_{HF}$: 31.488
Power
Frequency (Hz)
mean_component_ica_s_all_,13
Peak Coordinates (mm)
(56.5,28.5,23.5)
-8.0
8.0

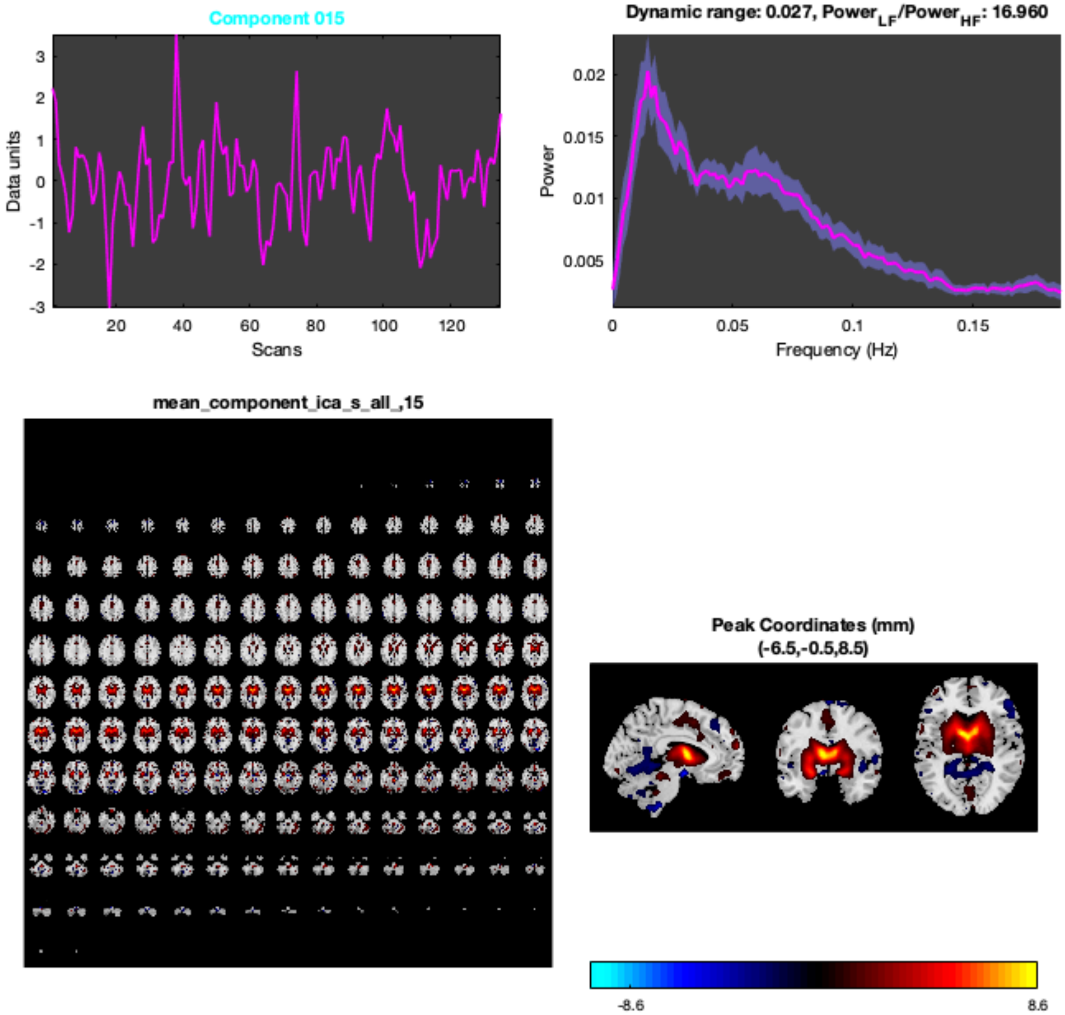
Component 015
Data units
Scans
Dynamic range: 0.027, Power$_{LF}$/Power$_{HF}$: 16.960
Power
Frequency (Hz)
mean_component_ica_s_all_,15
Peak Coordinates (mm)
(-6.5,-0.5,8.5)
-8.6
8.6

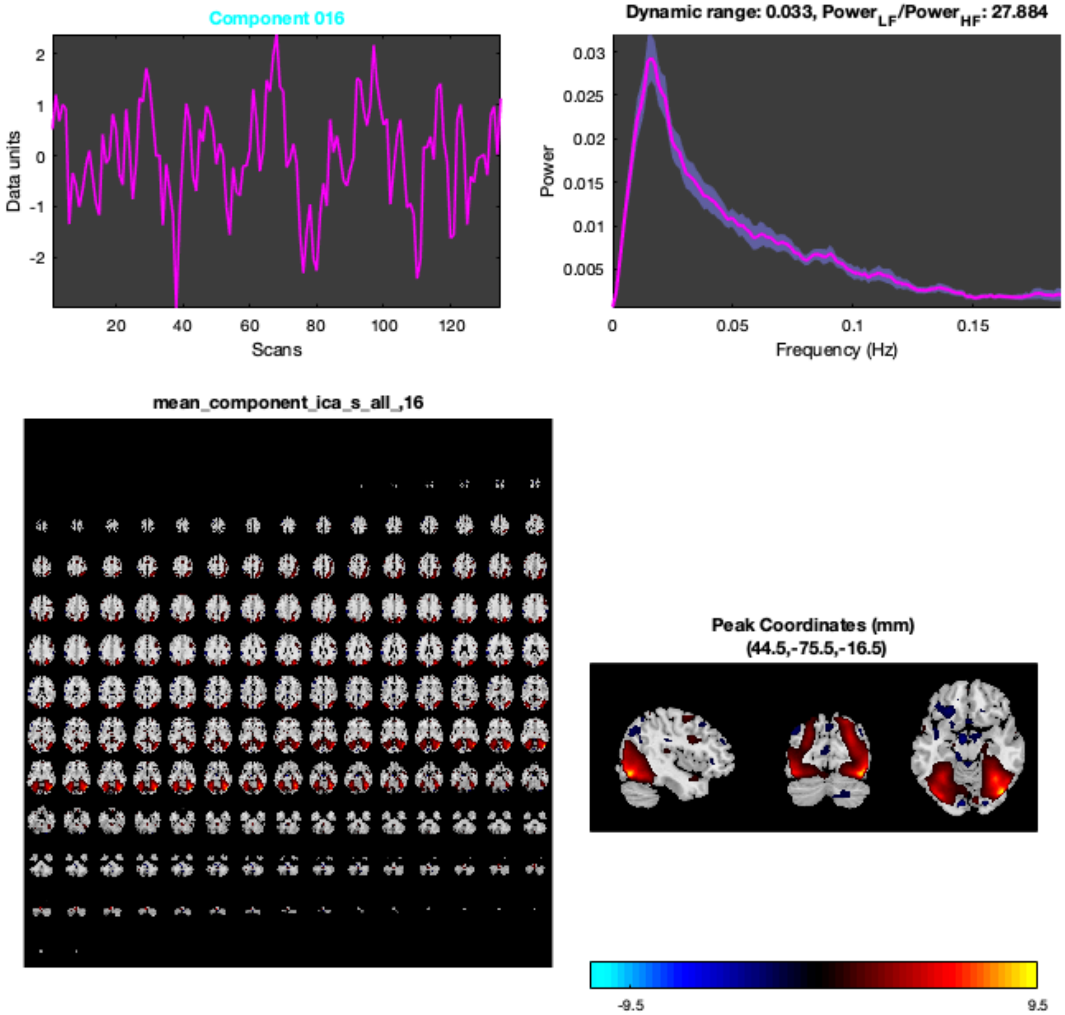
Component 016
Data units
Scans
Dynamic range: 0.033, Power$_{LF}$/Power$_{HF}$: 27.884
Power
Frequency (Hz)
mean_component_ica_s_all_,16
Peak Coordinates (mm)
(44.5,-75.5,-16.5)
-9.5
9.5

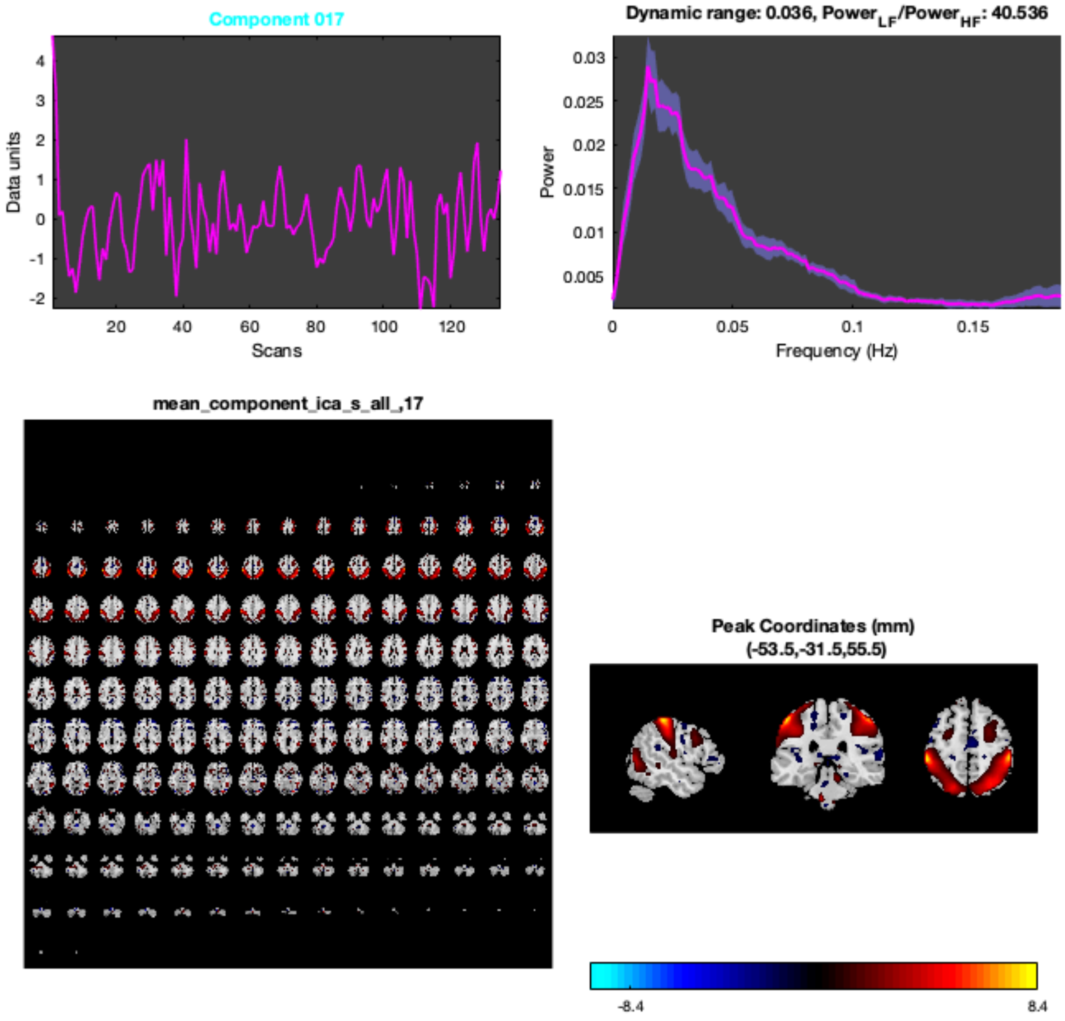
Component 017
Data units
Scans
Dynamic range: 0.036, Power$_{LF}$/Power$_{HF}$: 40.536
Power
Frequency (Hz)
mean_component_ica_s_all_,17
Peak Coordinates (mm)
(-53.5,-31.5,55.5)
-8.4
8.4

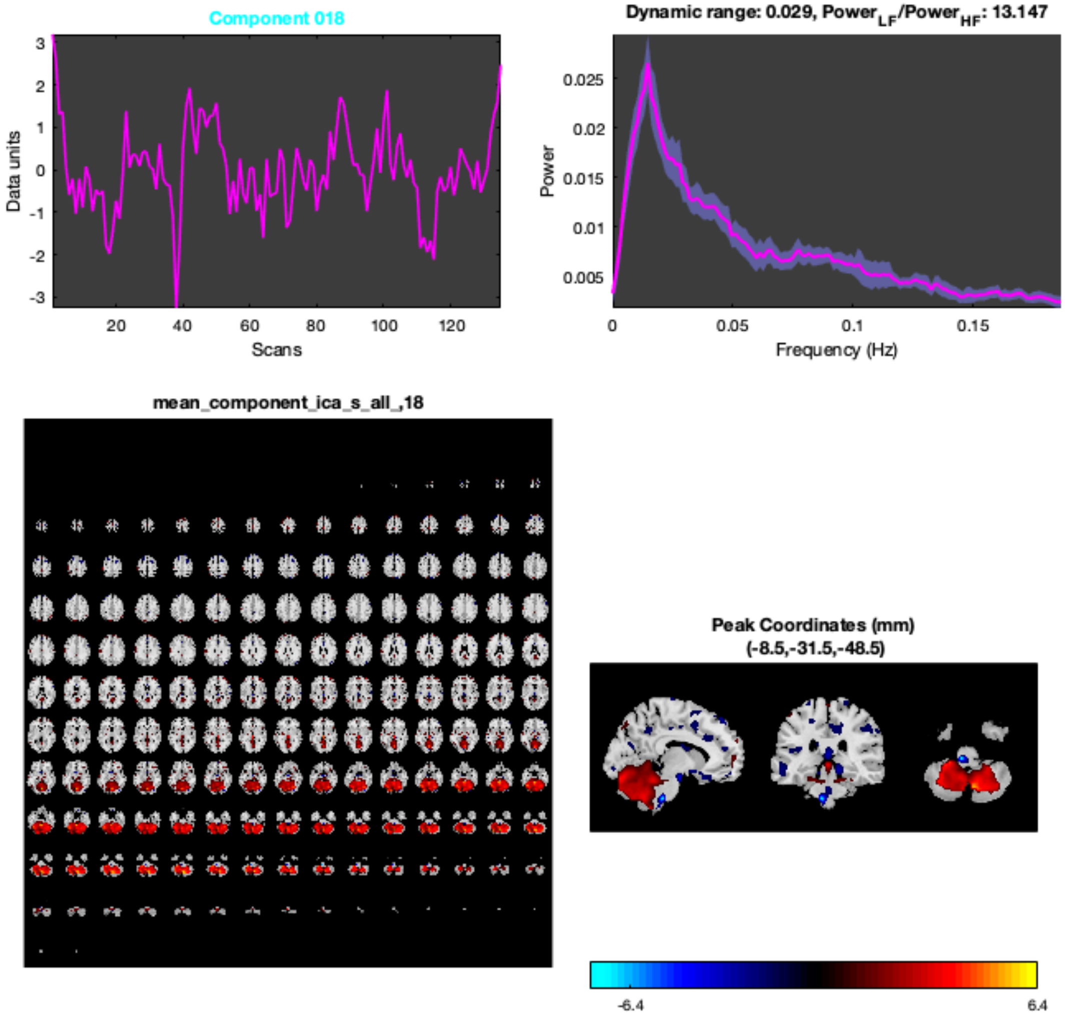
Component 018
Data units
Scans
Dynamic range: 0.029, Power$_{LF}$/Power$_{HF}$: 13.147
Power
Frequency (Hz)
mean_component_ica_s_all_,18
Peak Coordinates (mm)
(-8.5,-31.5,-48.5)
-6.4
6.4

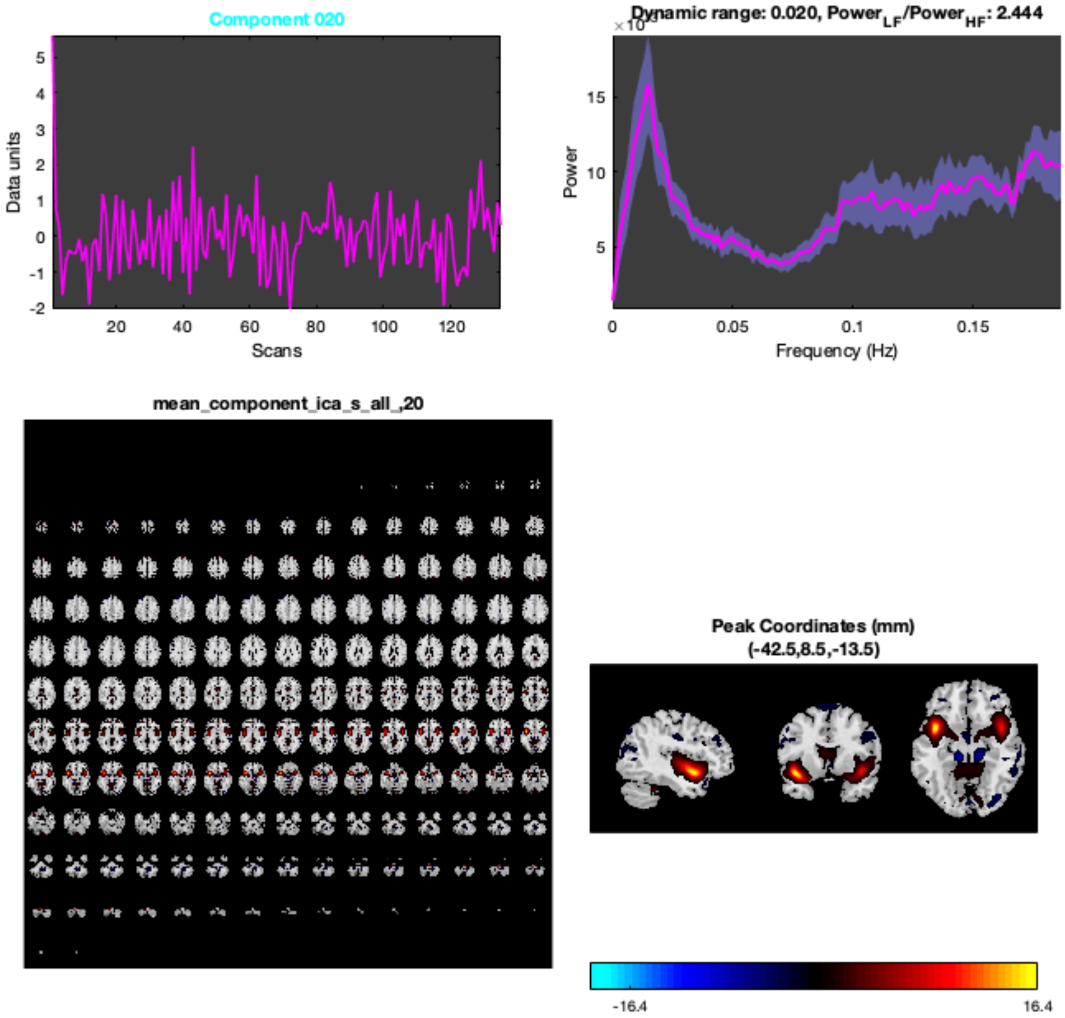
Component 020
Data units
Scans
Dynamic range: 0.020, Power_LF/Power_HF: 2.444
Power
Frequency (Hz)
mean_component_ica_s_all_,20
Peak Coordinates (mm)
(-42.5,8.5,-13.5)
-16.4
16.4

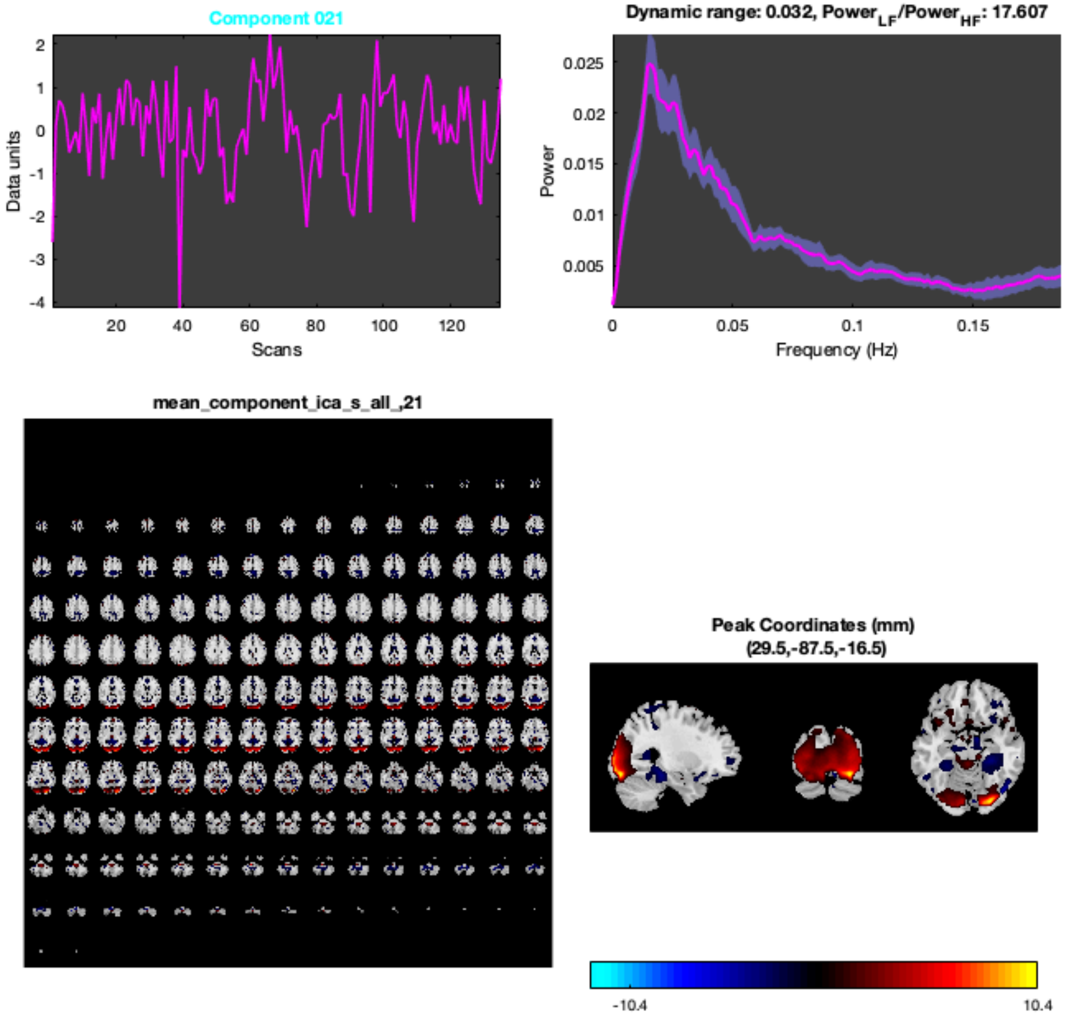

Component 021
Data units
Scans
Dynamic range: 0.032, Power_LF/Power_HF: 17.607
Power
Frequency (Hz)
mean_component_ica_s_all_,21
Peak Coordinates (mm)
(29.5,-87.5,-16.5)
-10.4
10.4

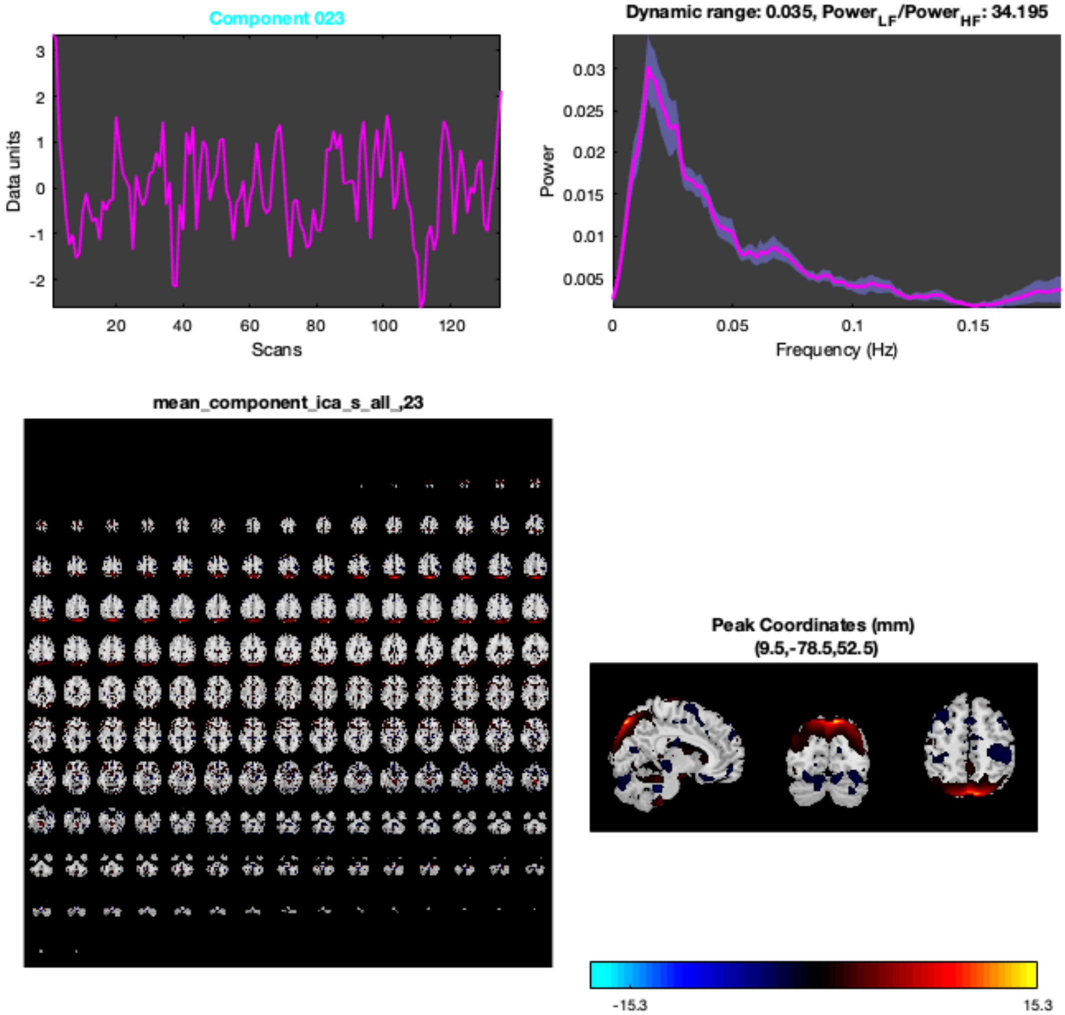
Component 023
Data units
Scans
Dynamic range: 0.035, Power$_{LF}$/Power$_{HF}$: 34.195
Power
Frequency (Hz)
mean_component_ica_s_all_,23
Peak Coordinates (mm)
(9.5,-78.5,52.5)
-15.3
15.3

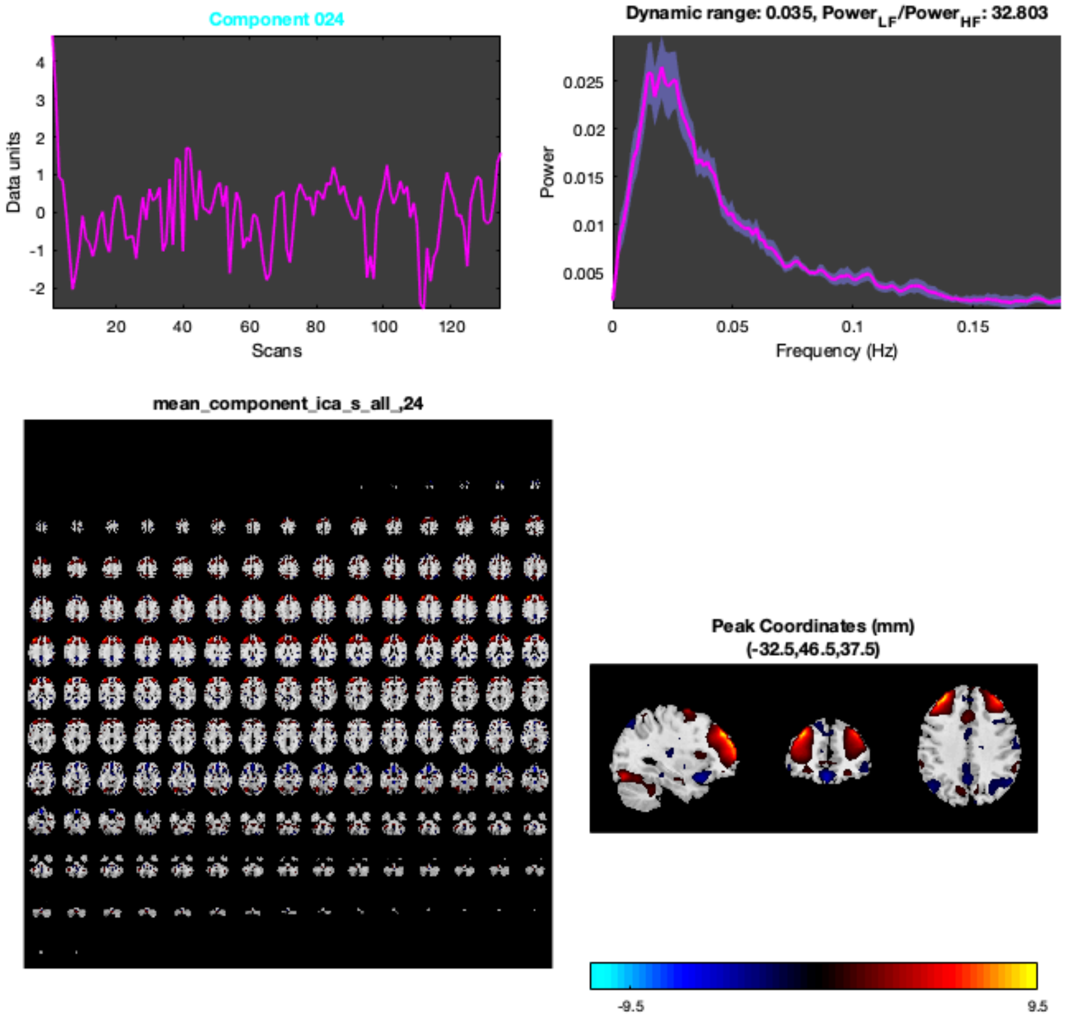
Component 024
Data units
Scans
Dynamic range: 0.035, Power$_{LF}$/Power$_{HF}$: 32.803
Power
Frequency (Hz)
mean_component_ica_s_all_,24
Peak Coordinates (mm)
(-32.5,46.5,37.5)
-9.5
9.5

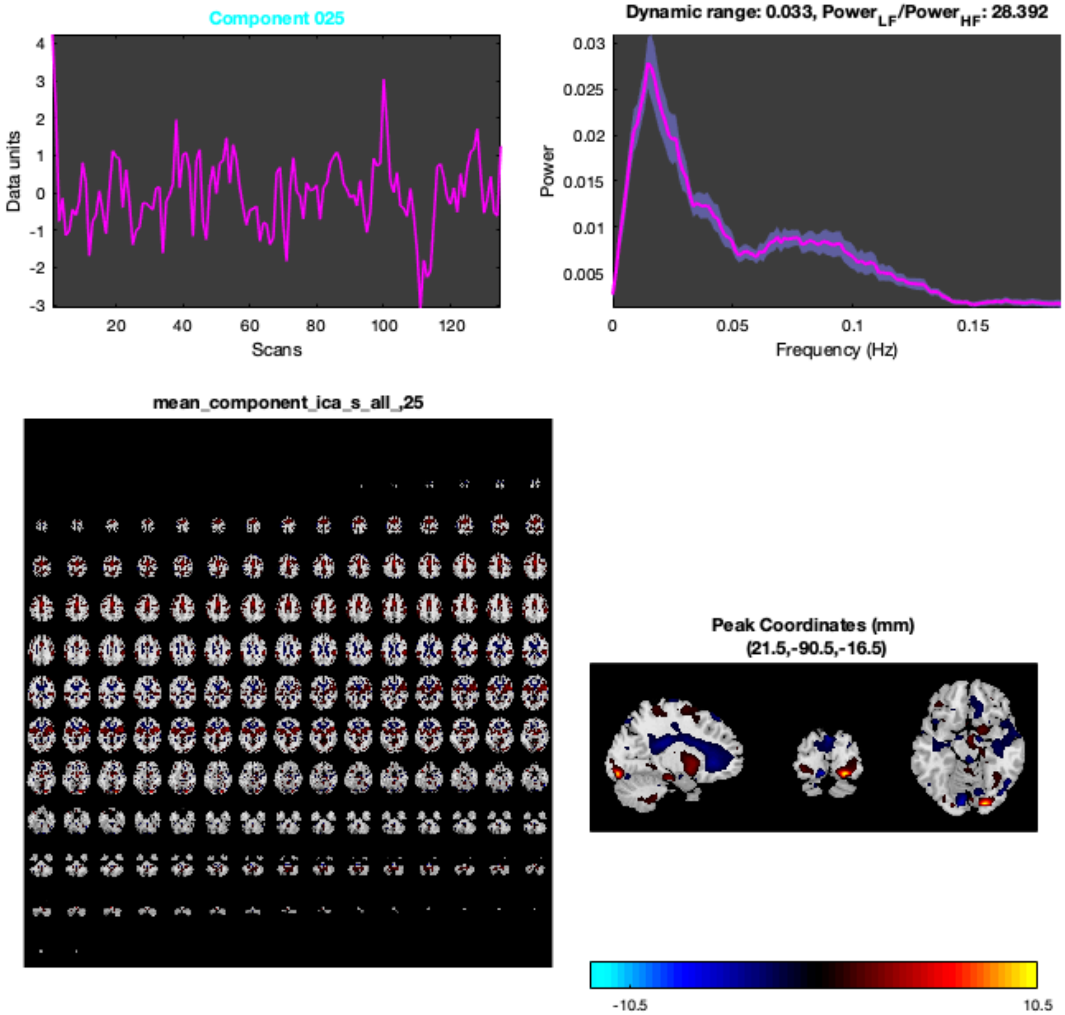
Component 025
Data units
Scans
Dynamic range: 0.033, Power$_{LF}$/Power$_{HF}$: 28.392
Power
Frequency (Hz)
mean_component_ica_s_all_,25
Peak Coordinates (mm)
(21.5,-90.5,-16.5)
-10.5
10.5

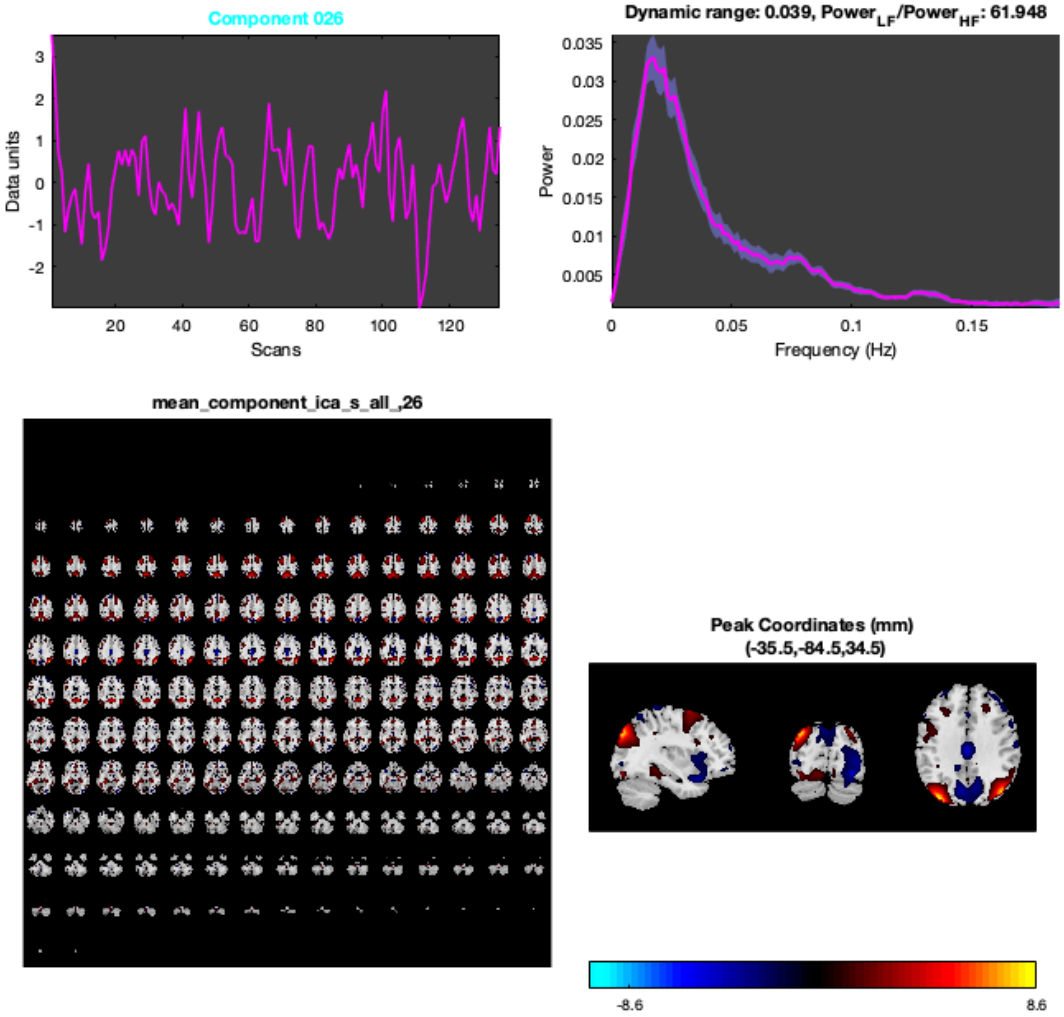
Component 026
Data units
Scans
Dynamic range: 0.039, Power_LF/Power_HF: 61.948
Power
Frequency (Hz)
mean_component_ica_s_all_,26
Peak Coordinates (mm)
(-35.5,-84.5,34.5)
-8.6
8.6

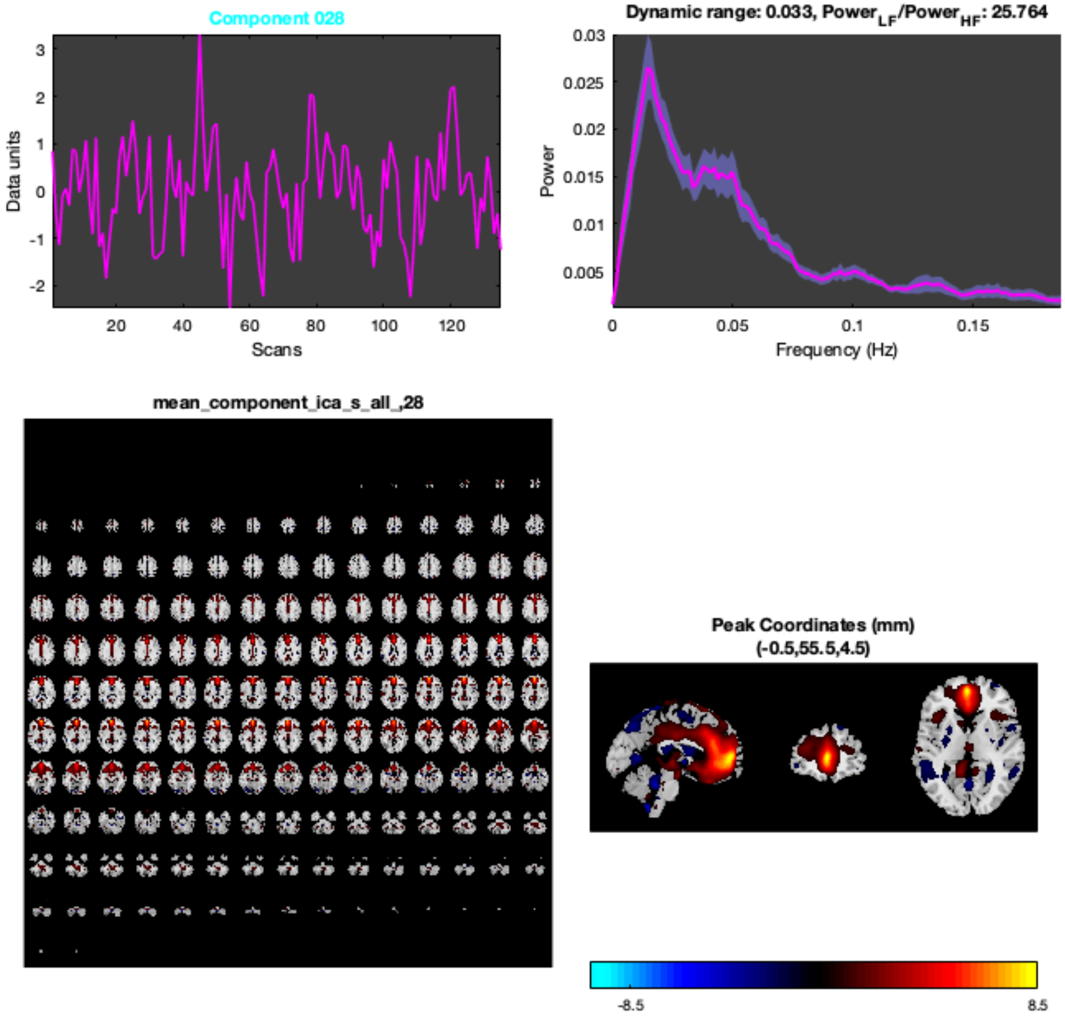
Component 028
Data units
Scans
Dynamic range: 0.033, Power$_{LF}$/Power$_{HF}$: 25.764
Power
Frequency (Hz)
mean_component_ica_s_all_,28
Peak Coordinates (mm)
(-0.5,55.5,4.5)
-8.5
8.5

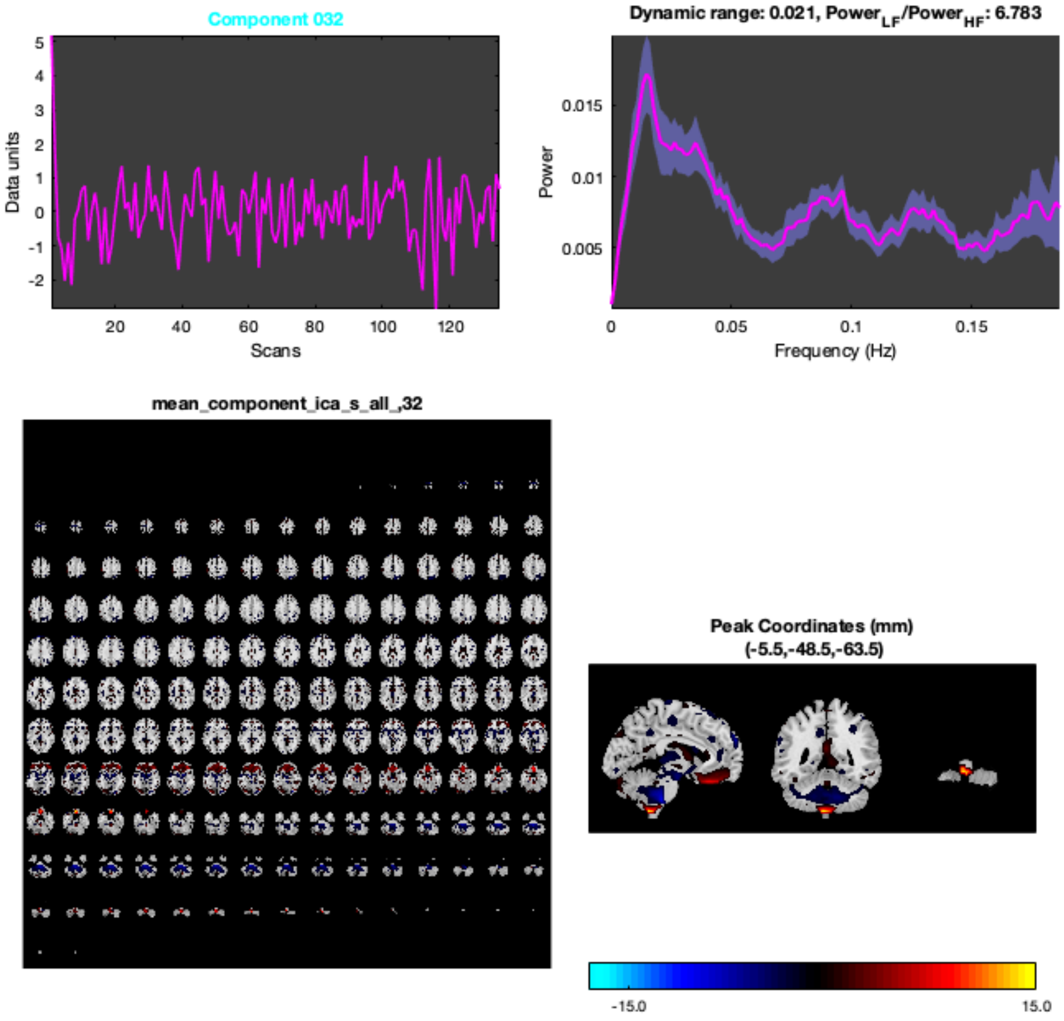
Component 032
Data units
Scans
Dynamic range: 0.021, Power$_{LF}$/Power$_{HF}$: 6.783
Power
Frequency (Hz)
mean_component_ica_s_all_,32
Peak Coordinates (mm)
(-5.5,-48.5,-63.5)
-15.0
15.0
