# Supplementary material for: Neural Correlates of Vocal Pitch Compensation in Individuals Who Stutter
Source: Front Hum Neurosci. 2020 Feb 25;14:18. doi: 10.3389/fnhum.2020.00018 (PMC7053555; doi:10.3389/fnhum.2020.00018)
Supplement: Supplementary file 4 [file Data_Sheet_3.PDF]

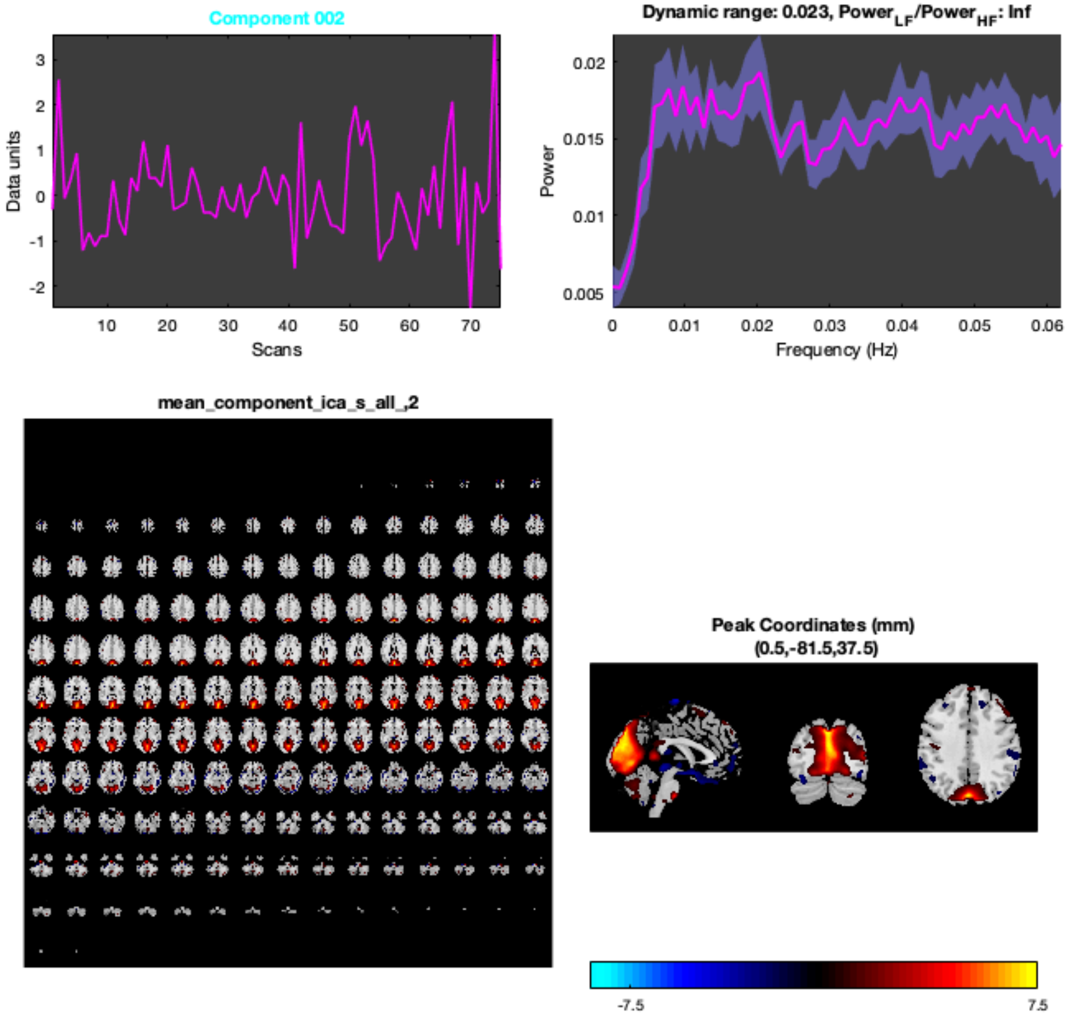
Component 002
Data units
Scans
Dynamic range: 0.023, Power$_{LF}$/Power$_{HF}$: Inf
Power
Frequency (Hz)
mean_component_ica_s_all_,2
Peak Coordinates (mm)
(0.5,-81.5,37.5)
-7.5
7.5

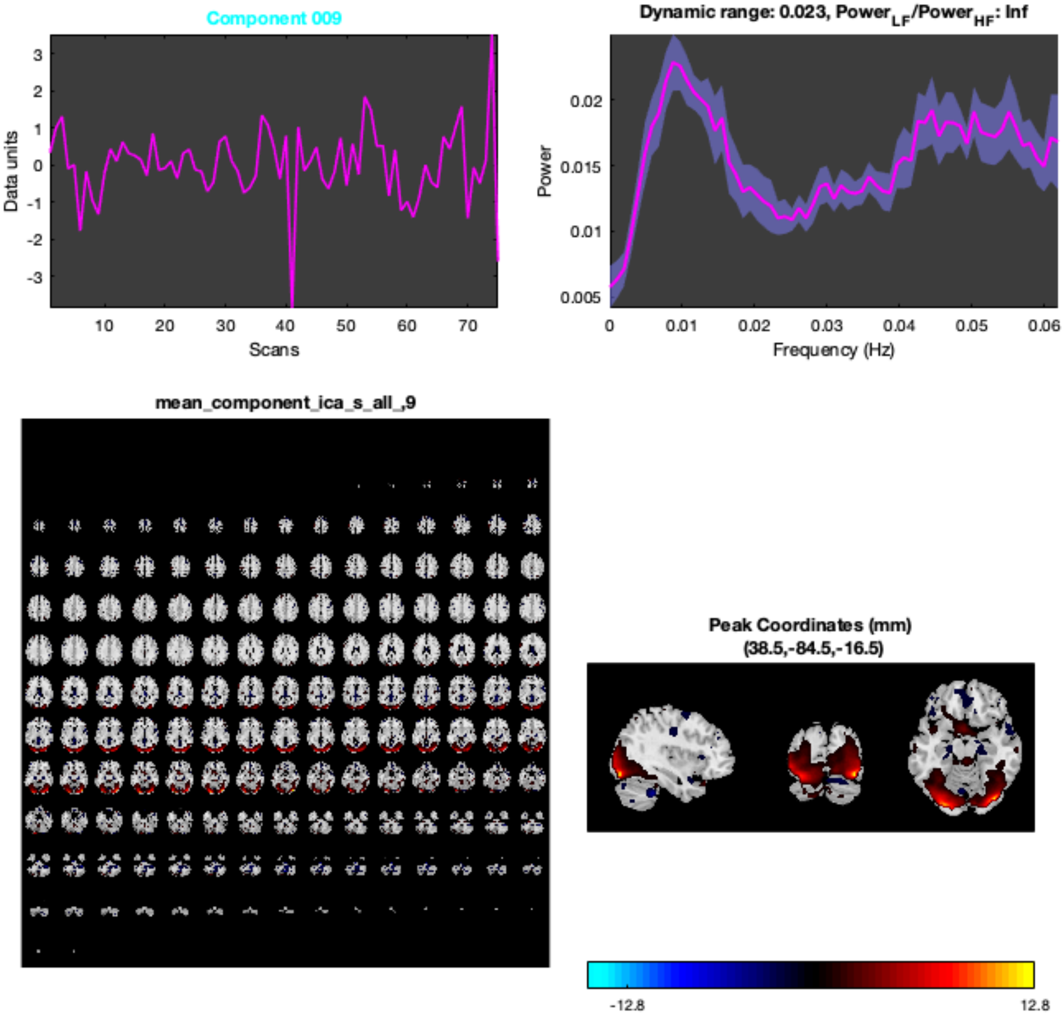
Component 009
Data units
Scans
Dynamic range: 0.023, Power$_{LF}$/Power$_{HF}$: Inf
Power
Frequency (Hz)
mean_component_ica_s_all_,9
Peak Coordinates (mm)
(38.5,-84.5,-16.5)
-12.8
12.8

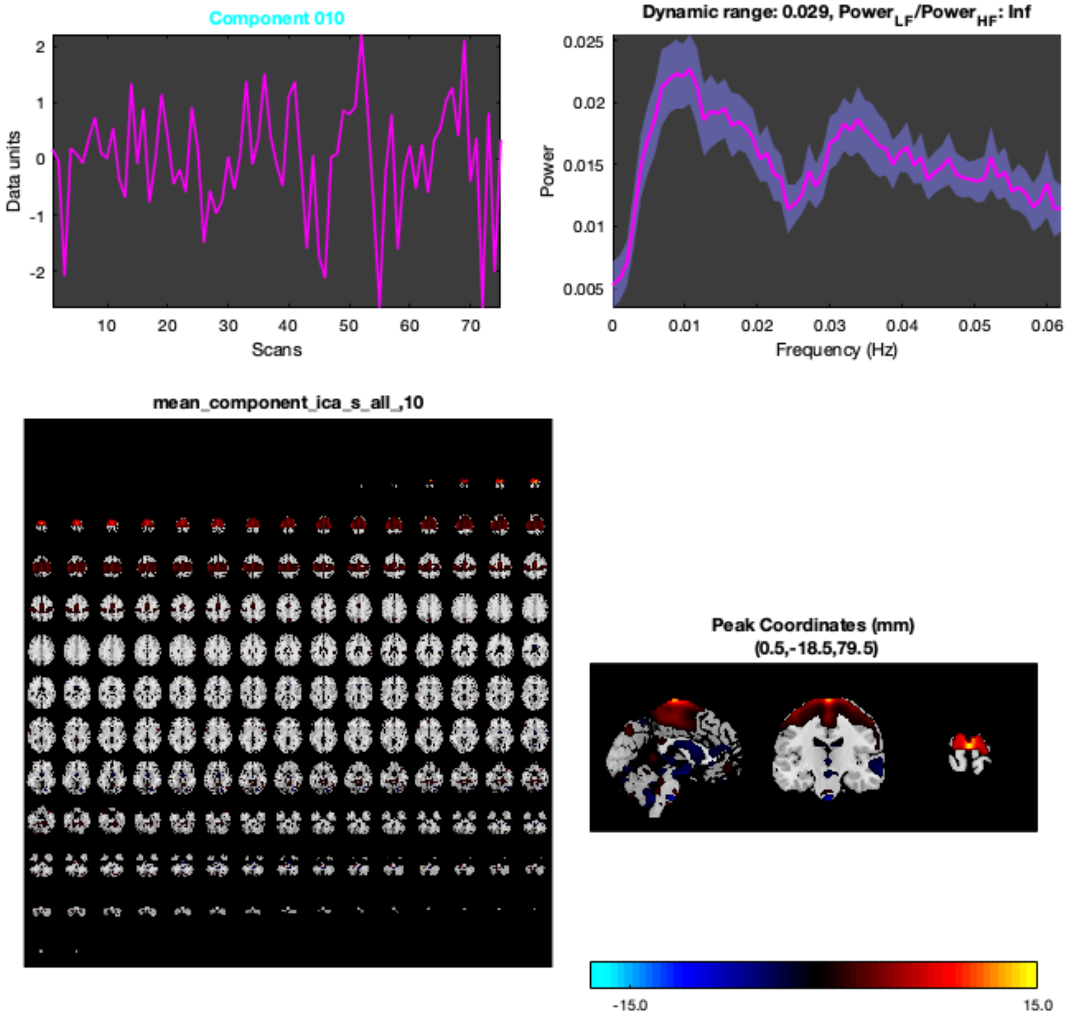

Component 010
Data units
Scans
Dynamic range: 0.029, Power$_{LF}$/Power$_{HF}$: Inf
Power
Frequency (Hz)
mean_component_ica_s_all_,10
Peak Coordinates (mm)
(0.5,-18.5,79.5)
-15.0
15.0

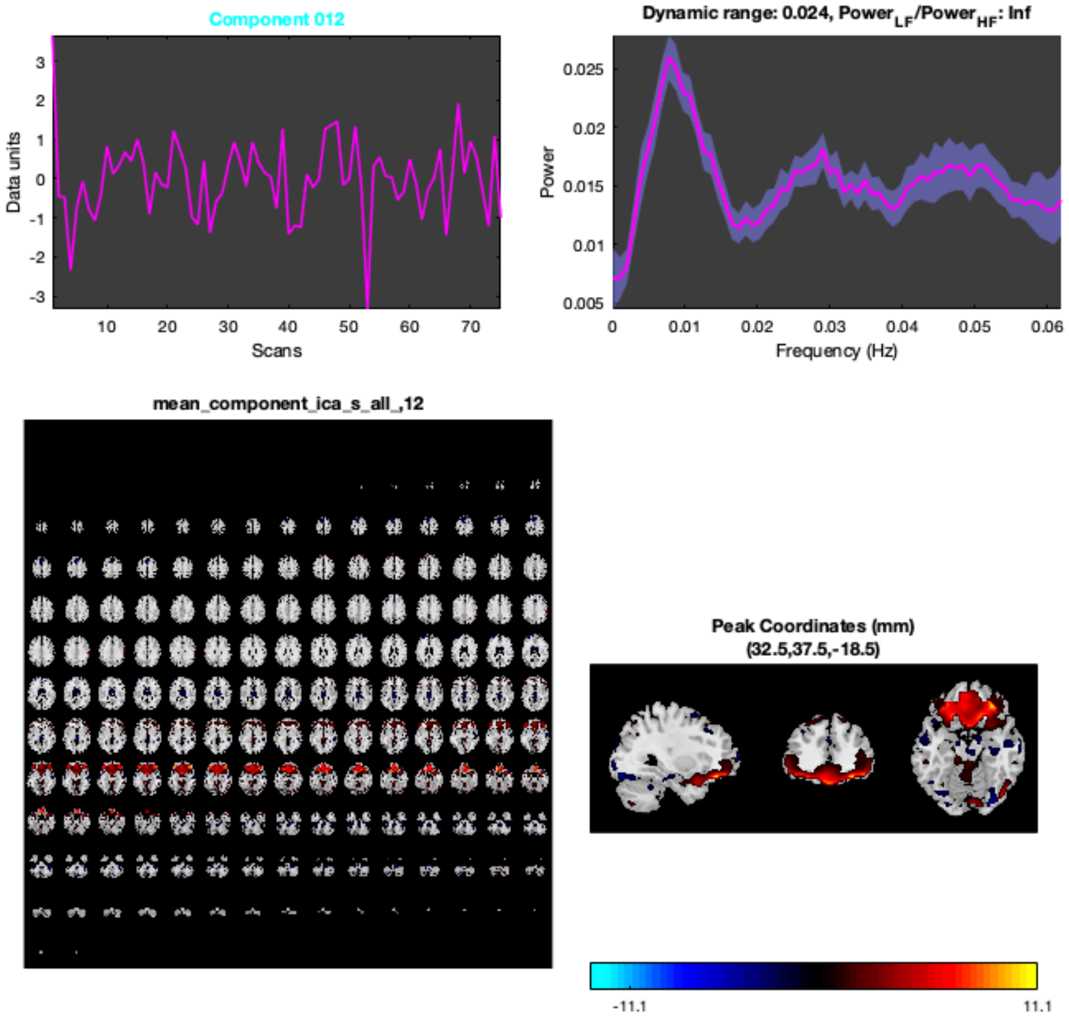
Component 012
Data units
Scans
Dynamic range: 0.024, Power_LF/Power_HF: Inf
Power
Frequency (Hz)
mean_component_ica_s_all_,12
Peak Coordinates (mm)
(32.5,37.5,-18.5)
-11.1
11.1

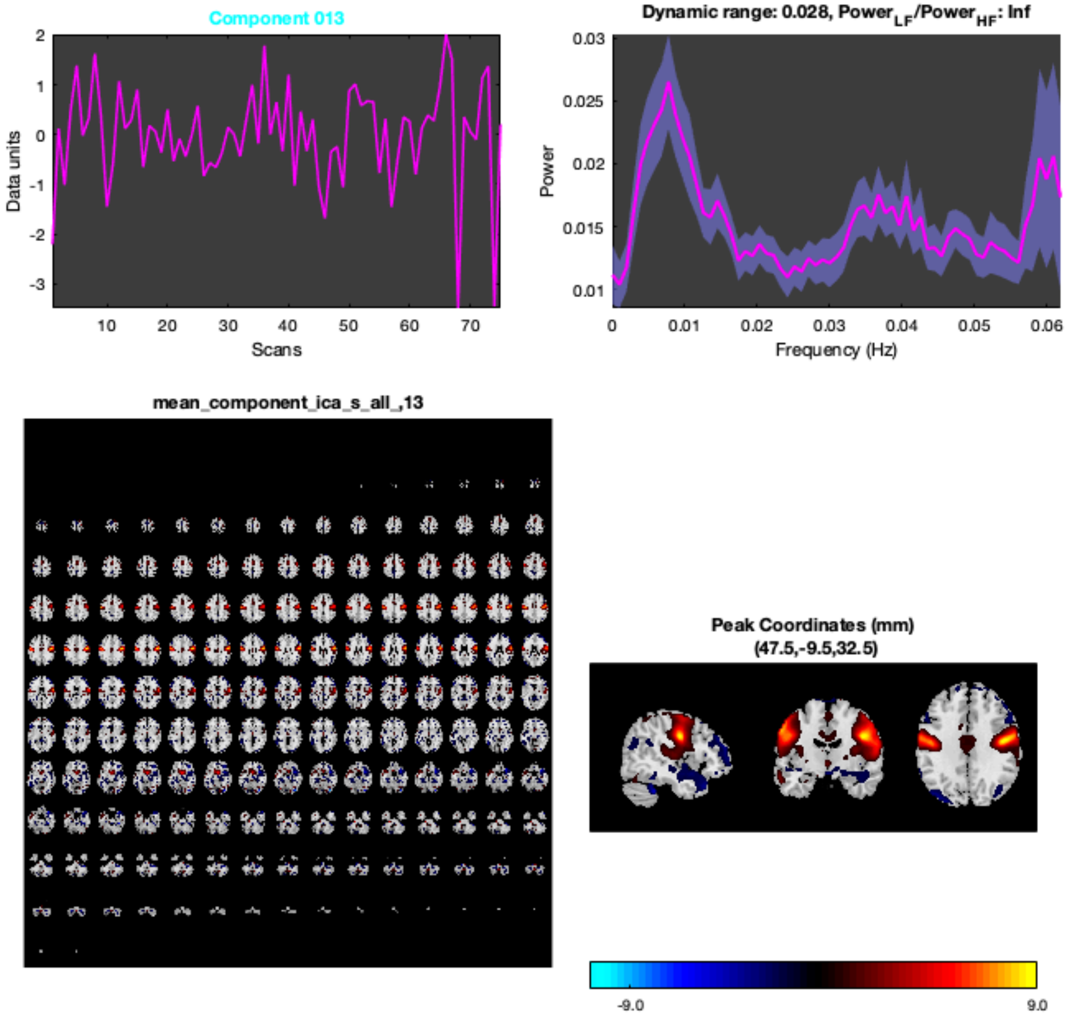
Component 013
Data units
Scans
Dynamic range: 0.028, Power_LF/Power_HF: Inf
Power
Frequency (Hz)
mean_component_ica_s_all_,13
Peak Coordinates (mm)
(47.5,-9.5,32.5)
-9.0
9.0

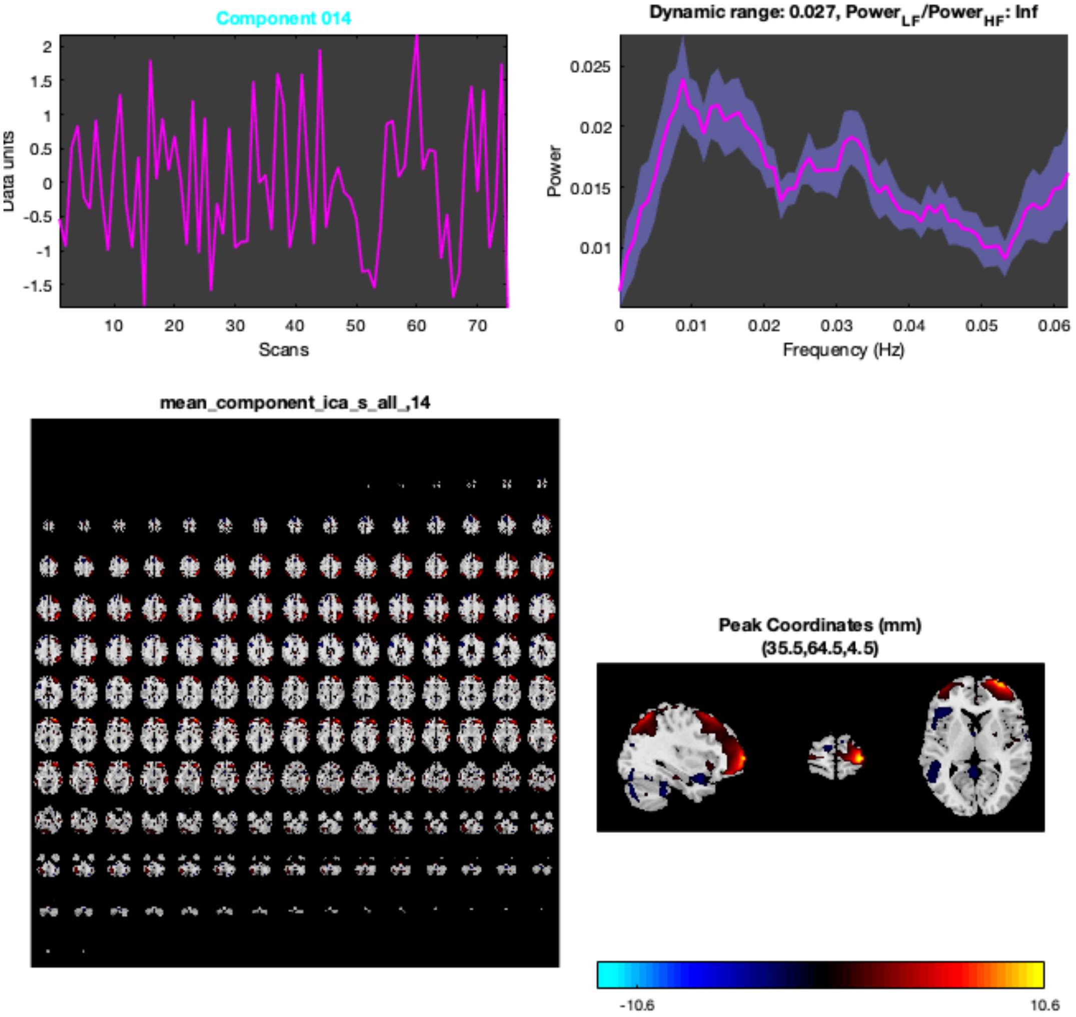
Component 014
Data units
Scans
Dynamic range: 0.027, Power$_{LF}$/Power$_{HF}$: Inf
Power
Frequency (Hz)
mean_component_ica_s_all_,14
Peak Coordinates (mm)
(35.5,64.5,4.5)
-10.6
10.6

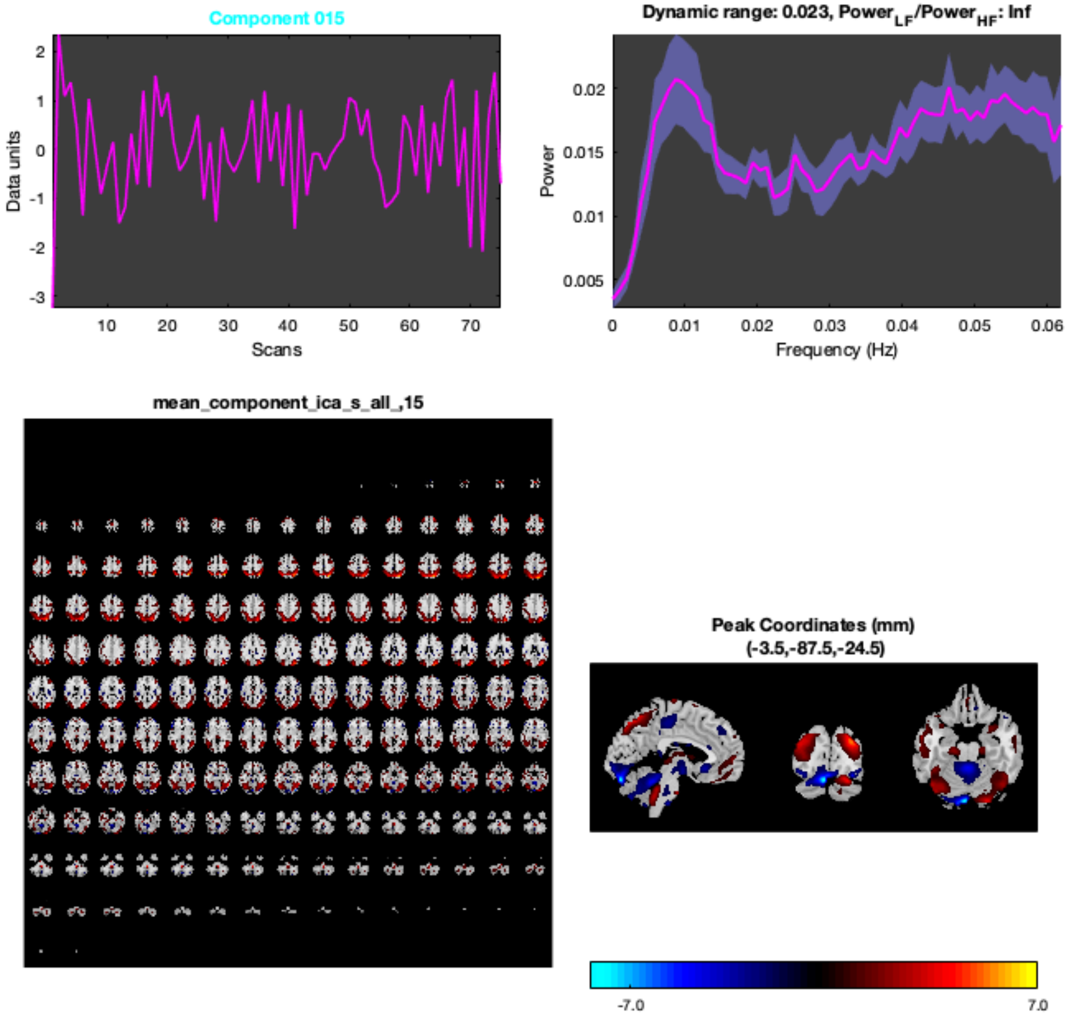
Component 015
Data units
Scans
Dynamic range: 0.023, Power_LF/Power_HF: Inf
Power
Frequency (Hz)
mean_component_ica_s_all_,15
Peak Coordinates (mm)
(-3.5,-87.5,-24.5)
-7.0
7.0

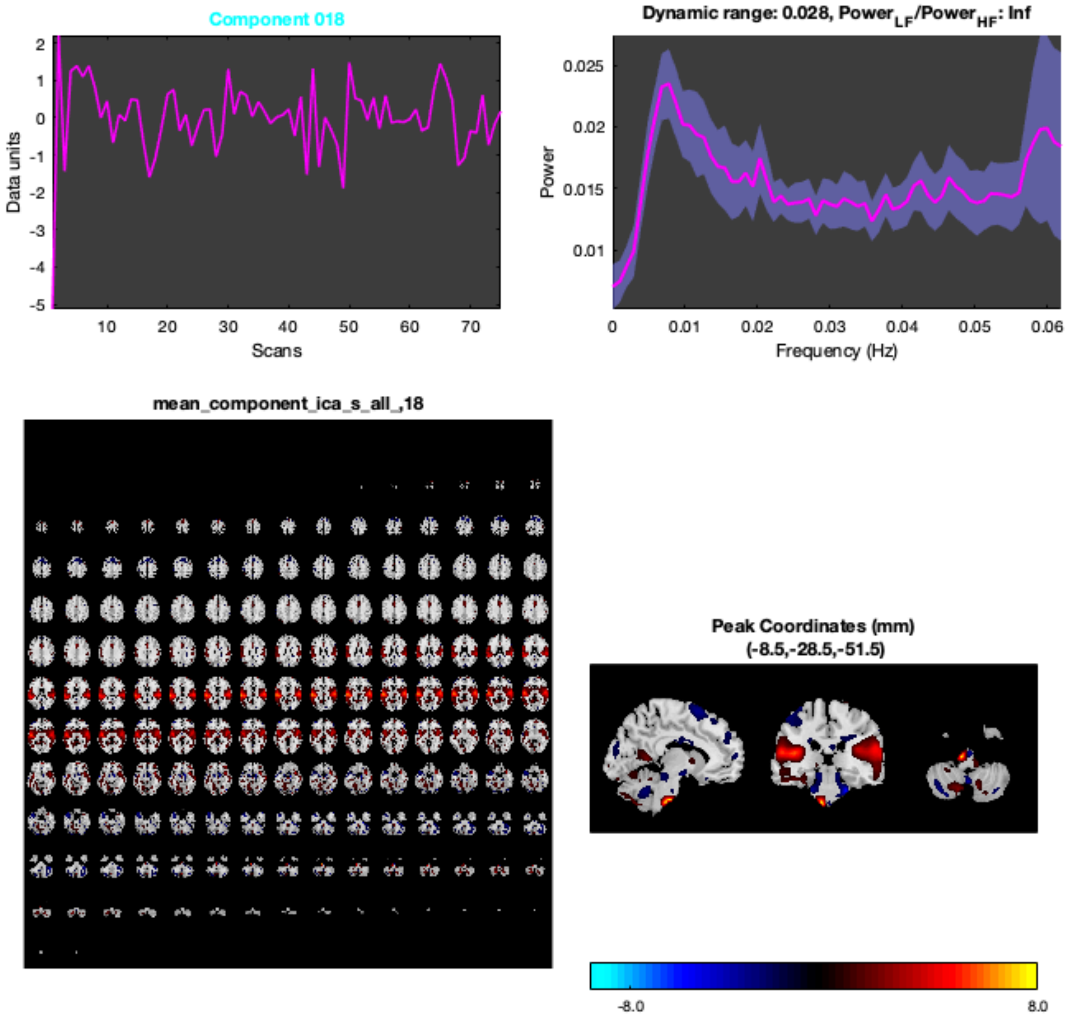
Component 018
Data units
Scans
Dynamic range: 0.028, Power$_{LF}$/Power$_{HF}$: Inf
Power
Frequency (Hz)
mean_component_ica_s_all_,18
Peak Coordinates (mm)
(-8.5,-28.5,-51.5)
-8.0
8.0

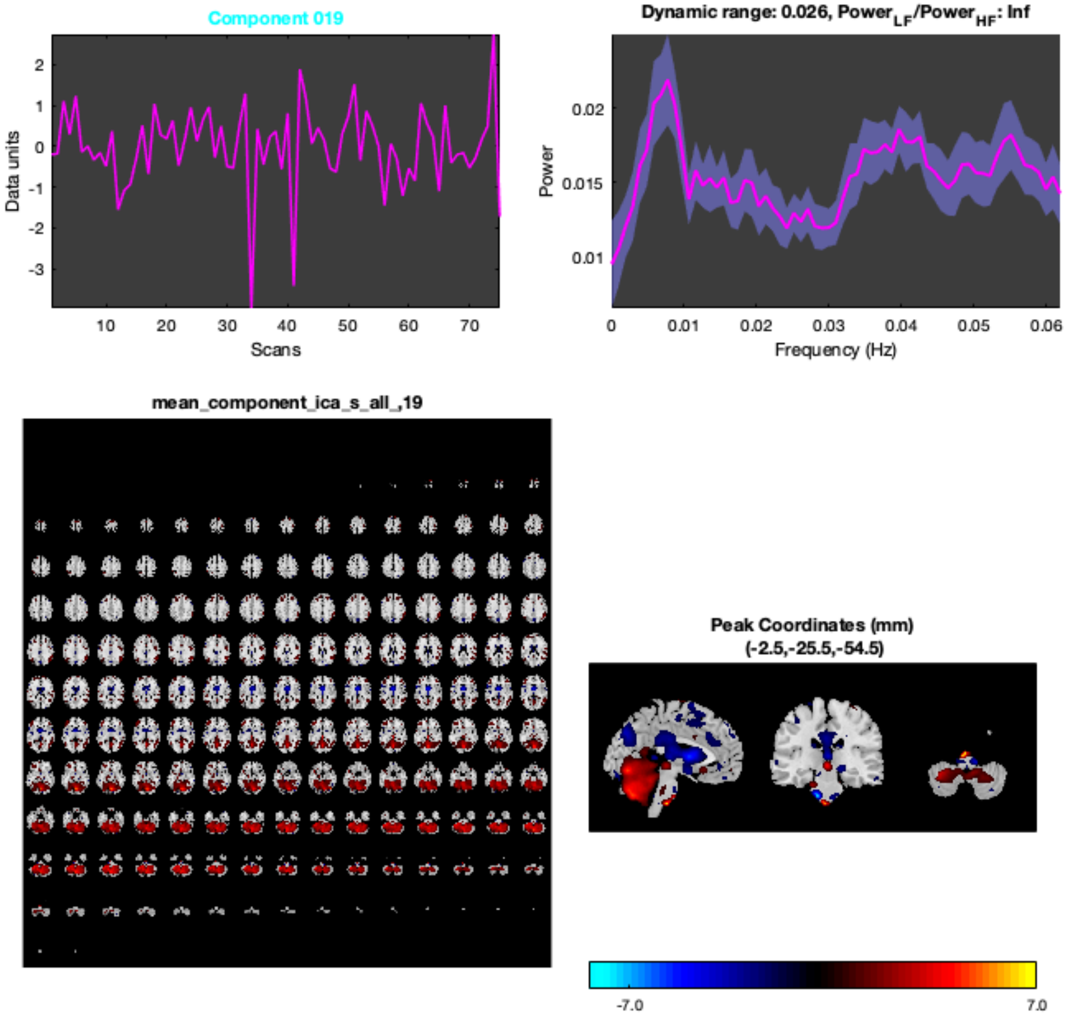
Component 019
Data units
Scans
Dynamic range: 0.026, Power$_{LF}$/Power$_{HF}$: Inf
Power
Frequency (Hz)
mean_component_ica_s_all_,19
Peak Coordinates (mm)
(-2.5,-25.5,-54.5)
-7.0
7.0

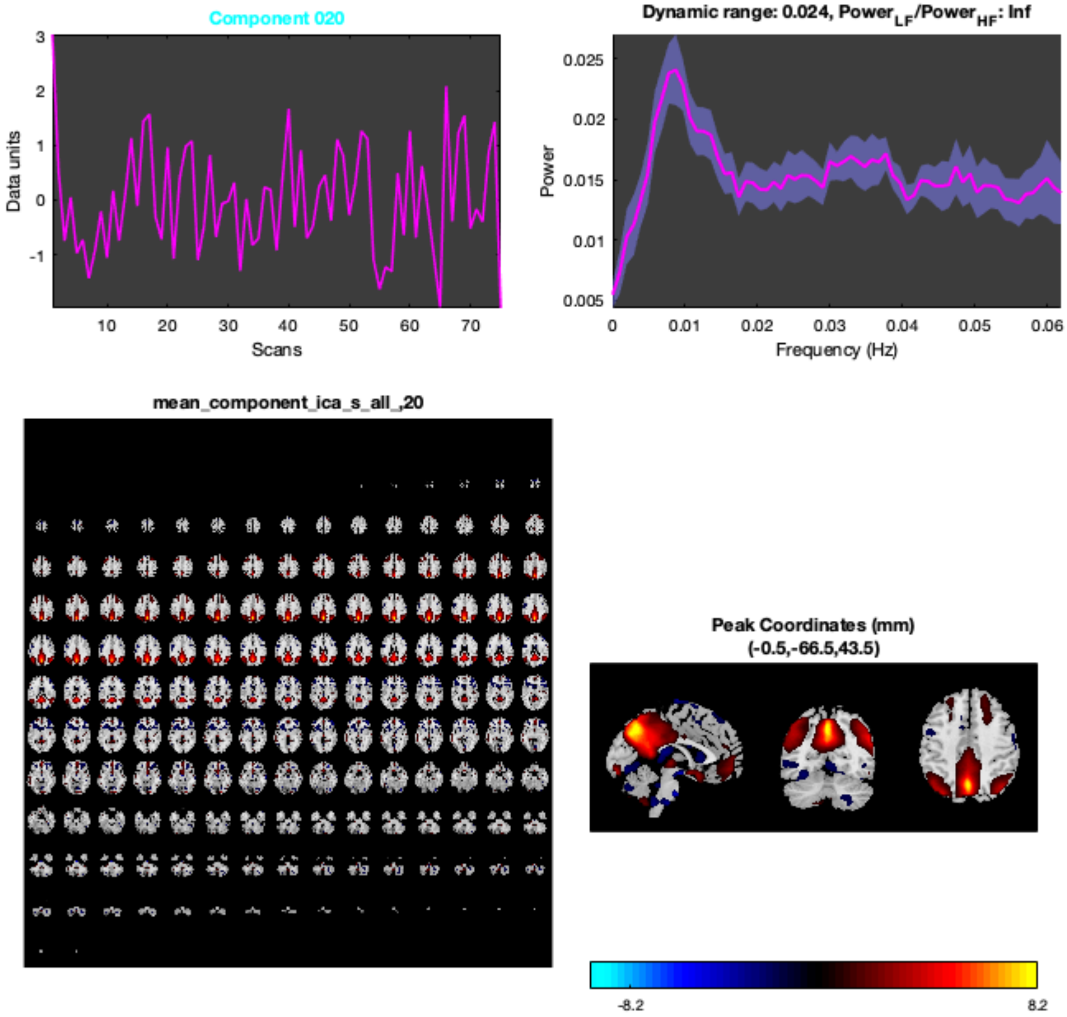
Component 020
Data units
Scans
Dynamic range: 0.024, Power$_{LF}$/Power$_{HF}$: Inf
Power
Frequency (Hz)
mean_component_ica_s_all_,20
Peak Coordinates (mm)
(-0.5,-66.5,43.5)
-8.2
8.2

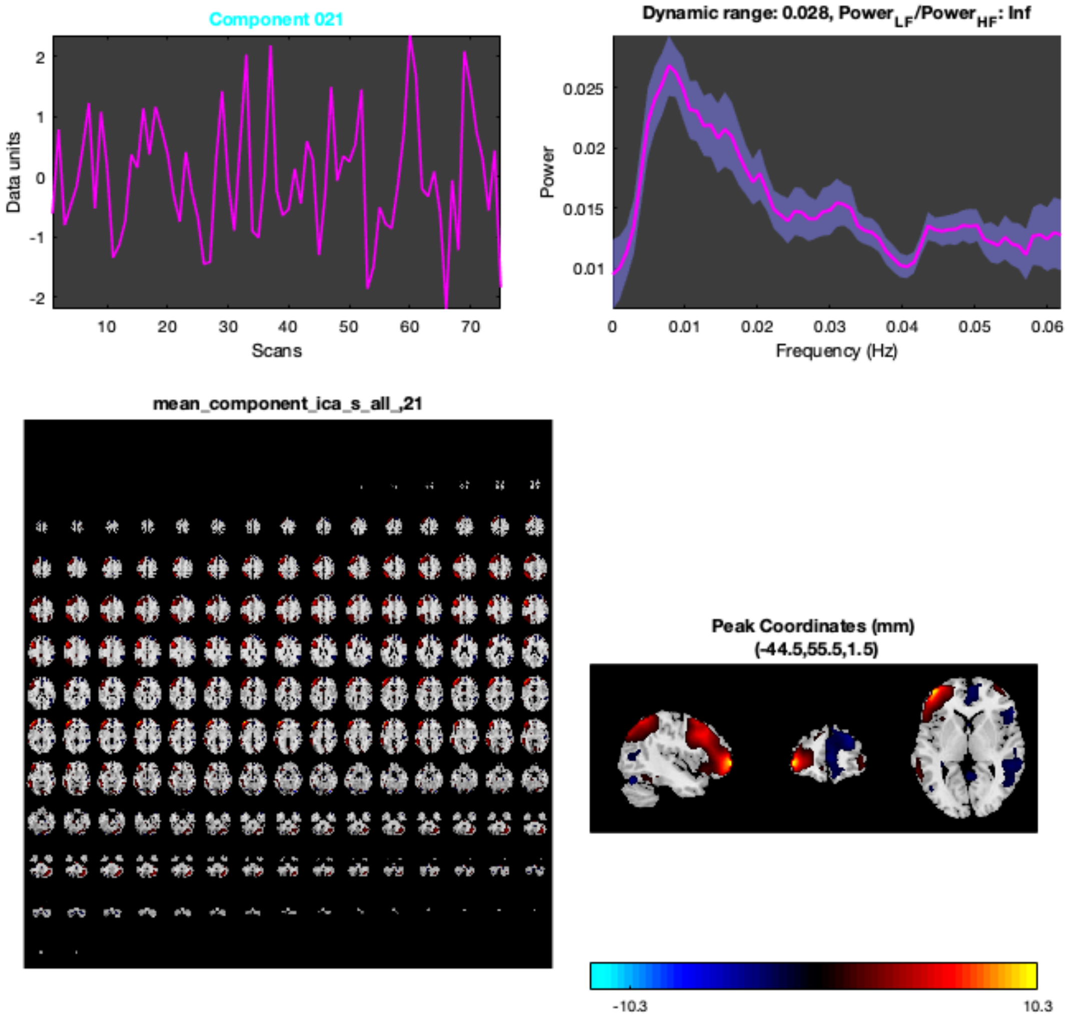
Component 021
Data units
Scans
Dynamic range: 0.028, Power_LF/Power_HF: Inf
Power
Frequency (Hz)
mean_component_ica_s_all_,21
Peak Coordinates (mm)
(-44.5,55.5,1.5)
-10.3
10.3

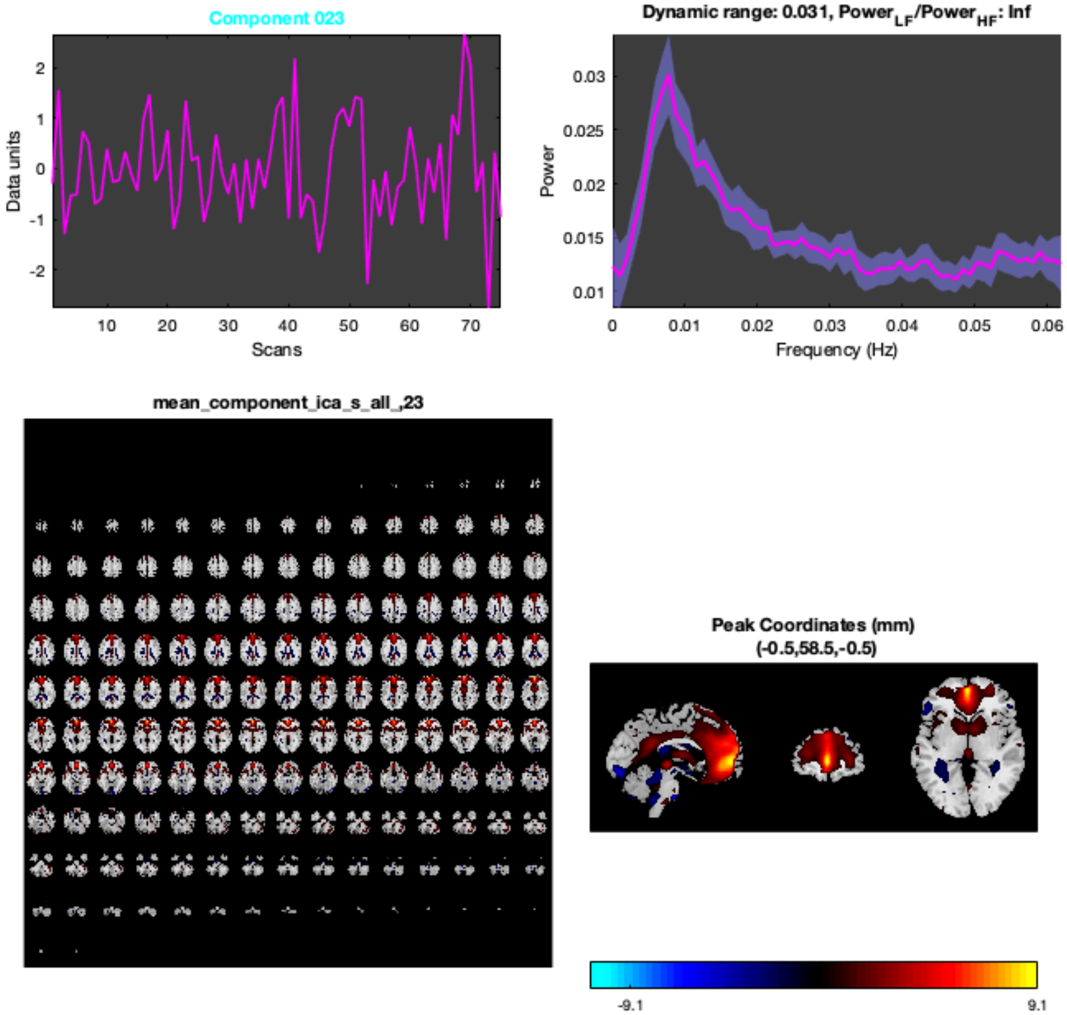
Component 023
Data units
Scans
Dynamic range: 0.031, Power_LF/Power_HF: Inf
Power
Frequency (Hz)
mean_component_ica_s_all_,23
Peak Coordinates (mm)
(-0.5,58.5,-0.5)
-9.1
9.1

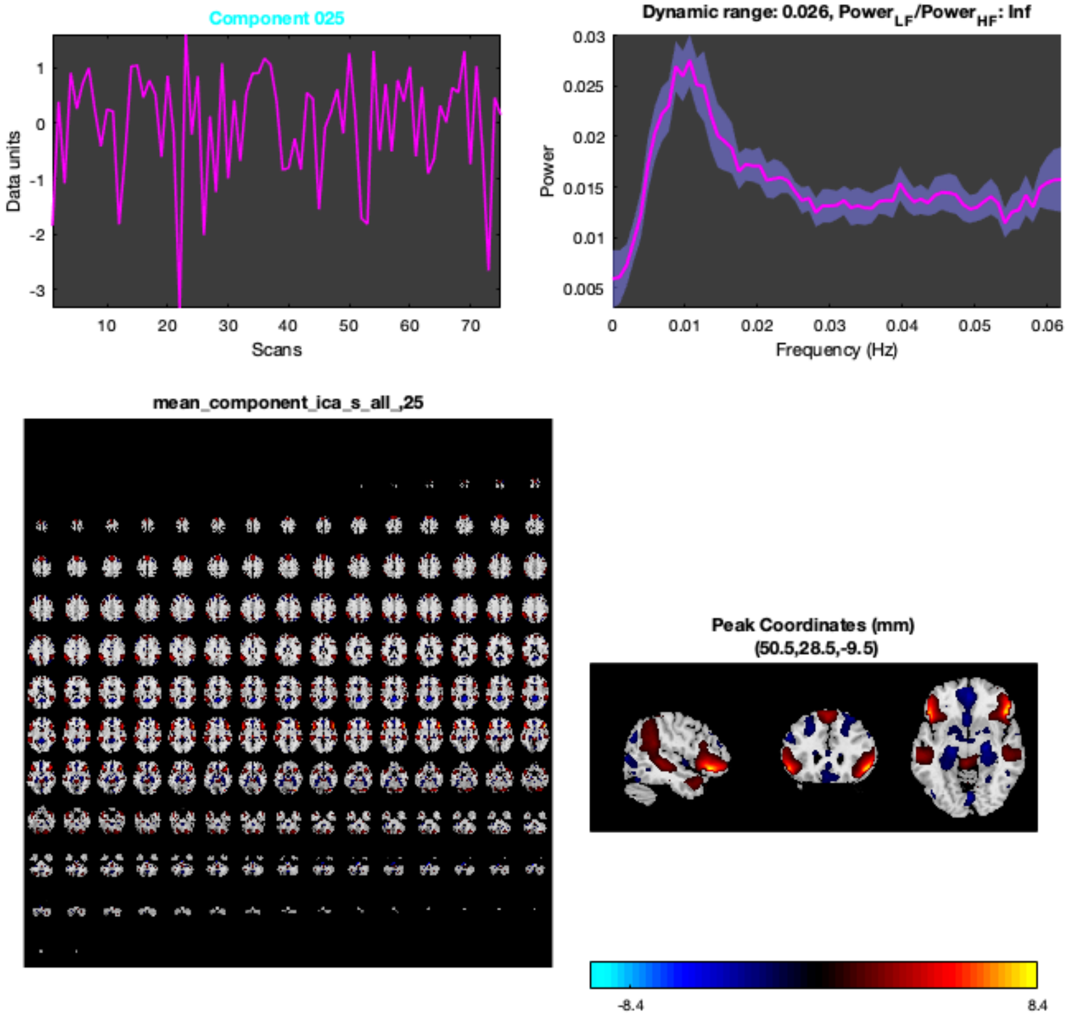

Component 025
Data units
Scans
Dynamic range: 0.026, Power$_{LF}$/Power$_{HF}$: Inf
Power
Frequency (Hz)
mean_component_ica_s_all_,25
Peak Coordinates (mm)
(50.5,28.5,-9.5)
-8.4
8.4

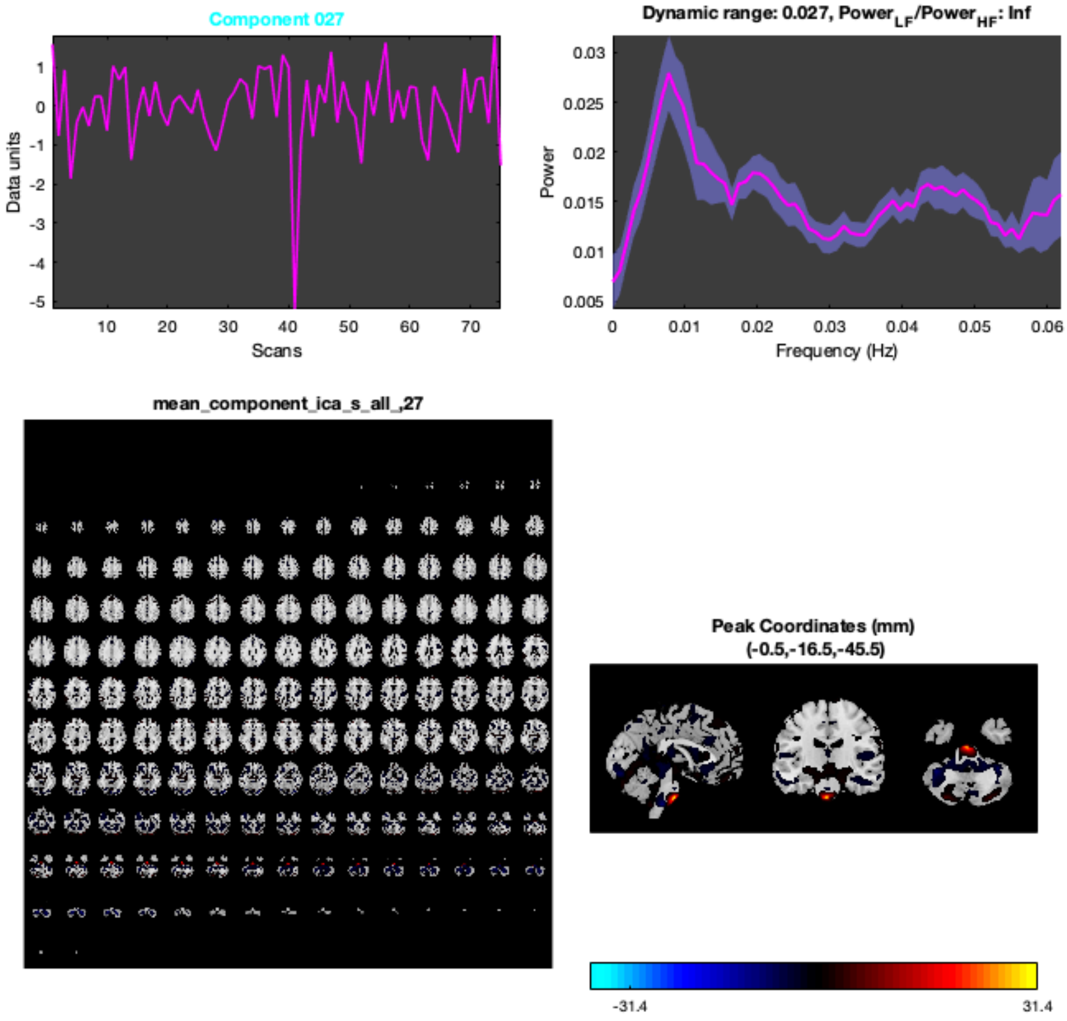
Component 027
Data units
Scans
Dynamic range: 0.027, Power$_{LF}$/Power$_{HF}$: Inf
Power
Frequency (Hz)
mean_component_ica_s_all_,27
Peak Coordinates (mm)
(-0.5,-16.5,-45.5)
-31.4
31.4

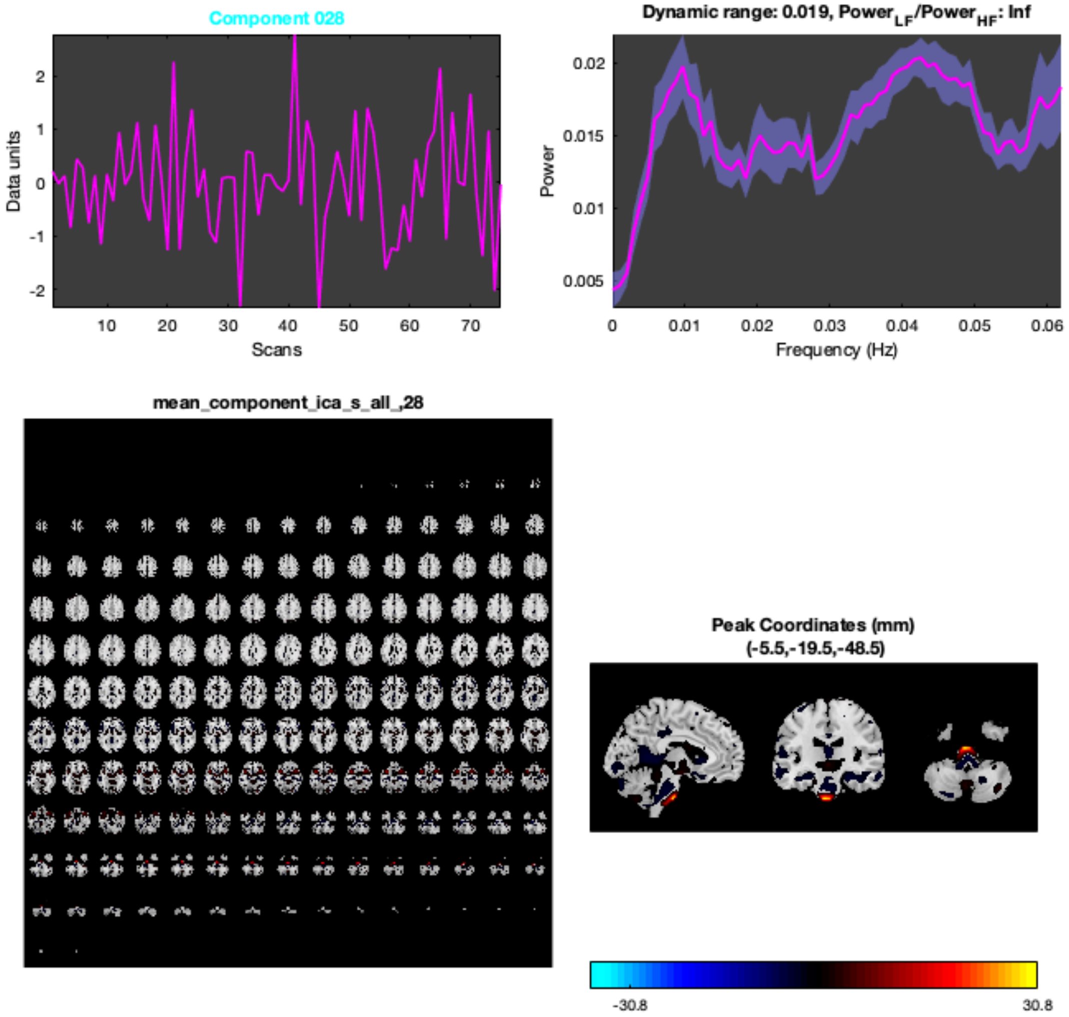
Component 028
Data units
Scans
Dynamic range: 0.019, Power$_{LF}$/Power$_{HF}$: Inf
Power
Frequency (Hz)
mean_component_ica_s_all_,28
Peak Coordinates (mm)
(-5.5,-19.5,-48.5)
-30.8
30.8

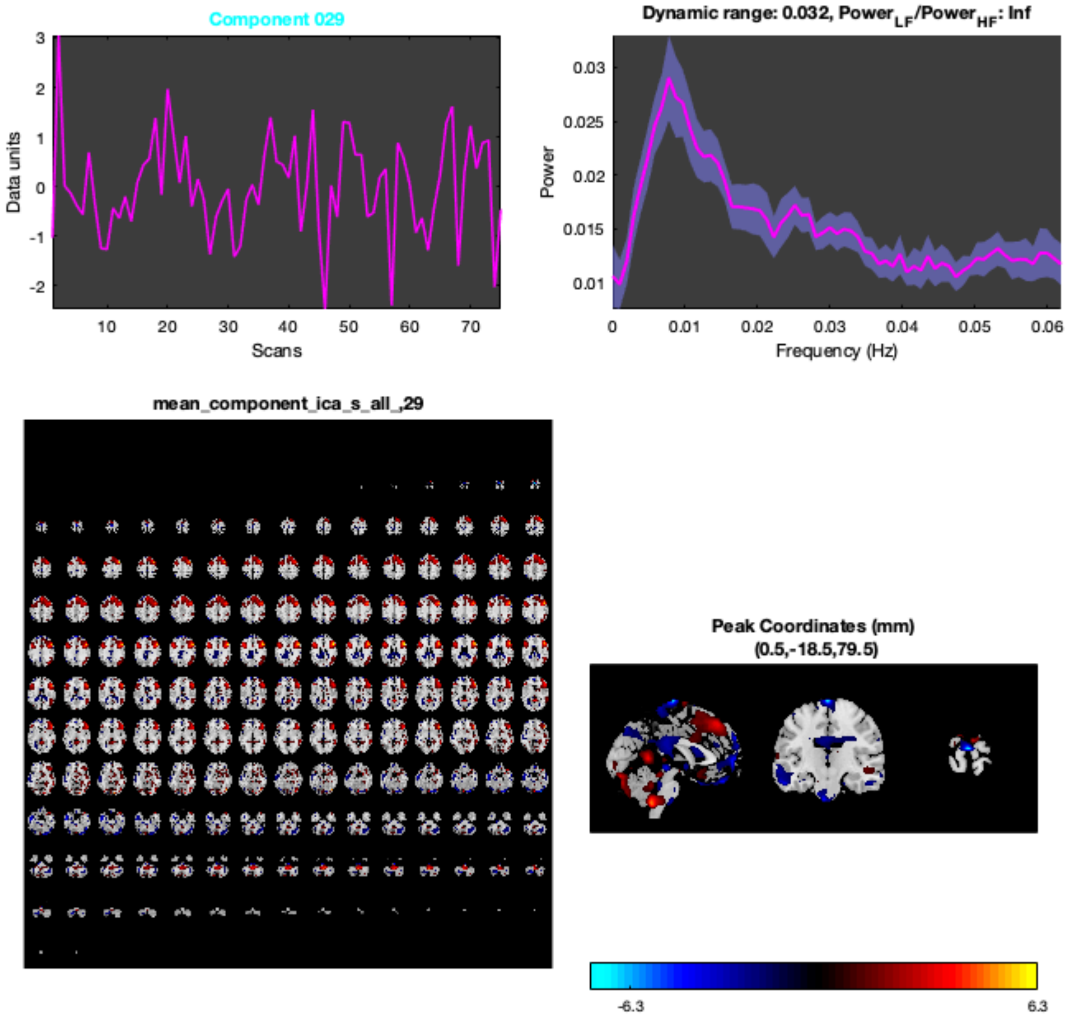

Spectral Summary

- **a) dynamic_range** - Difference between the peak power and minimum power at frequencies to the right of the peak.
- **b) fALFF** - Low frequency to high frequency power ratio.

ComponentNumber	*DynamicRange*	*fALFF*
1	*0.022208*	*Inf*
2	*0.023366*	*Inf*
3	*0.027416*	*Inf*
4	*0.017446*	*Inf*
5	*0.017061*	*Inf*
6	*0.023344*	*Inf*
7	*0.023867*	*Inf*
8	*0.03883*	*Inf*
