# Supplementary material for: Neural Correlates of Vocal Pitch Compensation in Individuals Who Stutter
Source: Front Hum Neurosci. 2020 Feb 25;14:18. doi: 10.3389/fnhum.2020.00018 (PMC7053555; doi:10.3389/fnhum.2020.00018)
Supplement: Supplementary file 5 [file Data_Sheet_4.PDF]

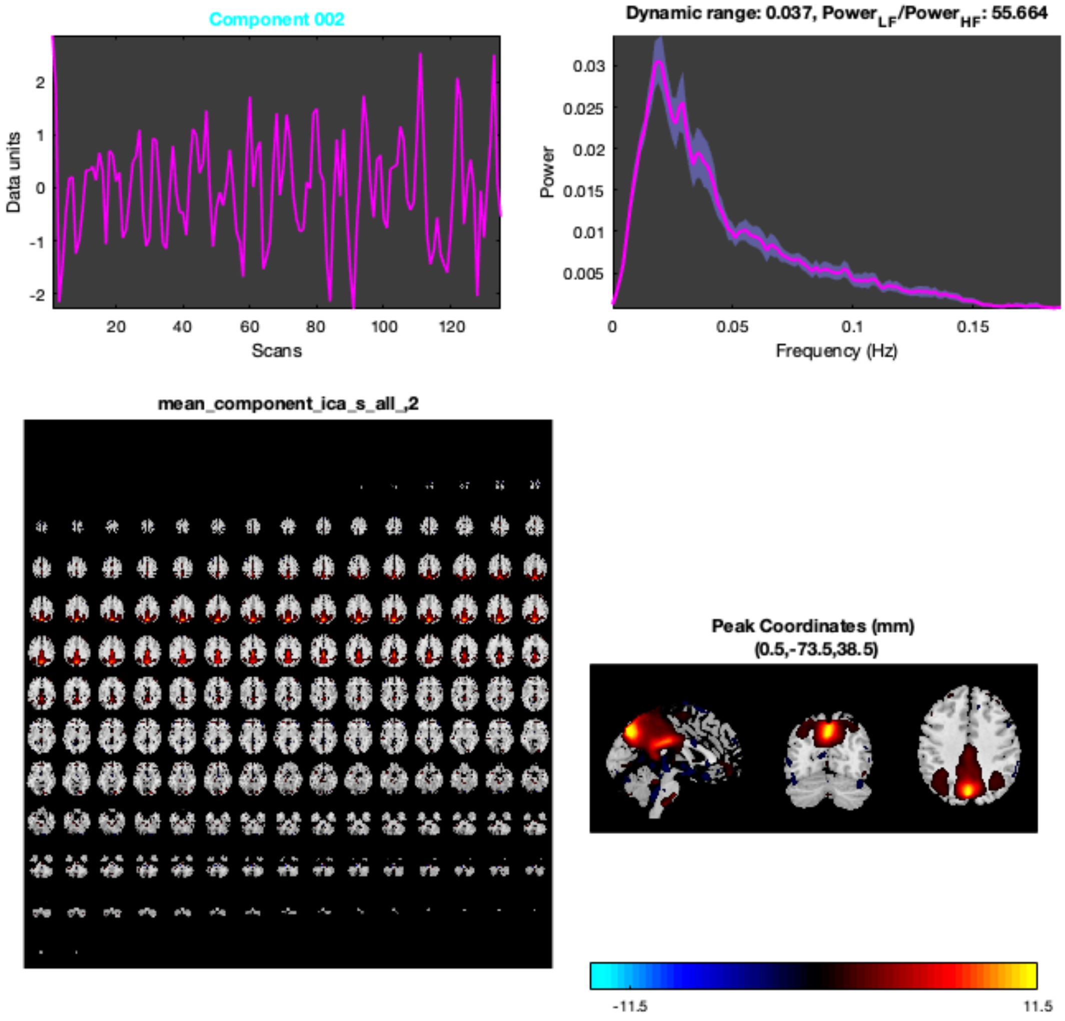
Component 002
Data units
Scans
Dynamic range: 0.037, Power_LF/Power_HF: 55.664
Power
Frequency (Hz)
mean_component_ica_s_all_,2
Peak Coordinates (mm)
(0.5,-73.5,38.5)
-11.5
11.5

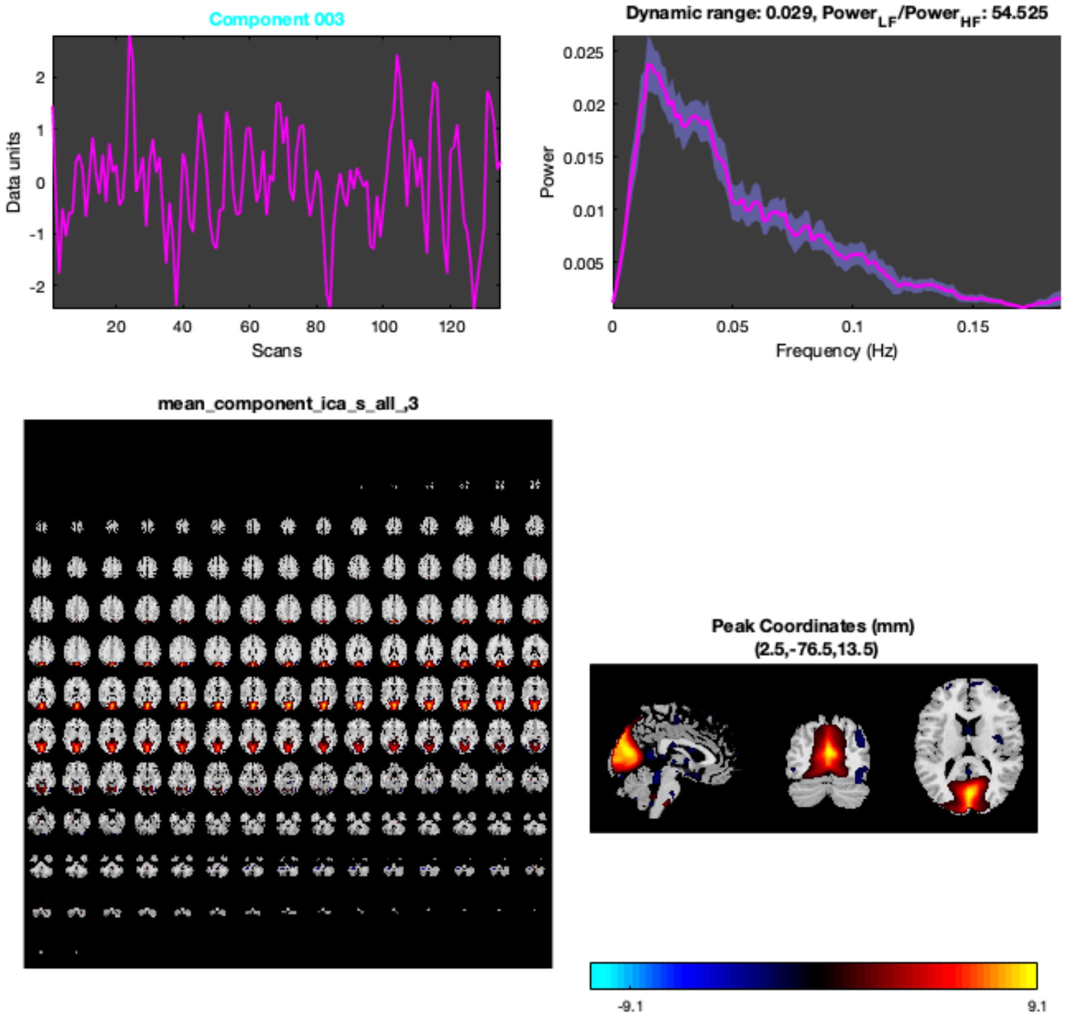
Component 003
Data units
Scans
Dynamic range: 0.029, Power_LF/Power_HF: 54.525
Power
Frequency (Hz)
mean_component_ica_s_all_,3
Peak Coordinates (mm)
(2.5,-76.5,13.5)
-9.1
9.1

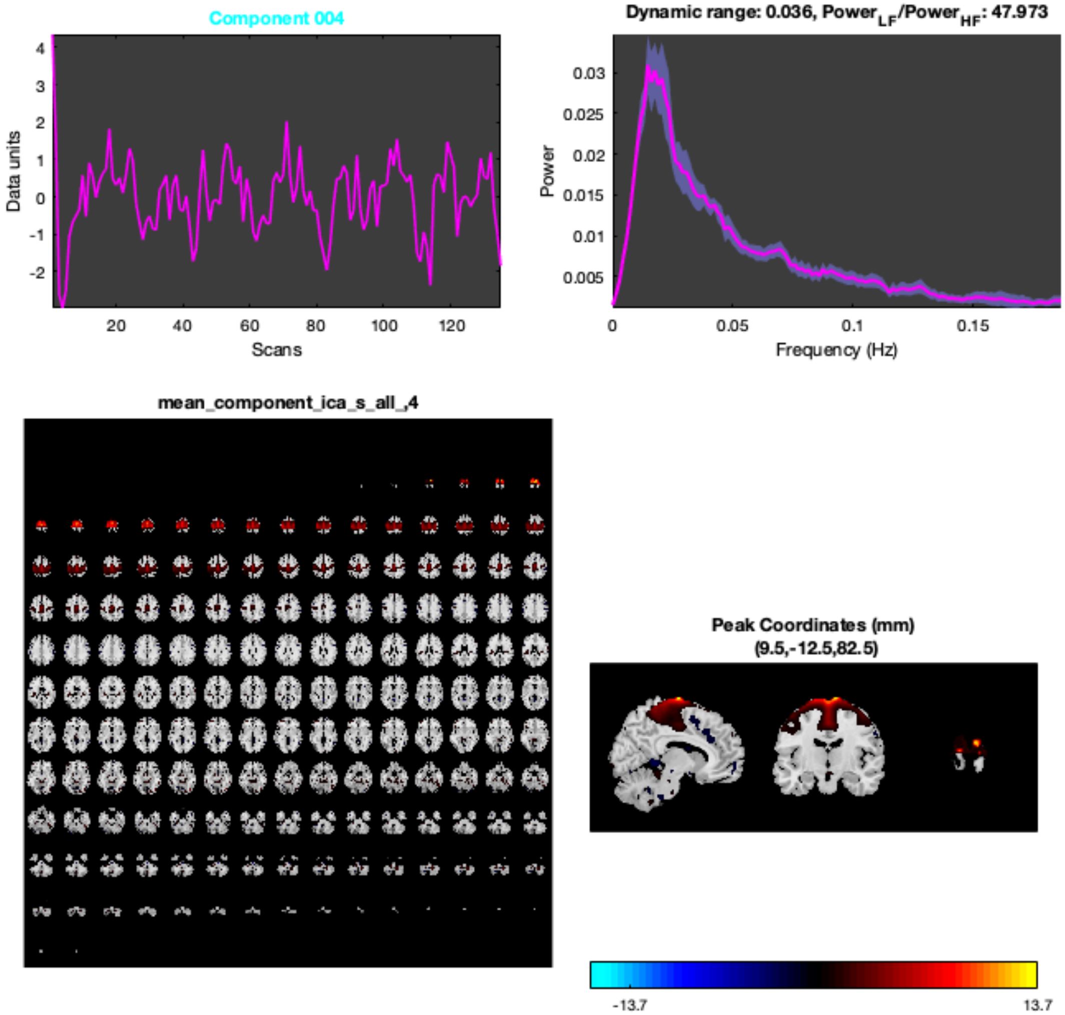
Component 004
Data units
Scans
Dynamic range: 0.036, Power$_{LF}$/Power$_{HF}$: 47.973
Power
Frequency (Hz)
mean_component_ica_s_all_,4
Peak Coordinates (mm)
(9.5,-12.5,82.5)
-13.7
13.7

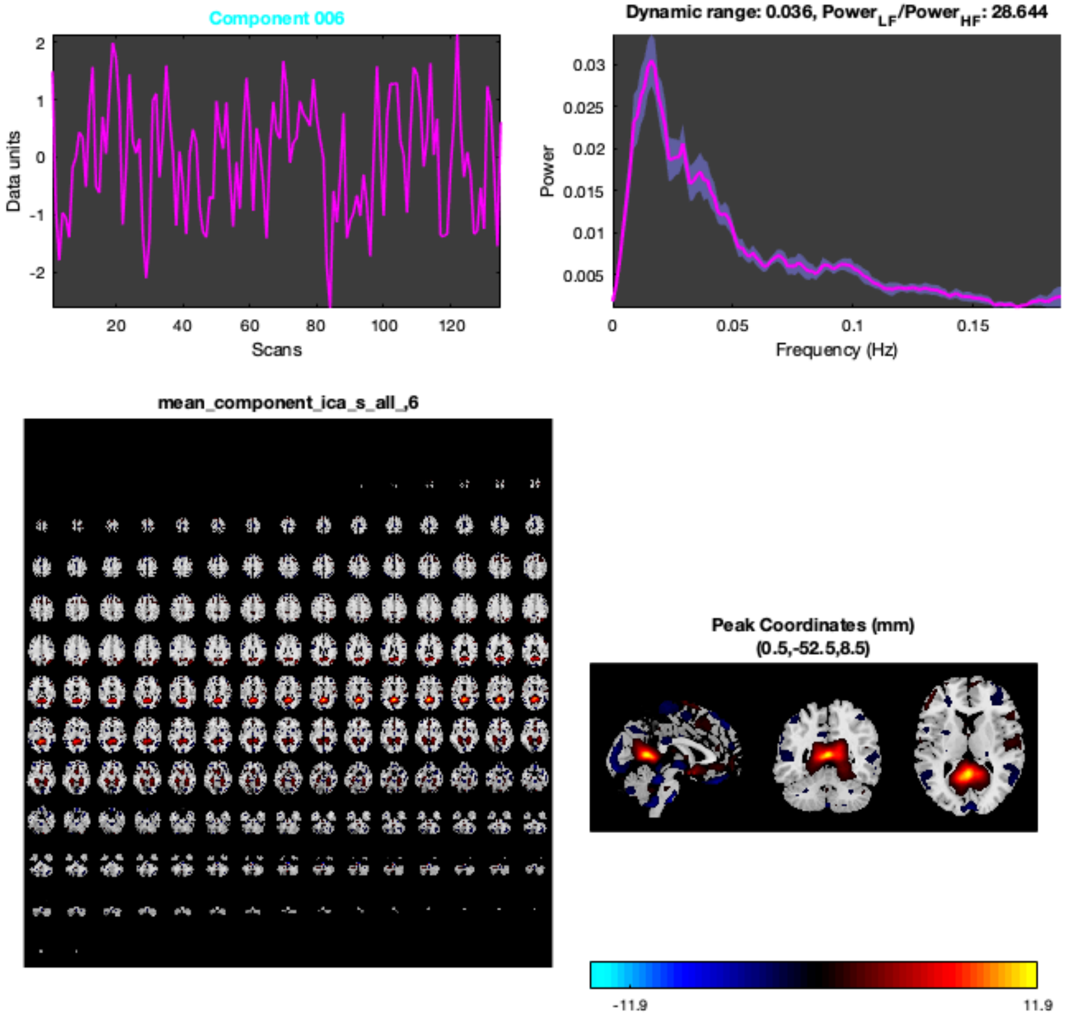

Component 006
Data units
Scans
Dynamic range: 0.036, Power_LF/Power_HF: 28.644
Power
Frequency (Hz)
mean_component_ica_s_all_,6
Peak Coordinates (mm)
(0.5,-52.5,8.5)
-11.9
11.9

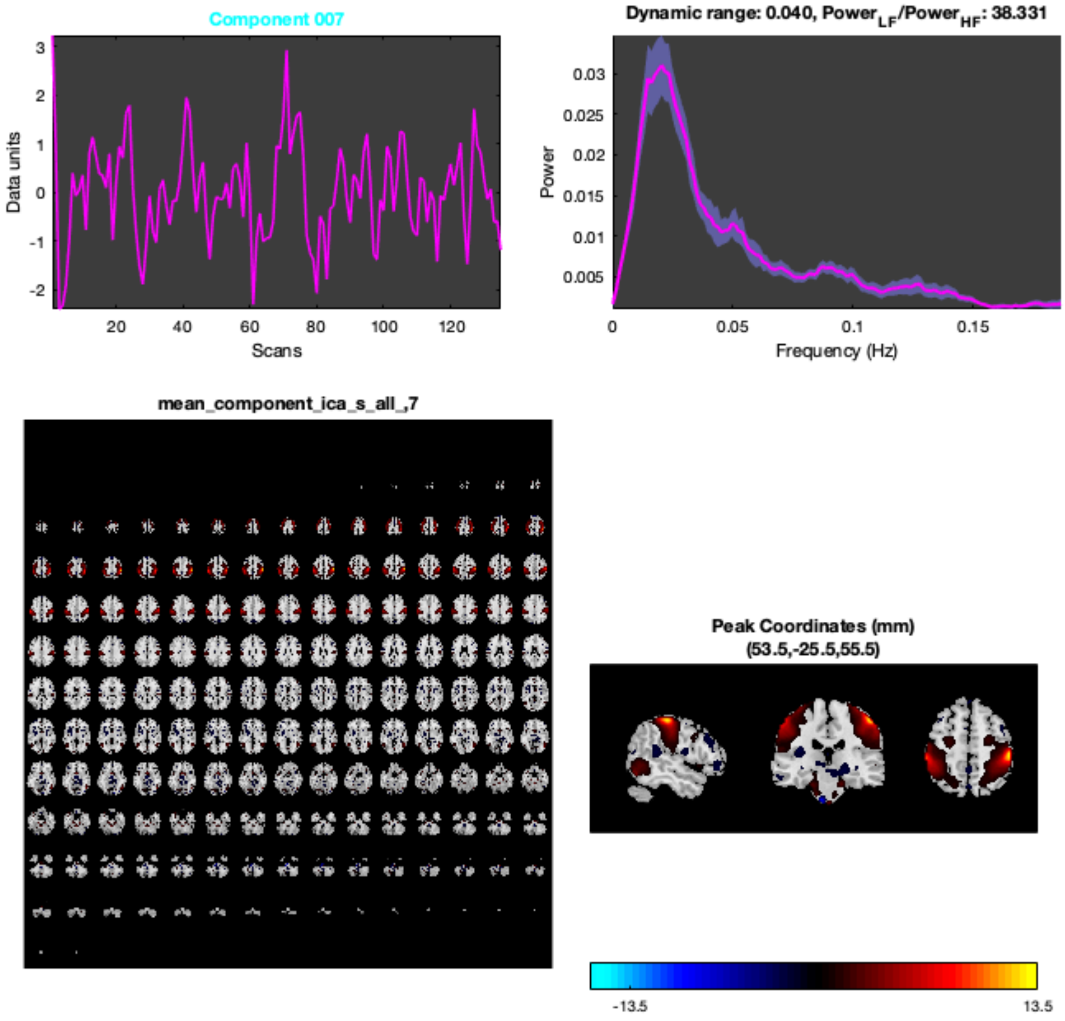

Component 007
Data units
Scans
Dynamic range: 0.040, Power$_{LF}$/Power$_{HF}$: 38.331
Power
Frequency (Hz)
mean_component_ica_s_all_,7
Peak Coordinates (mm)
(53.5,-25.5,55.5)
-13.5
13.5

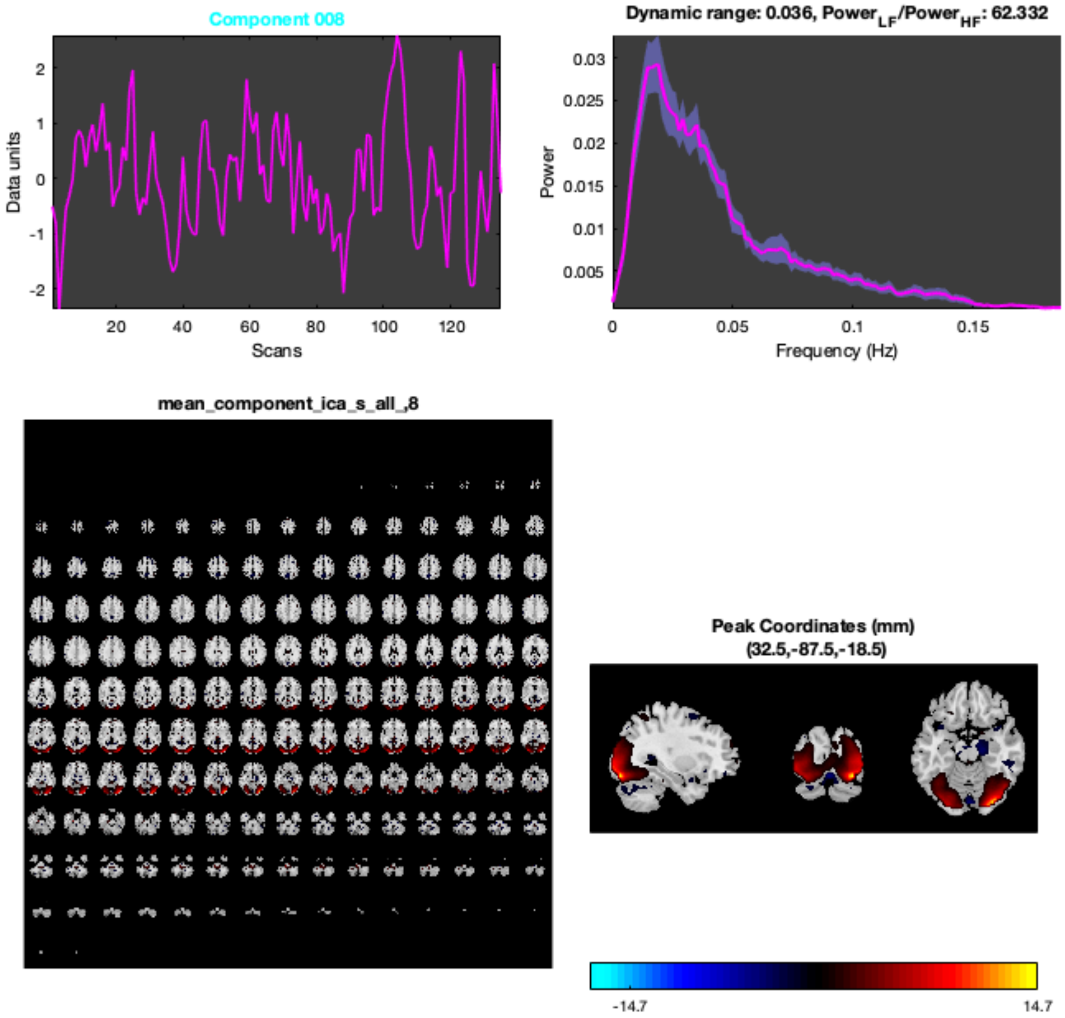
Component 008
Data units
Scans
Dynamic range: 0.036, Power$_{LF}$/Power$_{HF}$: 62.332
Power
Frequency (Hz)
mean_component_ica_s_all_,8
Peak Coordinates (mm)
(32.5,-87.5,-18.5)
-14.7
14.7

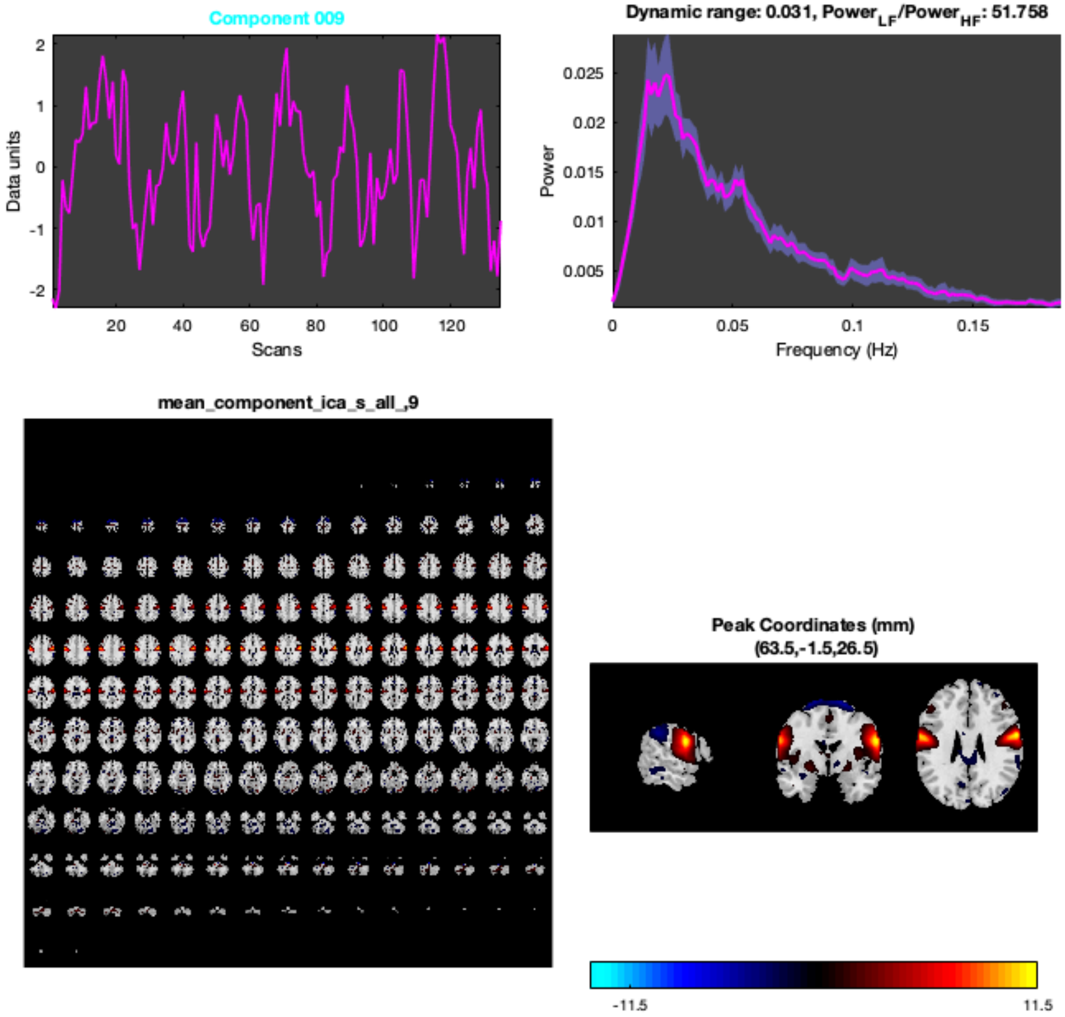
Component 009
Data units
Scans
Dynamic range: 0.031, Power_LF/Power_HF: 51.758
Power
Frequency (Hz)
mean_component_ica_s_all_,9
Peak Coordinates (mm)
(63.5,-1.5,26.5)
-11.5
11.5

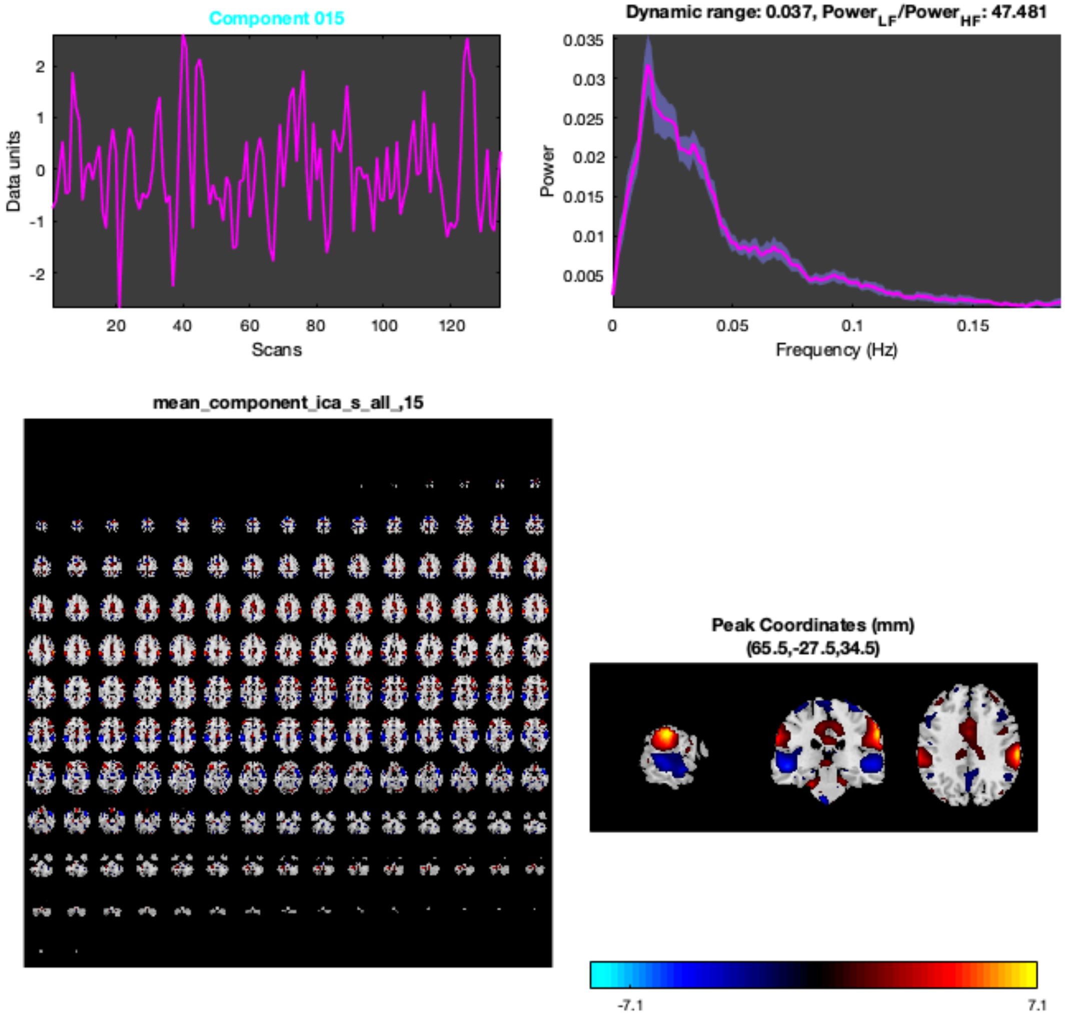
Component 015
Data units
Scans
Dynamic range: 0.037, Power$_{LF}$/Power$_{HF}$: 47.481
Power
Frequency (Hz)
mean_component_ica_s_all_,15
Peak Coordinates (mm)
(65.5,-27.5,34.5)
-7.1
7.1

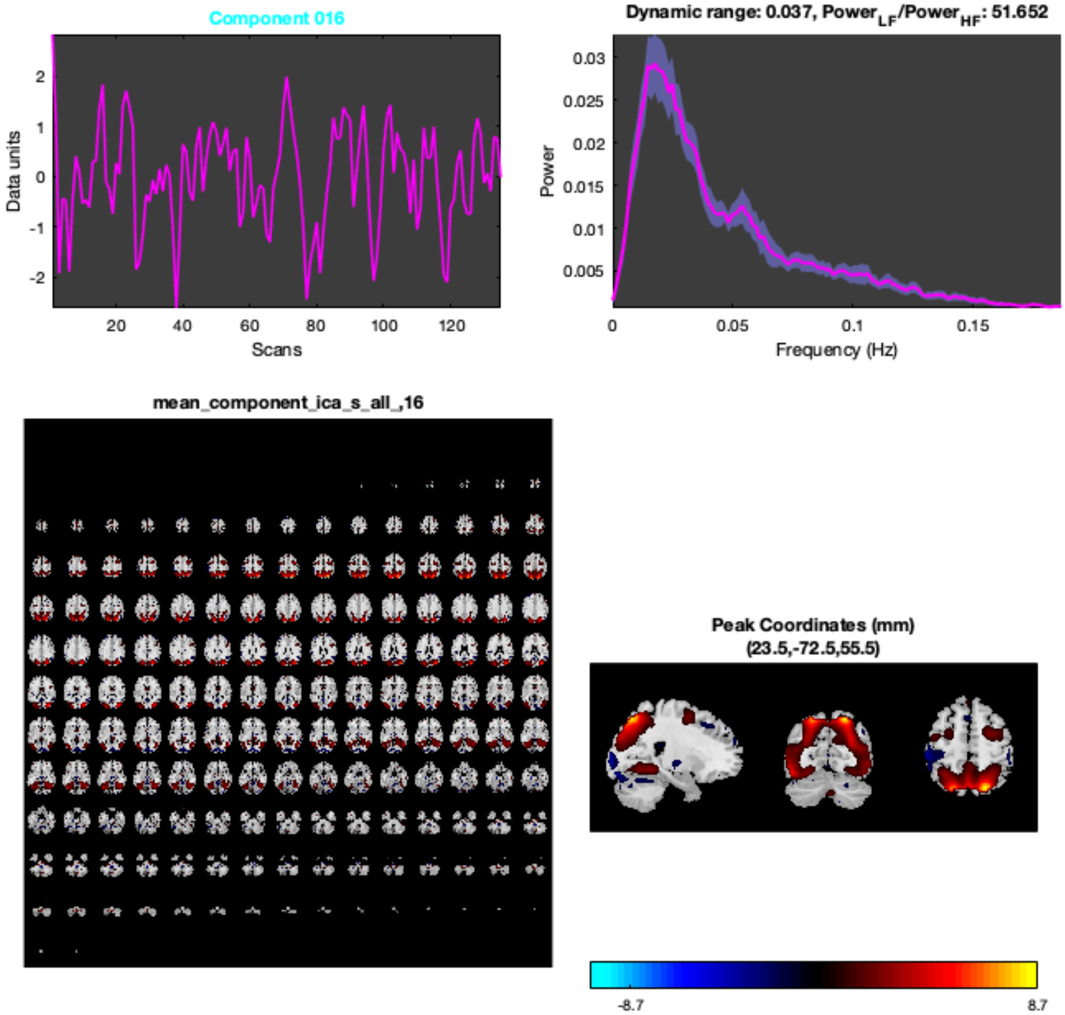
Component 016
Data units
Scans
Dynamic range: 0.037, Power$_{LF}$/Power$_{HF}$: 51.652
Power
Frequency (Hz)
mean_component_ica_s_all_,16
Peak Coordinates (mm)
(23.5,-72.5,55.5)
-8.7
8.7

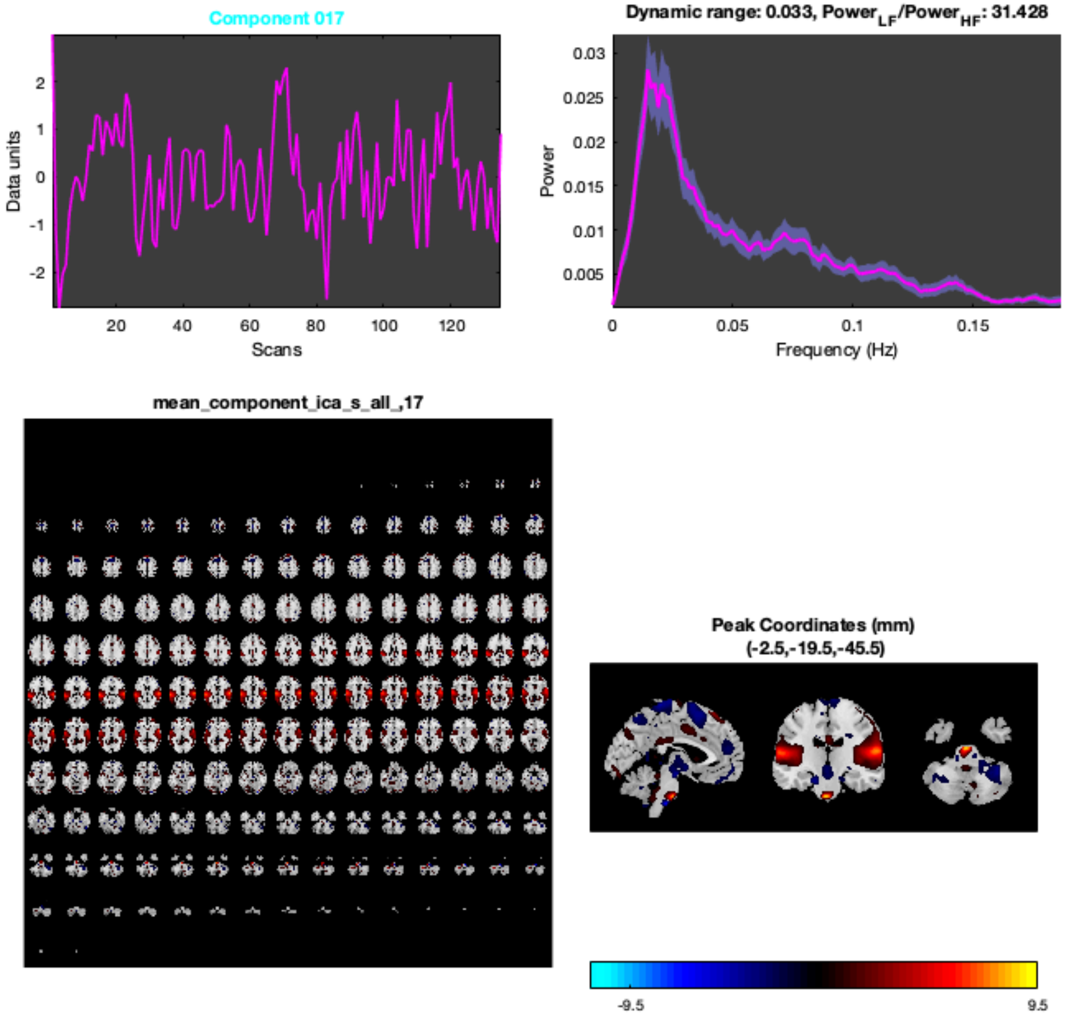
Component 017
Data units
Scans
Dynamic range: 0.033, Power$_{LF}$/Power$_{HF}$: 31.428
Power
Frequency (Hz)
mean_component_ica_s_all_,17
Peak Coordinates (mm)
(-2.5,-19.5,-45.5)
-9.5
9.5

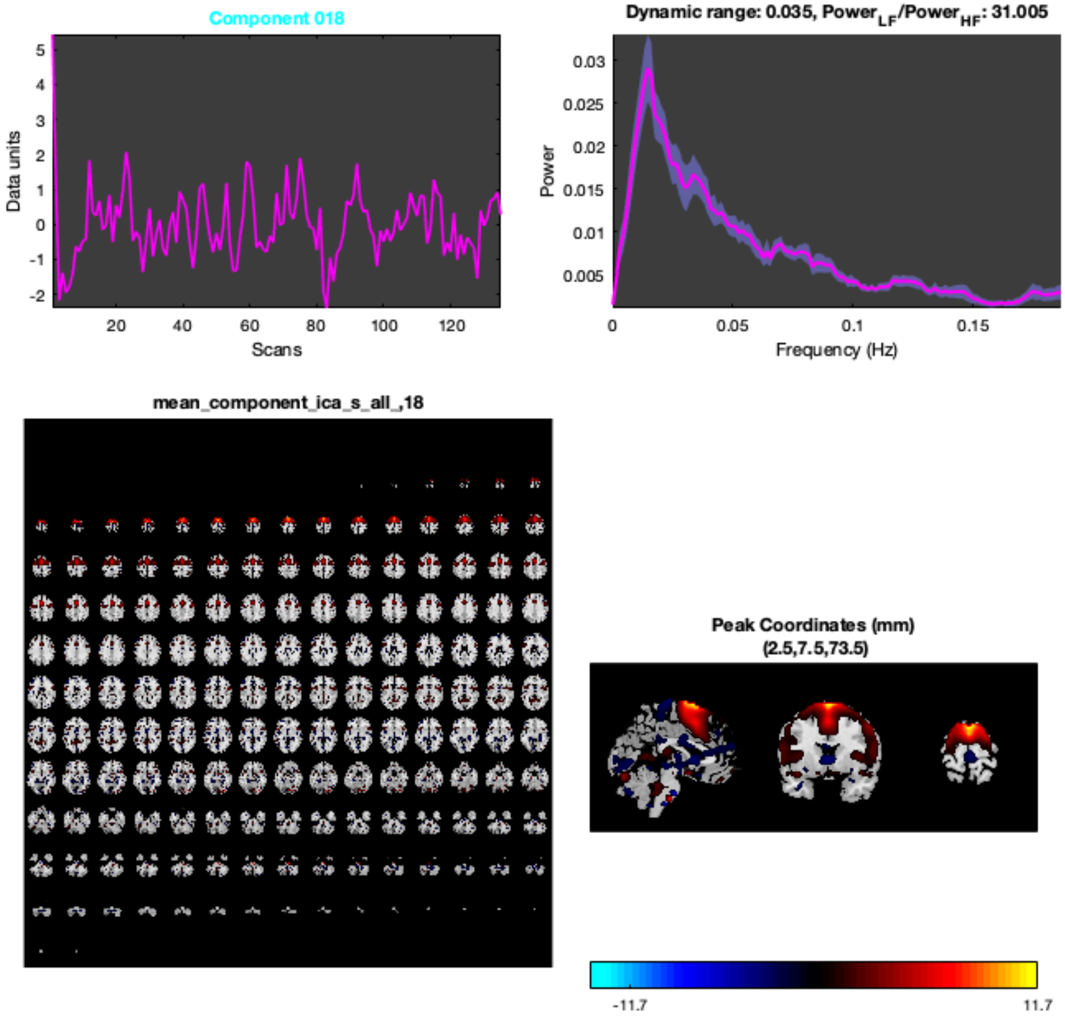
Component 018
Data units
Scans
Dynamic range: 0.035, Power_LF/Power_HF: 31.005
Power
Frequency (Hz)
mean_component_ica_s_all_,18
Peak Coordinates (mm)
(2.5,7.5,73.5)
-11.7
11.7

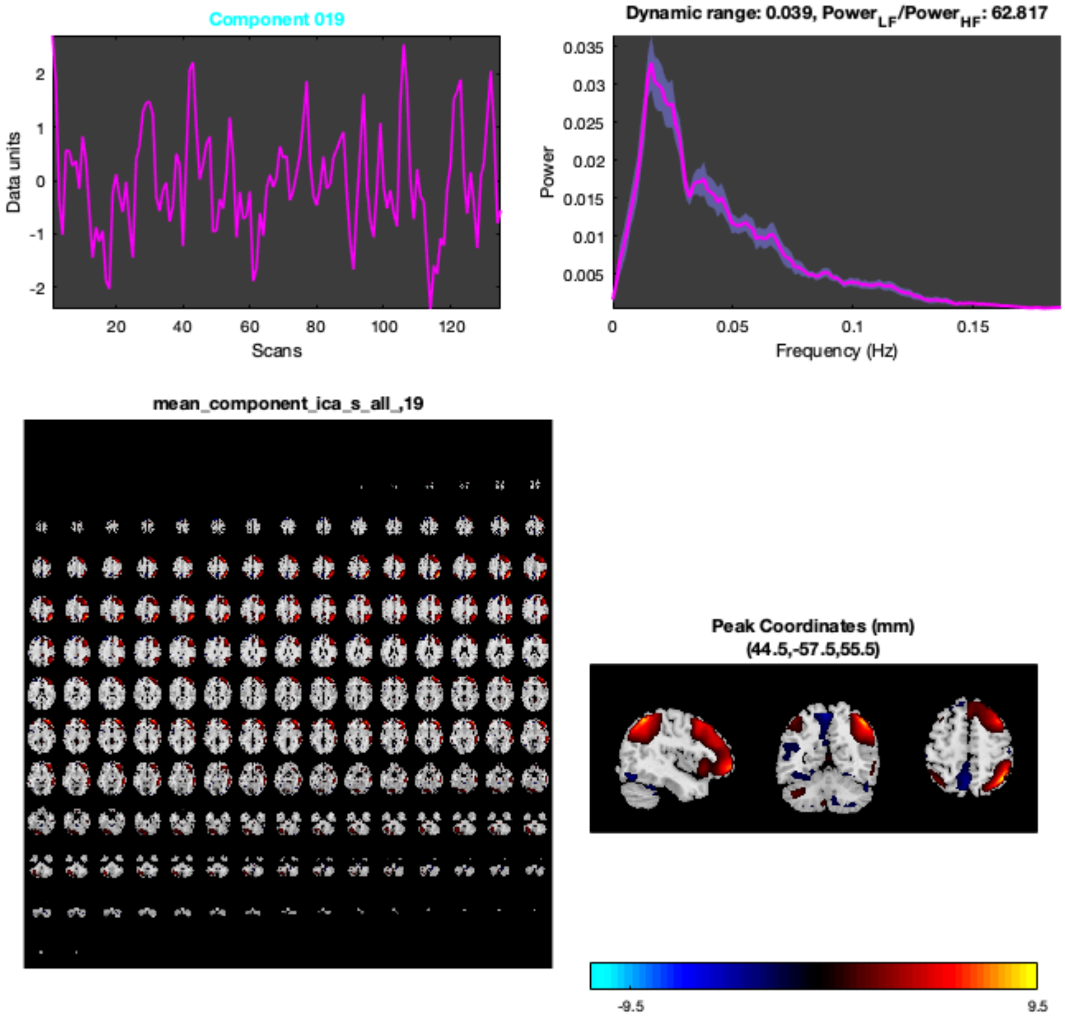
Component 019
Data units
Scans
Dynamic range: 0.039, Power$_{LF}$/Power$_{HF}$: 62.817
Power
Frequency (Hz)
mean_component_ica_s_all_,19
Peak Coordinates (mm)
(44.5,-57.5,55.5)
-9.5
9.5

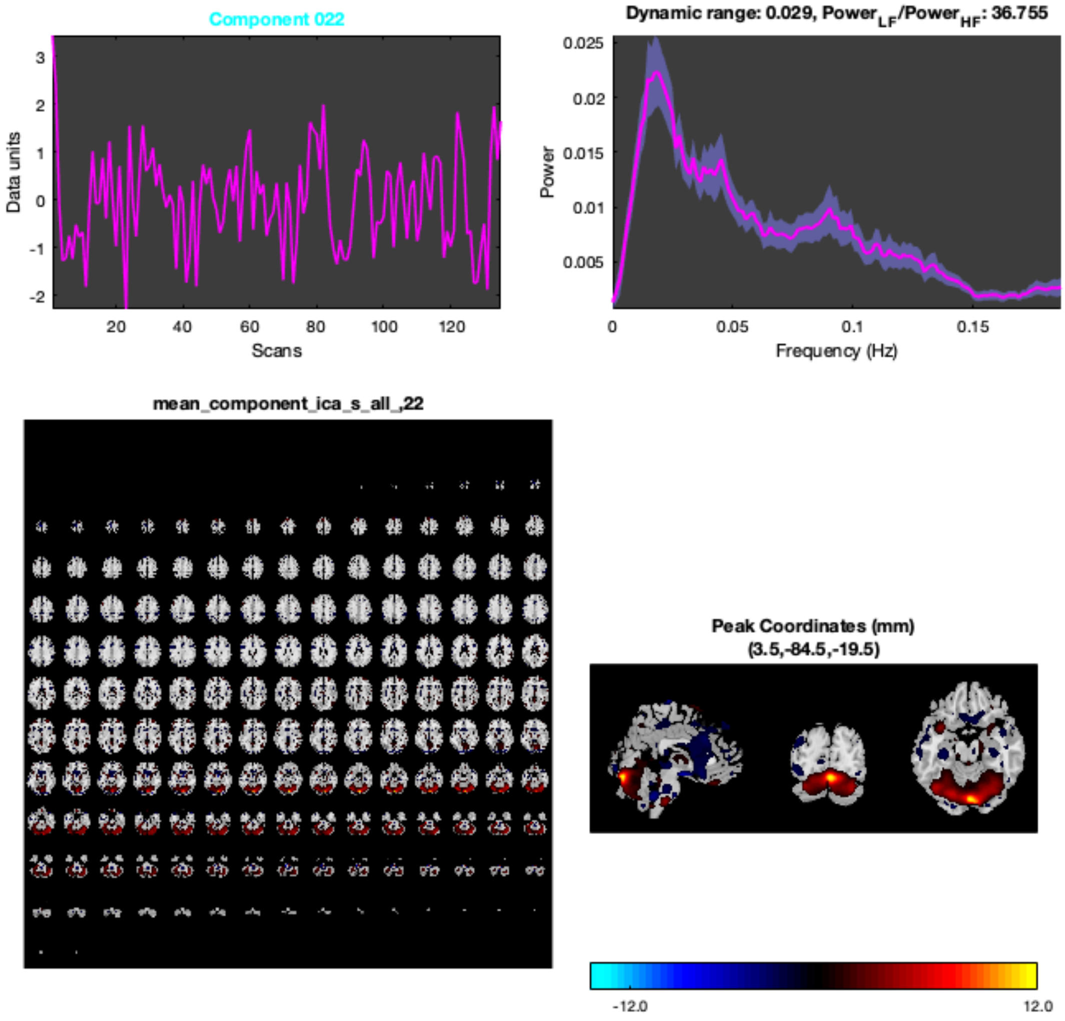
Component 022
Data units
Scans
Dynamic range: 0.029, Power$_{LF}$/Power$_{HF}$: 36.755
Power
Frequency (Hz)
mean_component_ica_s_all_,22
Peak Coordinates (mm)
(3.5,-84.5,-19.5)
-12.0
12.0

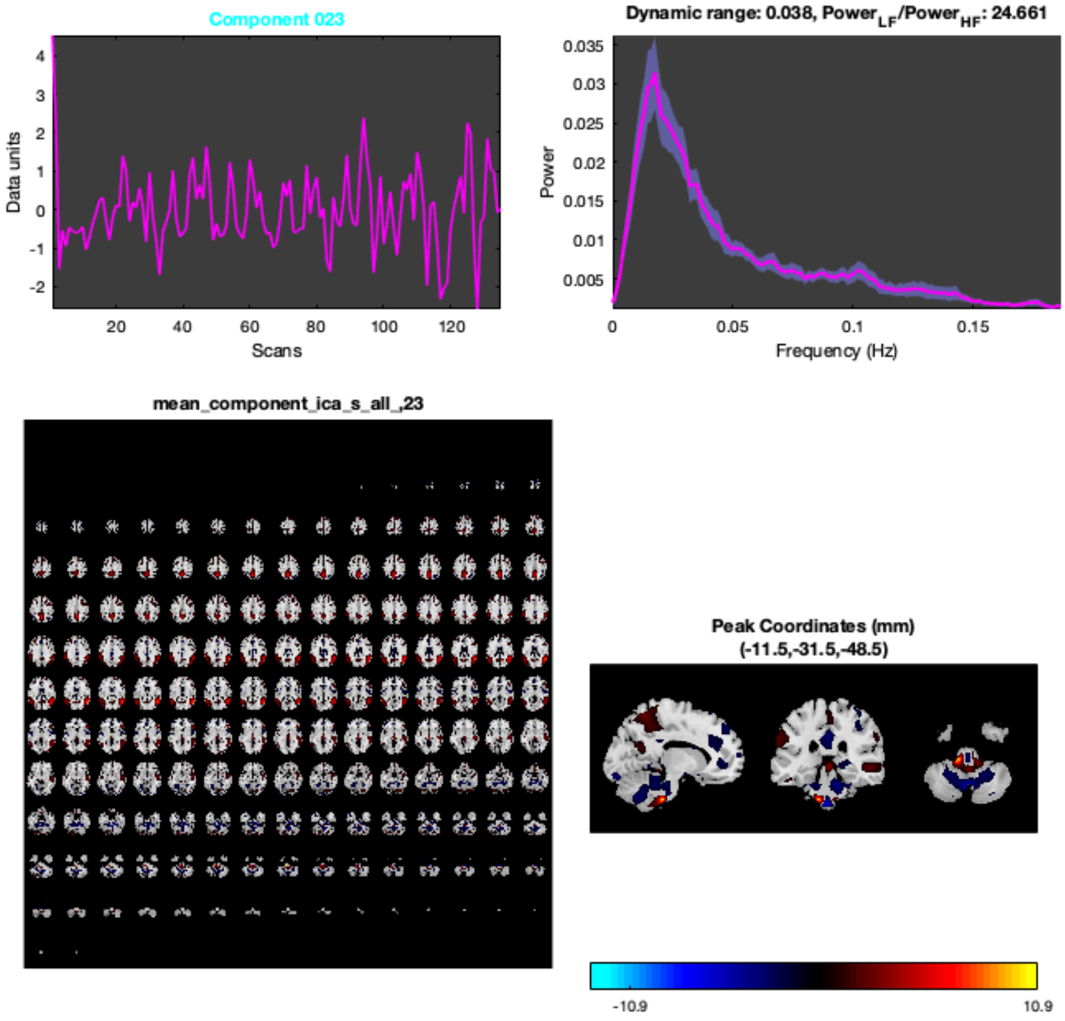
Component 023
Data units
Scans
Dynamic range: 0.038, Power_LF/Power_HF: 24.661
Power
Frequency (Hz)
mean_component_ica_s_all_,23
Peak Coordinates (mm)
(-11.5,-31.5,-48.5)
-10.9
10.9

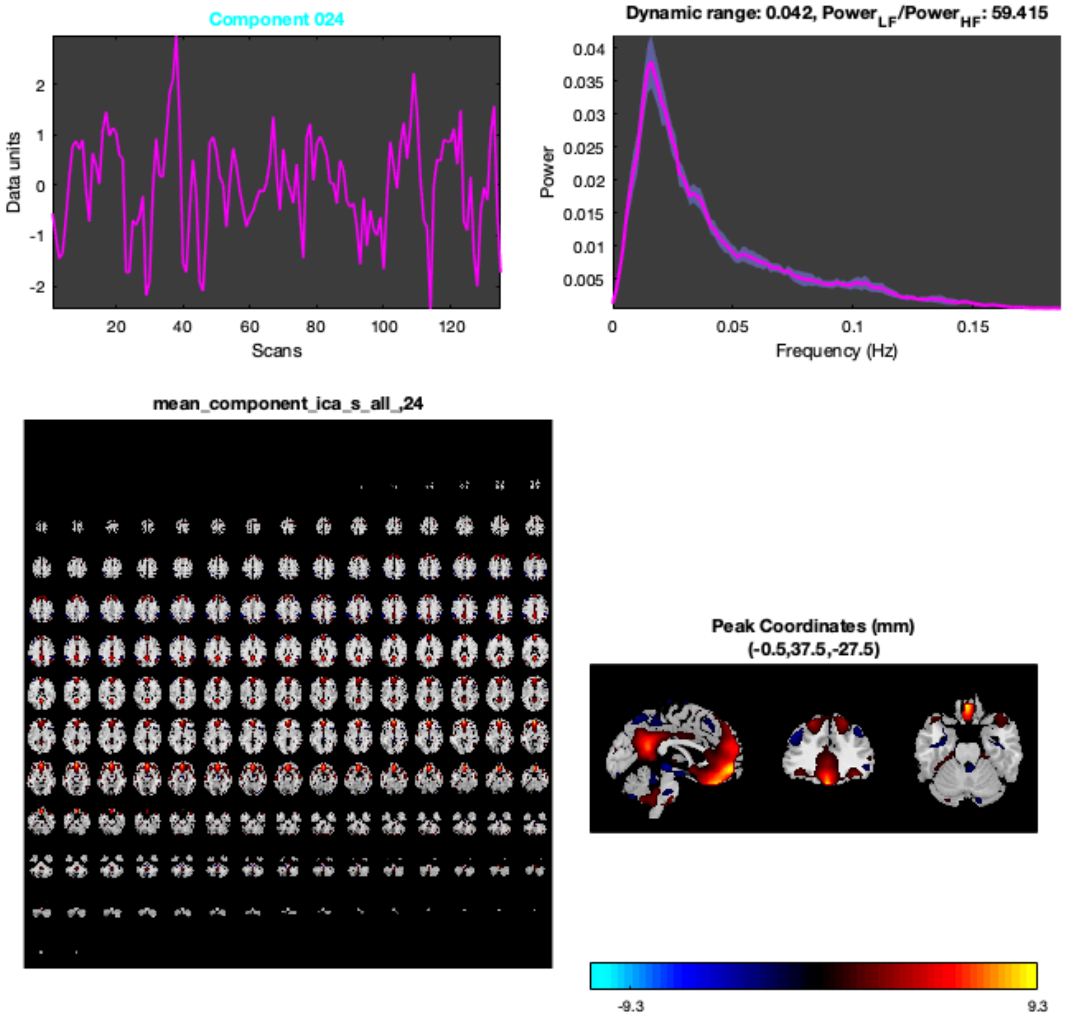
Component 024
Data units
Scans
Dynamic range: 0.042, Power$_{LF}$/Power$_{HF}$: 59.415
Power
Frequency (Hz)
mean_component_ica_s_all_,24
Peak Coordinates (mm)
(-0.5,37.5,-27.5)
-9.3
9.3

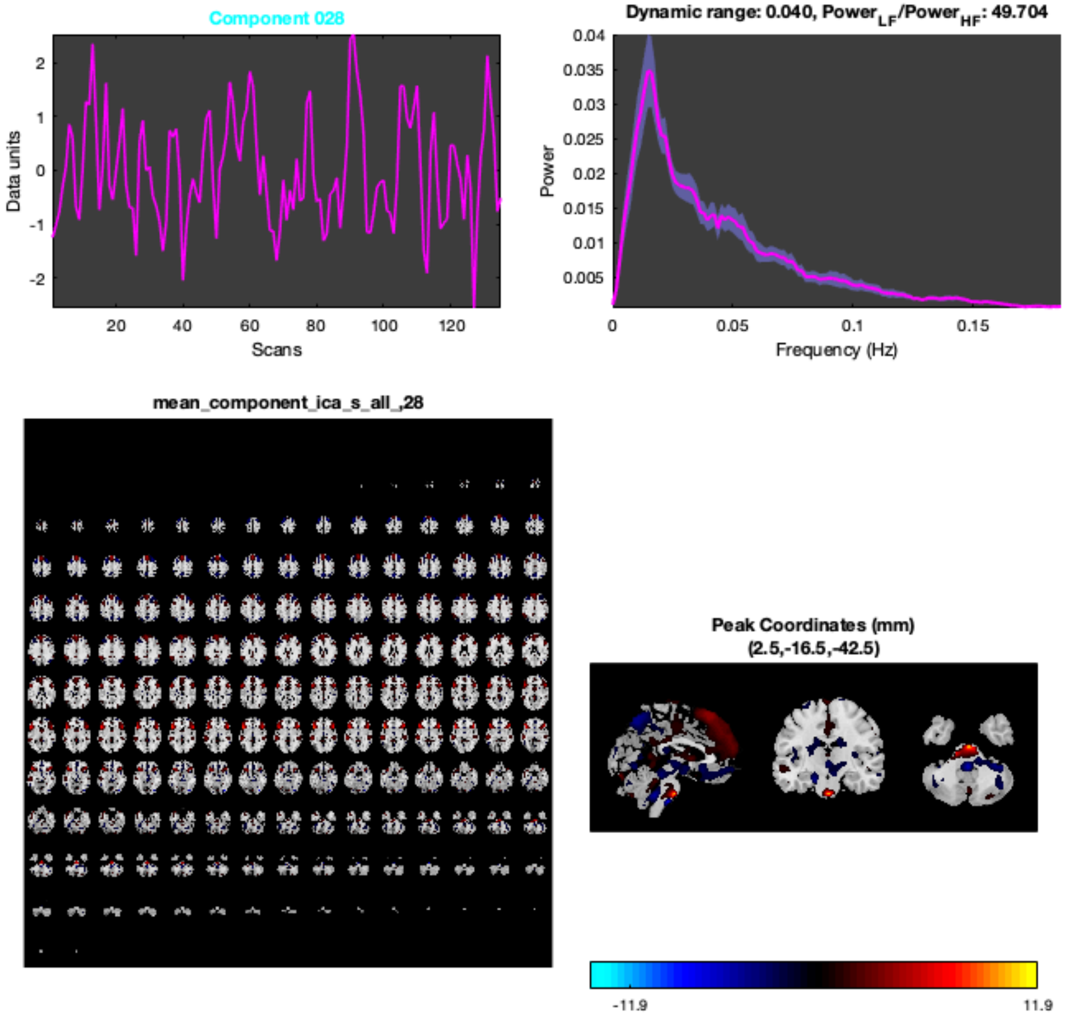
Component 028
Data units
Scans
Dynamic range: 0.040, Power_LF/Power_HF: 49.704
Power
Frequency (Hz)
mean_component_ica_s_all_,28
Peak Coordinates (mm)
(2.5,-16.5,-42.5)
-11.9
11.9

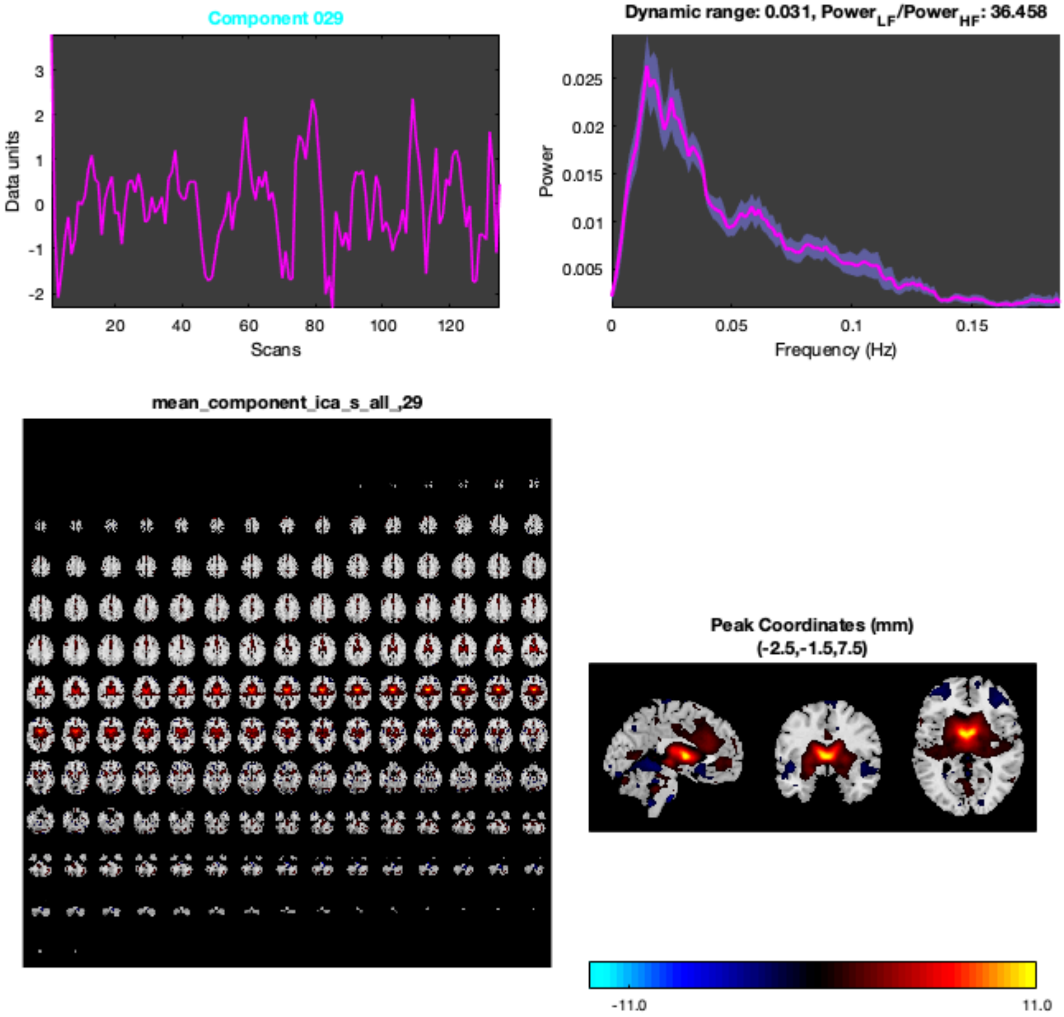
Component 029
Data units
Scans
Dynamic range: 0.031, Power_LF/Power_HF: 36.458
Power
Frequency (Hz)
mean_component_ica_s_all_,29
Peak Coordinates (mm)
(-2.5,-1.5,7.5)
-11.0
11.0

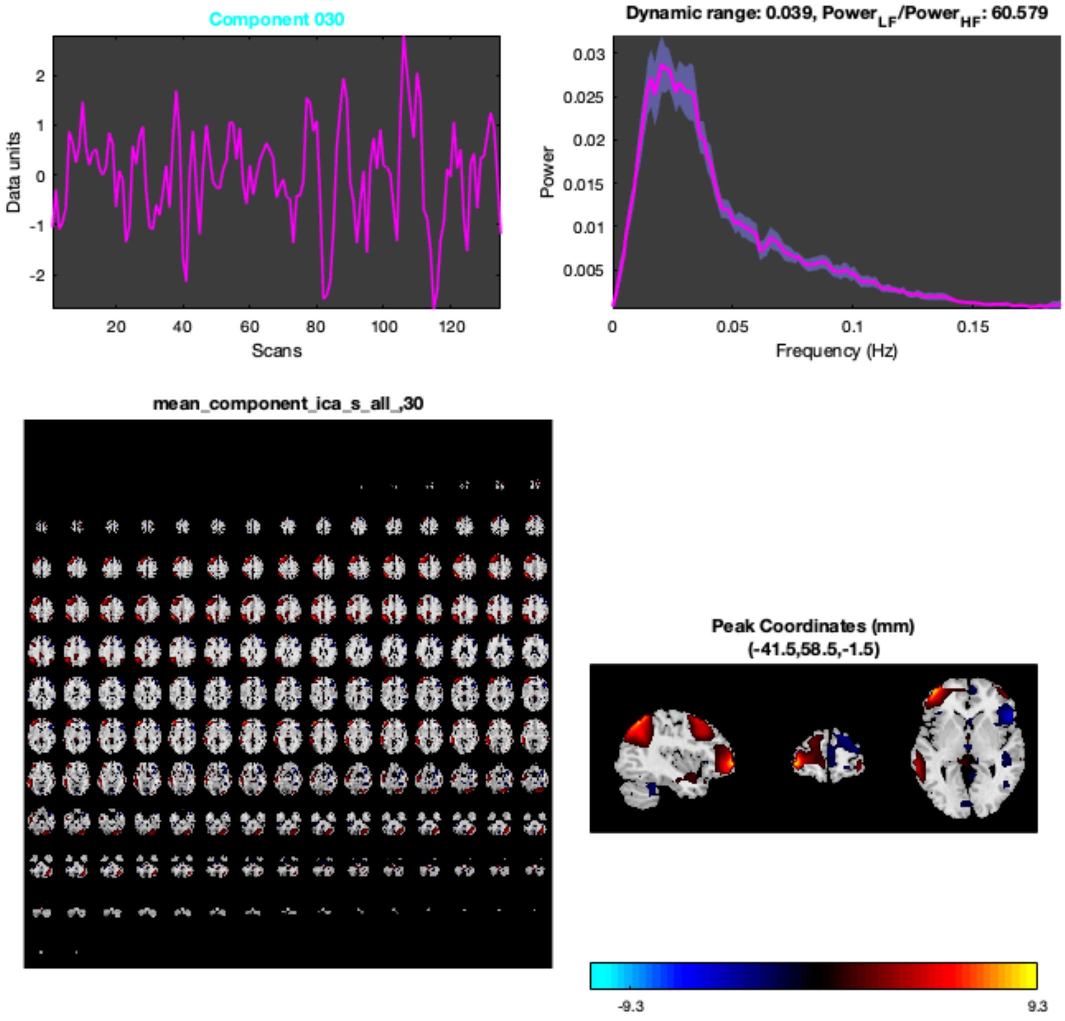

Component 030
Data units
Scans
Dynamic range: 0.039, Power$_{LF}$/Power$_{HF}$: 60.579
Power
Frequency (Hz)
mean_component_ica_s_all_,30
Peak Coordinates (mm)
(-41.5,58.5,-1.5)
-9.3
9.3

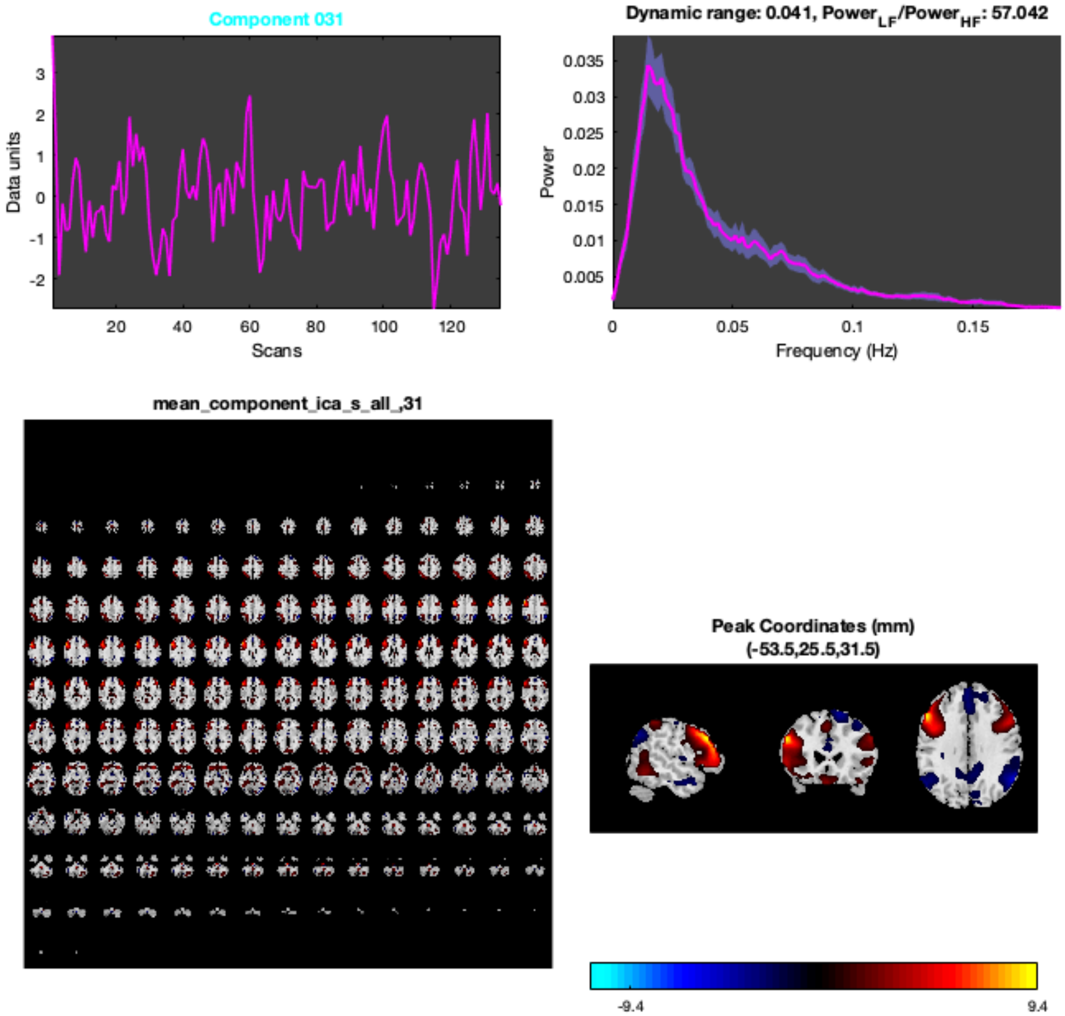
Component 031
Data units
Scans
Dynamic range: 0.041, Power$_{LF}$/Power$_{HF}$: 57.042
Power
Frequency (Hz)
mean_component_ica_s_all_,31
Peak Coordinates (mm)
(-53.5,25.5,31.5)
-9.4
9.4

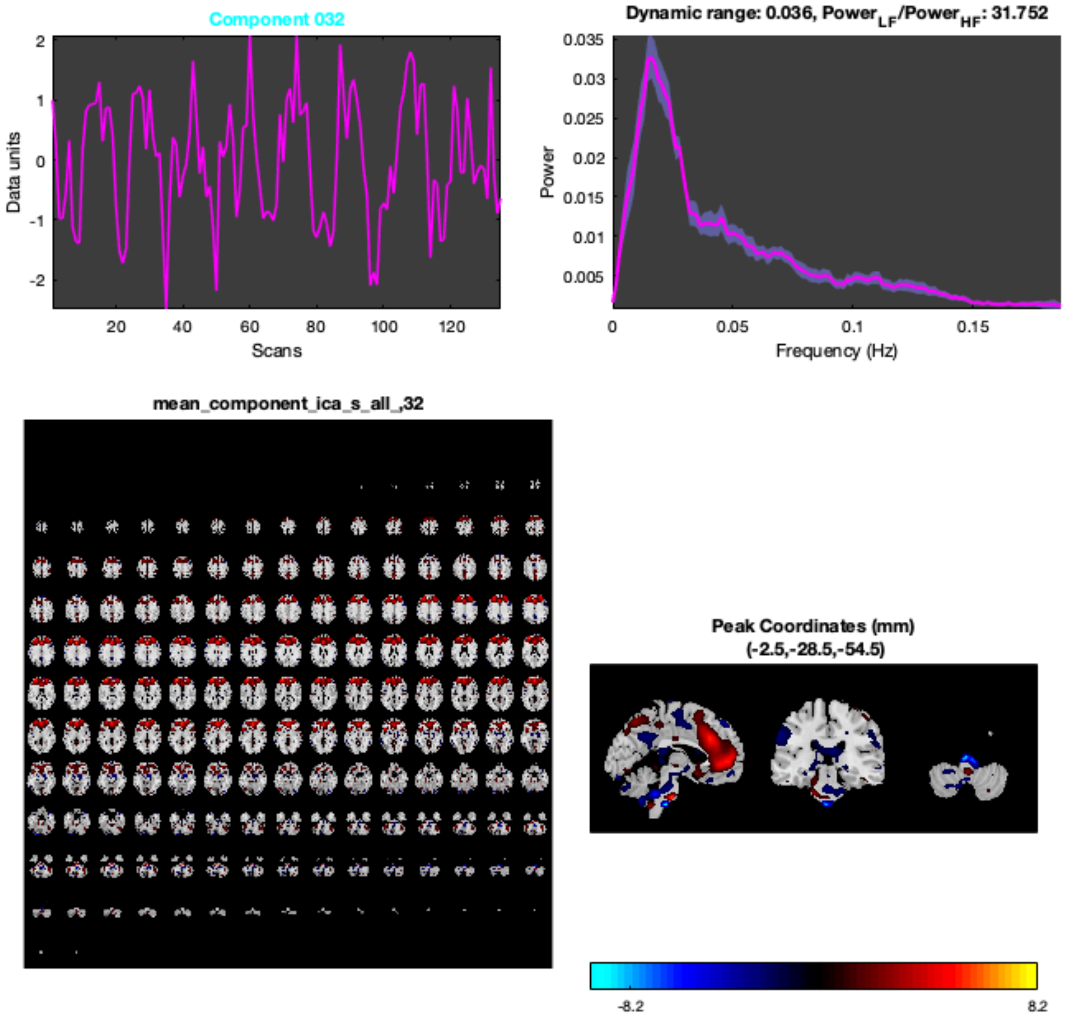
Component 032
Data units
Scans
Dynamic range: 0.036, Power$_{LF}$/Power$_{HF}$: 31.752
Power
Frequency (Hz)
mean_component_ica_s_all_,32
Peak Coordinates (mm)
(-2.5,-28.5,-54.5)
-8.2
8.2

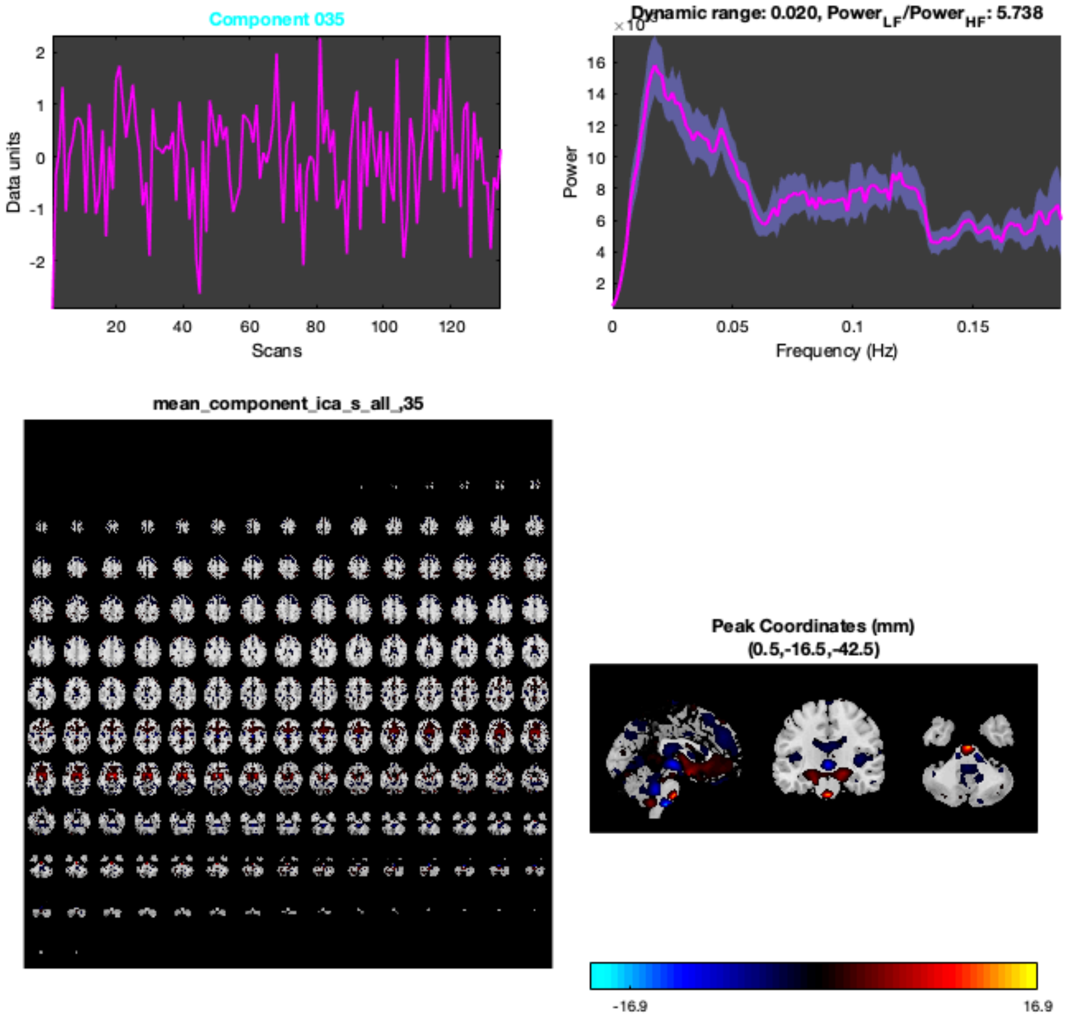

Spectral Summary

- **a) dynamic_range** - Difference between the peak power and minimum power at frequencies to the right of the peak.
- **b) fALFF** - Low frequency to high frequency power ratio.

ComponentNumber	DynamicRange	fALFF
1	0.029288	36.479
2	0.036517	55.664
3	0.029407	54.525
4	0.035968	47.973
5	0.028705	9.6307
6	0.036207	28.644
7	0.040002	38.331
8	0.036455	62.332
