# Supplementary material for: Neural Correlates of Vocal Pitch Compensation in Individuals Who Stutter
Source: Front Hum Neurosci. 2020 Feb 25;14:18. doi: 10.3389/fnhum.2020.00018 (PMC7053555; doi:10.3389/fnhum.2020.00018)
Supplement: Supplementary file 6 [file Data_Sheet_5.PDF]

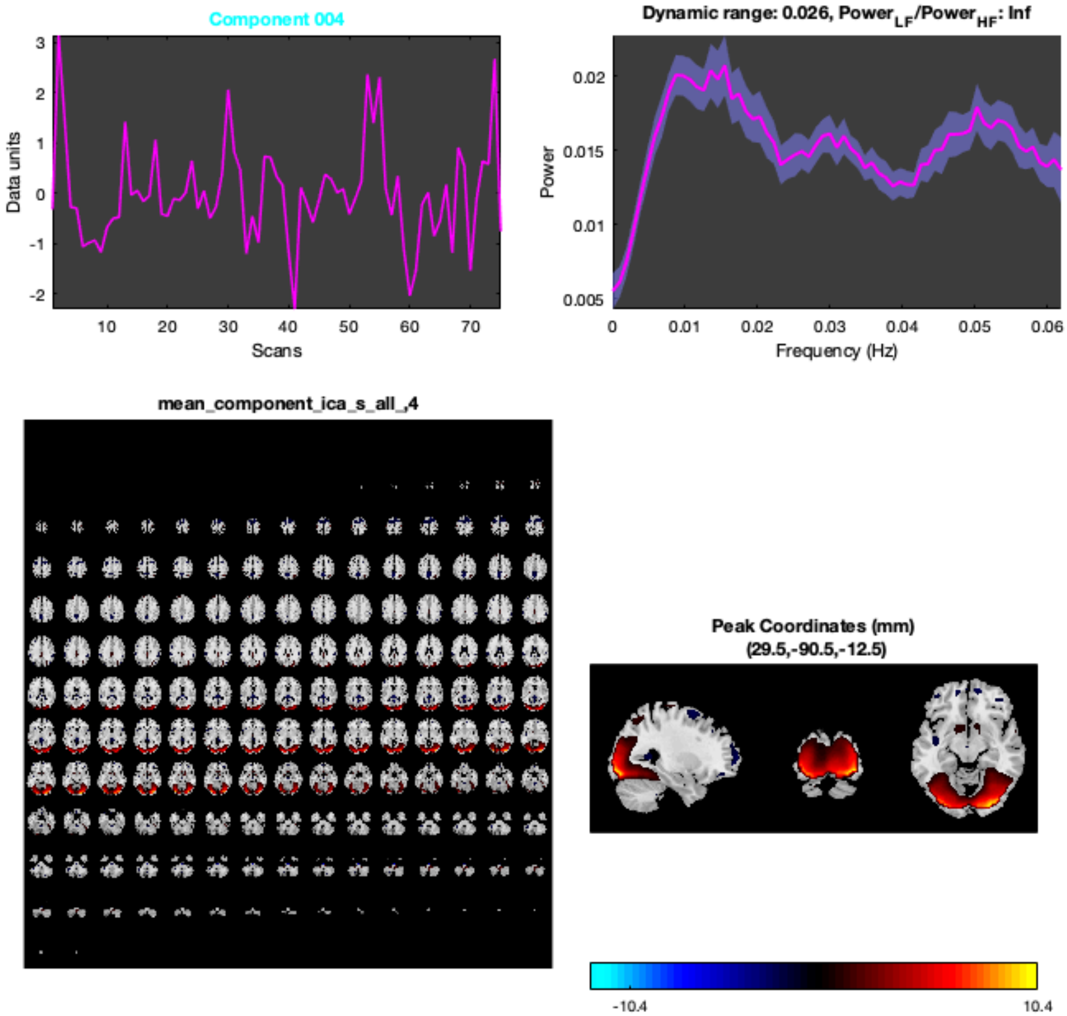
Component 004
Data units
Scans
Dynamic range: 0.026, Power$_{LF}$/Power$_{HF}$: Inf
Power
Frequency (Hz)
mean_component_ica_s_all_,4
Peak Coordinates (mm)
(29.5,-90.5,-12.5)
-10.4
10.4

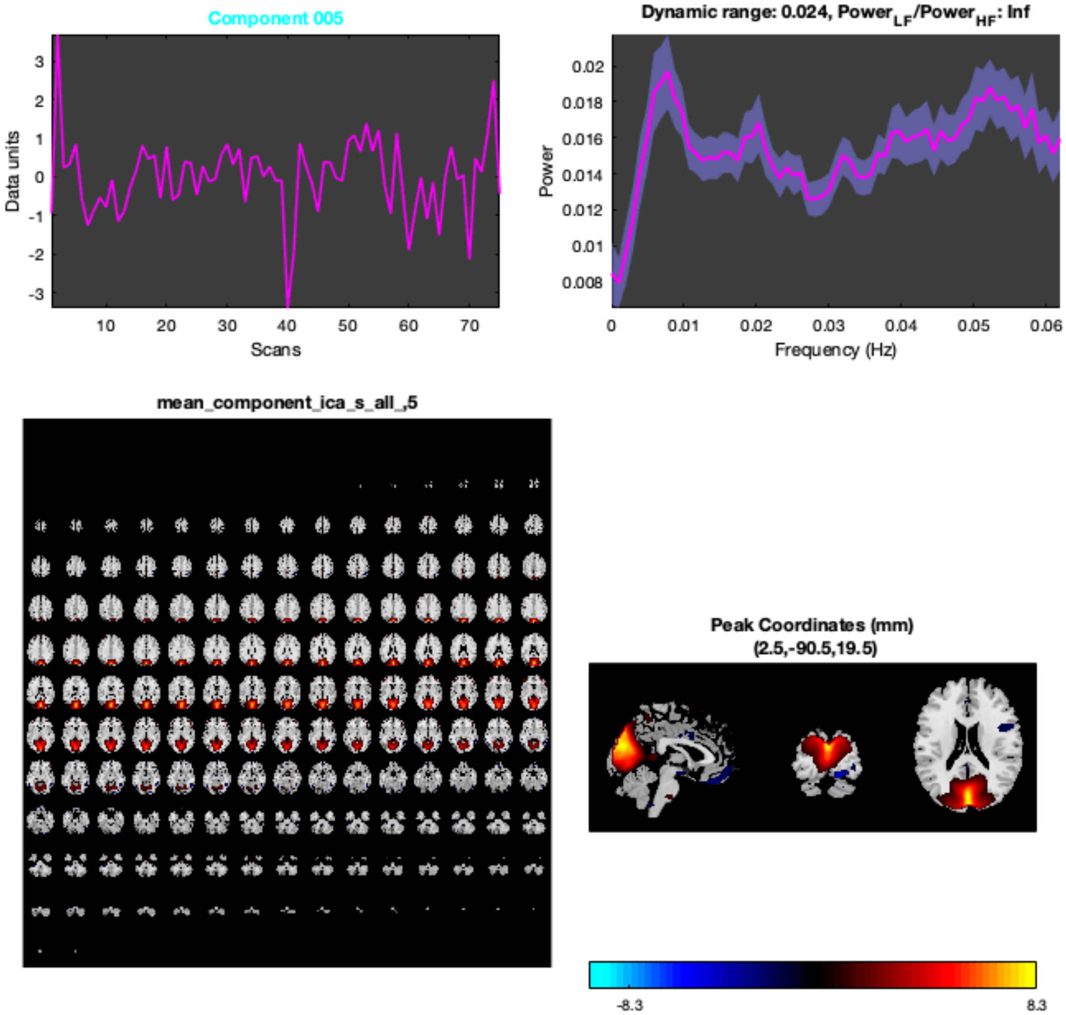
Component 005
Data units
Scans
Dynamic range: 0.024, Power$_{LF}$/Power$_{HF}$: Inf
Power
Frequency (Hz)
mean_component_ica_s_all_,5
Peak Coordinates (mm)
(2.5,-90.5,19.5)
-8.3
8.3

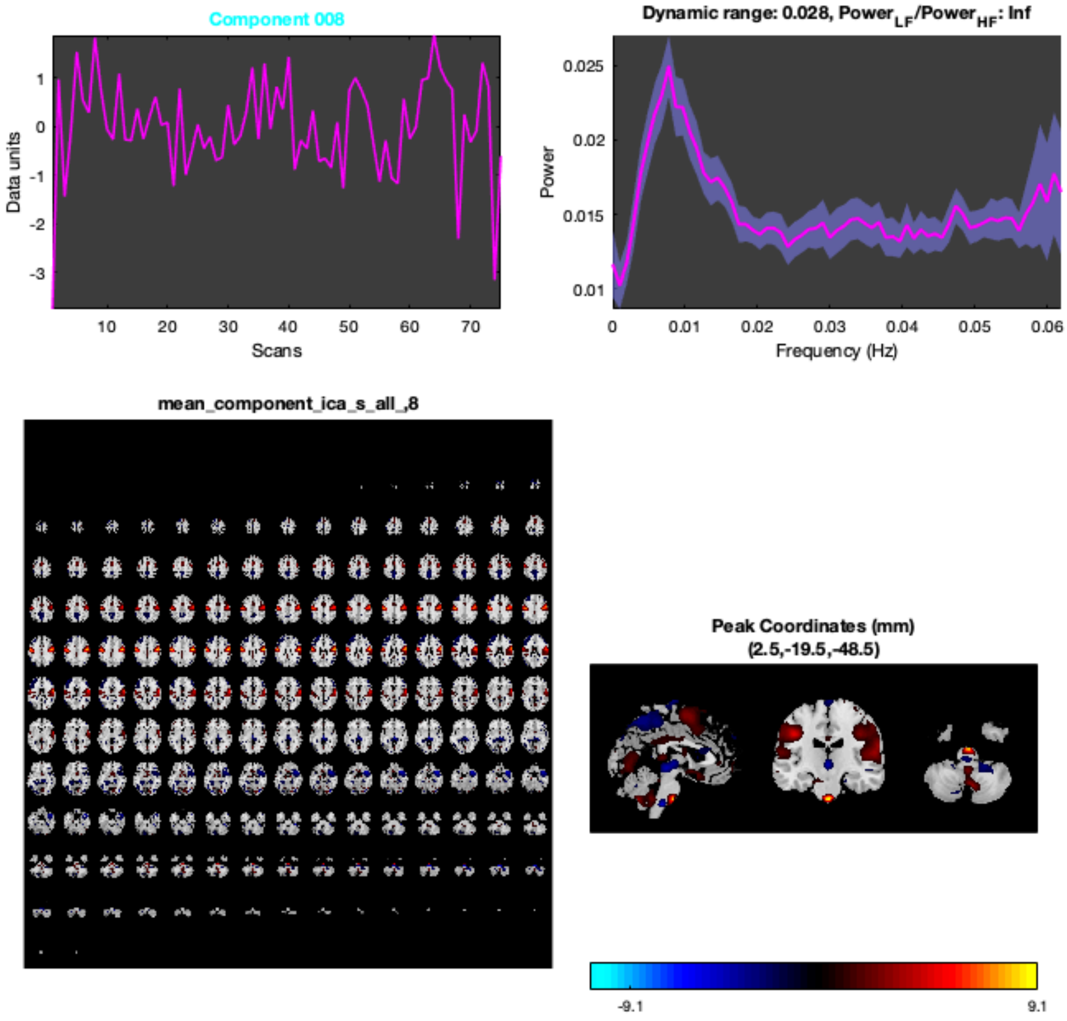
Component 008
Data units
Scans
Dynamic range: 0.028, Power$_{LF}$/Power$_{HF}$: Inf
Power
Frequency (Hz)
mean_component_ica_s_all_,8
Peak Coordinates (mm)
(2.5,-19.5,-48.5)
-9.1
9.1

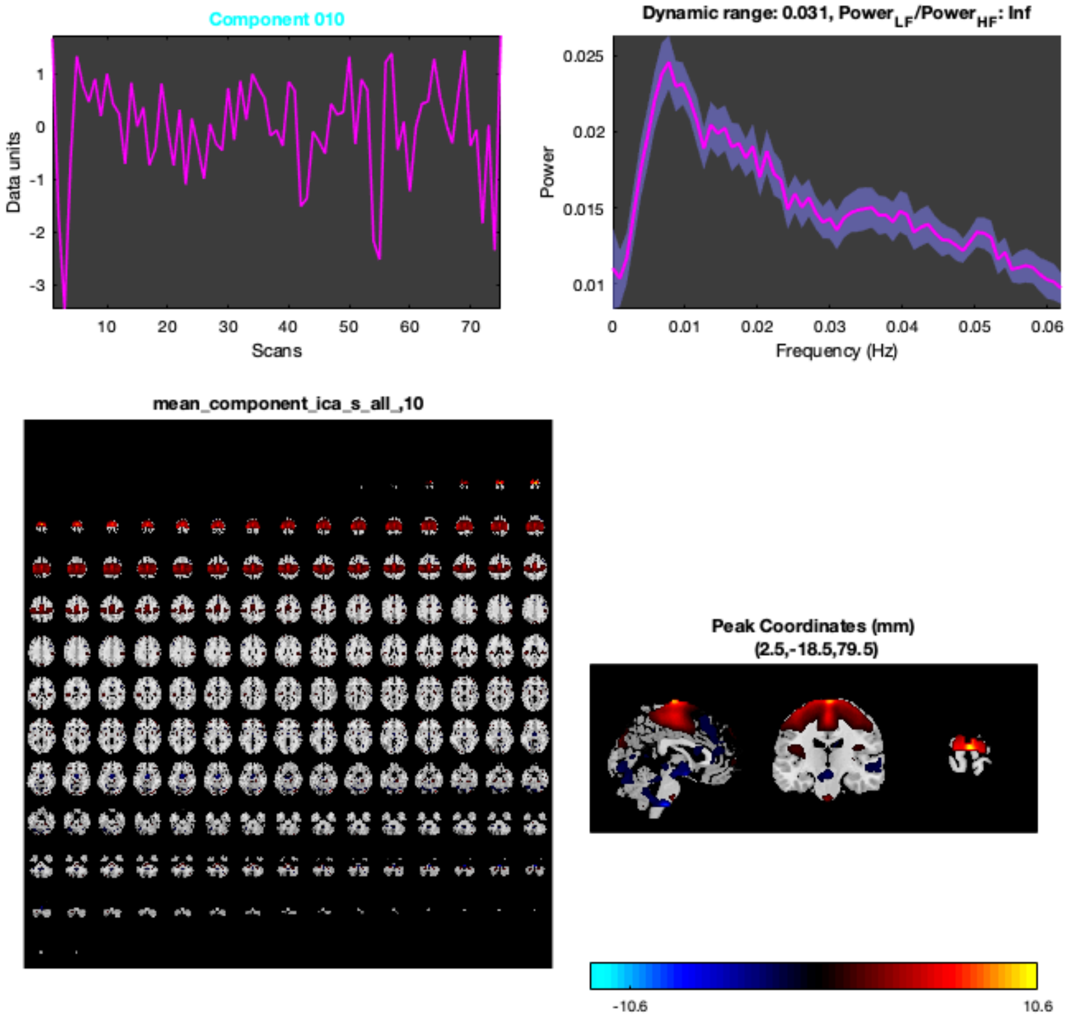
Component 010
Data units
Scans
Dynamic range: 0.031, Power_LF/Power_HF: Inf
Power
Frequency (Hz)
mean_component_ica_s_all_,10
Peak Coordinates (mm)
(2.5,-18.5,79.5)
-10.6
10.6

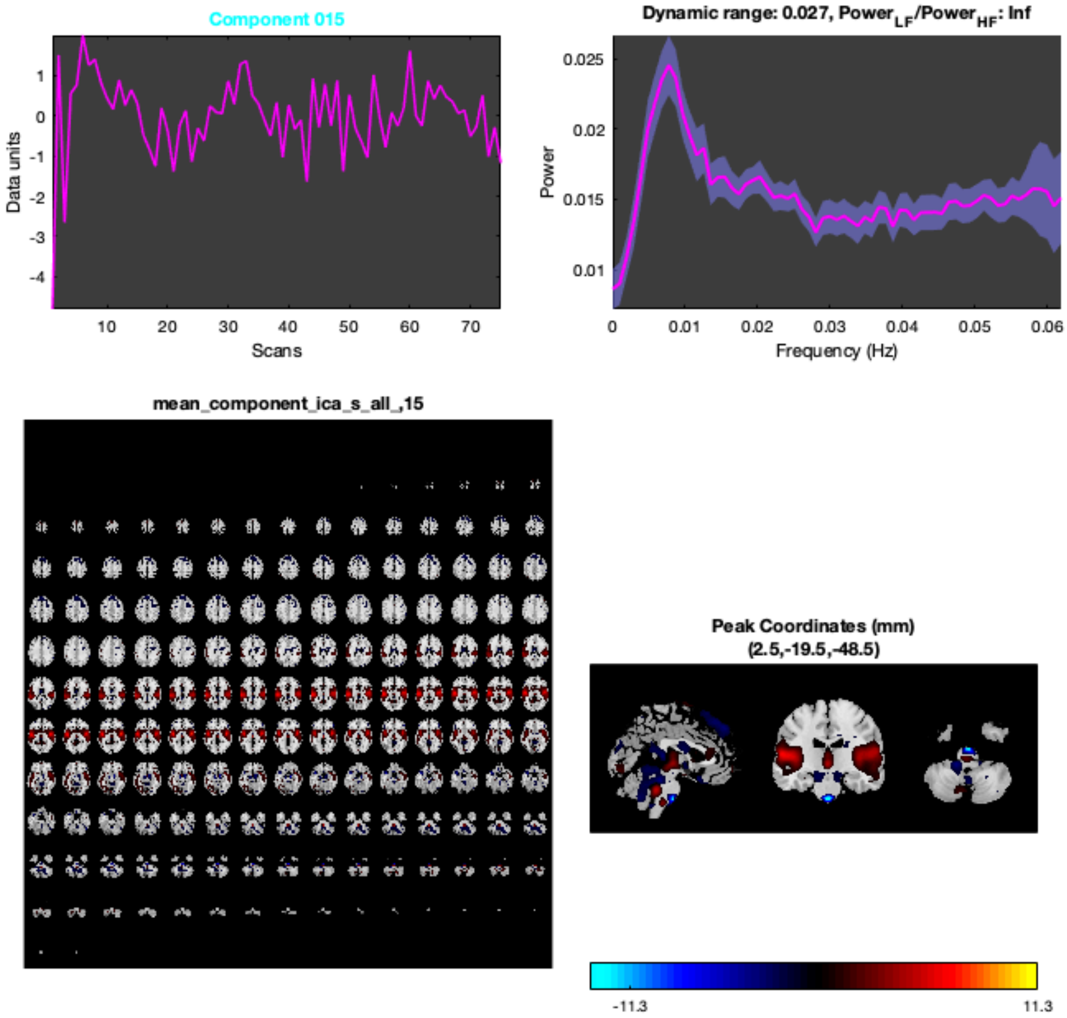
Component 015
Data units
Scans
Dynamic range: 0.027, Power_LF/Power_HF: Inf
Power
Frequency (Hz)
mean_component_ica_s_all_,15
Peak Coordinates (mm)
(2.5,-19.5,-48.5)
-11.3
11.3

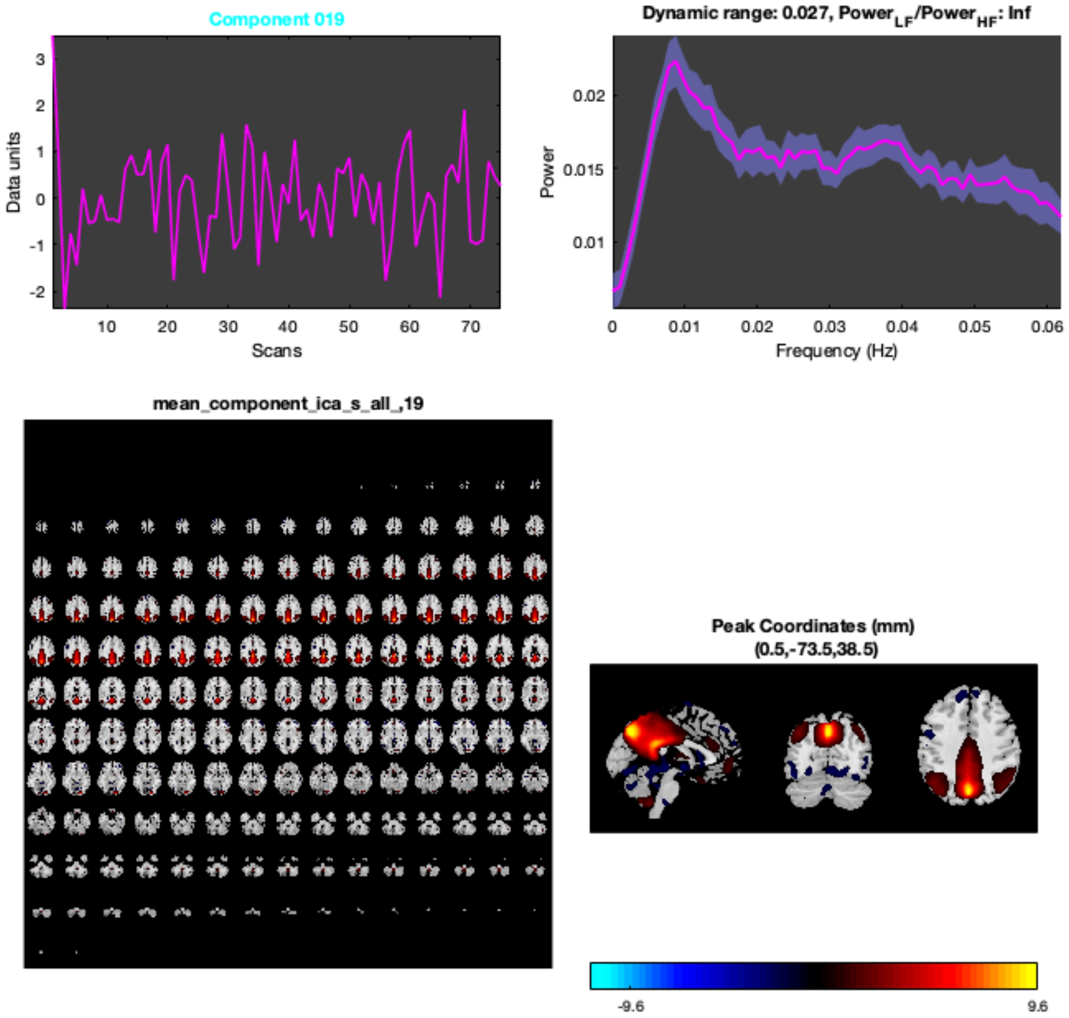
Component 019
Data units
Scans
Dynamic range: 0.027, Power$_{LF}$/Power$_{HF}$: Inf
Power
Frequency (Hz)
mean_component_ica_s_all_,19
Peak Coordinates (mm)
(0.5,-73.5,38.5)
-9.6
9.6

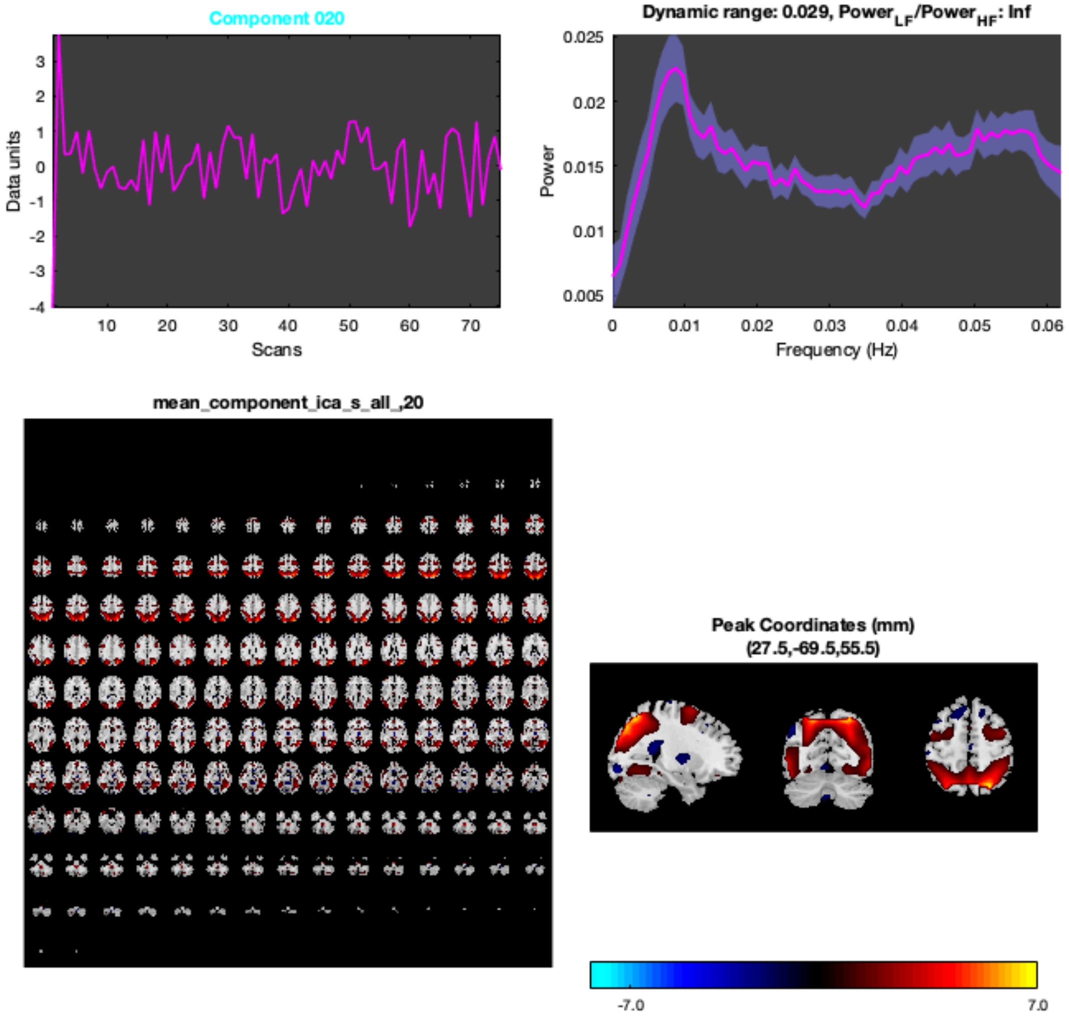
Component 020
Data units
Scans
Dynamic range: 0.029, Power$_{LF}$/Power$_{HF}$: Inf
Power
Frequency (Hz)
mean_component_ica_s_all_,20
Peak Coordinates (mm)
(27.5,-69.5,55.5)
-7.0
7.0

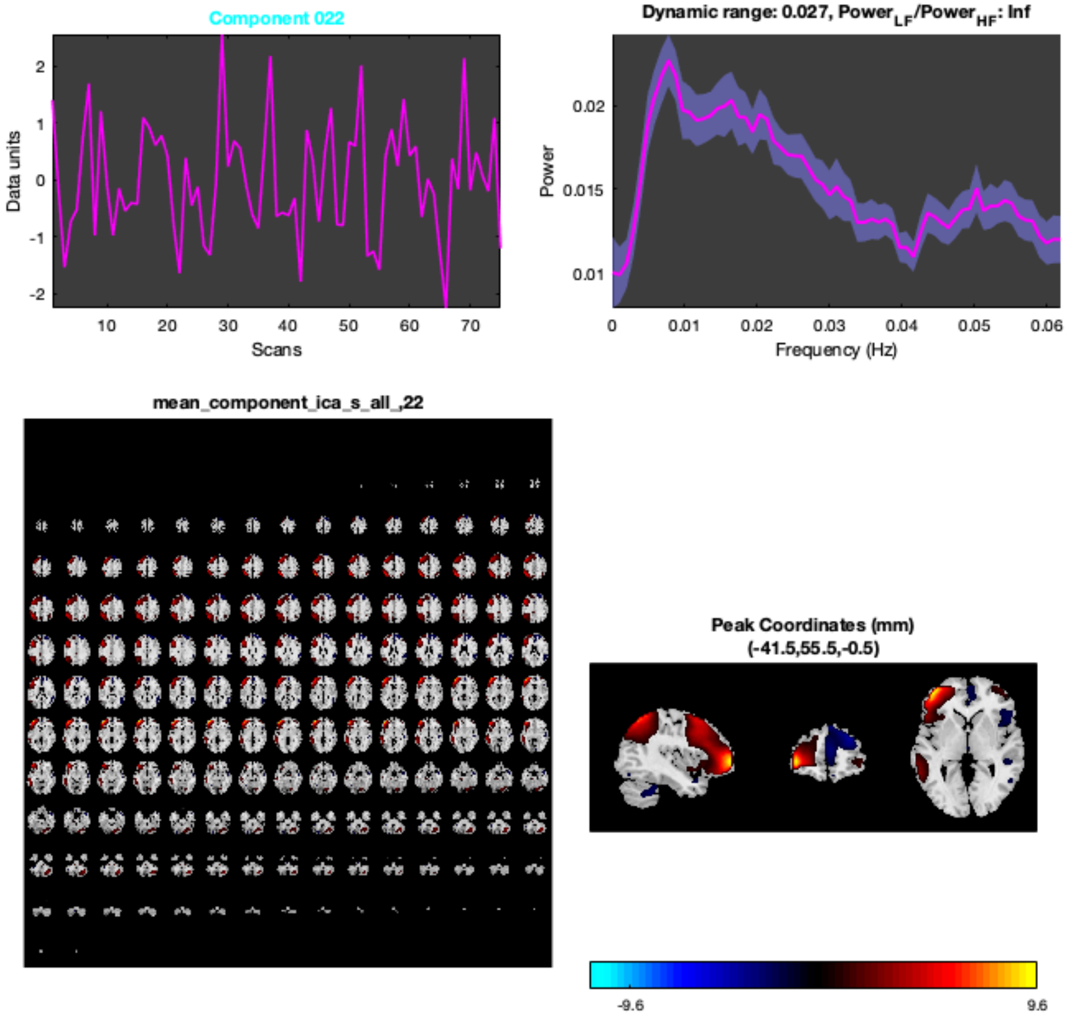
Component 022
Data units
Scans
Dynamic range: 0.027, Power$_{LF}$/Power$_{HF}$: Inf
Power
Frequency (Hz)
mean_component_ica_s_all_,22
Peak Coordinates (mm)
(-41.5,55.5,-0.5)
-9.6
9.6

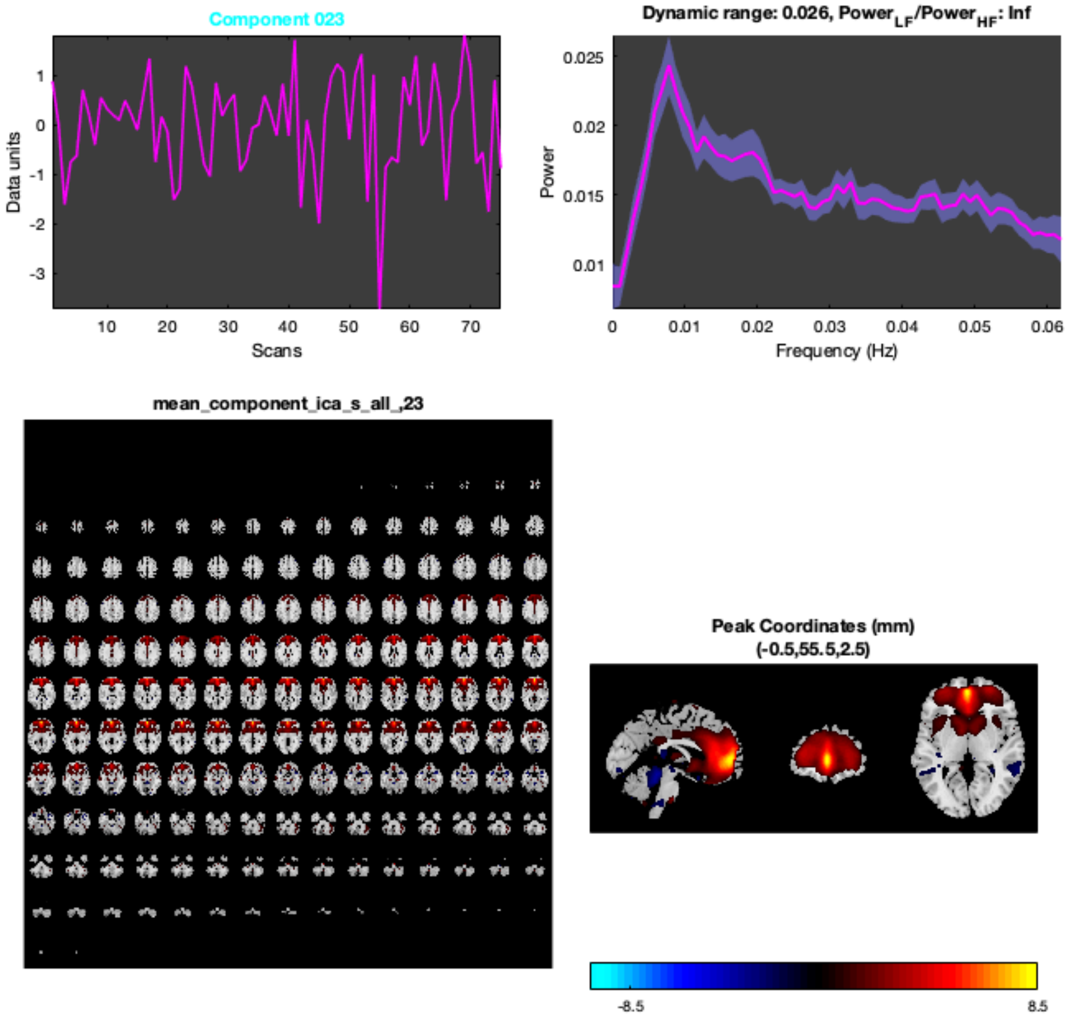

Component 023
Data units
Scans
Dynamic range: 0.026, Power_LF/Power_HF: Inf
Power
Frequency (Hz)
mean_component_ica_s_all_,23
Peak Coordinates (mm)
(-0.5,55.5,2.5)
-8.5
8.5

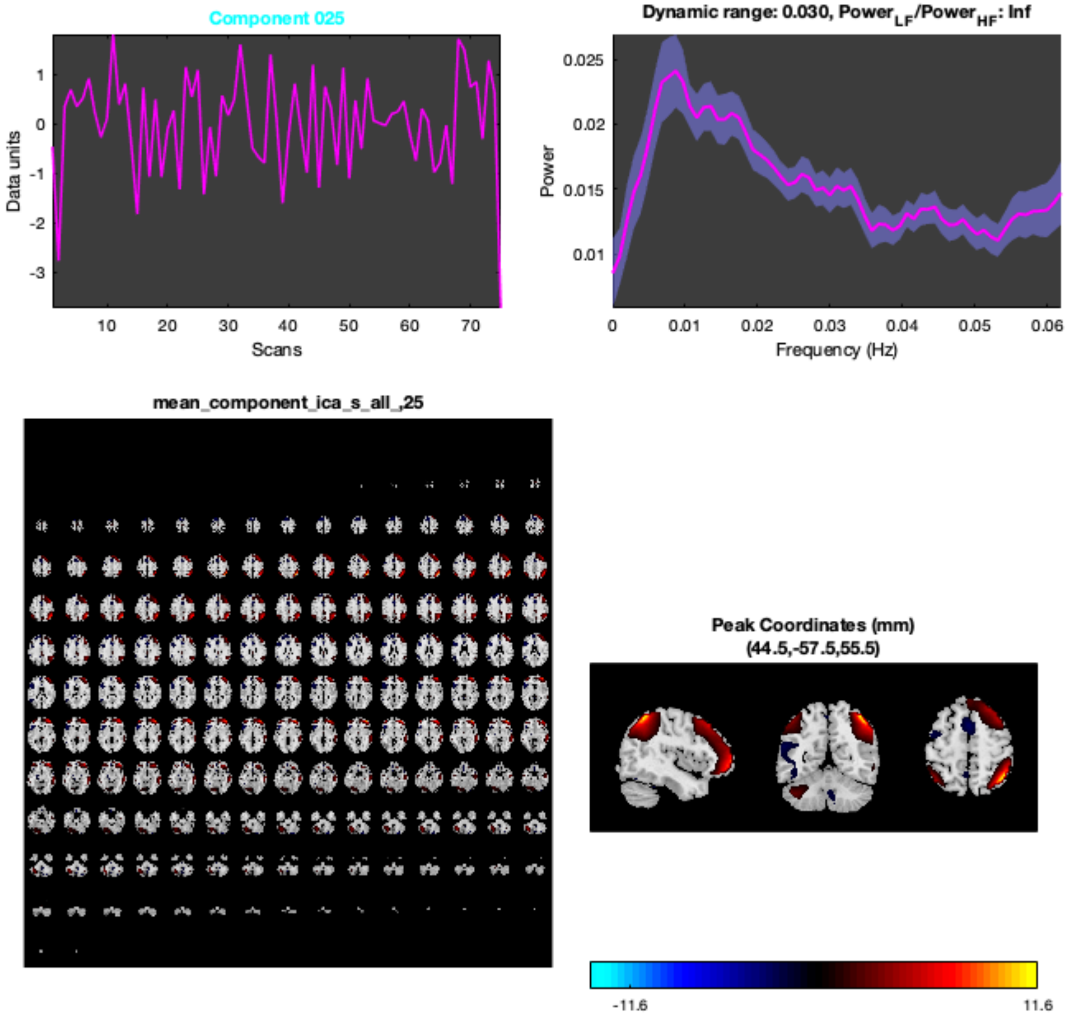
Component 025
Data units
Scans
Dynamic range: 0.030, Power$_{LF}$/Power$_{HF}$: Inf
Power
Frequency (Hz)
mean_component_ica_s_all_,25
Peak Coordinates (mm)
(44.5,-57.5,55.5)
-11.6
11.6

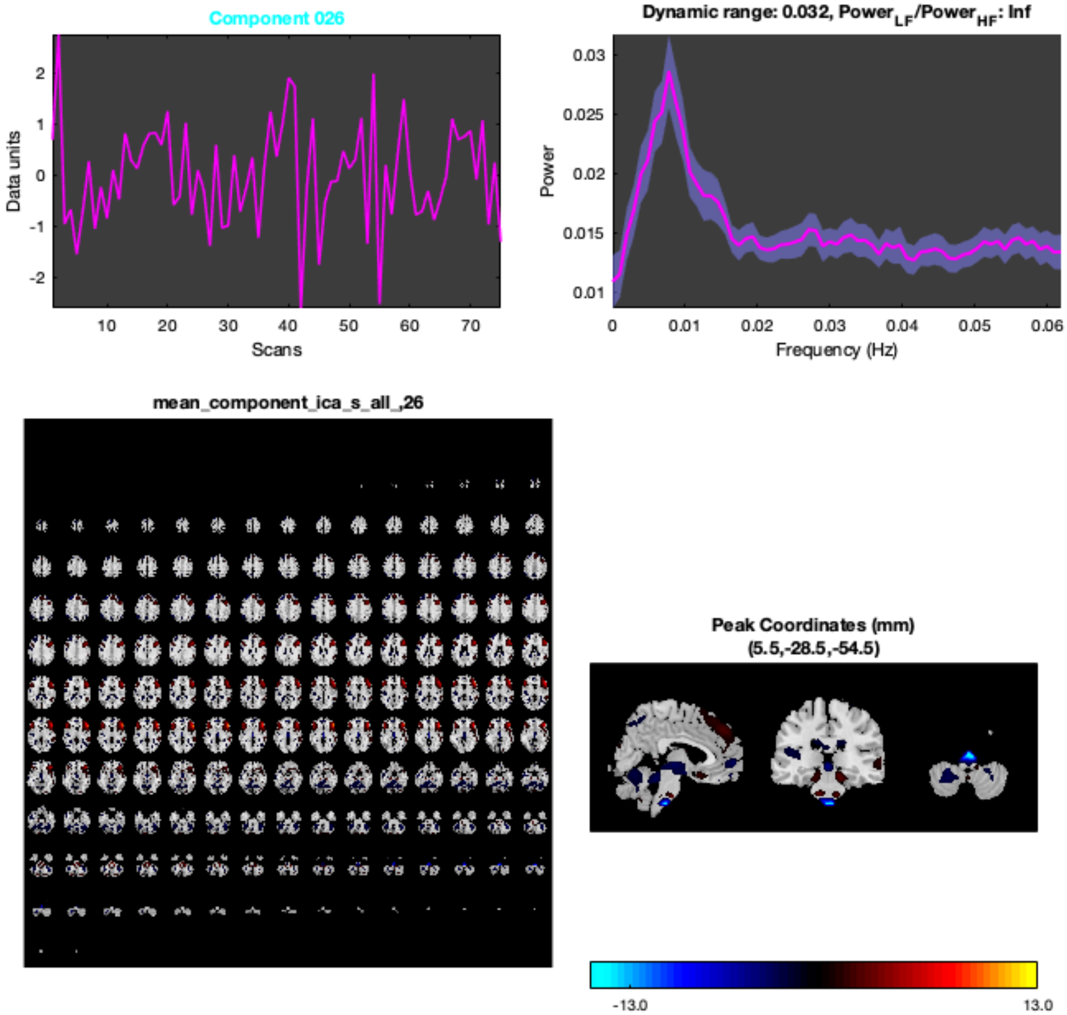
Component 026
Data units
Scans
Dynamic range: 0.032, Power$_{LF}$/Power$_{HF}$: Inf
Power
Frequency (Hz)
mean_component_ica_s_all_,26
Peak Coordinates (mm)
(5.5,-28.5,-54.5)
-13.0
13.0

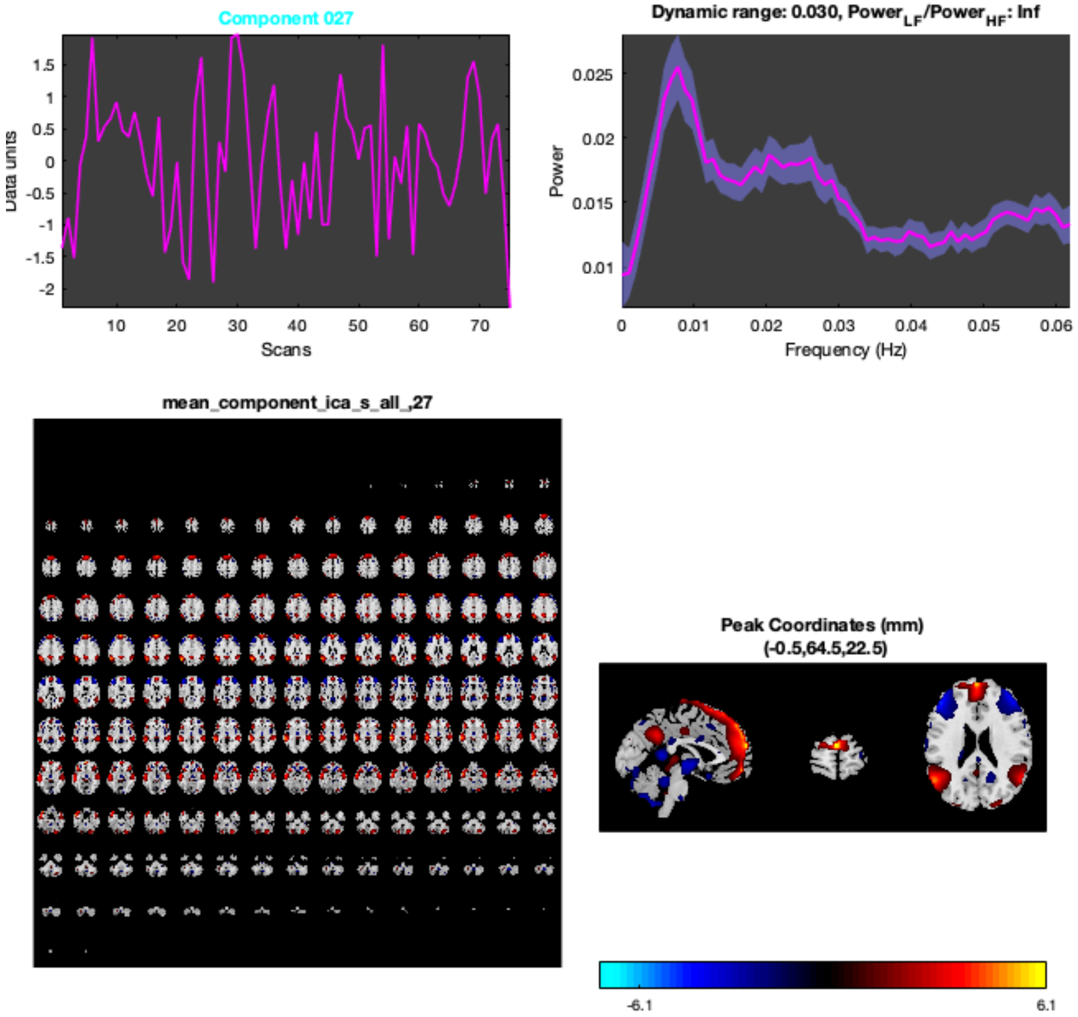
Component 027
Data units
Scans
Dynamic range: 0.030, Power_LF/Power_HF: Inf
Power
Frequency (Hz)
mean_component_ica_s_all_,27
Peak Coordinates (mm)
(-0.5,64.5,22.5)
-6.1
6.1

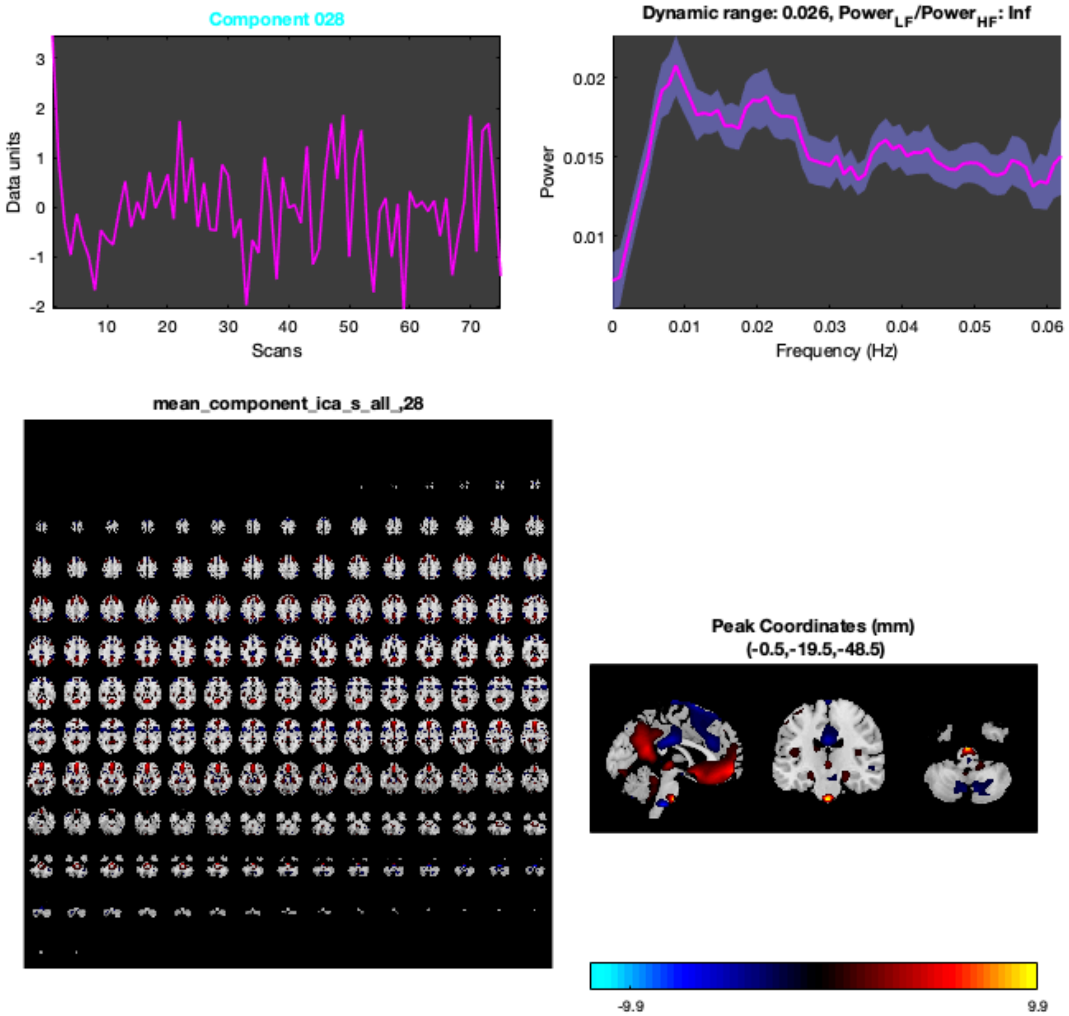

Spectral Summary

- **a) dynamic_range** - Difference between the peak power and minimum power at frequencies to the right of the peak.
- **b) fALFF** - Low frequency to high frequency power ratio.

ComponentNumber	*DynamicRange*	*fALFF*
1	*0.020048*	*Inf*
2	*0.020829*	*Inf*
3	*0.019831*	*Inf*
4	*0.025715*	*Inf*
5	*0.024474*	*Inf*
6	*0.022603*	*Inf*
7	*0.023544*	*Inf*
8	*0.02805*	*Inf*
