# Supplementary figures and images for: Neural Correlates of Vocal Pitch Compensation in Individuals Who Stutter
Source: Front Hum Neurosci. 2020 Feb 25;14:18. doi: 10.3389/fnhum.2020.00018 (PMC7053555; doi:10.3389/fnhum.2020.00018)

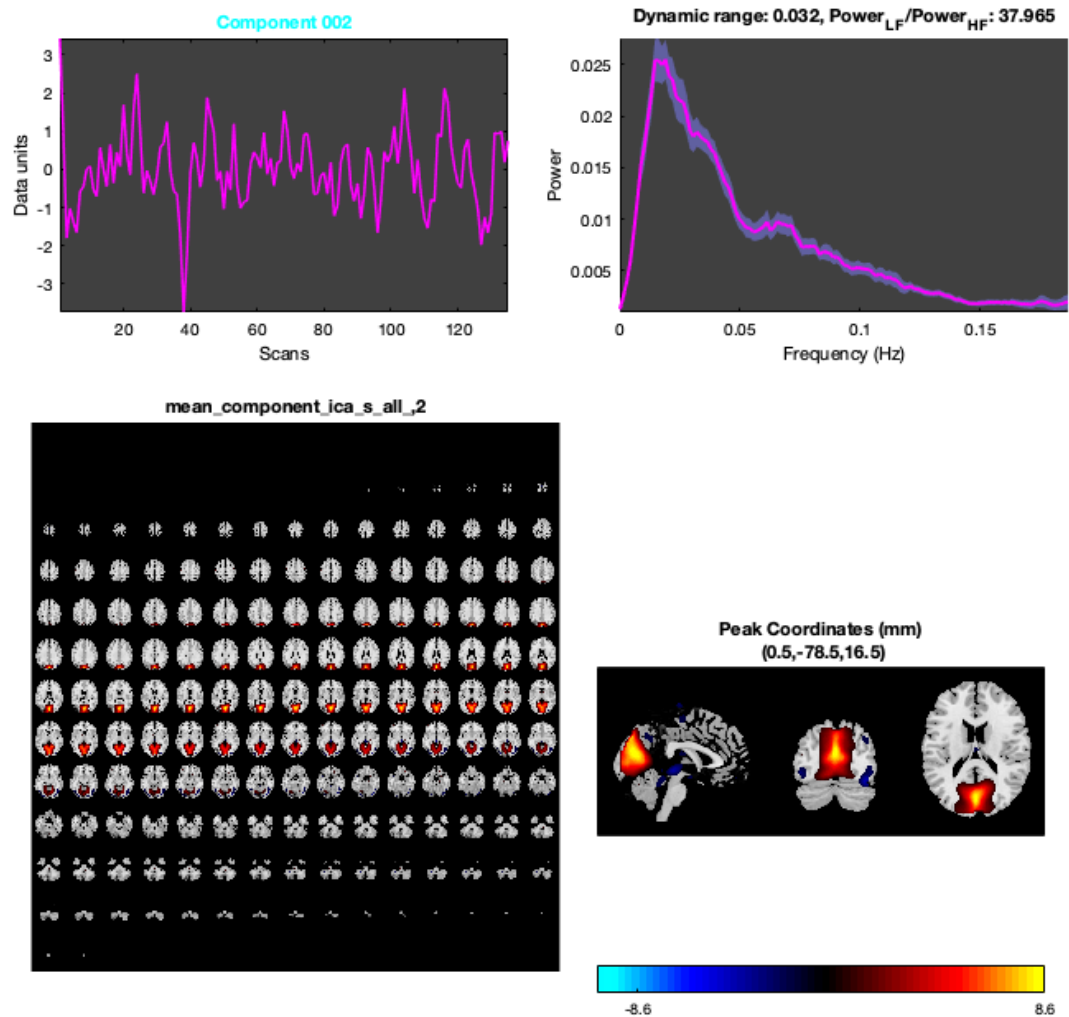

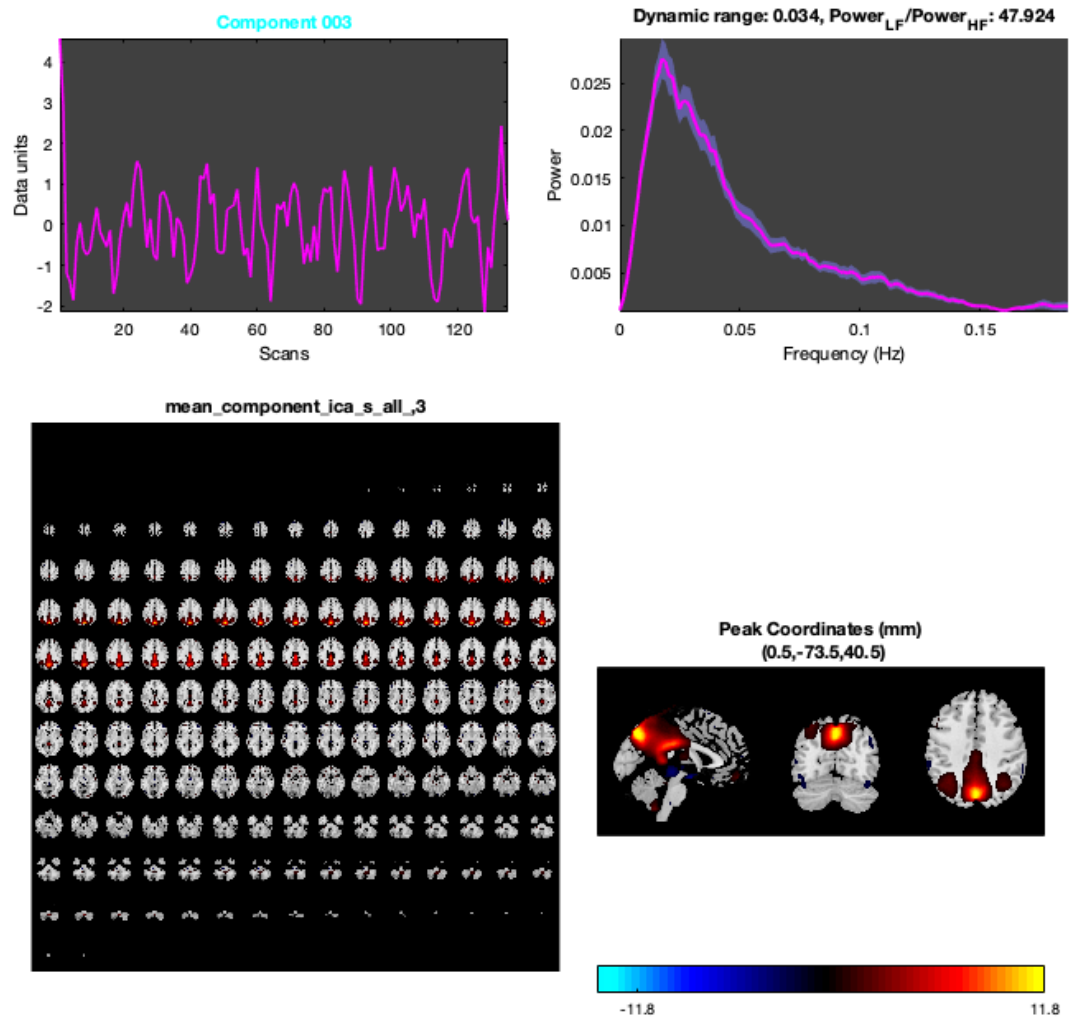

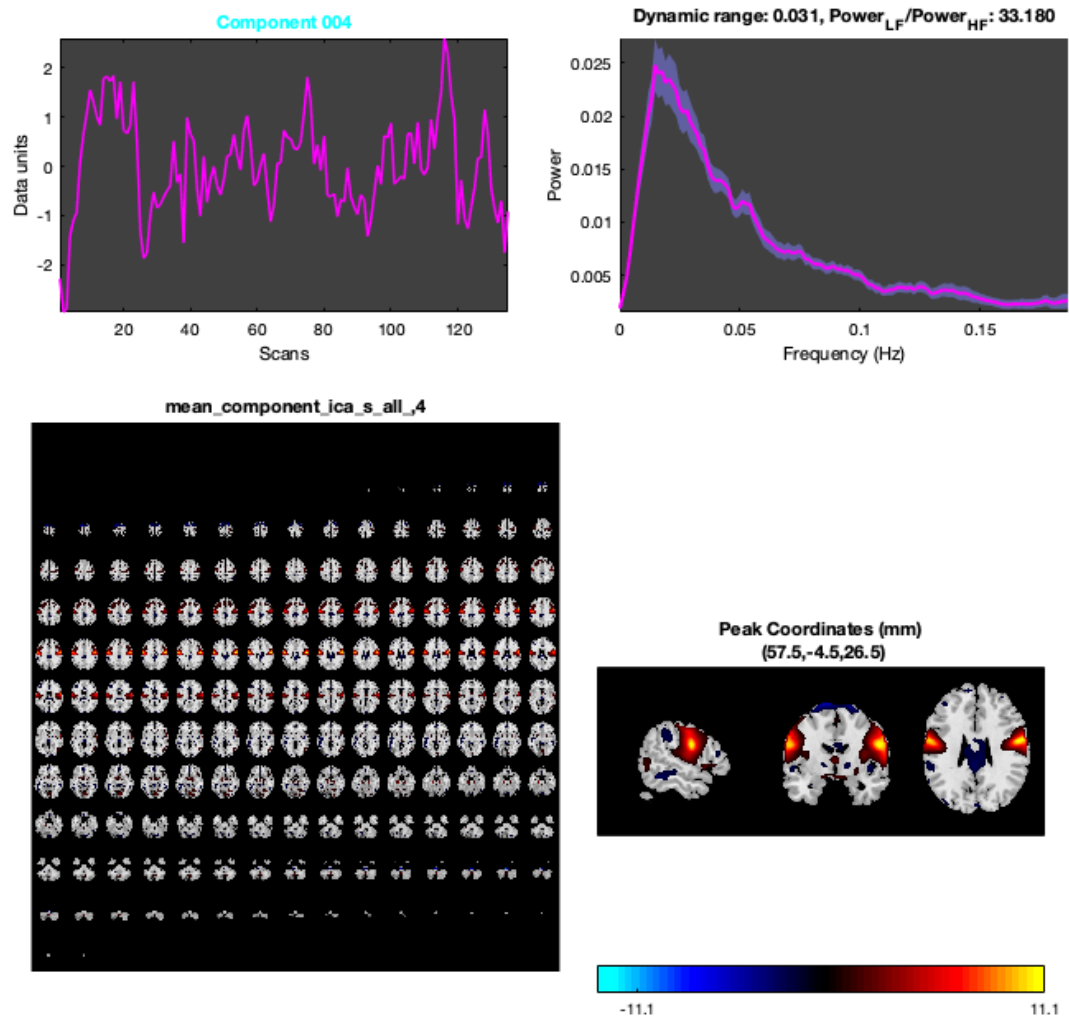

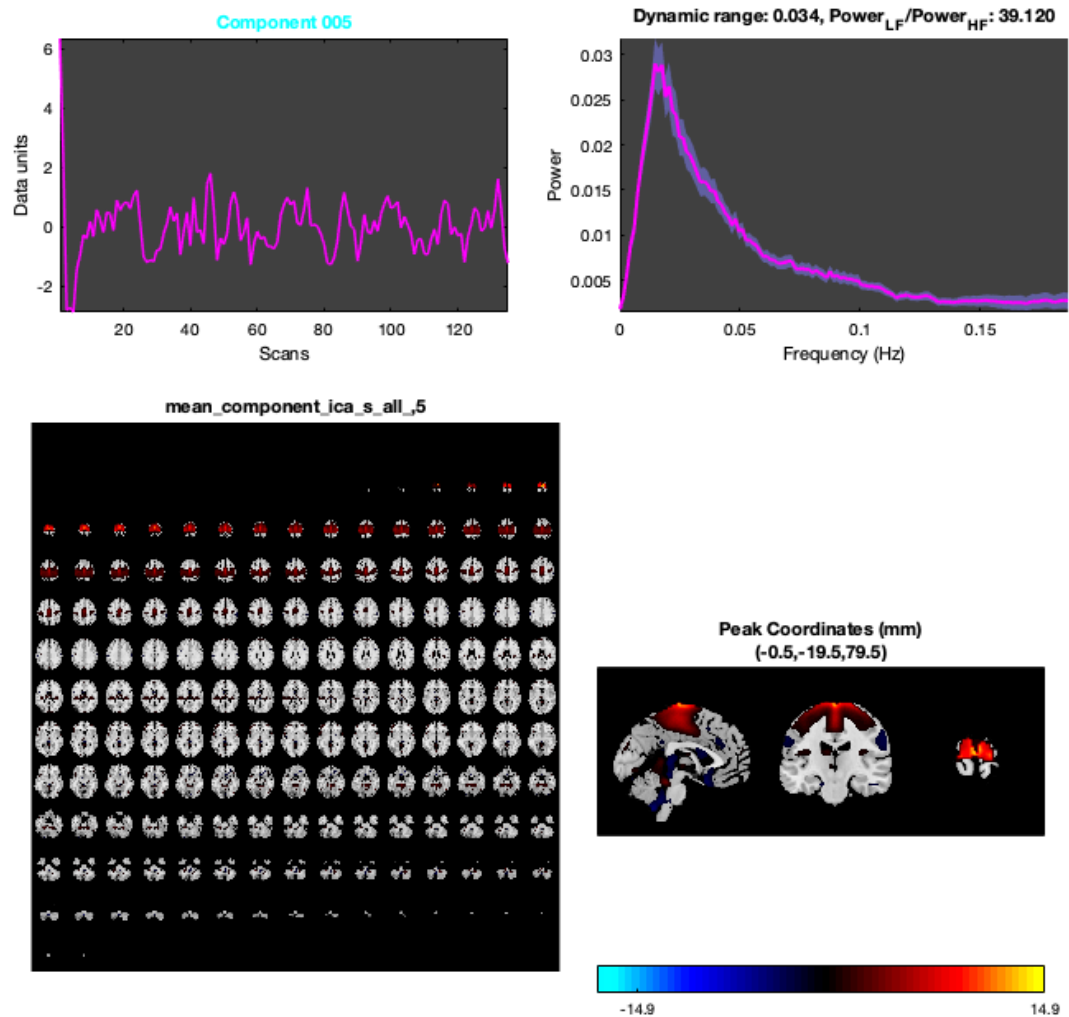

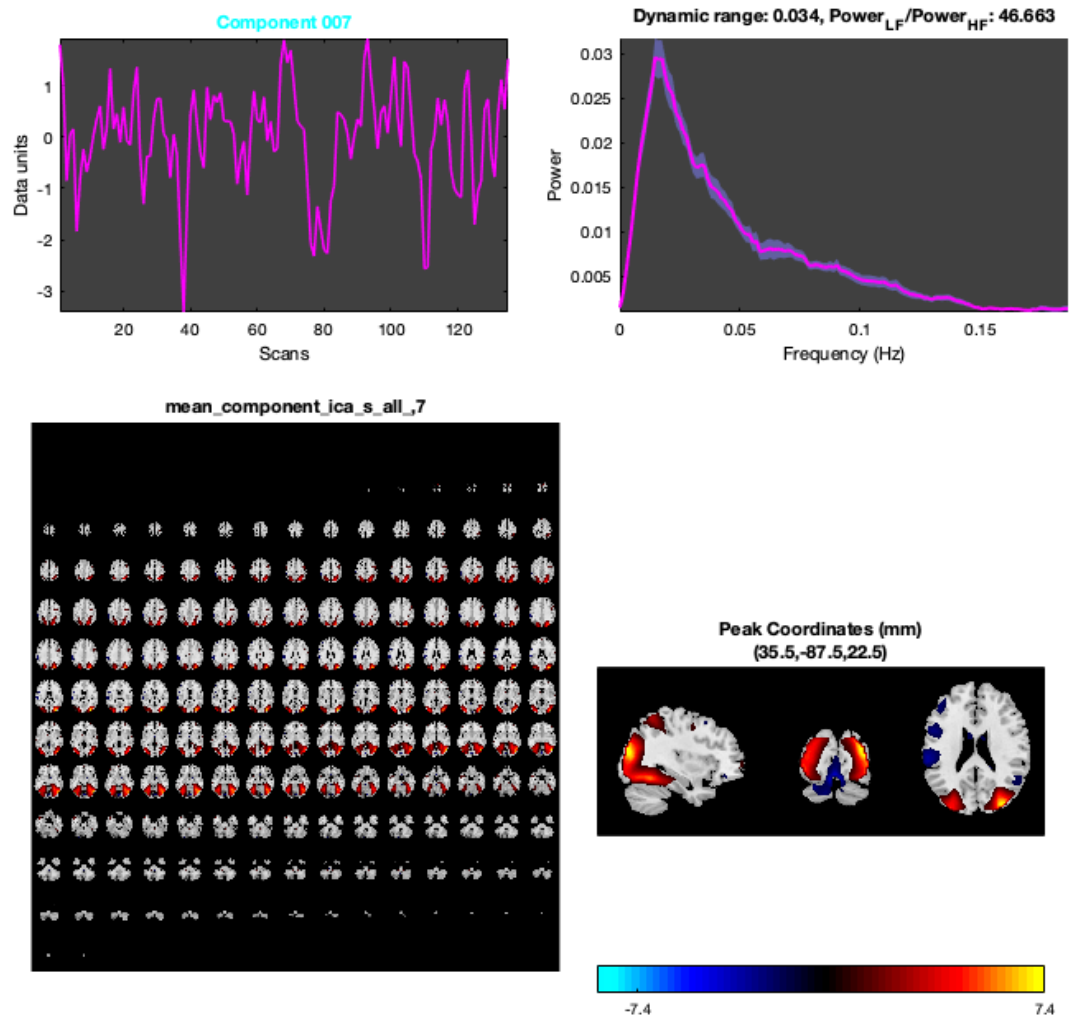

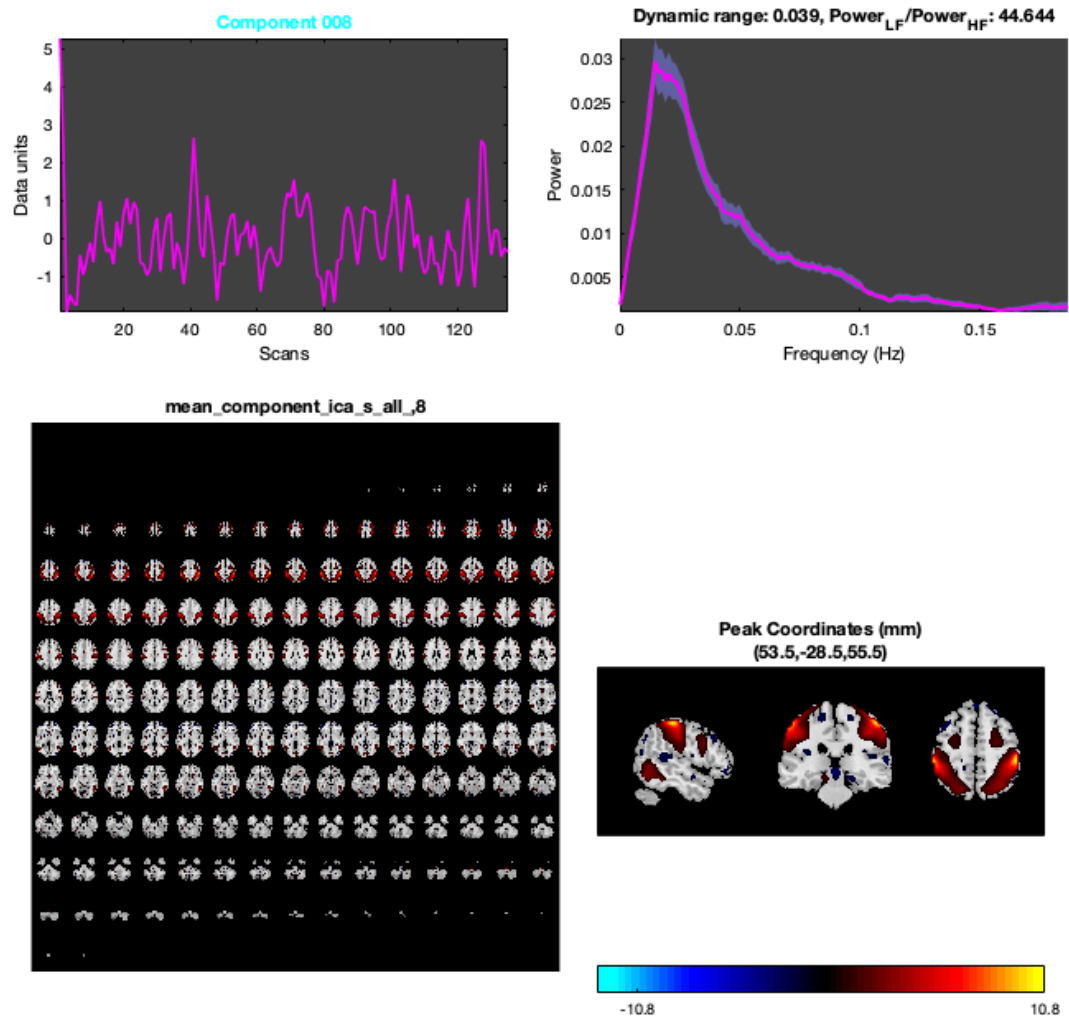

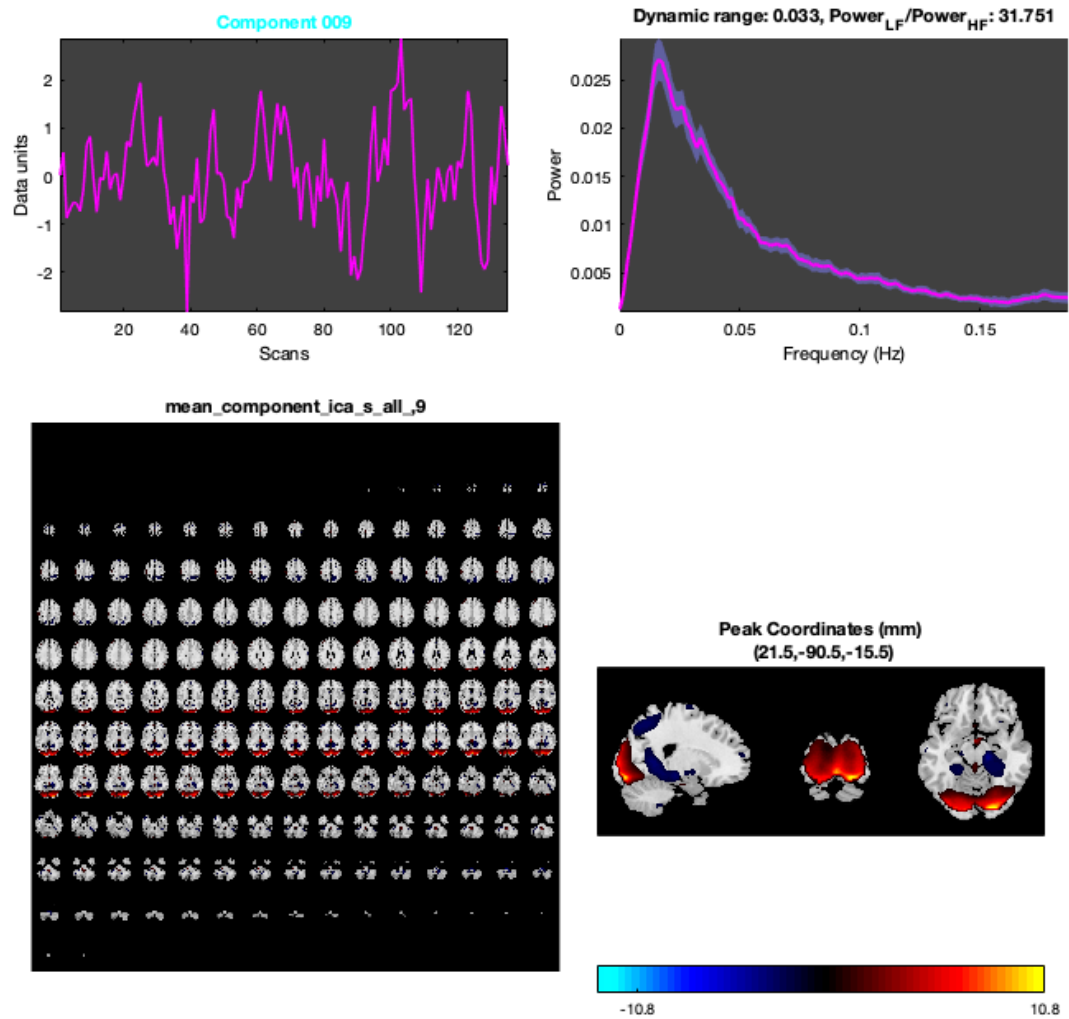

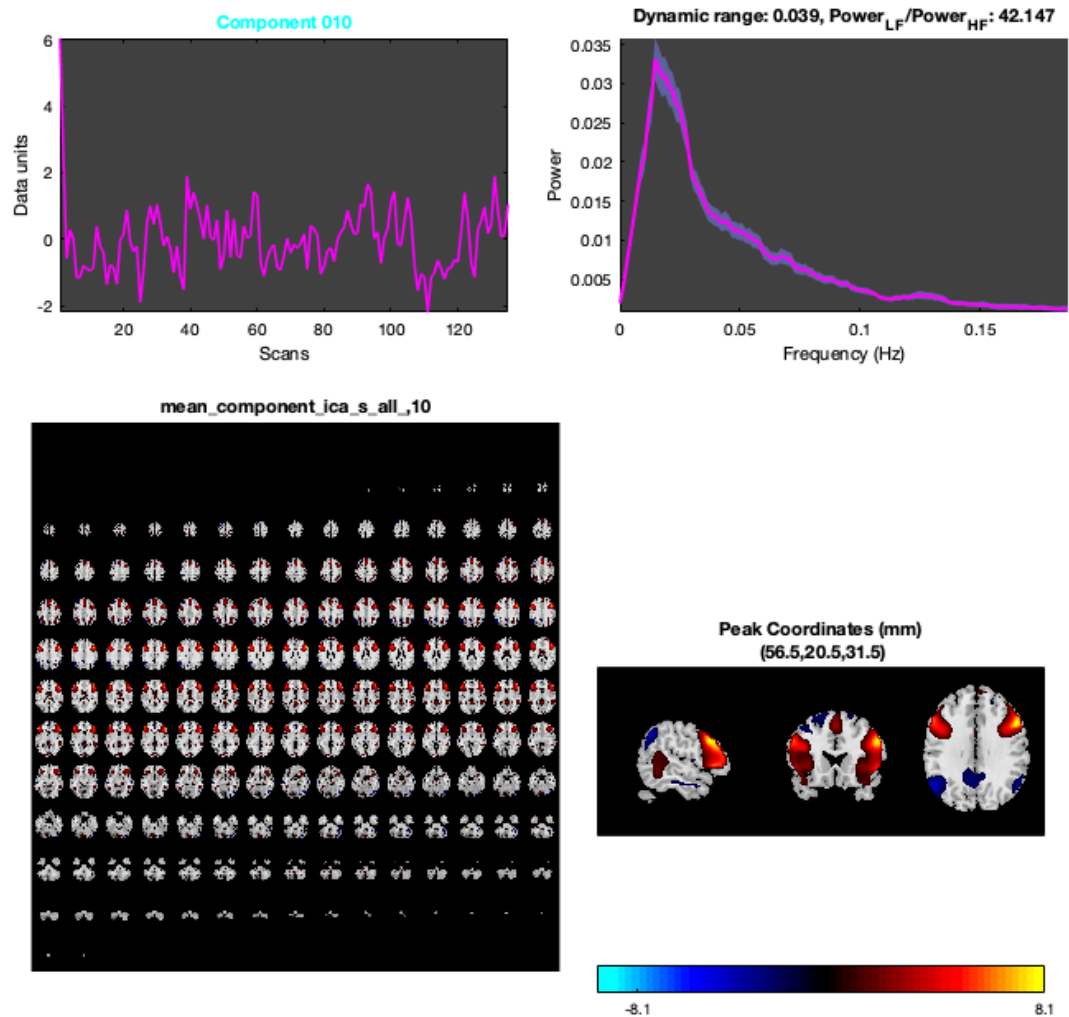

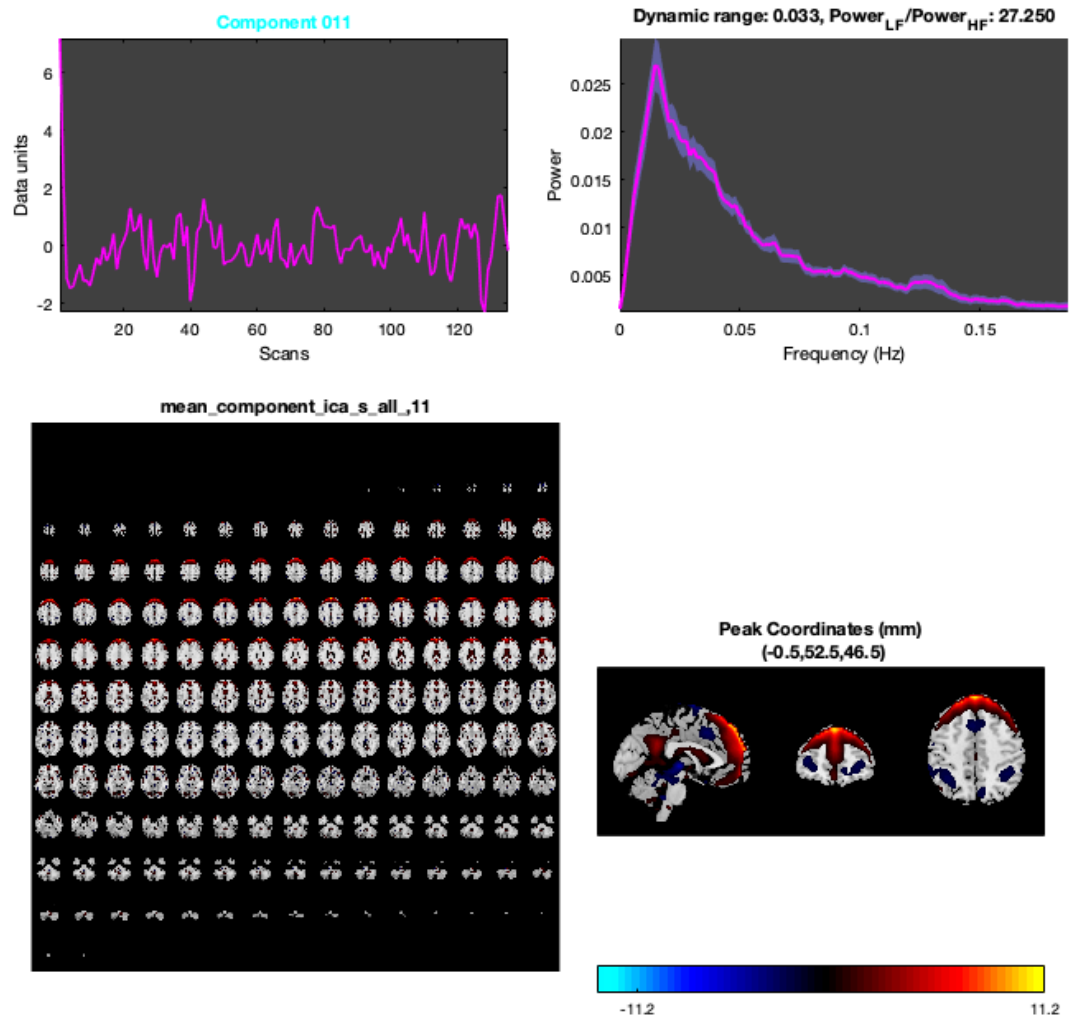

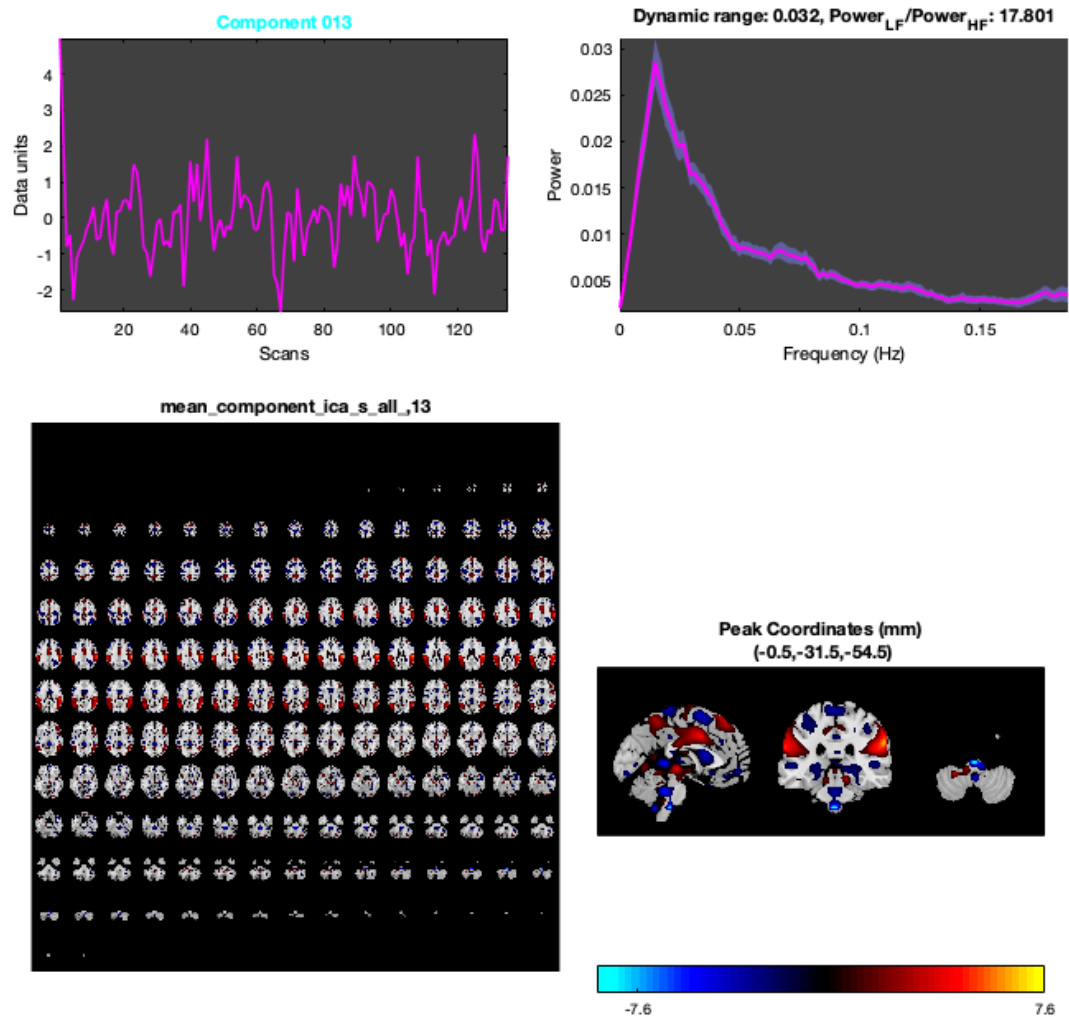

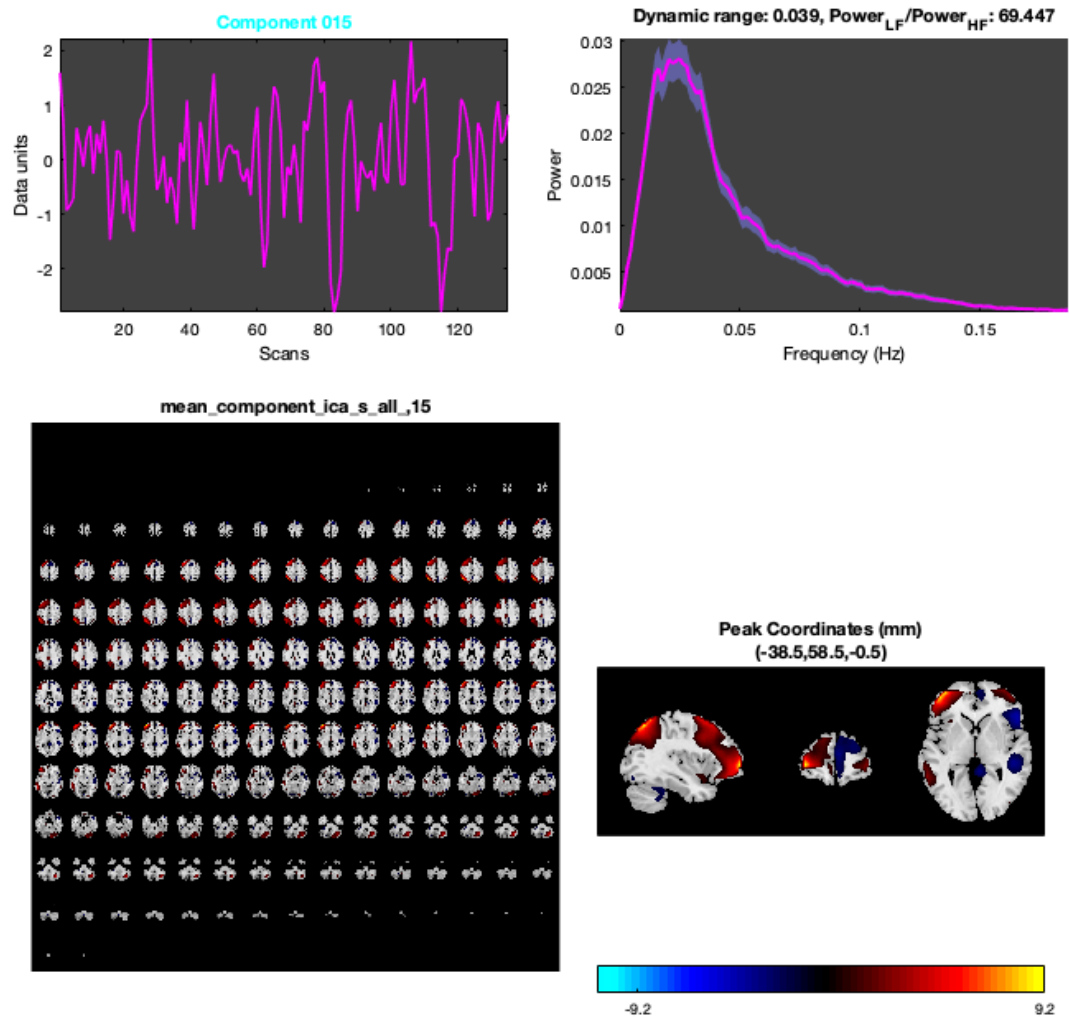

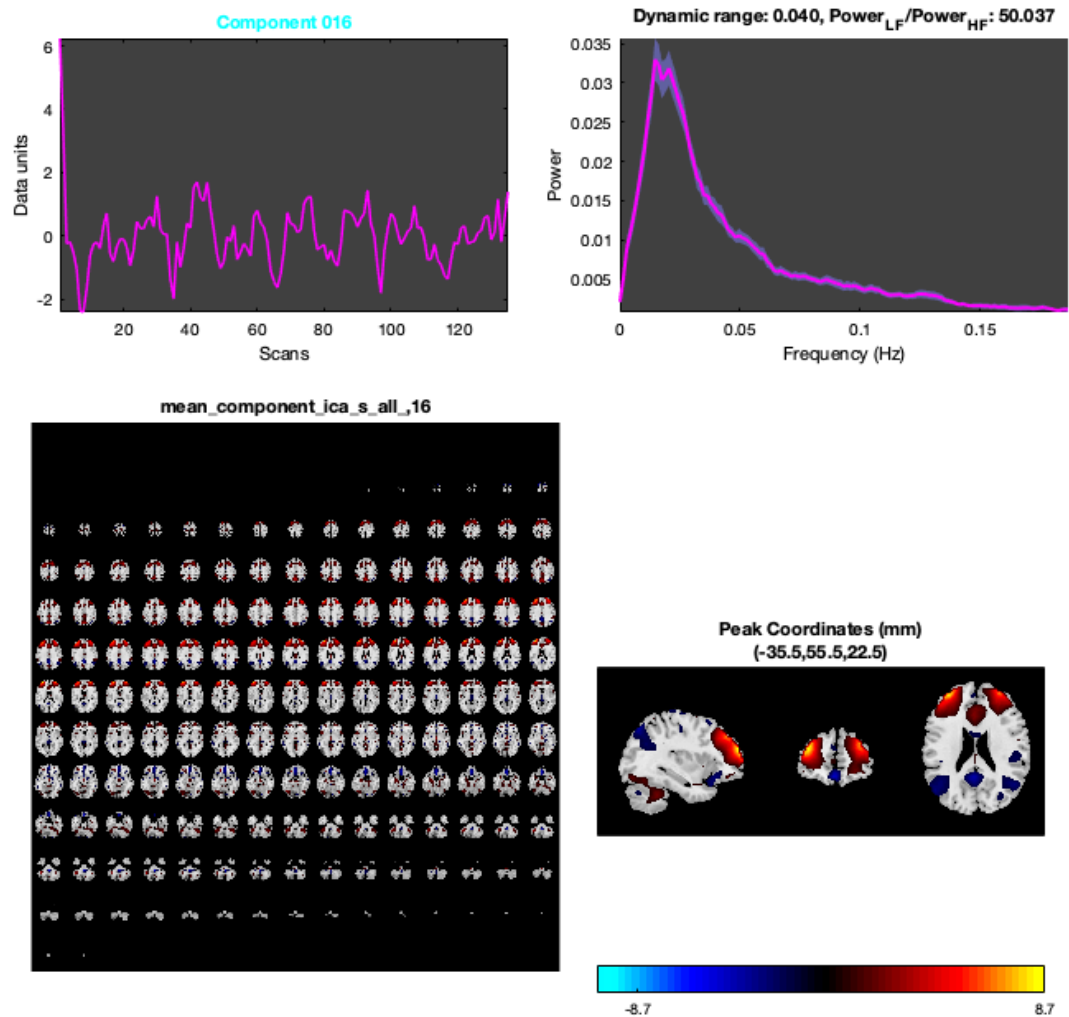

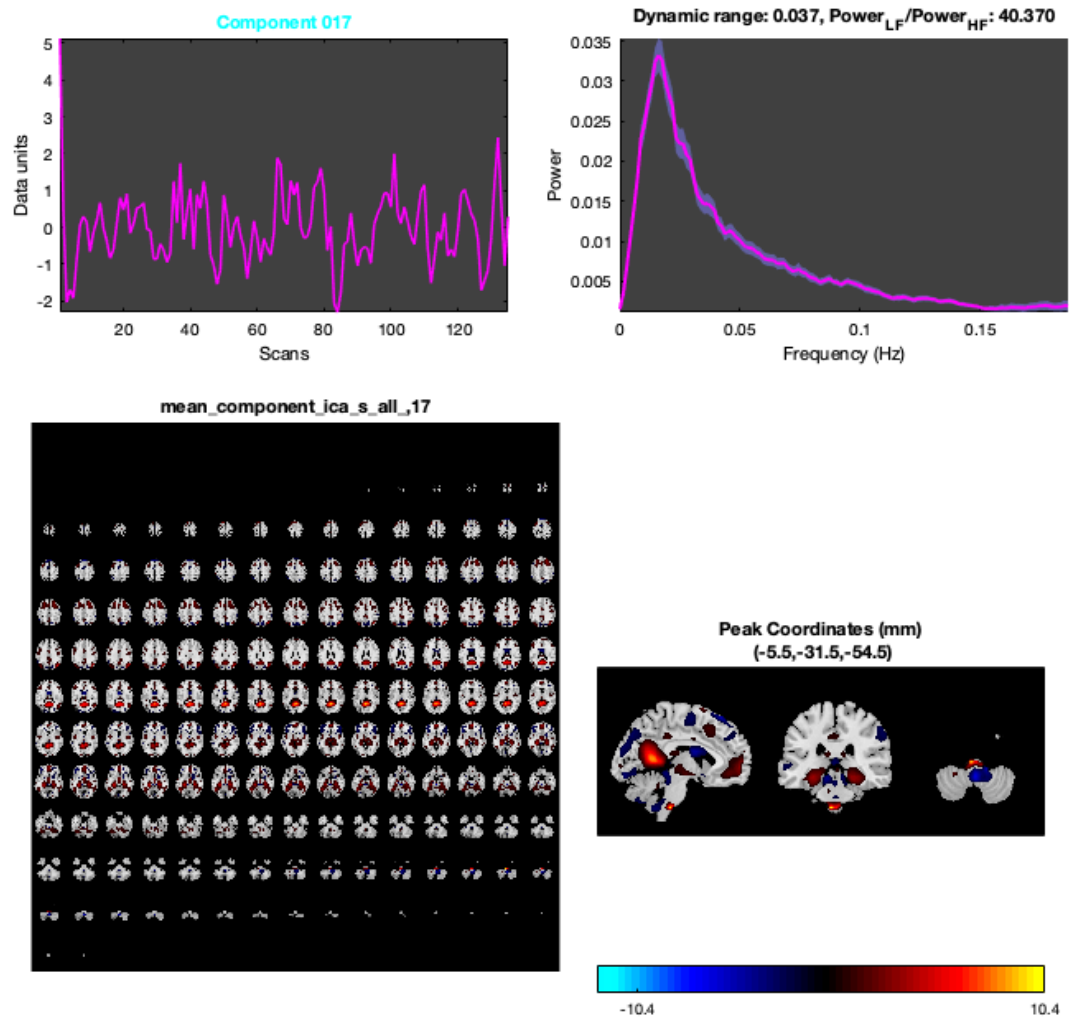

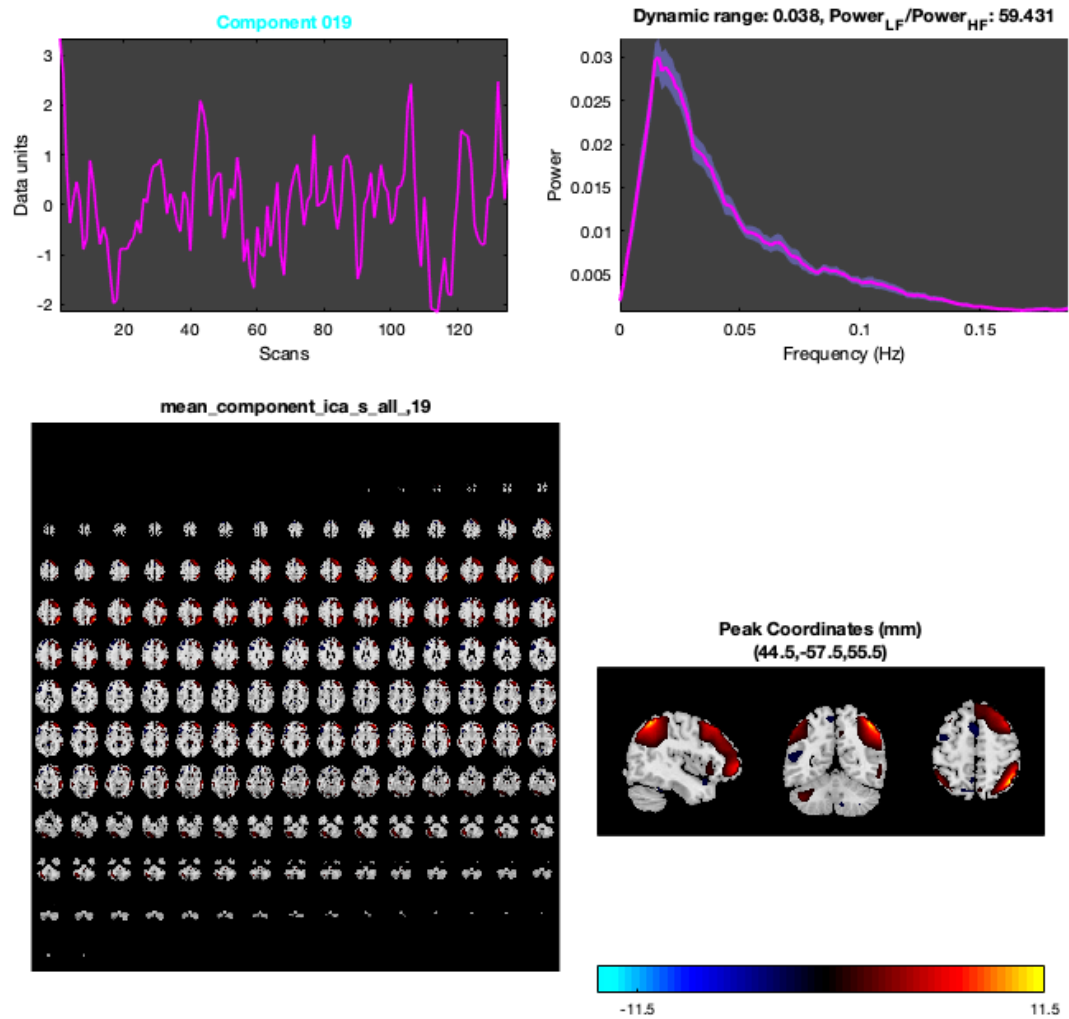

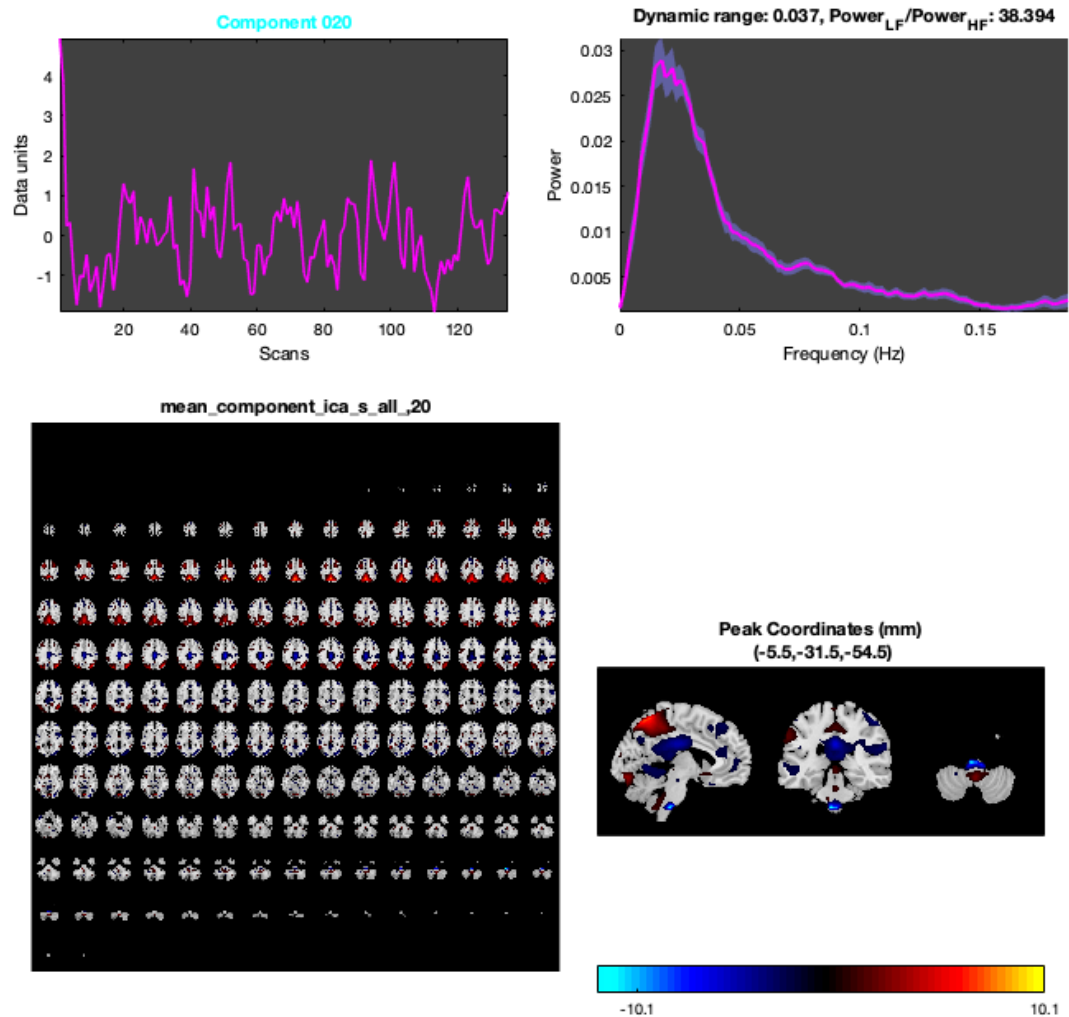

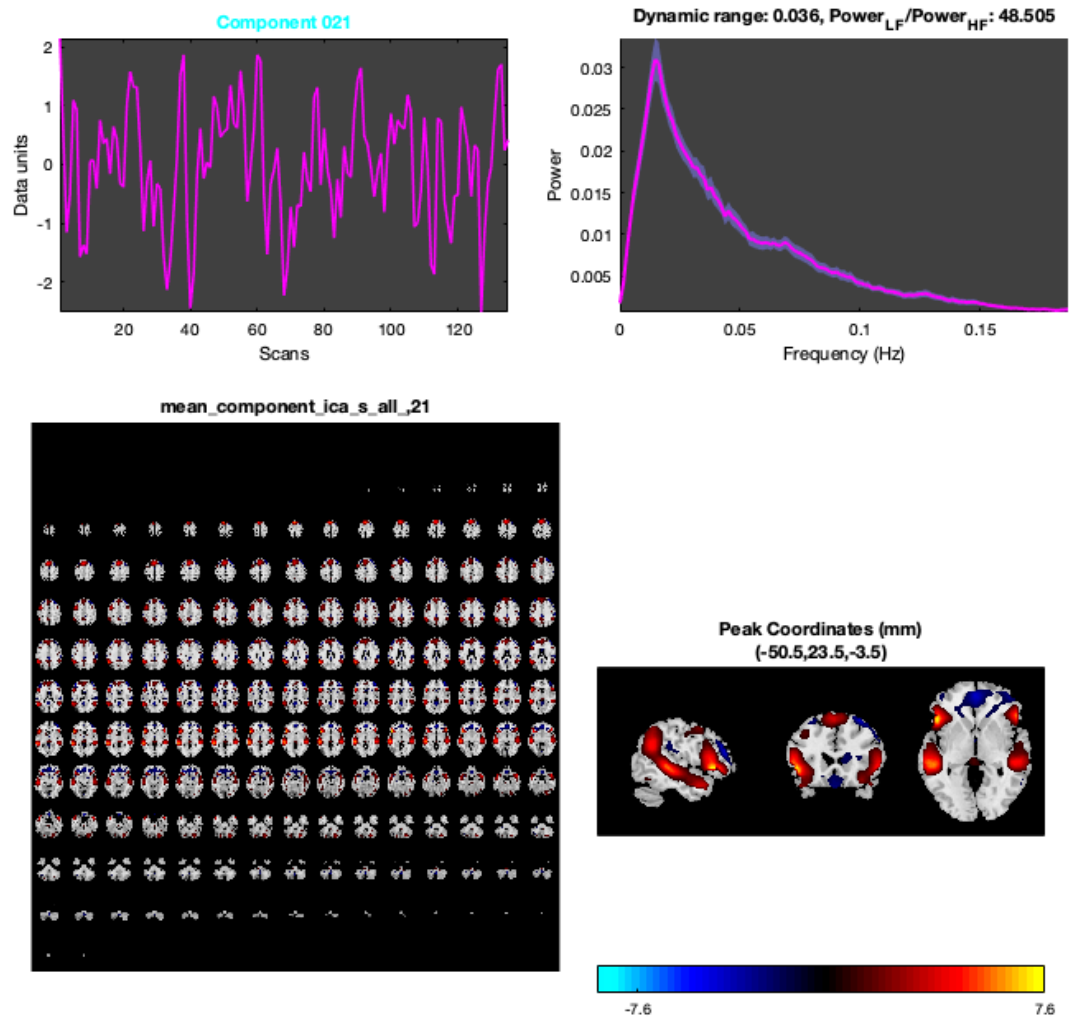

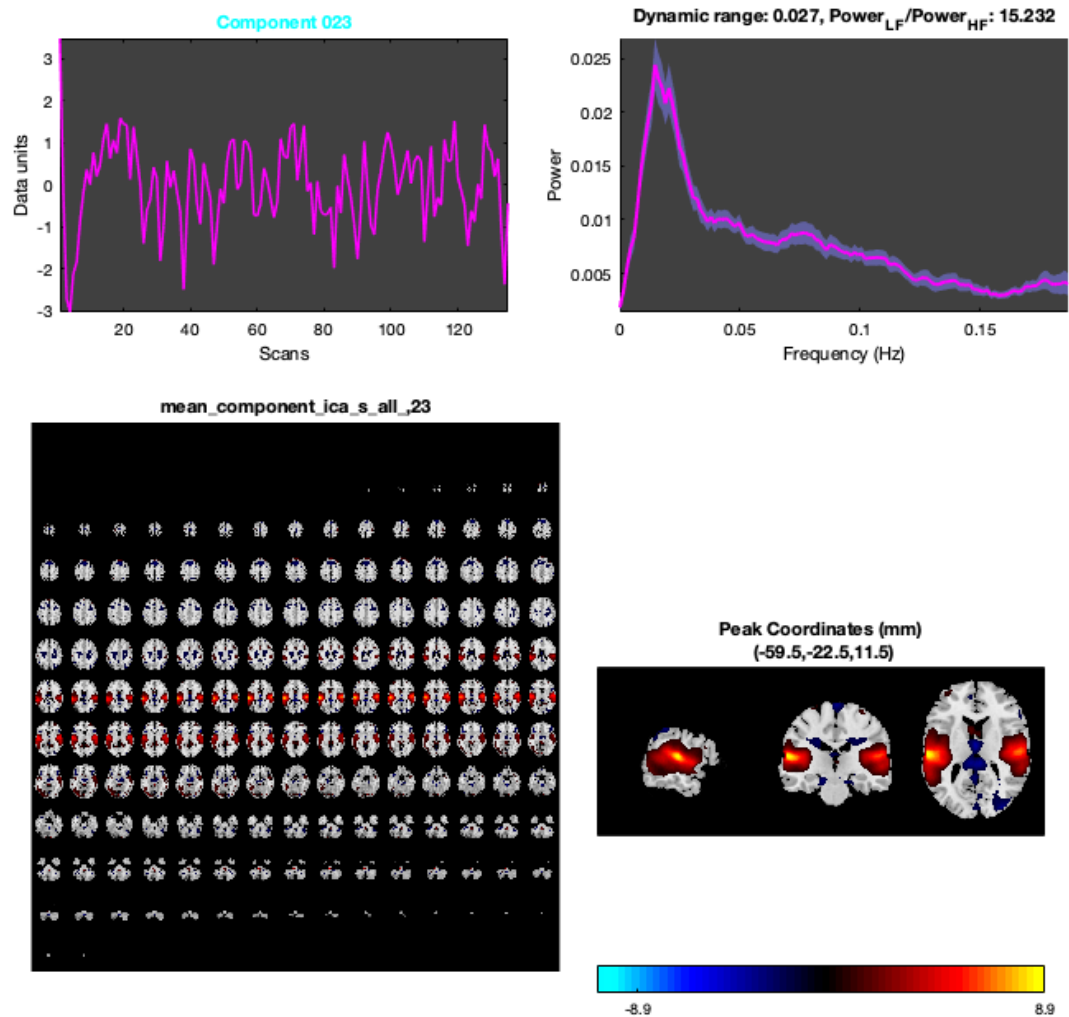

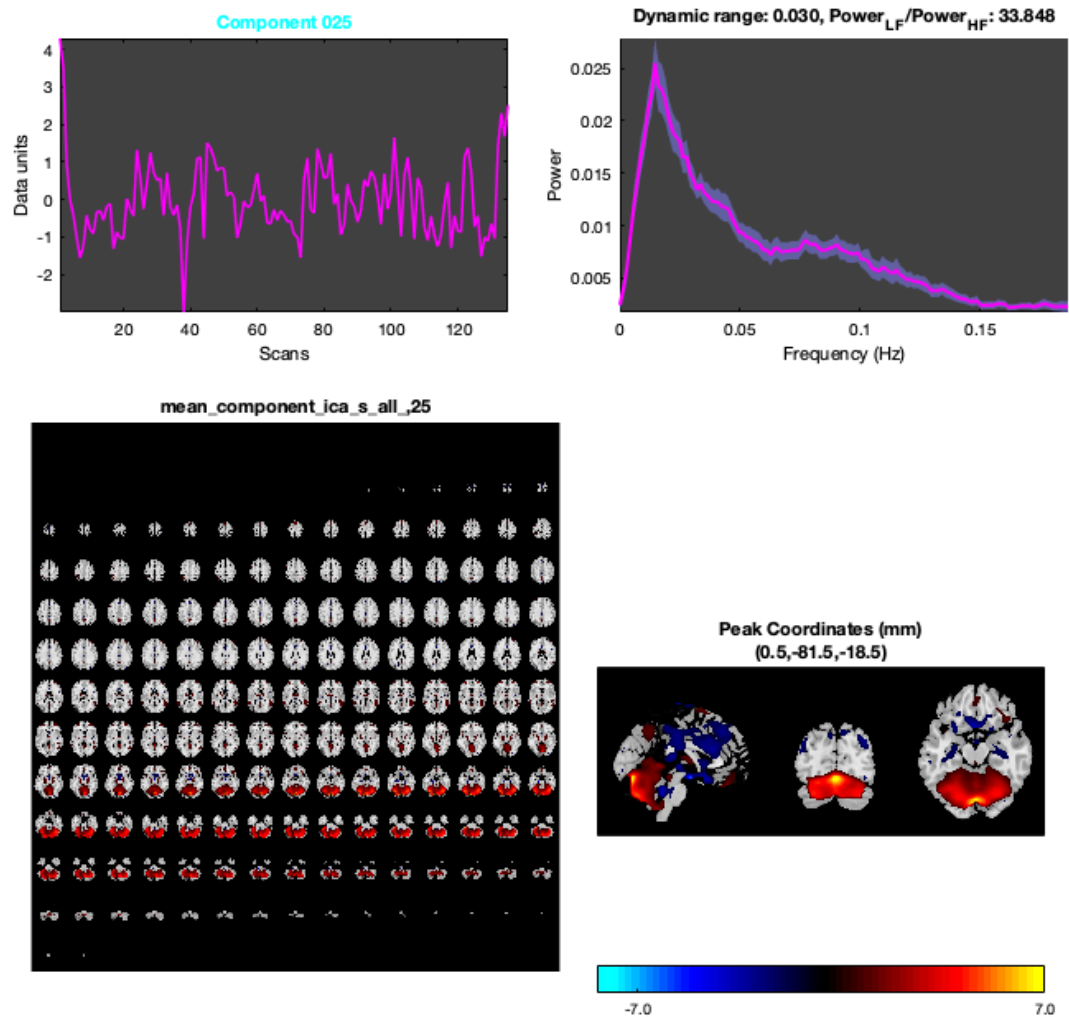

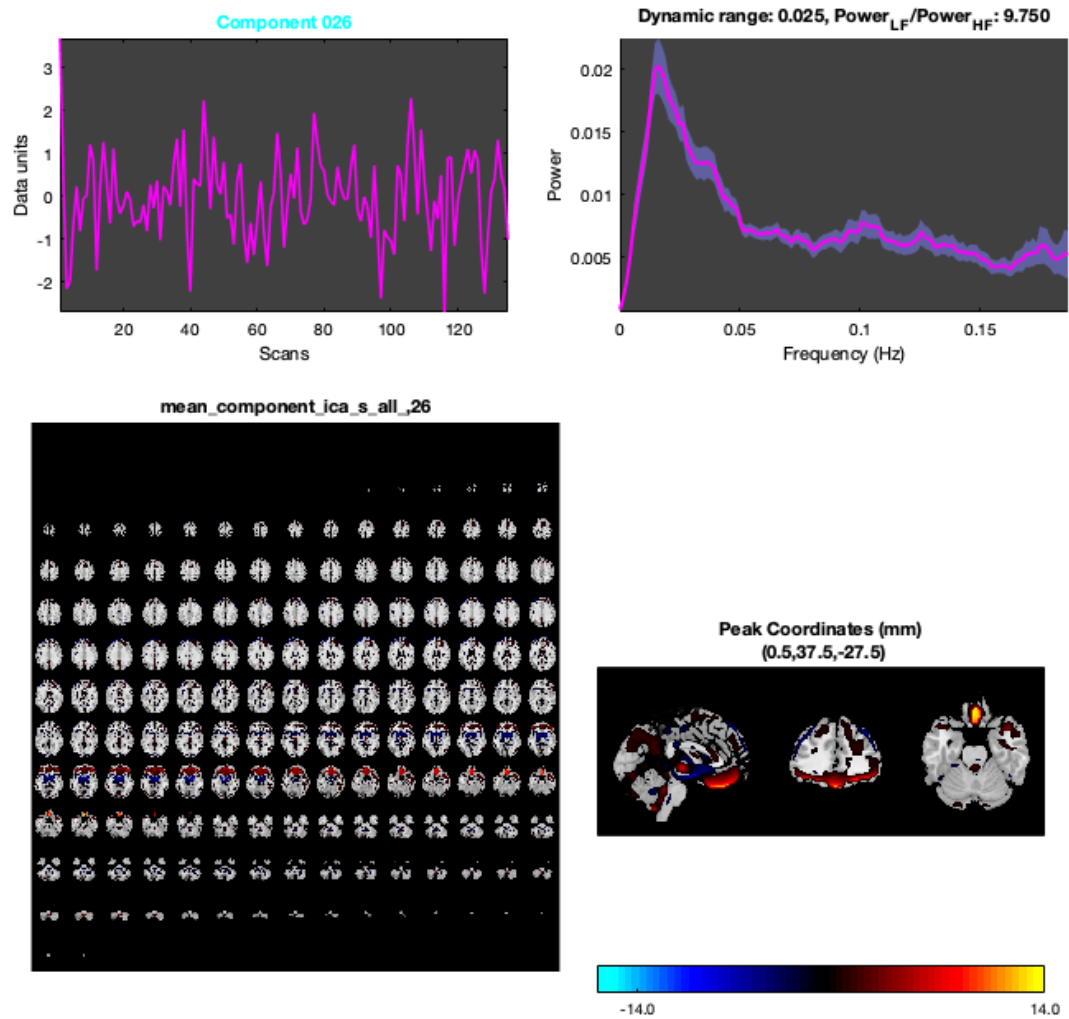

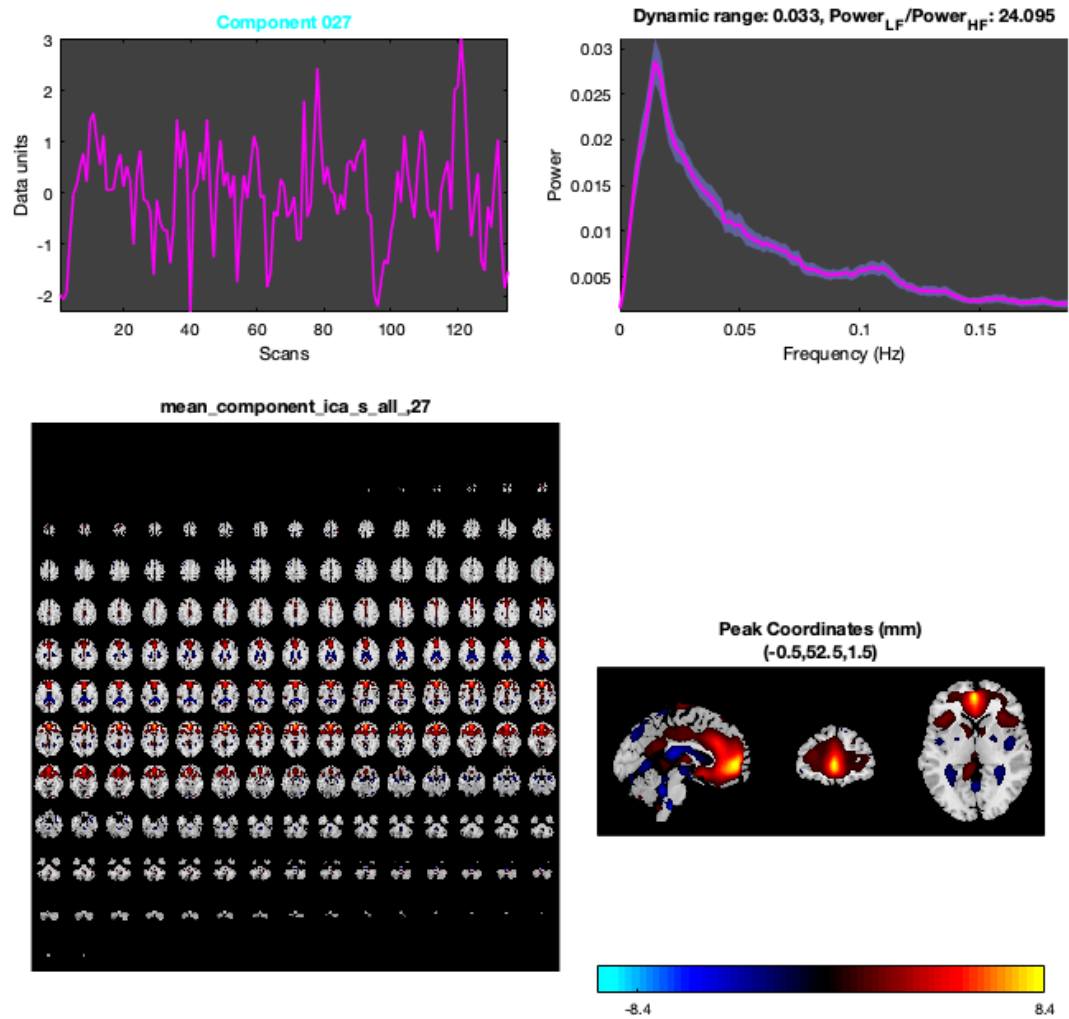

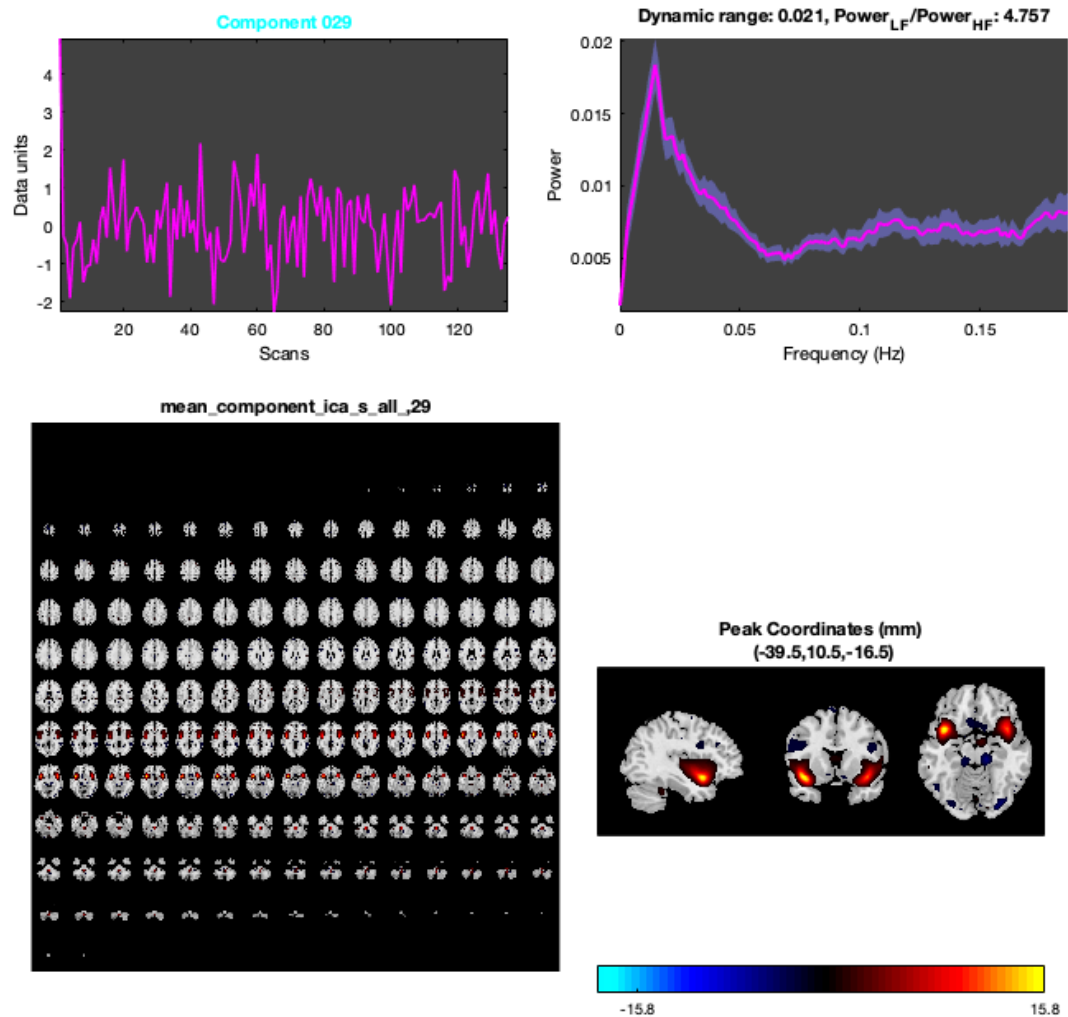

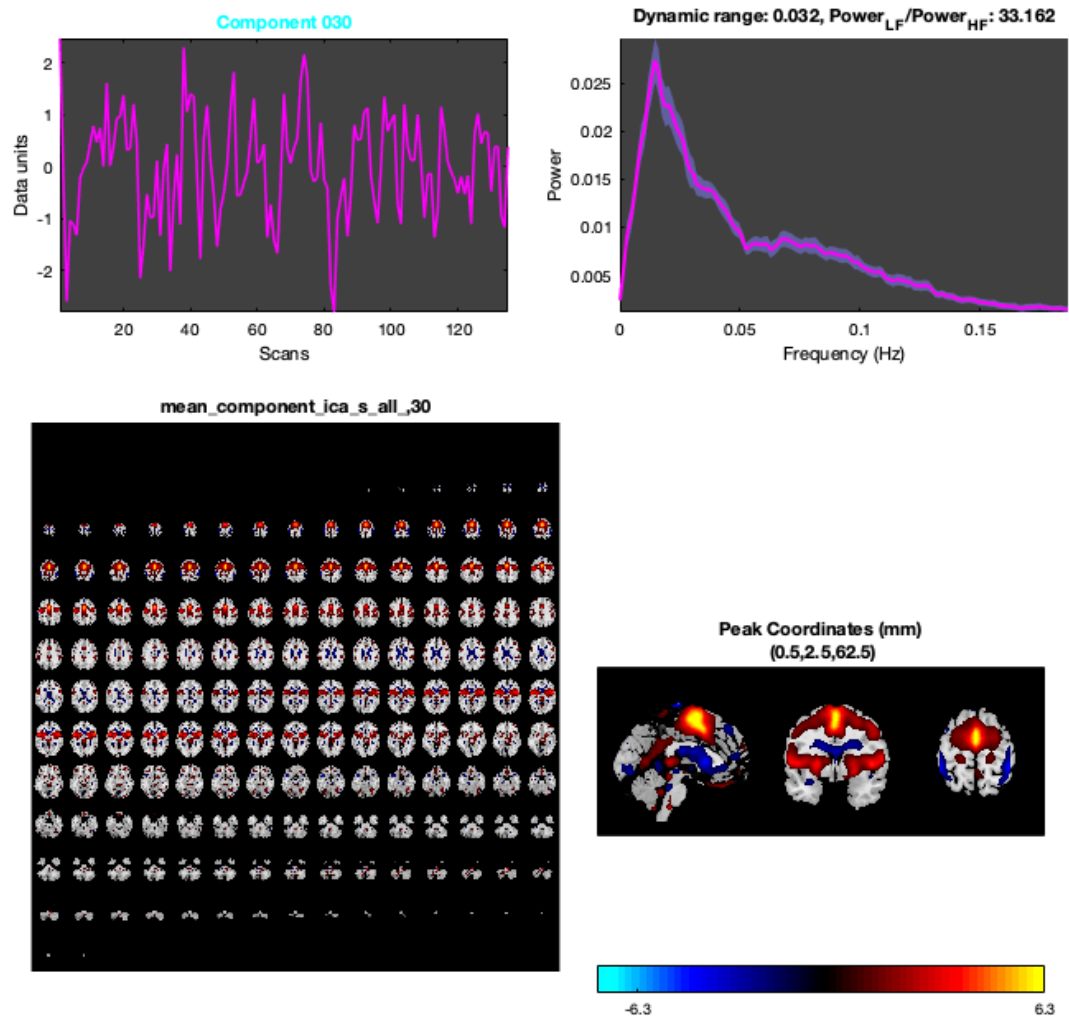

Supplement: Supplementary file 7 [file Data_Sheet_6.PDF]
